# Supplementary material for: In situ cell-surface conformation of the TCR-CD3 signaling complex
Source: EMBO Rep. 2024 Nov 7;25(12):26. doi: 10.1038/s44319-024-00314-3 (PMC11624261; doi:10.1038/s44319-024-00314-3)
Supplement: Supplementary file 1 — Appendix [file 44319_2024_314_MOESM1_ESM.pdf]

Appendix for "In situ cell-surface conformation of the TCR-CD3 signaling complex"

Table of Contents:

1) Appendix Figure S1. TCRβ and CD3ε expression plots of Cα DE loop mutants. Western blot analysis of Cα DE loop mutants – page 3

2) Appendix Figure S2. TCRβ and CD3ε expression plots of Cβ CC' loop mutants. Western blot analysis of Cβ CC' loop mutants – page 4

3) Appendix Figure S3. TCRβ and CD3ε expression plots of Cα AB loop mutants. Western blot analysis of Cα AB loop mutants – page 5

4) Appendix Figure S4. TCRβ and CD3ε expression plots of Cβ FG loop mutants. Western blot analysis of Cβ FG loop mutants – page 6

5) Appendix Figure S5. TCRβ and CD3ε expression plots of Cβ G strand mutants. Western blot analysis of Cβ G strand mutants – page 7

6) Appendix Figure S6. TCRβ and CD3ε expression plots of Cβ helix 3 mutants. Western blot analysis of Cβ helix 3 mutants – page 8

7) Appendix Figure S7. TCRβ and CD3ε expression plots of Cβ helix 4–F strand mutants. Western blot analysis of Cβ helix 4-F strand mutants – page 9

8) Appendix Figure S8. TCRβ and CD3ε expression plots of CD3δ mutants. Western blot analysis of CD3δ mutants – page 11

9) Appendix Figure S9. TCRβ and CD3ε expression plots of CD3γ mutants. Western blot analysis of CD3γ mutants – page 12

10) Appendix Figure S10. Crosslinking in purified TCR-CD3 complex with and without cholesterol, digitonin – page 13

11) Appendix Figure S11. Surface charge comparison of TCR-CD3 interface residues between human and mouse species – page 14

12) Appendix Figure S12. Overlay of crosslink-guided and cryoEM TCR-CD3 structures – page 15

13) Appendix Figure S13. Crosslinking in signaling-reducing mutants – page 16

14) Appendix Figure S14. Fixing TCR-CD3 complex via photo-crosslinking did not influence antigen binding – page 17

15) Appendix Figure S15. IE^k tetramer binding and activation assays – page 18

16) Appendix Figure S16. Crosslinking reveals lack of conformation change in the TCRα-CD3δ interface upon antigen binding – page 19

17) Appendix Figure S17. CD3 tetramer assays reveal no major TCR-CD3 subunits reorganization upon activation – page 20

18) Appendix Table S1. TCR regions and residues tested for crosslinking – page 20

19) Appendix Table S2. CD3 subunit regions and residues tested for crosslinking – page 22

20) Appendix Table S3. PDB entries used for generating TCR-CD3 complex structure – page 23

21) Appendix Table S4. Effect of pAzpa substitutions in individual components of the TCR-CD3 complex – page 24

22) Appendix Table S5. Crosslinking residues to subunit distances – page 25

23) Appendix Table S6. Thermodynamics values associated with mutations identified from the crosslink-guided model and cryo-EM structure – page 25

24) Appendix Table S7. Unpaired student t-test p values comparing activation potential between mutants and wild type – page 26

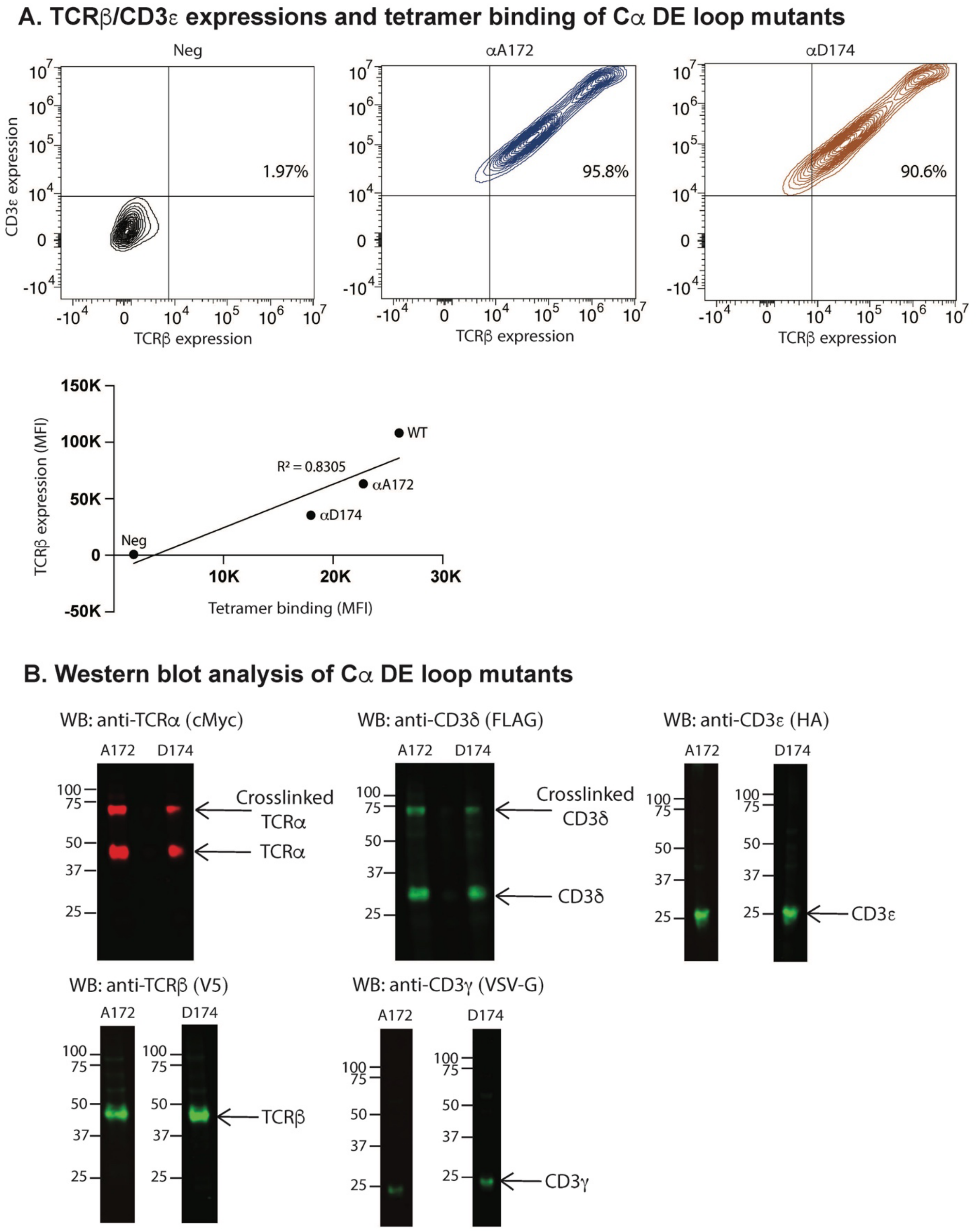

Appendix Figure S1. Related to Figure 2. CD3δ is in closer proximity to the TCR Cα DE loop. A) TCRβ and CD3ε expression plots of Cα DE loop mutants – αA172 and αD174 by flow cytometry. The percentage of cells positive for both TCRβ and CD3ε staining is indicated. Correlation plot between surface expression of TCRβ (MFI, stained with APC-conjugated H57-

597 antibody) and IE^K/MCC tetramer staining (MFI, IE^k/MCC-APC tetramer) for the mutants is provided. B) Western blot analysis of Cα DE loop mutants - αA172 and αD174. TCRα+CD3δ crosslinked bands for αA172 and αD174 are present around 75 kDa. The blot was stained with anti-TCRα (cMyc) antibody and mouse anti-CD3δ (FLAG); and separately for anti-CD3ε (HA), anti-TCRβ (V5) and anti-CD3γ (VSV-G). Anti-rabbit IRDye 680LT- and anti-mouse IRDye 800CW were used as secondary antibodies for detection.

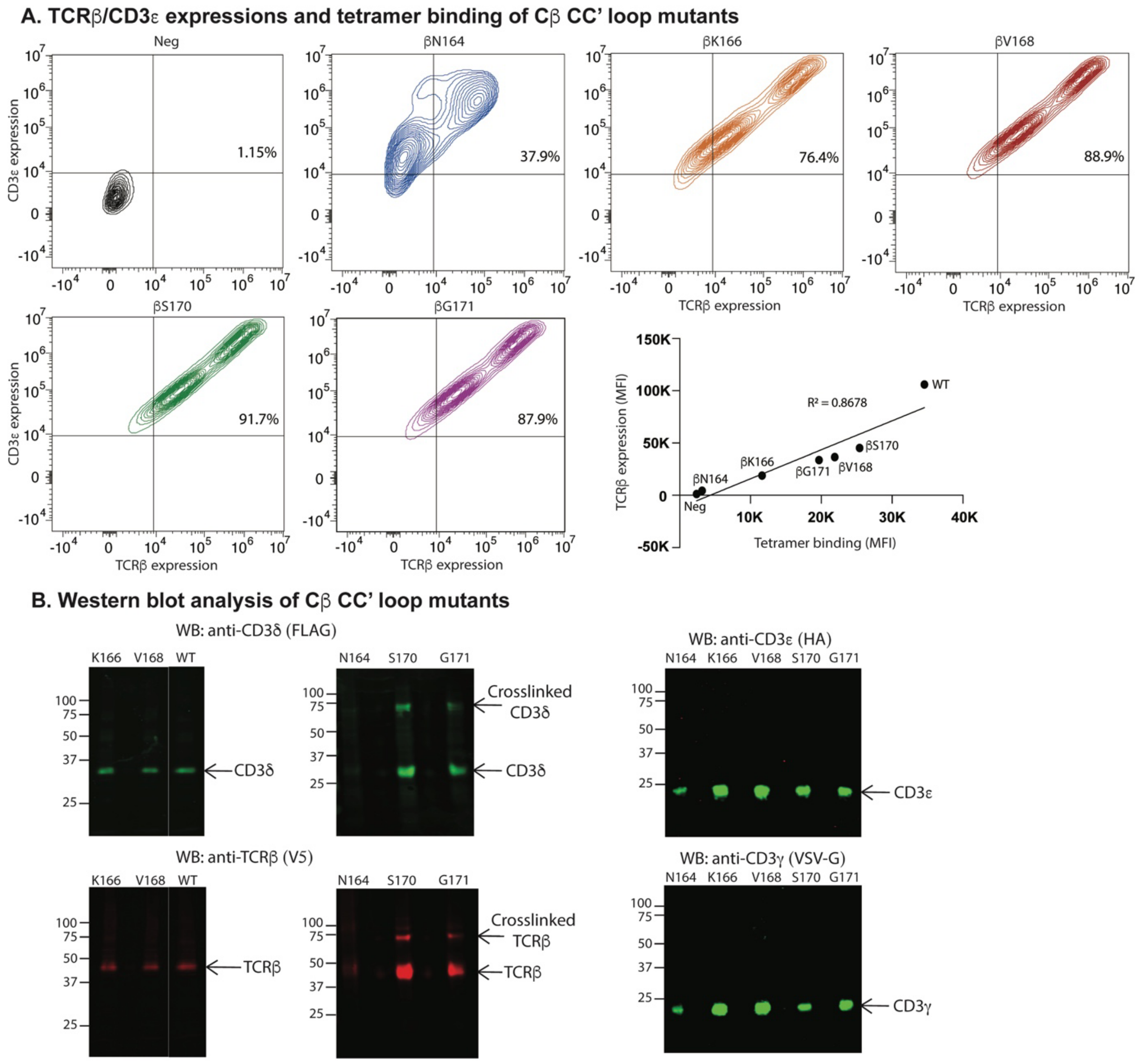

Appendix Figure S2. Related to Figure 2: CD3δ is in closer proximity to the TCR Cβ CC' loop. A) TCRβ and CD3ε expression plots of Cβ CC' loop mutants - βN164, βK166, βV168, βS170 and βG171 by flow cytometry. The percentage of cells positive for both TCRβ and CD3ε staining is indicated. Correlation plot between surface expression of TCRβ (MFI, stained with APC-conjugated H57-597 antibody) and IE^K/MCC tetramer staining (MFI, IE^k/MCC-APC tetramer) for the mutants is provided. B) Western blot analysis of Cβ CC' loop mutants - βN164, βK166, βV168, βS170 and βG171. TCRβ+CD3δ crosslinked bands for βS170 and βG171 are

present around 75 kDa. The blot was stained with anti-TCRβ (V5) antibody and anti-CD3δ (FLAG). Separate blots for the same mutants were stained with anti-CD3ε (HA) and anti-CD3γ (VSV-G). Anti-rabbit IRDye 680LT- and anti-mouse IRDye 800CW were used as secondary antibodies for detection.

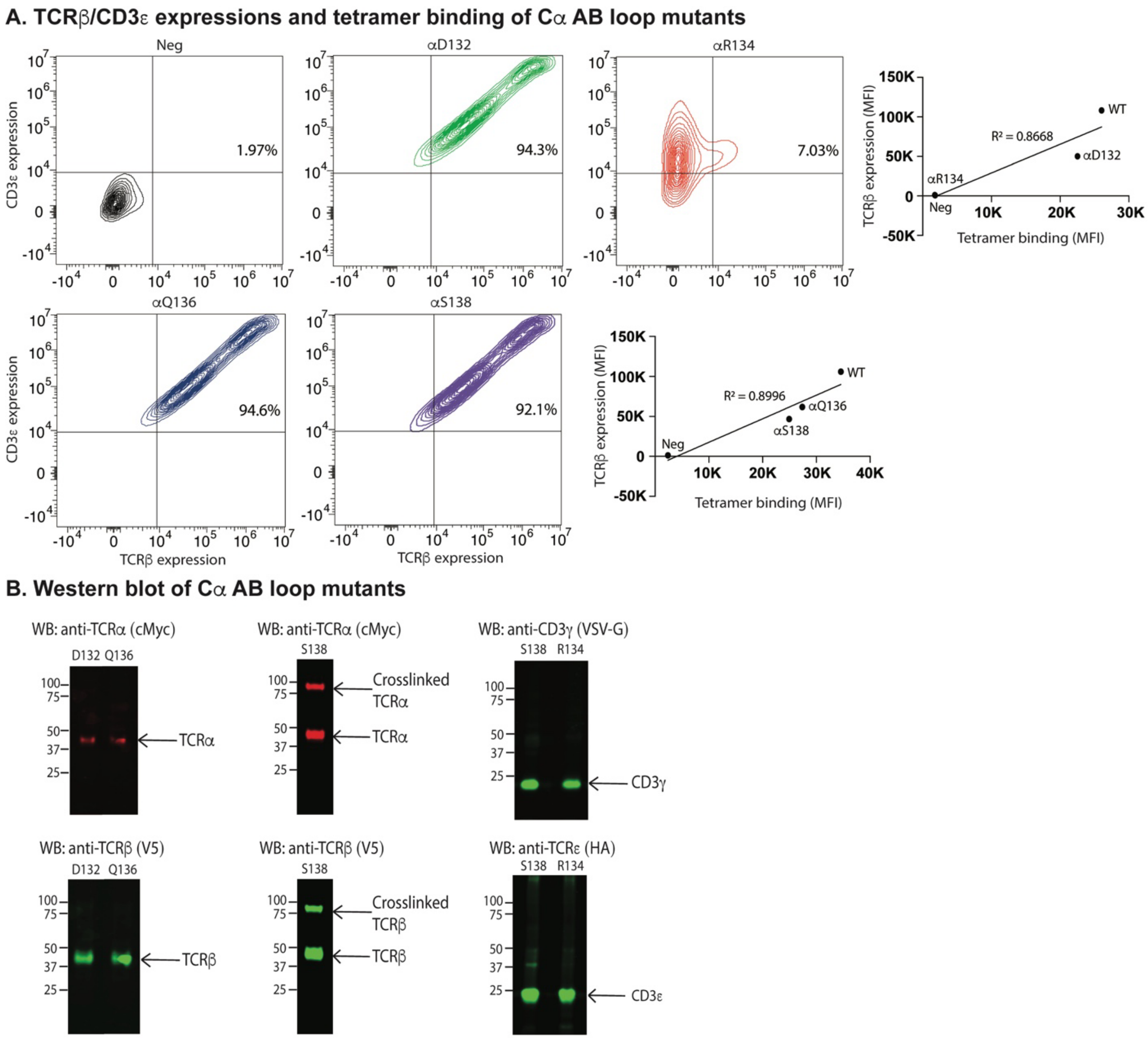

Appendix Figure S3. Related to Figure 2: Some regions of TCR Cα AB loop is in close proximity to TCRβ. A) TCRβ and CD3ε expression plots of Cα AB loop mutants - αD132, αR134, αQ136 and αS138 by flow cytometry. The percentage of cells positive for both TCRβ and CD3ε staining is indicated. Correlation plots between surface expression of TCRβ (MFI, stained with APC-conjugated H57-597 antibody) and IE^K/MCC tetramer staining (MFI, IE^k/MCC-APC tetramer) for the mutants are provided. B) Western blot analysis of Cα AB loop mutants - αD132, αR134, αQ136 and αS138. TCRα+TCRβ crosslinking band for αS138 is present above 75 kDa. αD132, αQ136 and αS138 blots were stained with anti-TCRα (cMyc) antibody and anti-TCRβ (V5). αR134 and αS138 blots were stained with anti-CD3γ (VSV-G) and anti-CD3ε (HA).

Anti-rabbit IRDye 680LT- and anti-mouse IRDye 800CW were used as secondary antibodies for detection.

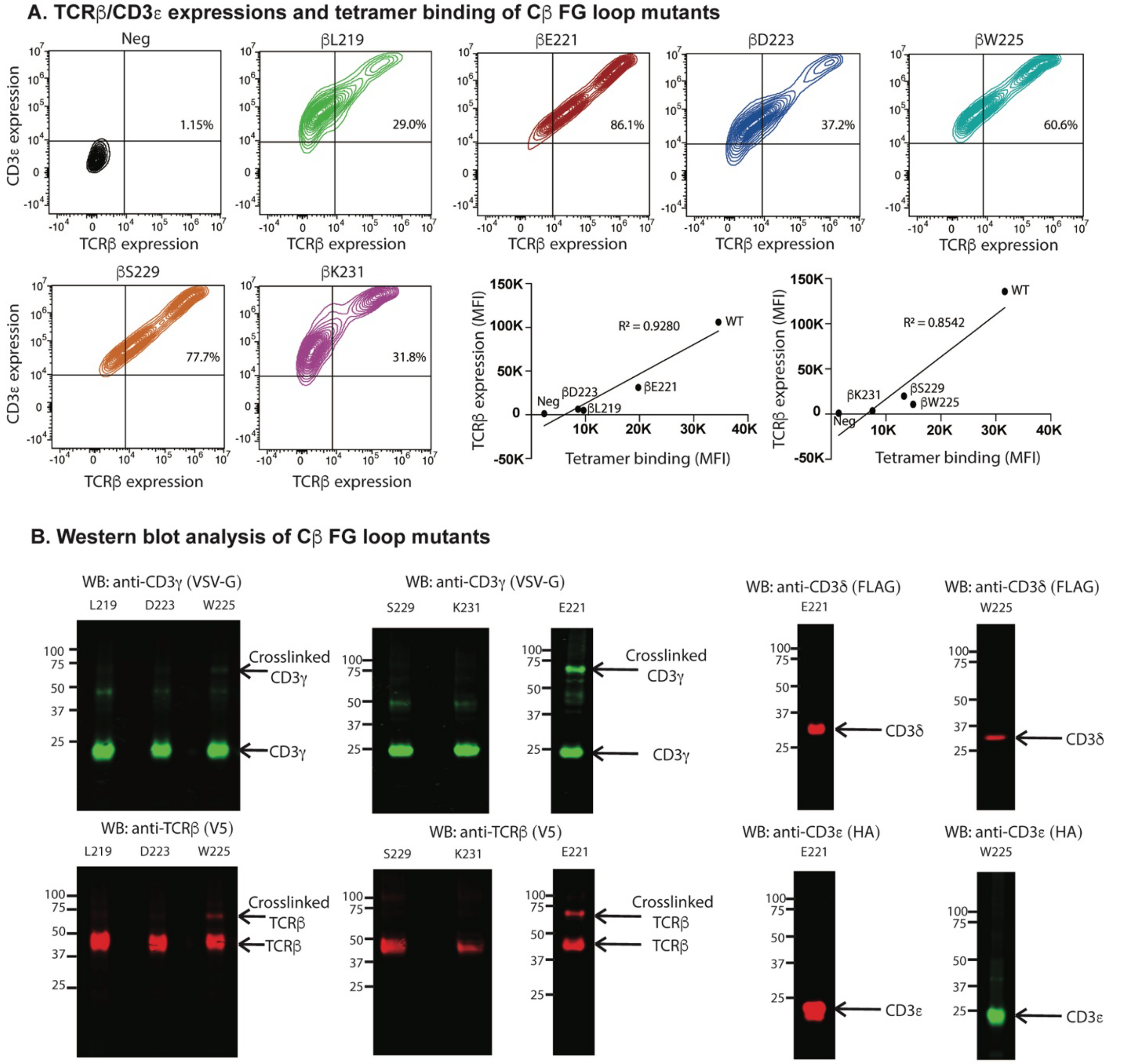

Appendix Figure S4. Related to Figure 2: CD3γ is in close proximity to the TCR Cβ FG loop. A) TCRβ and CD3ε expression plots of Cβ FG loop mutants - βL219, βE221, βD223, βW225, βS229 and βK231 by flow cytometry. The percentage of cells positive for both TCRβ and CD3ε staining is indicated. Correlation plots between surface expression of TCRβ (MFI, stained with APC-conjugated H57-597 antibody) and IE^{K}/MCC tetramer staining (MFI, IE^{k}/MCC-APC tetramer) for the mutants are provided. B) Western blot analysis of Cβ FG loop mutants - βL219, βE221, βD223, βW225, βS229 and βK231. TCRβ+CD3γ crosslinked bands for βE221 and βW225 are present below 75 kDa. The blots were stained with anti-TCRβ (V5) antibody and

anti-CD3γ (VSV-G). βE221 and βW225 were also stained for anti-CD3δ (FLAG) and anti-CD3ε (HA). Anti-rabbit IRDye 680LT- and anti-mouse IRDye 800CW were used as secondary antibodies for detection.

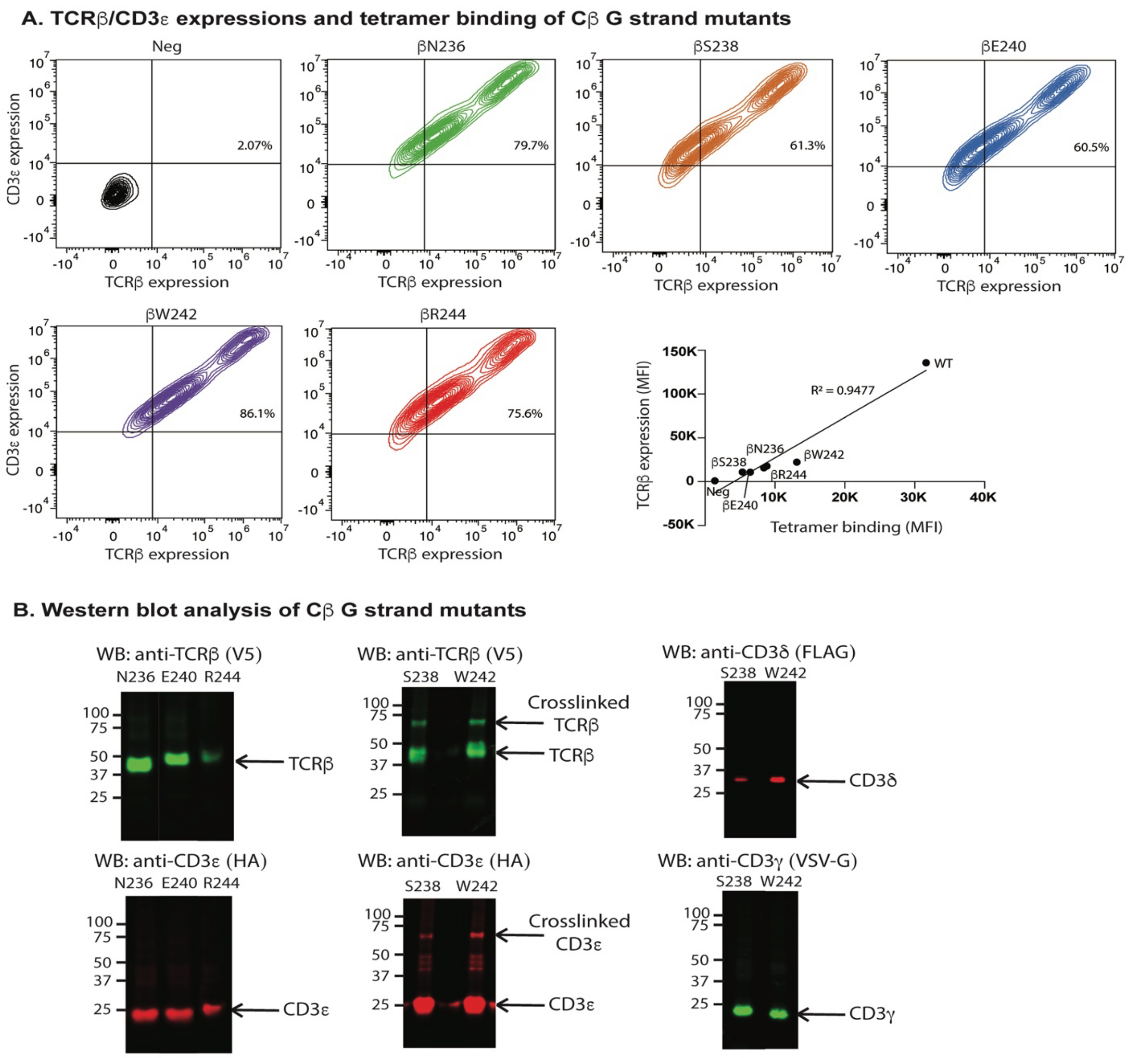

Appendix Figure S5. Related to Figure 2: CD3ε is in close proximity to the TCR Cβ G strand. A) TCRβ and CD3ε expression plots of Cβ G strand mutants - βN236, βS238, βE240, βW242 and βR244 by flow cytometry. The percentage of cells positive for both TCRβ and CD3ε staining is indicated. Correlation plot between surface expression of TCRβ (MFI, stained with APC-conjugated H57-597 antibody) and IE^{K}/MCC tetramer staining (MFI, IE^{k}/MCC-APC tetramer) for the mutants is provided. B) Western blot analysis of Cβ G strand mutants - βN236, βS238, βE240, βW242 and βR244. TCRβ+CD3ε crosslinked bands for βS238, βE240 and βW242 are present below 75 kDa. βN236, βS238, βE240, βW242 and βR244 blots were stained with anti-TCRβ (V5) antibody and anti-CD3ε (HA). Separate blots of βS238 and βW242 were

stained with anti-CD3γ (FLAG) antibody and anti-CD3γ (VSV-G). Anti-rabbit IRDye 680LT- and anti-mouse IRDye 800CW were used as secondary antibodies for detection.

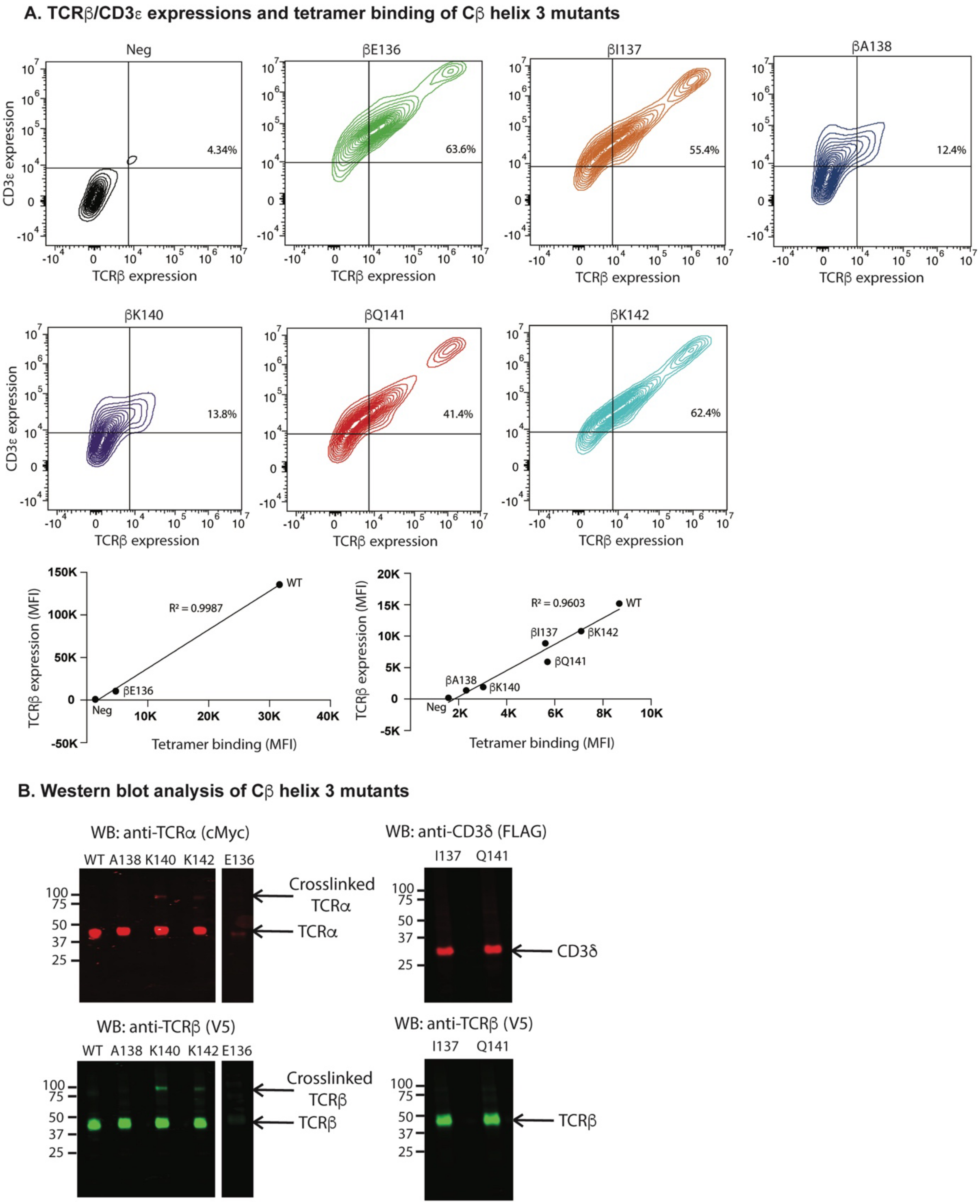

Appendix Figure S6. Related to Figure 2: Cβ helix 3 residues are in close proximity to the TCRα subunit. A) TCRβ and CD3ε expression plots of Cβ helix 3 mutants – βE136, βI137, βA138, βK140, βQ141 and βK142 by flow cytometry. The percentage of cells positive for both

TCRβ and CD3ε staining is indicated. Correlation plots between surface expression of TCRβ (MFI, stained with APC-conjugated H57-597 antibody) and IE^{K}/MCC tetramer staining (MFI, IE^{k}/MCC-APC tetramer) for the mutants are provided. B) Western blot analysis of Cβ helix 3 mutants - βE136, βI137, βA138, βK140, βQ141 and βK142. TCRβ+TCRα crosslinked bands or βK140 and βK142 are present between 75 and 100 kDa. WT, βE136, βA138, βK140 and βK142 blots were stained with anti-TCRα (cMyc) antibody and anti-TCRβ (V5). βI137 and βQ141 blots were stained with anti-TCRβ (V5) antibody and anti-CD3δ (FLAG). Anti-rabbit IRDye 680LT- and anti-mouse IRDye 800CW were used as secondary antibodies for detection.

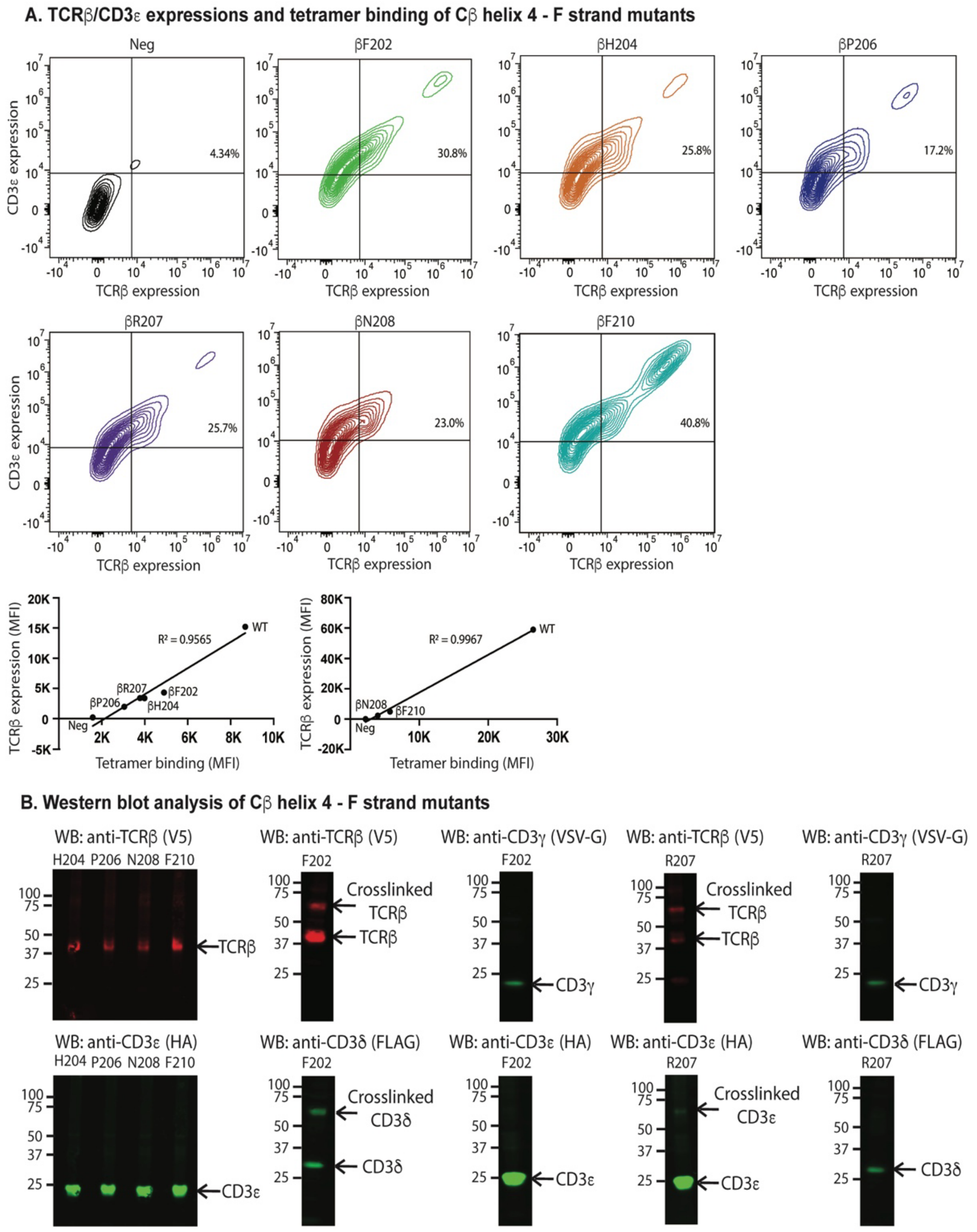

Appendix Figure S7. Related to Figure 2: CD3δε is in close proximity to the TCR Cβ helix 4-F strand region. A) TCRβ and CD3ε expression plots of Cβ helix 4–F strand mutants – βF202, βH204, βP206, βR207, βN208 and βF210 by flow cytometry. The percentage of cells positive for both TCRβ and CD3ε staining is indicated. Correlation plots between surface expression of TCRβ (MFI, stained with APC-conjugated H57-597 antibody) and IE^K/MCC tetramer staining (MFI, IE^k/MCC-APC tetramer) for the mutants are provided. B) Western blot analysis of Cβ helix 4-F strand mutants - βF202, βH204, βP206, βR207, βN208 and βF210. TCRβ+CD3δ and TCRβ+CD3ε crosslinked bands for βF202 and βR207, are present below 75 kDa. βH204, βP206, βR207, βN208 and βF210 blots were stained with anti-TCRβ (V5) antibody and anti-CD3ε (HA). βF202 blot was stained with anti-TCRβ (V5) antibody and anti-CD3δ (FLAG). Separate βF202 blots were stained with anti-CD3γ (VSV-G) and anti-CD3ε (HA). Separate βR207 blots were stained with anti-CD3γ (VSV-G) and anti-CD3δ (FLAG). Anti-rabbit IRDye 680LT- and anti-mouse IRDye 800CW were used as secondary antibodies for detection.

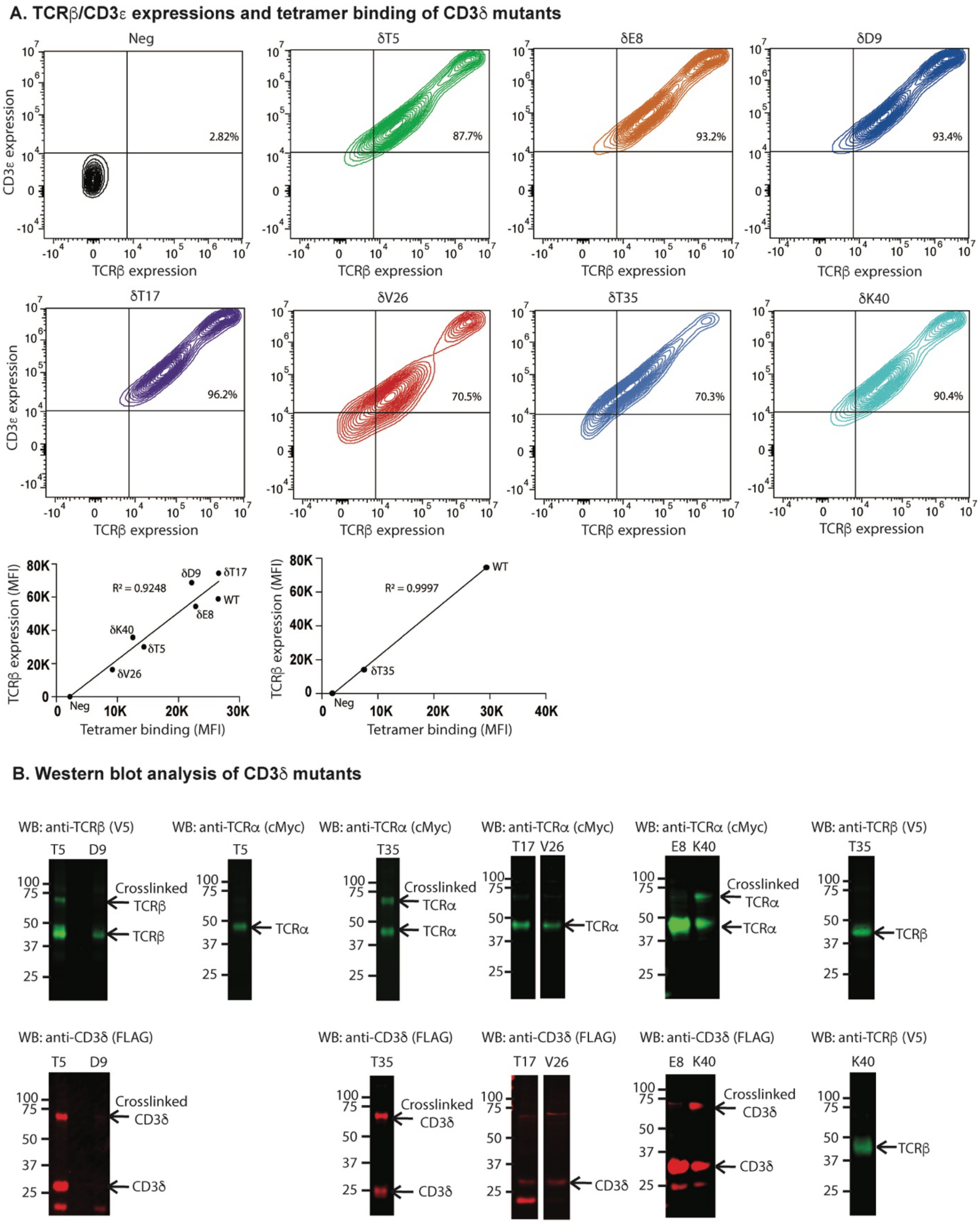

Appendix Figure S8. Related to Figure 3: TCRα is in close proximity to CD3δ E strand, CD3δ EF loop and TCRβ is in close proximity to CD3δ A strand. A) TCRβ and CD3ε

expression plots of CD3δ mutants – A strand: T5; AB loop: E8, D9; BC loop: T17; CD loop: V26; E strand: T35; and EF loop: K40 by flow cytometry. The percentage of cells positive for both TCRβ and CD3ε staining is indicated. Correlation plots between surface expression of TCRβ (MFI, stained with APC-conjugated H57-597 antibody) and IE^K/MCC tetramer staining (MFI, IE^k/MCC-APC tetramer) for the mutants are provided. Western blot analysis revealed CD3δ T5 crosslinked with TCRβ and CD3δ T35 and K40 crosslinked with TCRα with crosslinking bands present below 75 kDa. δE8, δT35 and δK40 were stained with anti-TCRα (cMyc) antibody and anti-CD3δ (FLAG). δT5 and δD9 were stained with anti-TCRβ (V5) and anti-CD3δ (FLAG). δT17 and δV26 were stained with anti-TCRα (cMyc) and anti-CD3δ (FLAG). Additionally, separate δT5 blot was stained with anti-TCRα (cMyc) and δT35 and δK40 blots were stained with anti-TCRβ (V5). Anti-rabbit IRDye 680LT- and anti-mouse IRDye 800CW were used as secondary antibodies for detection.

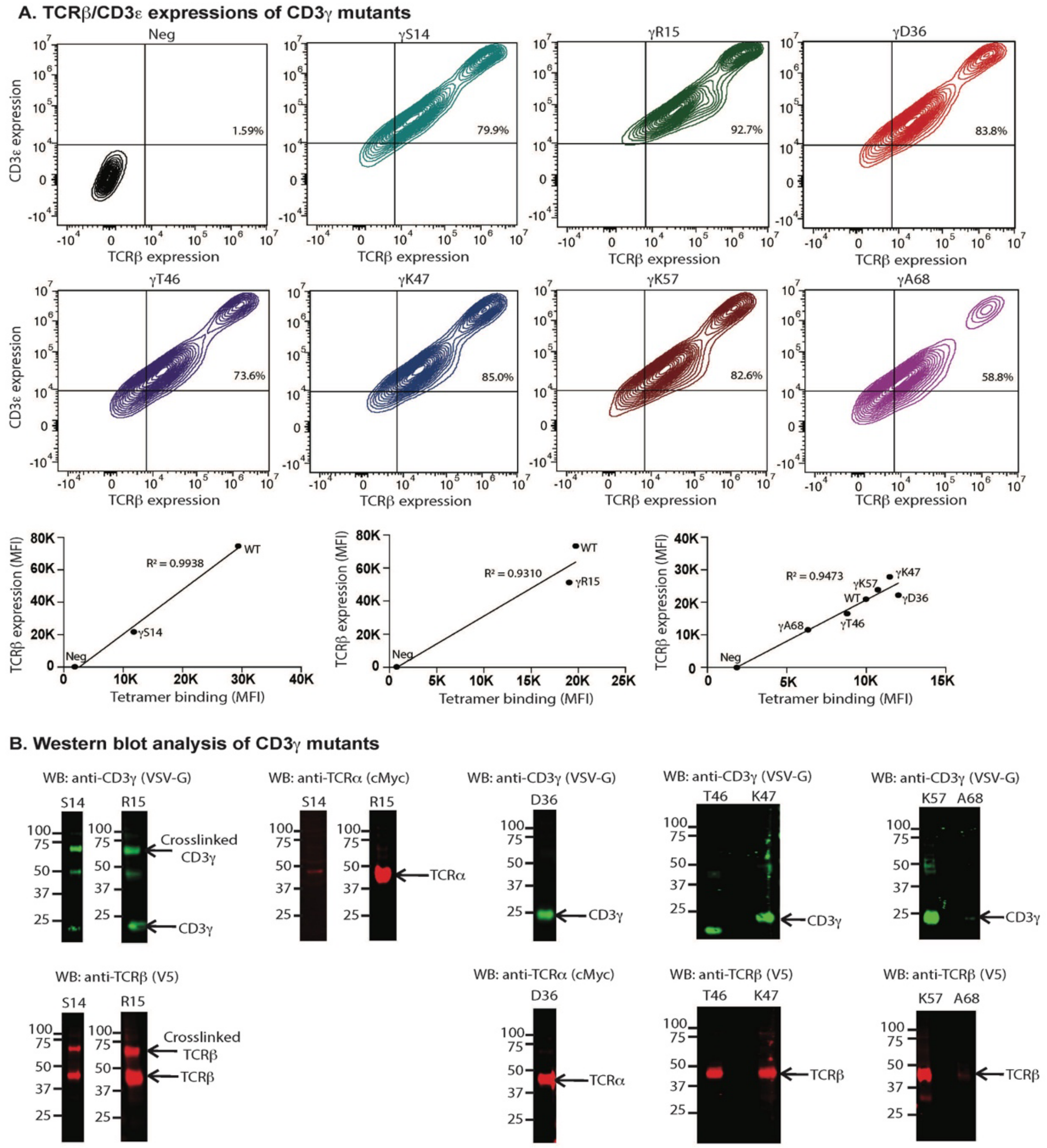

Appendix Figure S9. Related to Figure 3: TCRβ is in close proximity to the CD3γ AB loop. A) TCRβ and CD3ε expression plots of CD3γ mutants – AB loop: S14, R15; CD loop: D36; DE loop: T46, K47; EF loop: K57 and FG loop: A68 by flow cytometry. The percentage of cells positive for both TCRβ and CD3ε staining is indicated. Correlation plots between surface expression of TCRβ (MFI, stained with APC-conjugated H57-597 antibody) and IE^K/MCC tetramer staining (MFI, IE^k/MCC-APC tetramer) for the mutants are provided. B) Western blot analysis revealed CD3γ S14 and R15 crosslinked with TCRβ with crosslinking bands present below 75 kDa. S14, R15, K57 and A68 were stained with anti-CD3γ (VSV-G) and anti-TCRβ (V5) antibody. T46 and K47 were stained with anti-CD3γ (VSV-G) and anti-CD3ε (HA). D36 was stained with anti-CD3γ (VSV-G) and anti-TCRα (cMyc). Separate γS14 and γR15 bands were stained with anti-TCRα (cMyc). Anti-rabbit IRDye 680LT- and anti-mouse IRDye 800CW were used as secondary antibodies for detection.

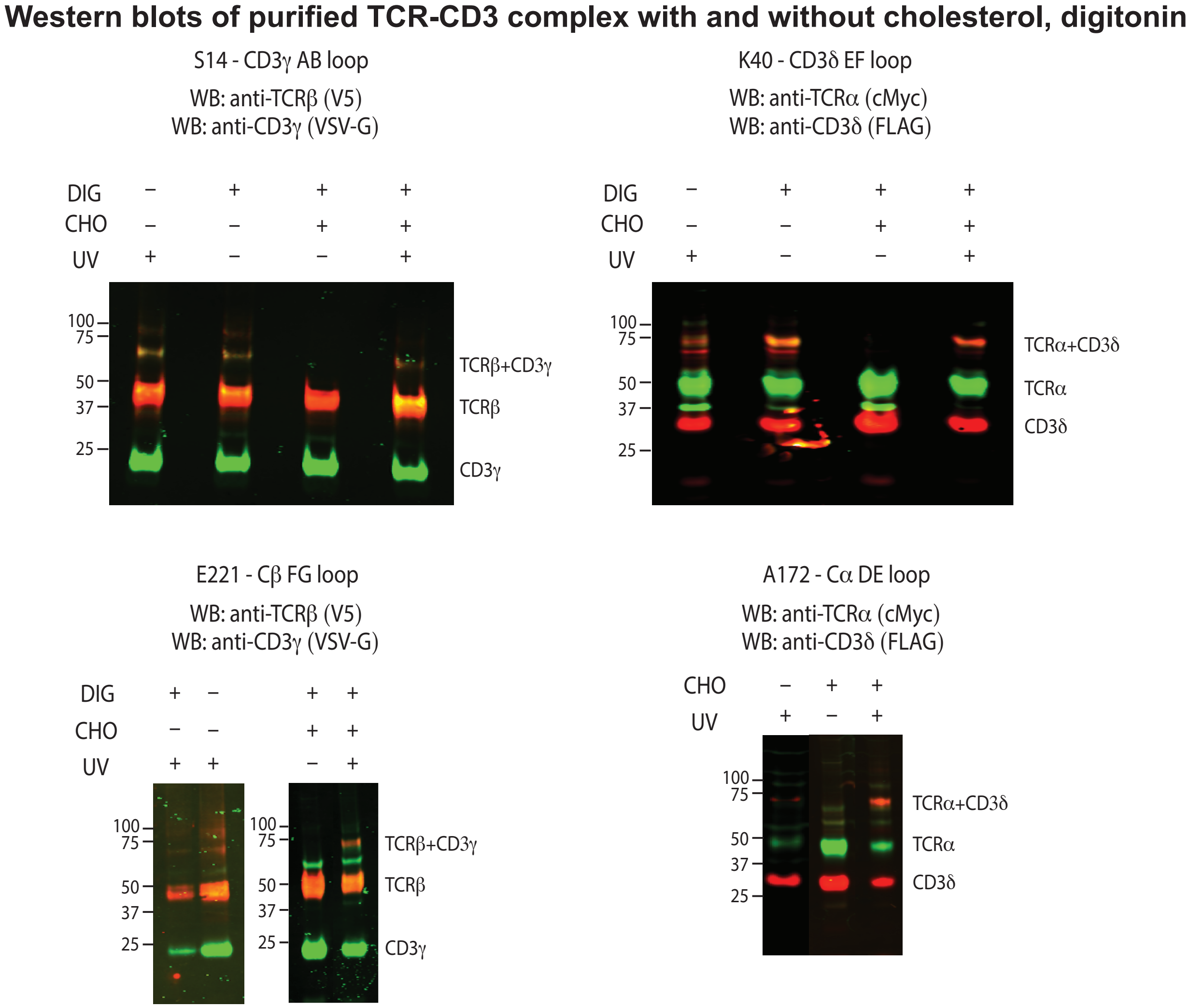

Appendix Figure S10: Crosslinking in purified TCR-CD3 complex with and without cholesterol, digitonin. Western blots of purified mutant TCR-CD3 complexes –γS14, δK40, βE221, and αA172 in the presence and absence of cholesterol and digitonin during purification

process. ‘DIG’ represents digitonin and ‘CHO’ represents cholesterol (cholesteryl hemisuccinate). TCRβ+CD3γ crosslinked bands for βE221 and gS14 are present below 75 kDa. Both blots were stained with anti-TCRβ (V5) antibody and anti-CD3γ (VSV-G). TCRα+CD3δ crosslinked bands for δK40 and αA172 are present below 75 kDa. Both blots were stained with anti-TCRα (cMyc) antibody and anti-CD3δ (FLAG). Anti-rabbit IRDye 680LT- and anti-mouse IRDye 800CW were used as secondary antibodies for detection.

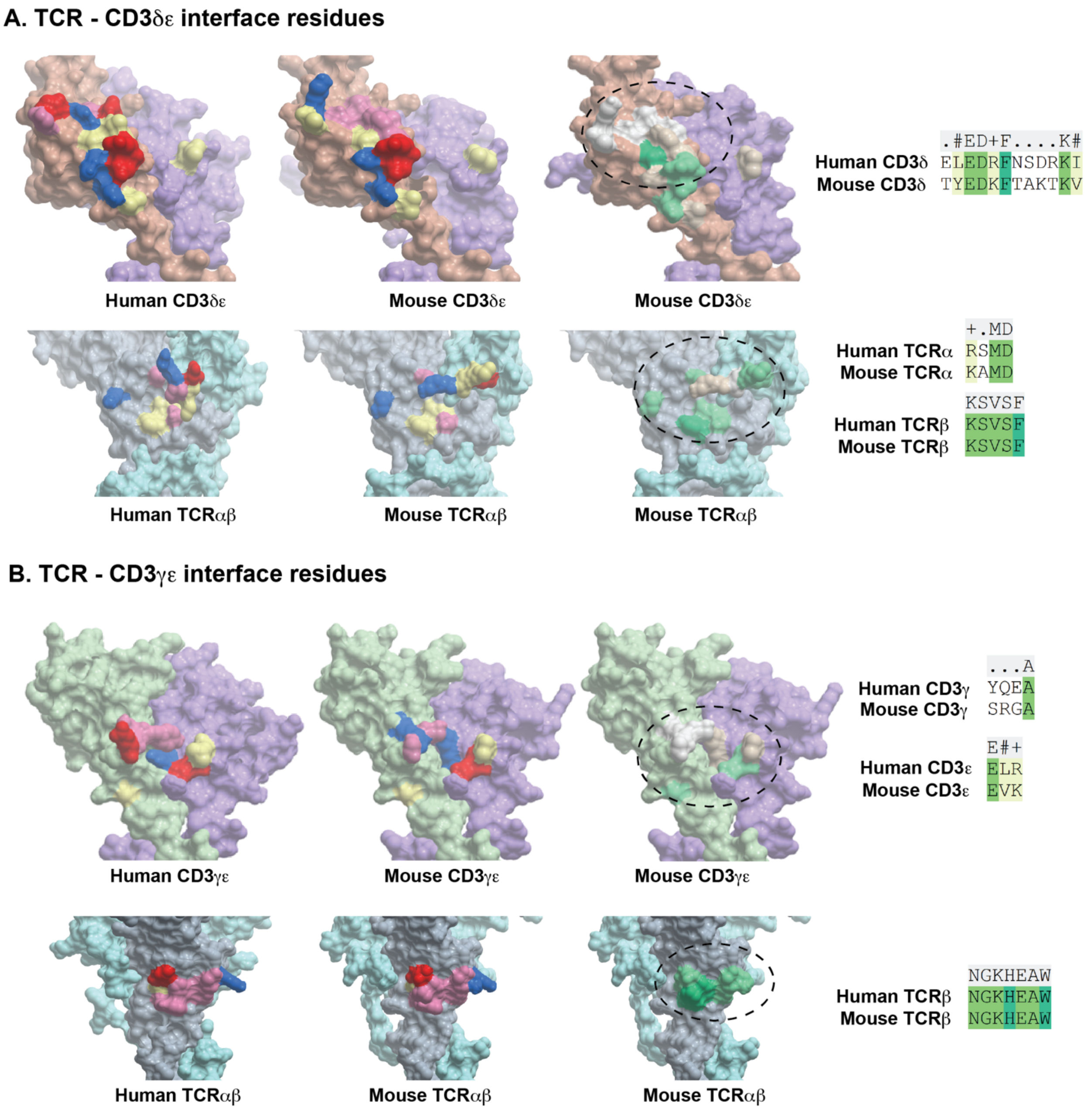

Appendix Figure S11. Related to Figure 4: Comparison of surface charges of TCR-CD3 interface residues between human and mouse species. Positive charges are indicated in red, negative charges in blue. Green residues indicate identical residues. Overall, there is better conservation in the TCR interface between human and mouse than CD3δε and CD3γε interfaces.

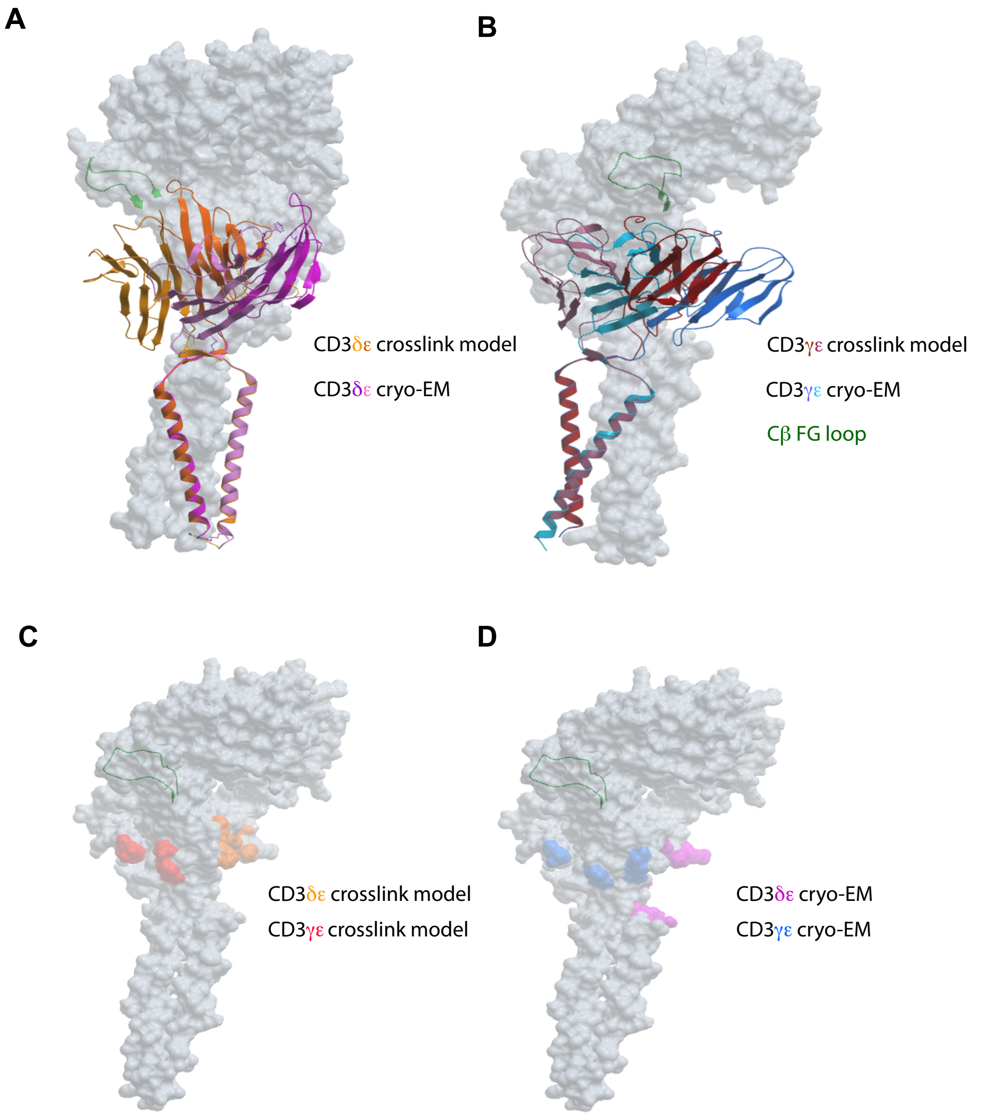

Appendix Figure S12. Related to Figure 4: Overlay of crosslink-guided and cryoEM TCR-CD3 structures. A) Overlay of TCR-CD3δε crosslink (orange) and cryoEM (magenta) structures, B) Overlay of TCR-CD3γε crosslink (red) and cryoEM (blue) structures with TCR indicated in surface (grey) representation. C) The CD3δε (orange) and CD3γε (red) interface residues located on the TCR (grey) in the crosslink model. D) The CD3δε (magenta) and CD3γε (blue) interface residues located on the TCR (grey) in the cryo-EM structure.

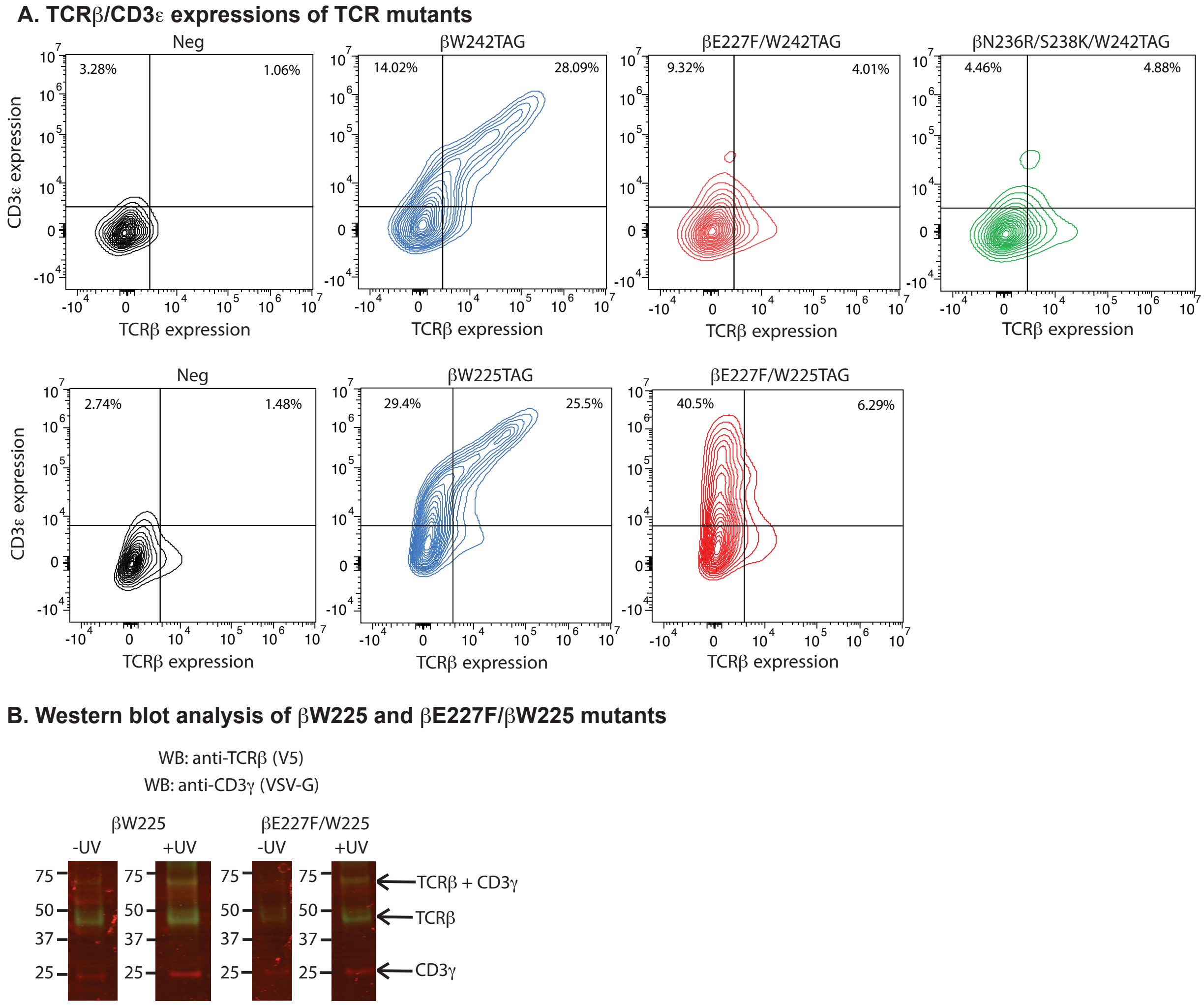

Appendix Figure S13. Related to Figure 5. Crosslinking in signaling-reducing mutants. A) TCRβ and CD3ε expression plots of TCR mutants with signaling reducing mutations – Negative, βW225TAG, βE227F/W242TAG, βN236R/S238K/W242TAG, βW225TAG and βE227F/W225TAG by flow cytometry. The percentage of cells positive for CD3ε and for both TCRβ and CD3ε staining is indicated. B) Western blot analysis of TCR mutants - βW225TAG and βE227F/W225TAG with and without UV irradiation. TCRβ+CD3γ crosslinked bands for are present below 75 kDa in the UV irradiated lane. The blots were stained with anti-TCRβ (V5) antibody and anti-CD3γ (VSV-G). Anti-mouse IRDye 680RD- and anti-rabbit IRDye 800CW were used as secondary antibodies for detection.

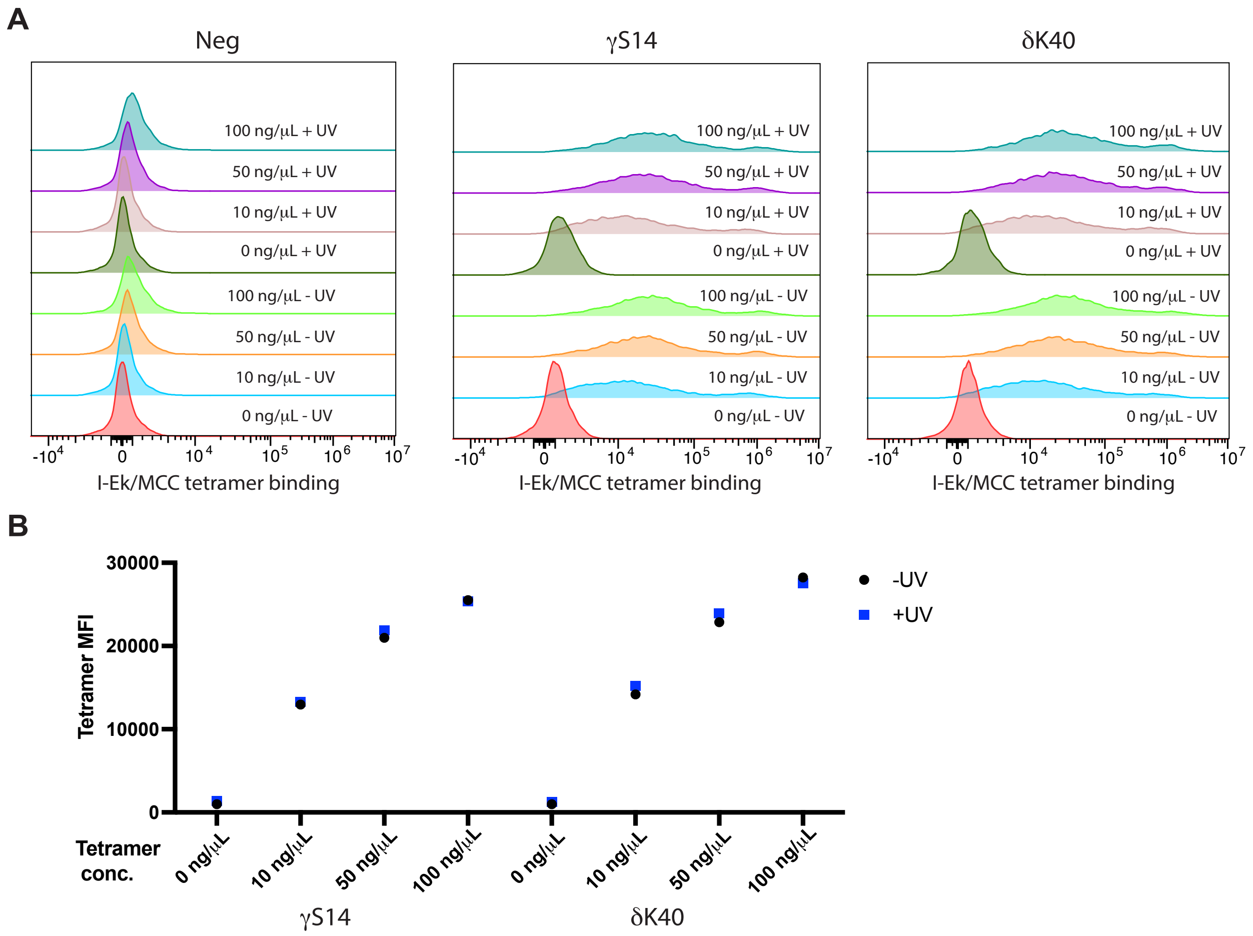

Appendix Figure S14. Related to Figure 6. Fixing TCR-CD3 complex via photo-crosslinking did not influence antigen binding. A) Histograms of IE^k/MCC-APC tetramer binding at indicated concentrations to 293T cells untransfected (left), transfected with γS14 (middle) and δK40 (right) TCR-CD3 complexes with and without UV-irradiation. B) Plot of tetramer binding MFI versus concentration of tetramer used with (blue) and without (black) UV-irradiation.

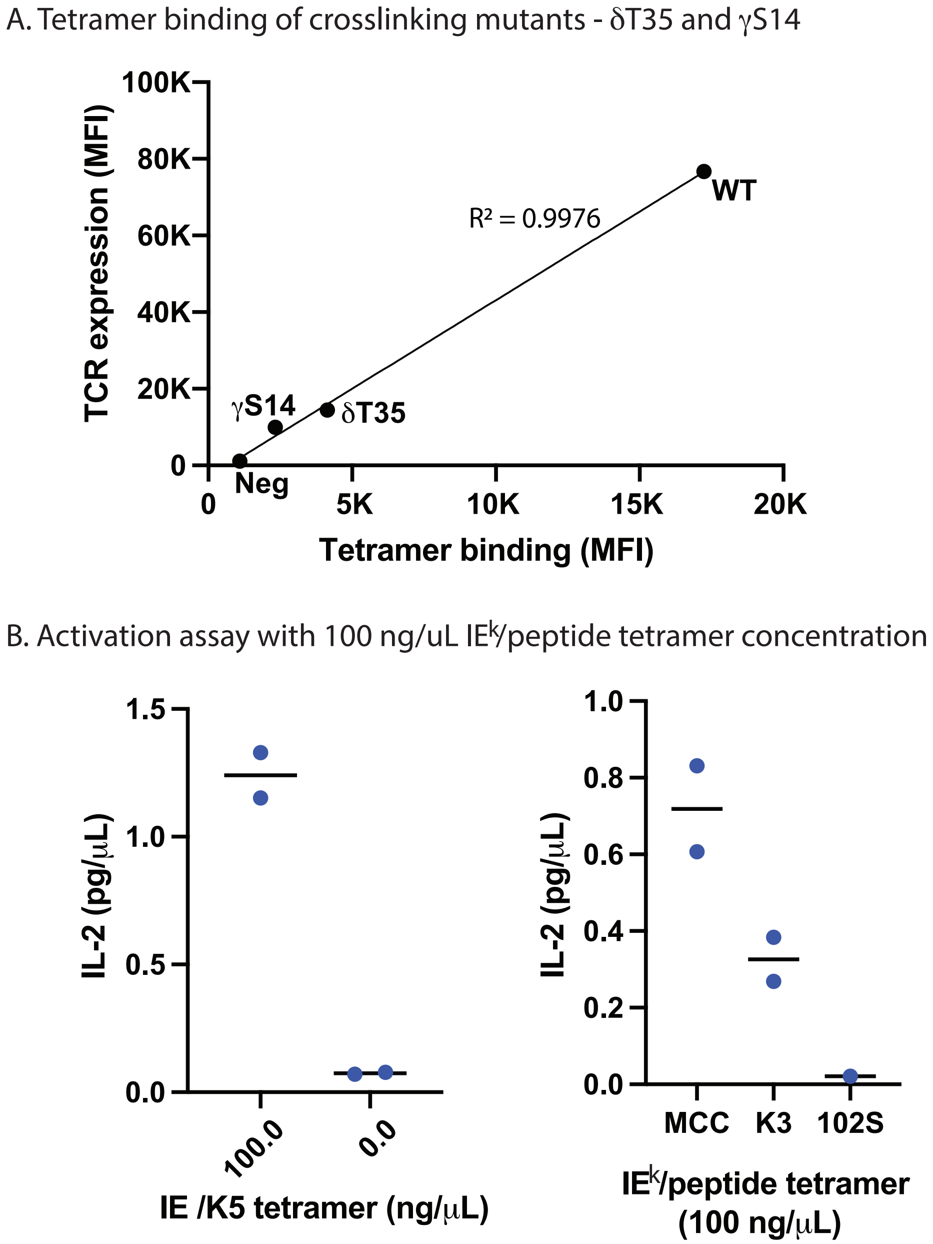

Appendix Figure S15. Related to Figure 6. IE^k tetramer binding and activation assays: A) Correlation plots between surface expression of TCRβ (MFI, stained with APC-conjugated H57-597 antibody) and IE^K/MCC tetramer staining (MFI, IE^k/MCC-APC tetramer) for the 2 mutants tested along with the wild type for 1 million cells at 100 ng/mL tetramer concentration (condition used for tetramer activation in crosslinking experiments). B) Area under the curve for IL-2 production with 100,000 WT 2B4 hybridoma cells activated with indicated pMHC IE^k tetramer at 100 ng/μL concentration (biological replicates, n = 2).

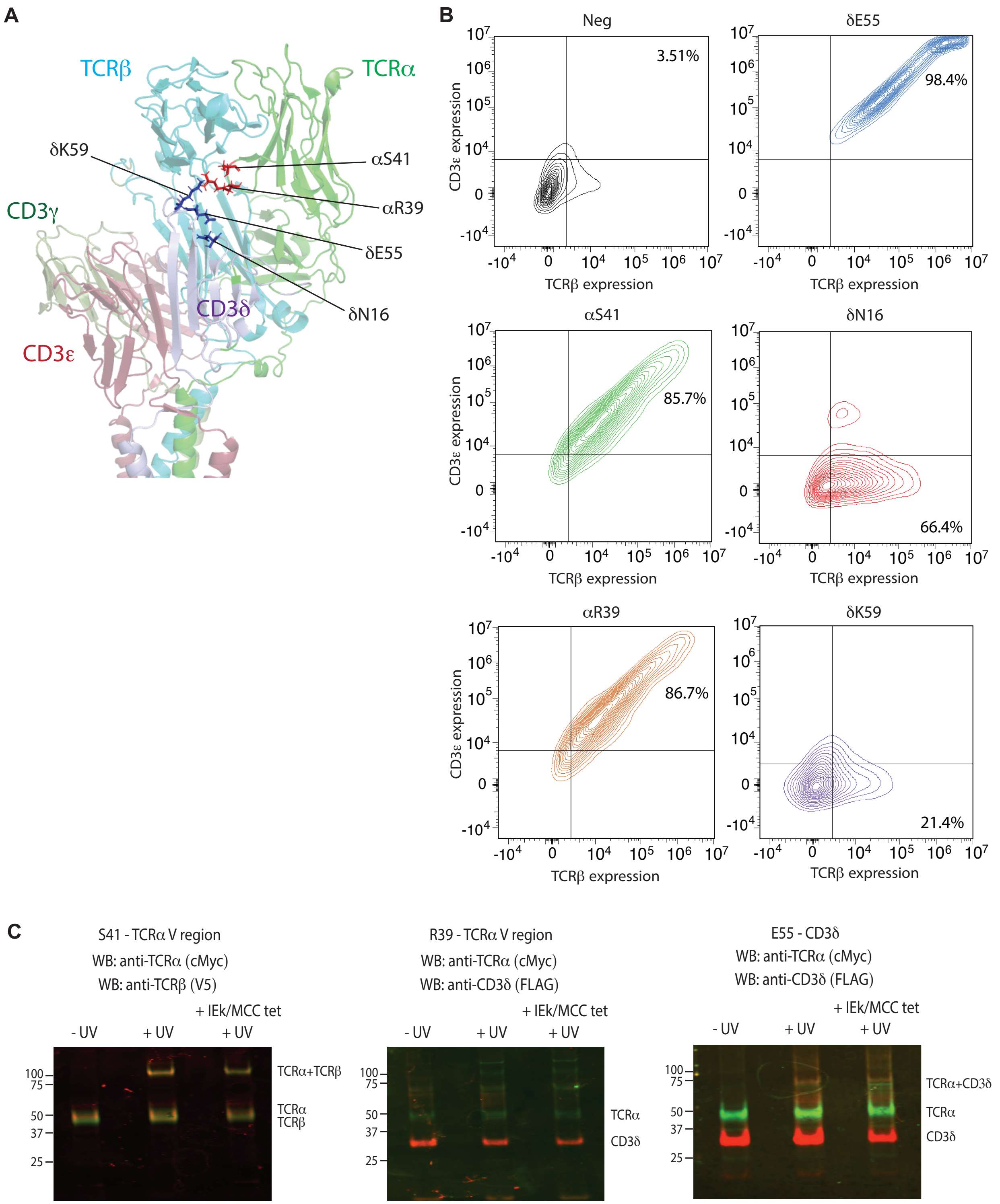

Appendix Figure S16. Related to Figure 6. Crosslinking reveals lack of conformation change in the TCRα-CD3δ interface upon antigen binding. A) Location of mutants – αR39, αS41, δN16, δE55 and δK59 in the crosslink-guided TCR-CD3 model. B) TCRβ and CD3ε expression plots of TCR and CD3 mutants – Negative, δE55, αS41, δN16, αR39 and δK59 by

flow cytometry. The percentage of cells positive for TCRβ and for both TCRβ and CD3ε staining is indicated. C) Western blot analysis of TCR and CD3 mutants - αS41, αR39, δE55 without UV irradiation, with UV irradiation and with IE^{k}/MCC tetramer and UV irradiation. TCRβ+TCRα crosslinked bands for are present above 75 kDa in the UV irradiated lanes for αS41. Blot was stained with anti-TCRβ (V5) antibody and anti-TCRα (cMyc). TCRα+CD3δ crosslinked bands for are present below 75 kDa in the UV irradiated lanes for δE55. Blot was stained with anti-TCRα (cMyc) antibody and anti-CD3δ (FLAG). Anti-mouse IRDye 680RD- and anti-rabbit IRDye 800CW were used as secondary antibodies for detection.

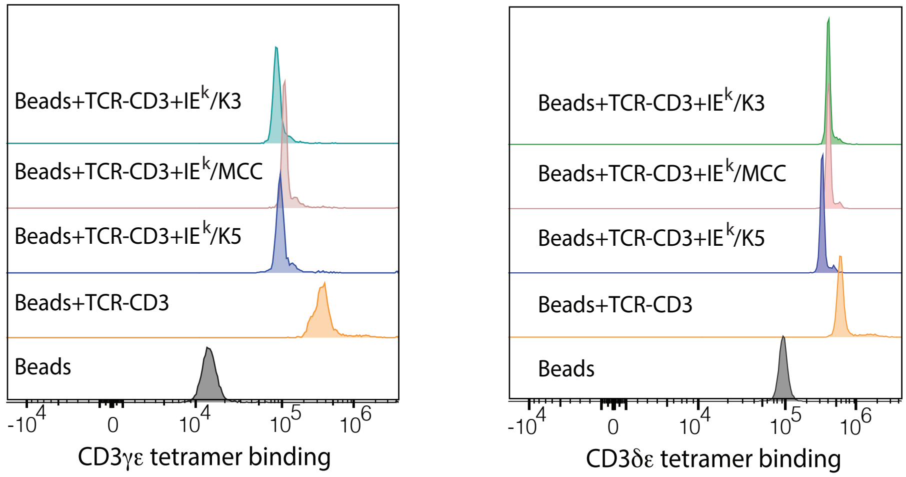

Appendix Figure S17. Related to Figure 7: CD3 tetramer assays reveal no major TCR-CD3 subunits reorganization upon activation. Left, Histogram of beads coated with the complex and bound with IE^{k}/K3 tetramer (green), IE^{k}/MCC tetramer (brown), IE^{k}/K5 tetramer (blue) and control beads (black), each stained with 50 ng/μL CD3γε tetramer. Right, Histogram of beads coated with the complex and bound with IE^{k}/K3 tetramer (green), IE^{k}/MCC tetramer (brown), IE^{k}/K5 tetramer (blue) and control beads (black), each stained with 50 ng/μL CD3δε tetramer.

Region	Sequence	Interacts with	Methodology	Reference
Cβ CC' loop	^{164}NGKEVHSG^{171}	CD3γε	Mutagenesis, EM structure	(Dong et al., 2019; Kuhns and Davis, 2007)

Cα DE loop	^{170}MKAMDS^{175}	CD3δε	Mutagenesis, EM structure	(Dong et al., 2019; Kuhns and Davis, 2007)
Cα AB loop	^{132}DPRSQDS^{138}	Conformational change upon antigen ligation	NMR, fluorescence and mutagenesis	(Beddoe et al., 2009; Rangarajan et al., 2018)
Cβ FG loop	^{219}LSEEDKWPEGSPK^{231}	CD3γε	NMR, antibody binding and docking	(Kim et al., 2009; Natarajan et al., 2016)
Cβ Helix 3	^{136}EIANKQK^{142}	CD3γε	NMR, mutagenesis and docking	(He et al., 2015; Kim et al., 2010; Natarajan et al., 2016; Natarajan et al., 2017)
Cβ Helix 4-F strand	^{204}HNPRNHFRC^{212}	CD3γε	NMR, EM structure, mutagenesis and docking	(Dong et al., 2019; He et al., 2015; Kim et al.,

				2010; Natarajan et al., 2016)
Cβ G strand	^{232}PVTQNISAEAWGRADC^{247}	CD3ε	EM structure	(Dong et al., 2019)

Appendix Table S1. Related to Figure 2: The regions of the TCR from which specific residues were tested for crosslinking. The TCR region, sequence, speculated interacting CD3 subunits, methodology used and reference are tabled.

Region	Sequence	Interacts with	Methodology	Reference
CD3δ A strand	^{3}QVT^{5}	TCRα	EM structure	(Dong et al., 2019)
CD3δ AB loop	^{7}YEDK^{10}	TCRα	EM structure	(Dong et al., 2019)
CD3δ BC loop	^{16}NTS^{18}			
CD3δ CD loop	^{25}TVE^{27}			
CD3δ E strand	^{35}TLN^{37}	TCRα	EM structure	(Dong et al., 2019)
CD3δ EF loop	^{40}KGVLD^{44}	TCRα	EM structure	
CD3γ AB loop	^{3}GSRGDGSV^{5}	TCRβ	EM structure	(Dong et al., 2019)

CD3γ CD loop	^{36}DG^{37}			
CD3γ DE loop	^{45}ATKN^{48}			
CD3γ EF loop	^{55}NAKDP^{59}			
CD3γ FG loop	^{67}GAKET^{71}			

Appendix Table S2. Related to Figure 3: CD3 regions used for testing. The CD3 region, sequence, speculated interacting TCR subunit, methodology used and reference are tabled.

Subunit	Species *in vitro*	PDB	PDB Template-Species
TCR (α and β) V & C domains	*Mus musculus*	3QJF and 1TCR	*Mus musculus* and *Homo sapiens* chimera; *Mus musculus*
TCR (α and β) TMs	*Mus musculus*	6JXR	*Homo sapiens*
CD3γε	*Mus musculus*	1JBJ	*Mus musculus*
CD3δε	*Mus musculus*	1XIW and 3R08	*Homo sapiens* and *Mus musculus*

Appendix Table S3. Related to Figure 4: PDB structures used for the docking crosslink-guided structure.

Region	Mutation	Energy Native	Energy pAzpa	Difference
Cβ CC' loop	S170	-10	116	106
	G171	102	674	776
	V168	-59	152766	152707
	K166	-52.7	-43	-95.7
	N164	-75.18	532	456.82
Cα DE loop	A172	55.4	57.56	112.96
	D174	6.15	375	381.15
Cα AB loop	D132	-17	367321	367304
	R134	-35	489	454
	Q136	-9	12	3
	S138	31	602	633
Cβ FG loop	E221	-16	-13	-29
	W225	-53	-20	-73
	L219	-79	296751	296672
	D223	-48	1642	1594
	S229	-23	-17.59	-40.59
	K231	-48	38782	38734
Cβ Helix 3	K140	877	855540	856417
	K142	905	913	1818
	E136	856	161966	162822
	I137	858	62341	63199
	A138	895	449672	450567
	Q141	885	979	1864
Cβ Helix 4-F strand	F202	-78	-37	-115
	R207	2.3	26	28.3
	H204	158618	470798	629416
	P206	158821	517595	676416
	N208	-101	2745	2644
	F210	-155	3408	3253
Cβ G strand	S238	-14	642236	642222
	W242	-102	-57	-159
	N236	-74	-39	-113
	E240	-64	-41	-105
	R244	158282	1439529	1597811
CD3δ A strand	T5	1794	1884	3678
CD3δ AB loop	E8	-9.8	-16	-25.8
	D9	23.4	8	31.4
CD3δ BC loop	T17	-16.7	16342	16325.3

CD3δ CD loop	V26	922	976	1898
CD3δ E strand	T35	-22.29	221	198.71
CD3δ EF loop	K40	1786	617326	619112
CD3γ AB loop	S14	296	307	603
	R15	205	619	824
CD3γ CD loop	D36	-35	85	50
CD3γ DE loop	T46	-61	99521	99460
	K47	24.5	108725	108749.5
CD3γ EF loop	K57	28.2	-12.2	16
CD3γ FG loop	A68	119	196584	196703

Appendix Table S4. Related to Figure 4. Effect of pAzpa substitutions in the individual components of the TCR-CD3 complex calculated with ICM. The crosslinked residues are indicated in blue and values in red indicate energy changes > 100000 upon pAzpa substitution.

CL Subunit	CL Residue	Subunit binds to	Distance (Å)
TCR β	S238	CD3 ε	7
TCR β	W242	CD3 ε	3
TCR β	F202	CD3 δ	2
TCR β	S170	CD3 δ	3
TCR β	G171	CD3 δ	3
TCR β	R207	CD3 ε	6.5
TCR β	E221	CD3 γ	6
TCR β	W225	CD3 γ	7
TCR α	A172	CD3 δ	5
TCR α	D174	CD3 δ	7
CD3 γ	S14	TCR β	5
CD3 γ	R15	TCR β	8
CD3 δ	T5	TCR β	1
CD3 δ	T35	TCR α	2
CD3 δ	K40	TCR α	5

Appendix Table S5. Related to Figure 4: Crosslinking residues to subunit distances

Crosslink-guided model:

Chain	Residue #	WT res	Mut res	ΔE binding	ΔE Stability	ΔE Solubility
TCR α	169	D	K	-3.76	-2.39	-0.69
TCR β	227	E	F	-2.11	4.47	-3.19

TCR β	236	N	R	-2.59	-6.15	-0.44
TCR β	238	S	K	-2.08	-3.842	0.48

Cryo-EM structure:

Chain	Residue #	WT res	Mut res	ΔE binding	ΔE Stability	ΔE Solubility
TCR β	216	S	R	-1.10	1.89	0.80
TCR β	259	W	Y	-2.58	-1.39	0.47
TCR β	182	G	H	-1.42	2.32	0.23
TCR β	257	E	K	-3.44	-0.25	-1.76

Appendix Table S6. Related to Figure 5: List of mutations identified from the crosslink-guided model and cryo-EM structure and the thermodynamic values associated with each mutation.

Mutants based on crosslink-guided structure: CD69 expression:

	**αD169K**	**βN236R**	**βS238K**
10 μM K5	ns (0.0511)	** (0.0082)	** (0.0085)
1.1 μM K5	ns (0.9058)	ns (0.0940)	ns (0.1117)

Mutants based on crosslink-guided structure: ICOS expression:

	**αD169K**	**βN236R**	**βS238K**
10 μM K5	* (0.0344)	*** (0.0003)	ns (0.0601)
1.1 μM K5	** (0.0065)	*** (0.0002)	* (0.0240)

Mutants based on cryo-EM structure: CD69 expression:

	**βG182H**	**βS216R**	**βE257K**	**βW259Y**
10 μM K5	** (0.0051)	* (0.0169)	* (0.0401)	* (0.0344)
1.1 μM K5	ns (0.0517)	* (0.0172)	ns (0.0550)	ns (0.4282)

Appendix Table S7. Related to Figure 5 and Figure EV2: Unpaired student t-test p values comparing mutants with wild type. P > 0.05 indicate ns.
