# Supplementary figures and images for: In situ cell-surface conformation of the TCR-CD3 signaling complex (part 1 of 2)
Source: EMBO Rep. 2024 Nov 7;25(12):26. doi: 10.1038/s44319-024-00314-3 (PMC11624261; doi:10.1038/s44319-024-00314-3)

S41

K65

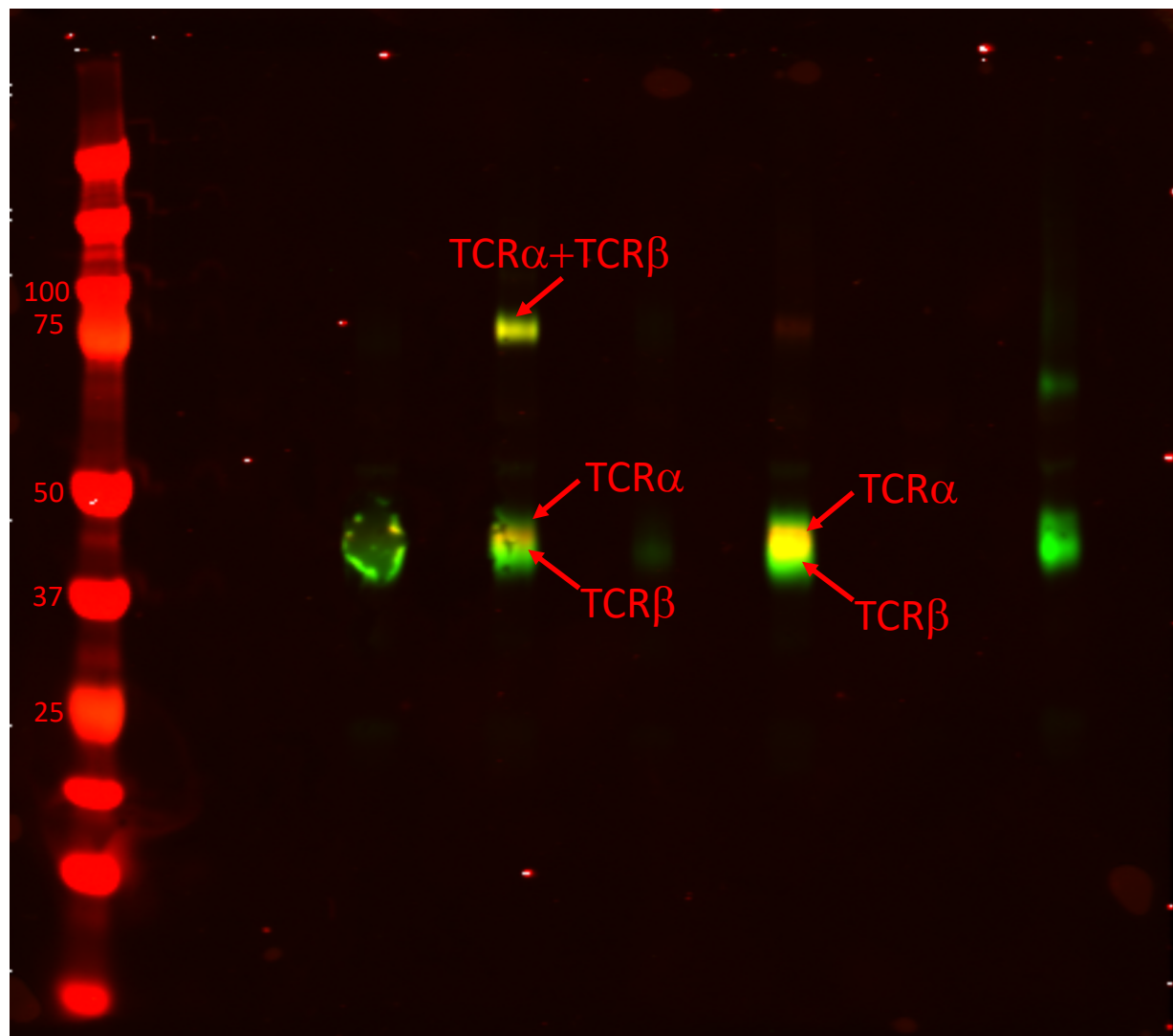

Supplement: Supplementary file 6 — Source data Fig. 1 [file 44319_2024_314_MOESM6_ESM.zip › Fig1_WB/Figure1G-full-labeled.pdf]

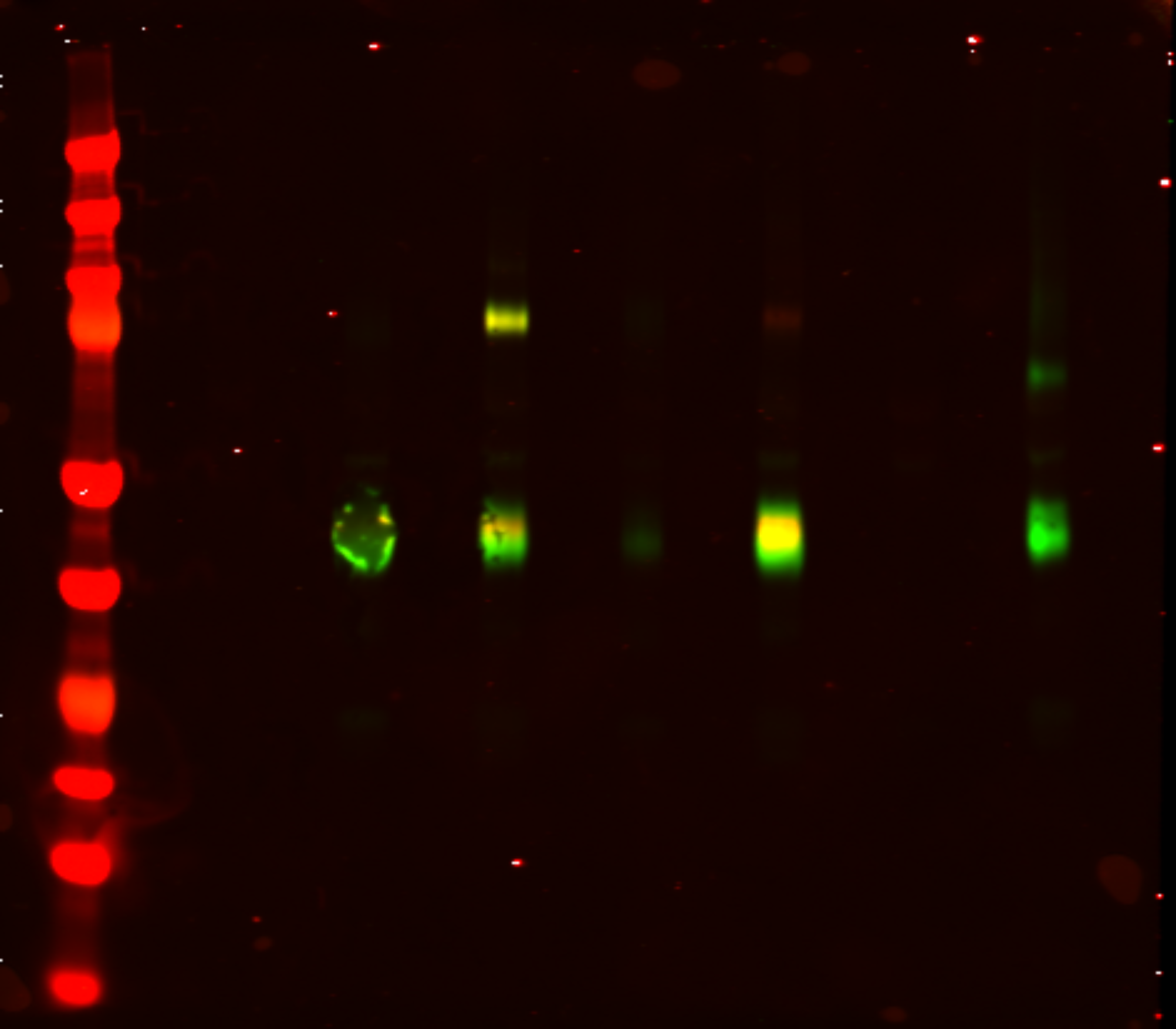

Supplement: Supplementary file 6 — Source data Fig. 1 [file 44319_2024_314_MOESM6_ESM.zip › Fig1_WB/Figure1G-full.png]

## Slide 1
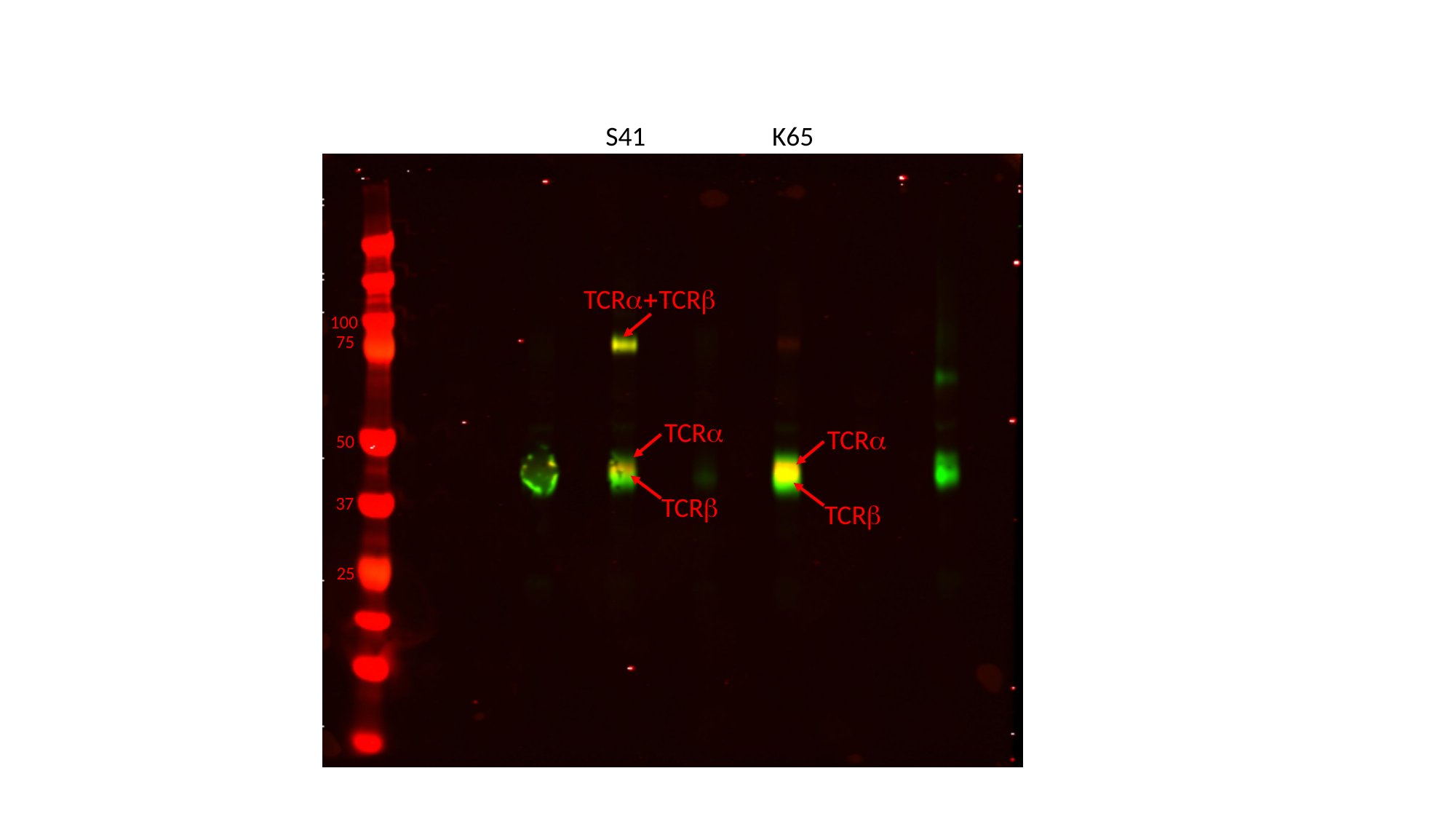

S41
K65
TCRa+TCRb
100
75
TCRa
TCRa
50
TCRb
37
TCRb
25

Supplement: Supplementary file 6 — Source data Fig. 1 [file 44319_2024_314_MOESM6_ESM.zip › Fig1_WB/Figure1G-full.pptx]

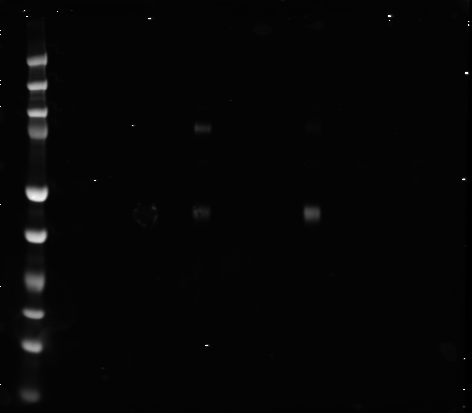

Supplement: Supplementary file 6 — Source data Fig. 1 [file 44319_2024_314_MOESM6_ESM.zip › Fig1_WB/Figure 1G-rawdata/700.TIF]

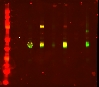

Supplement: Supplementary file 6 — Source data Fig. 1 [file 44319_2024_314_MOESM6_ESM.zip › Fig1_WB/Figure 1G-rawdata/2019-12-19-182820_new-S41K65_PU6_V5cmyc_TH.jpg]

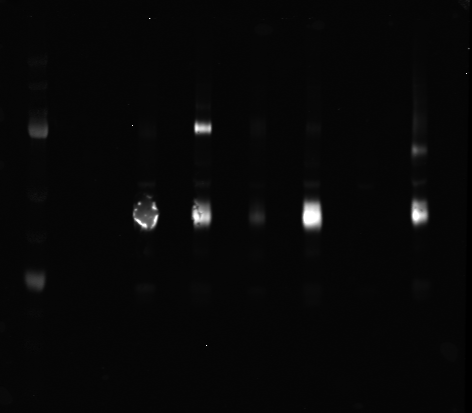

Supplement: Supplementary file 6 — Source data Fig. 1 [file 44319_2024_314_MOESM6_ESM.zip › Fig1_WB/Figure 1G-rawdata/800.TIF]

S138

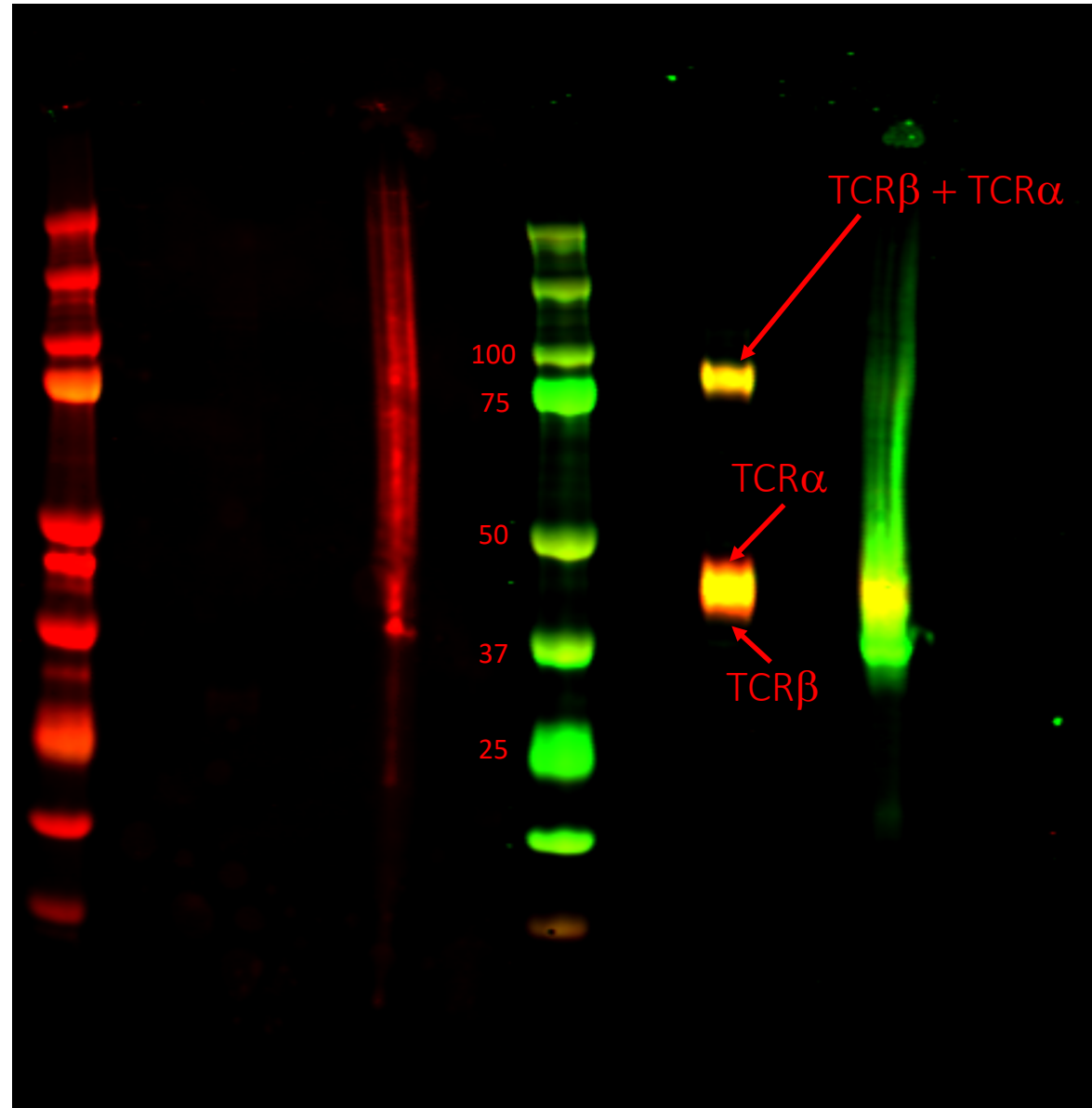

Supplement: Supplementary file 7 — Source data Fig. 2 [file 44319_2024_314_MOESM7_ESM.zip › Fig2_WB/Figure 2D-ABloop/S138-mCmyc-rV5-full-labeled.pdf]

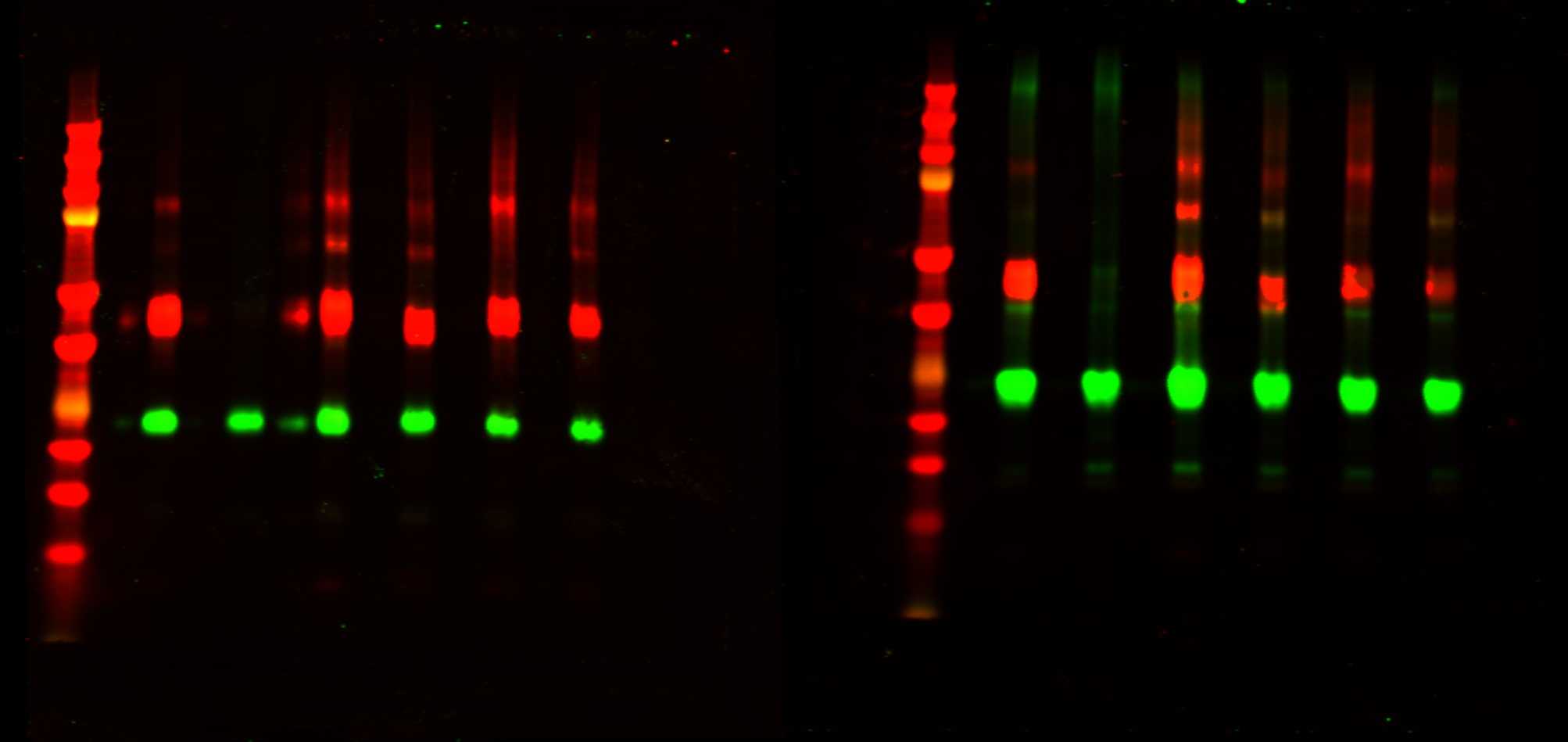

Supplement: Supplementary file 7 — Source data Fig. 2 [file 44319_2024_314_MOESM7_ESM.zip › Fig2_WB/Figure 2D-ABloop/R134S138-V5-VSVG-HA-full.png]

## Slide 1
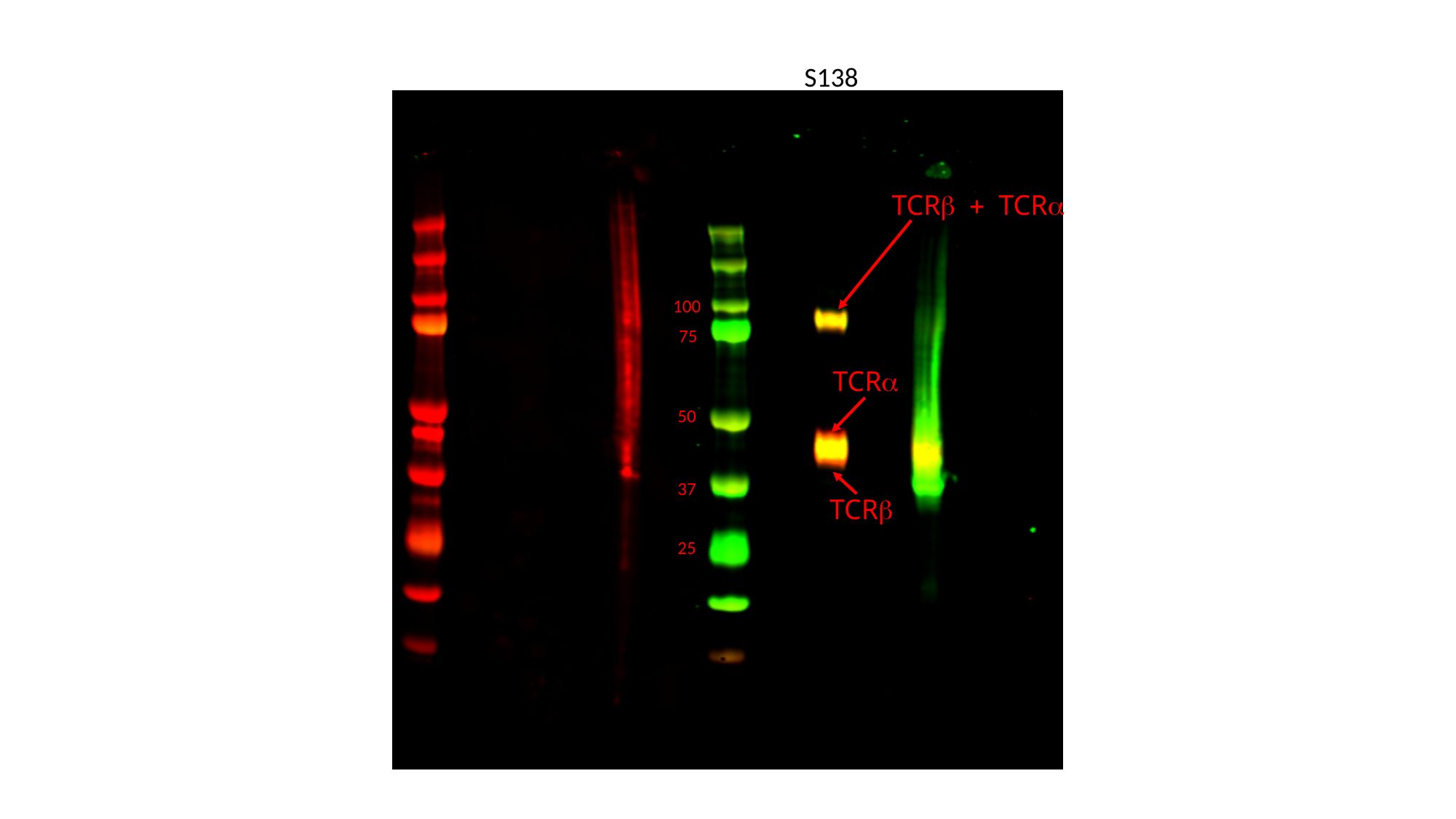

S138
TCRb + TCRa
100
75
TCRa
50
37
TCRb
25

Supplement: Supplementary file 7 — Source data Fig. 2 [file 44319_2024_314_MOESM7_ESM.zip › Fig2_WB/Figure 2D-ABloop/S138-mCmyc-rV5-full-labeled.pptx]

S138 R134

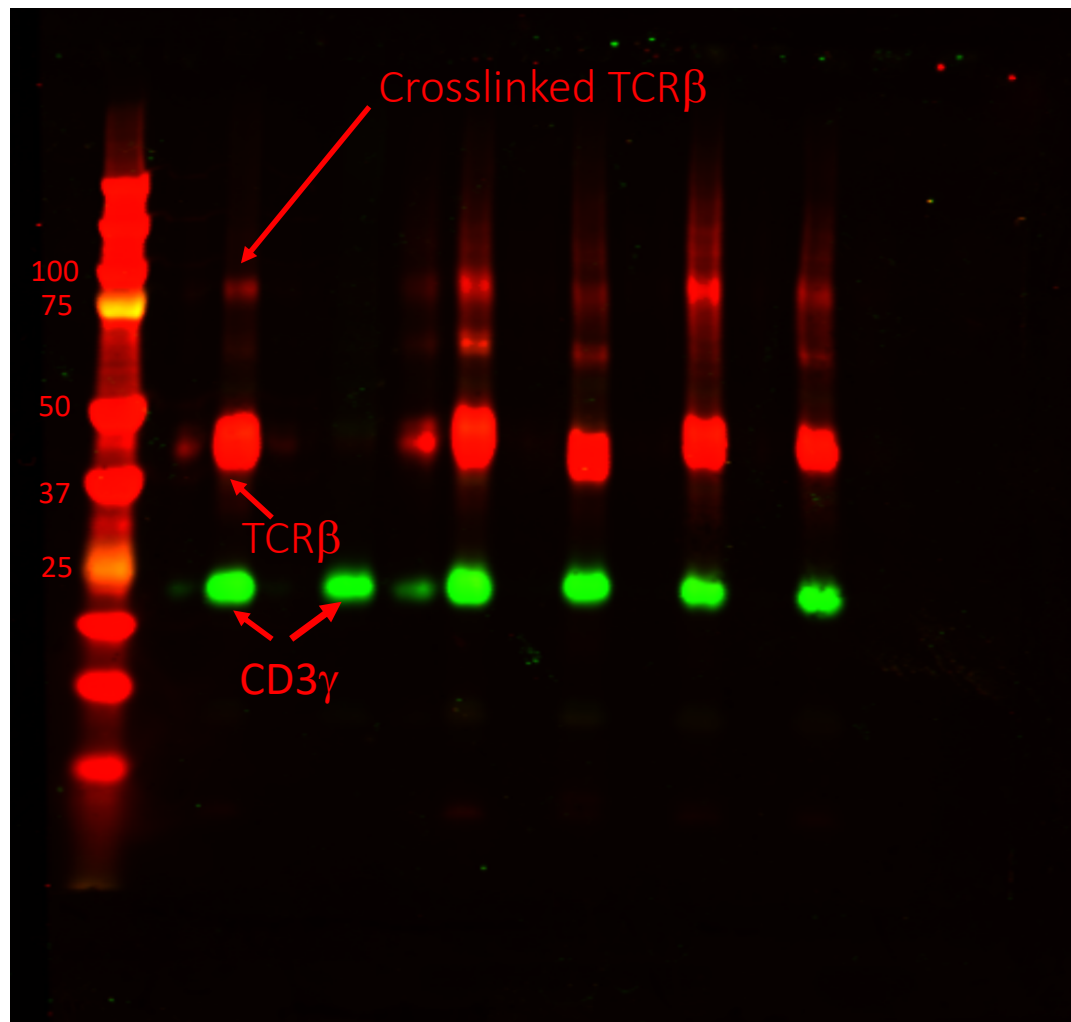

S138 R134

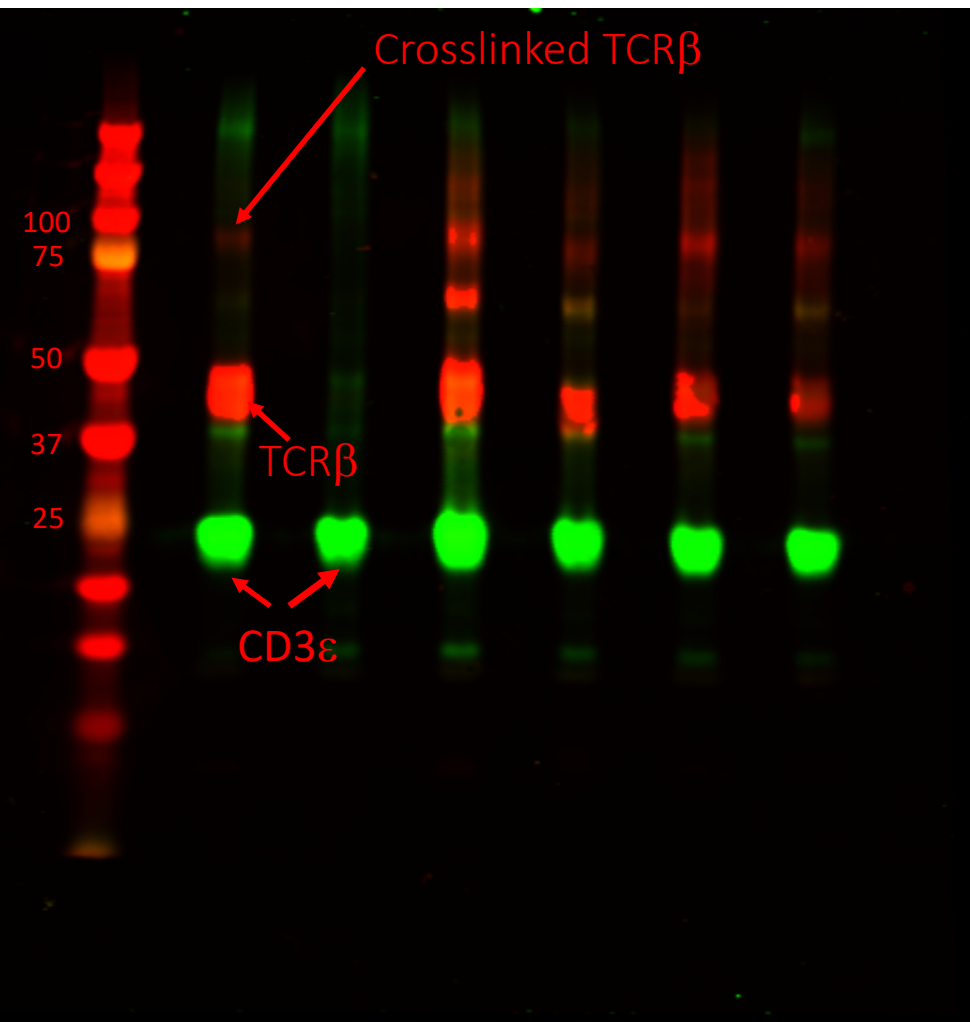

Supplement: Supplementary file 7 — Source data Fig. 2 [file 44319_2024_314_MOESM7_ESM.zip › Fig2_WB/Figure 2D-ABloop/R134S138-V5-VSVG-HA-full-labeled.pdf]

## Slide 1
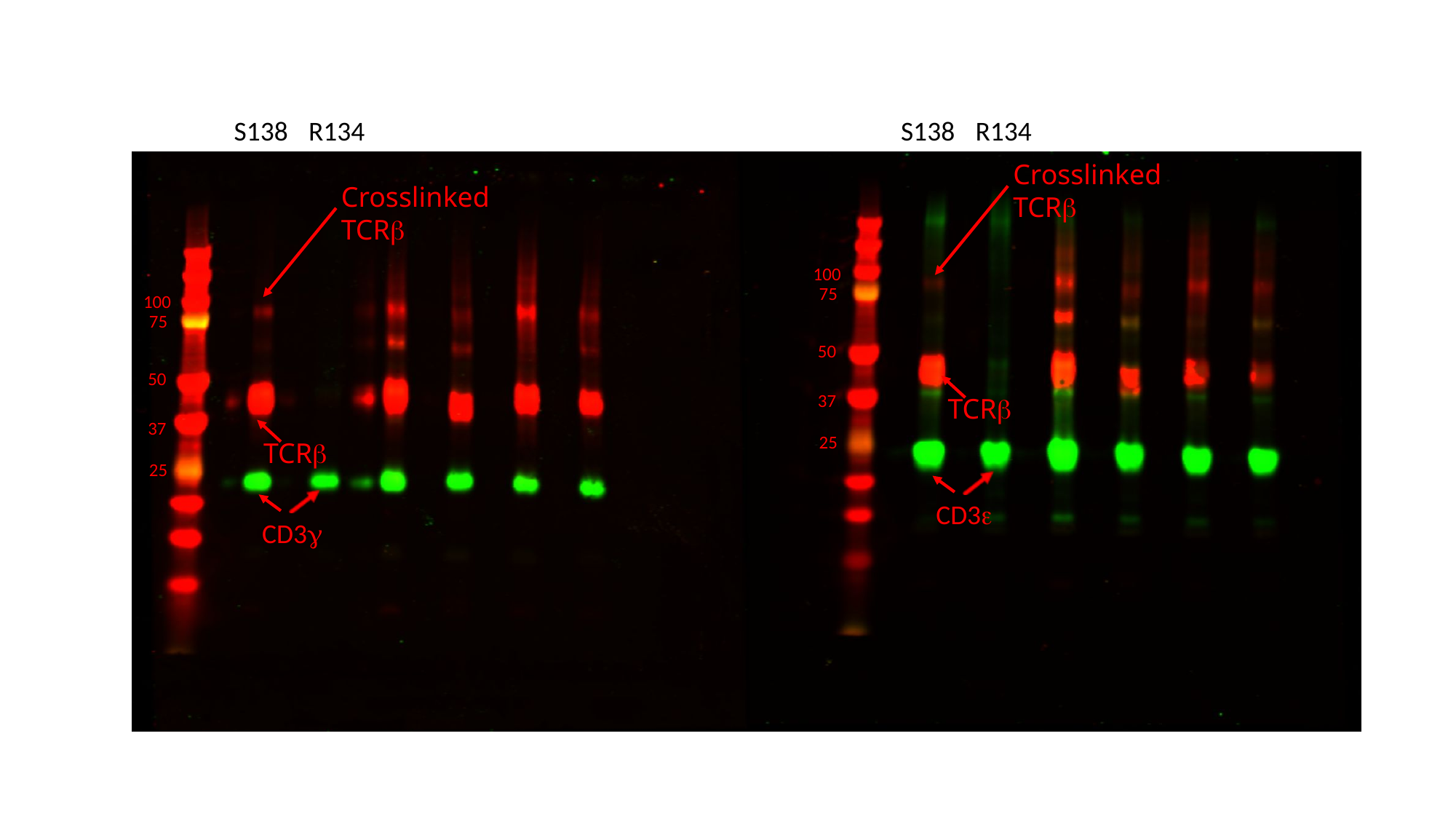

S138
R134
S138
R134
Crosslinked TCRb
Crosslinked TCRb
100
75
100
75
50
50
37
TCRb
37
25
TCRb
25
CD3e
CD3g

Supplement: Supplementary file 7 — Source data Fig. 2 [file 44319_2024_314_MOESM7_ESM.zip › Fig2_WB/Figure 2D-ABloop/R134S138-V5-VSVG-HA-full-labeled.pptx]

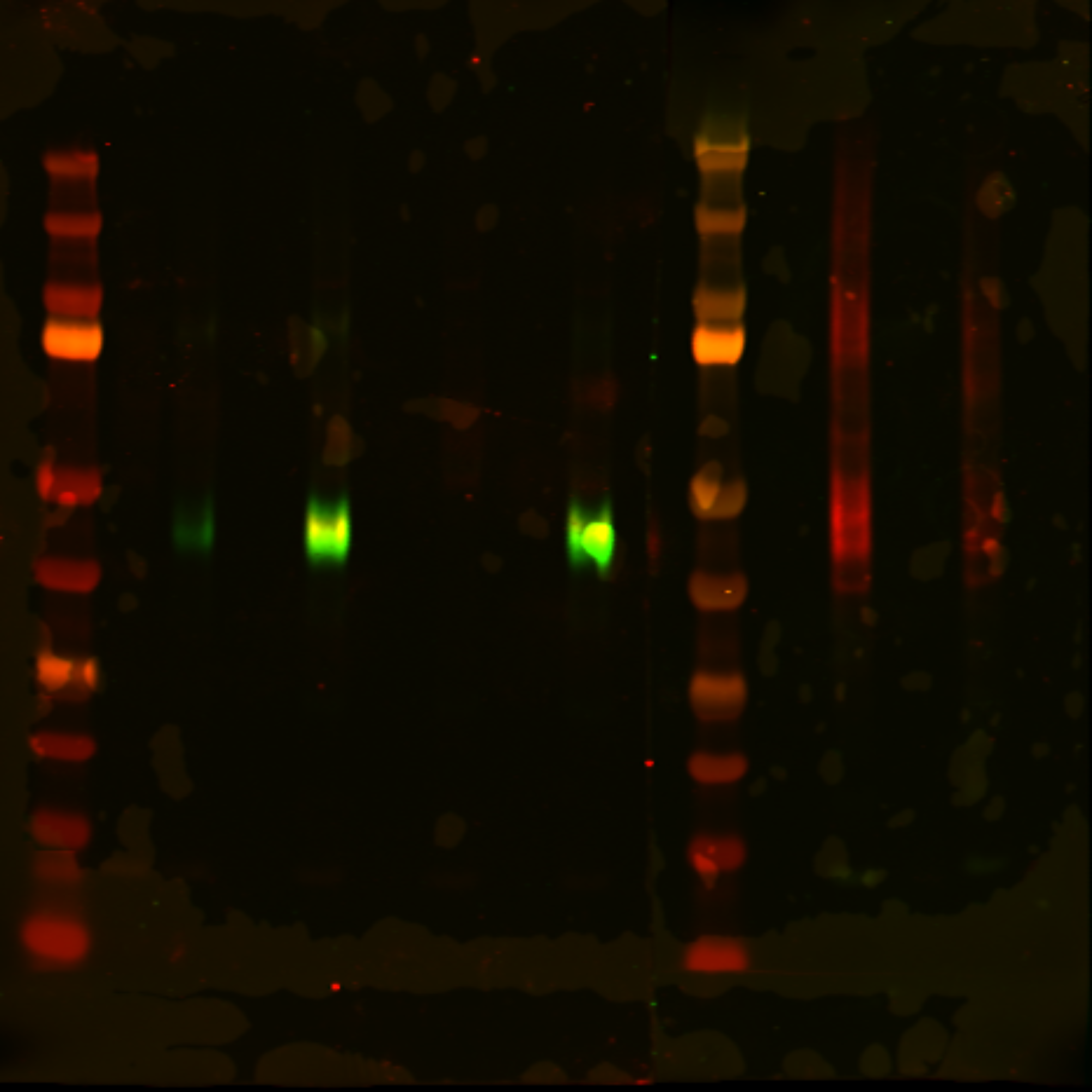

Supplement: Supplementary file 7 — Source data Fig. 2 [file 44319_2024_314_MOESM7_ESM.zip › Fig2_WB/Figure 2D-ABloop/Figure2-figure supplement 3- full.png.png]

## Slide 1
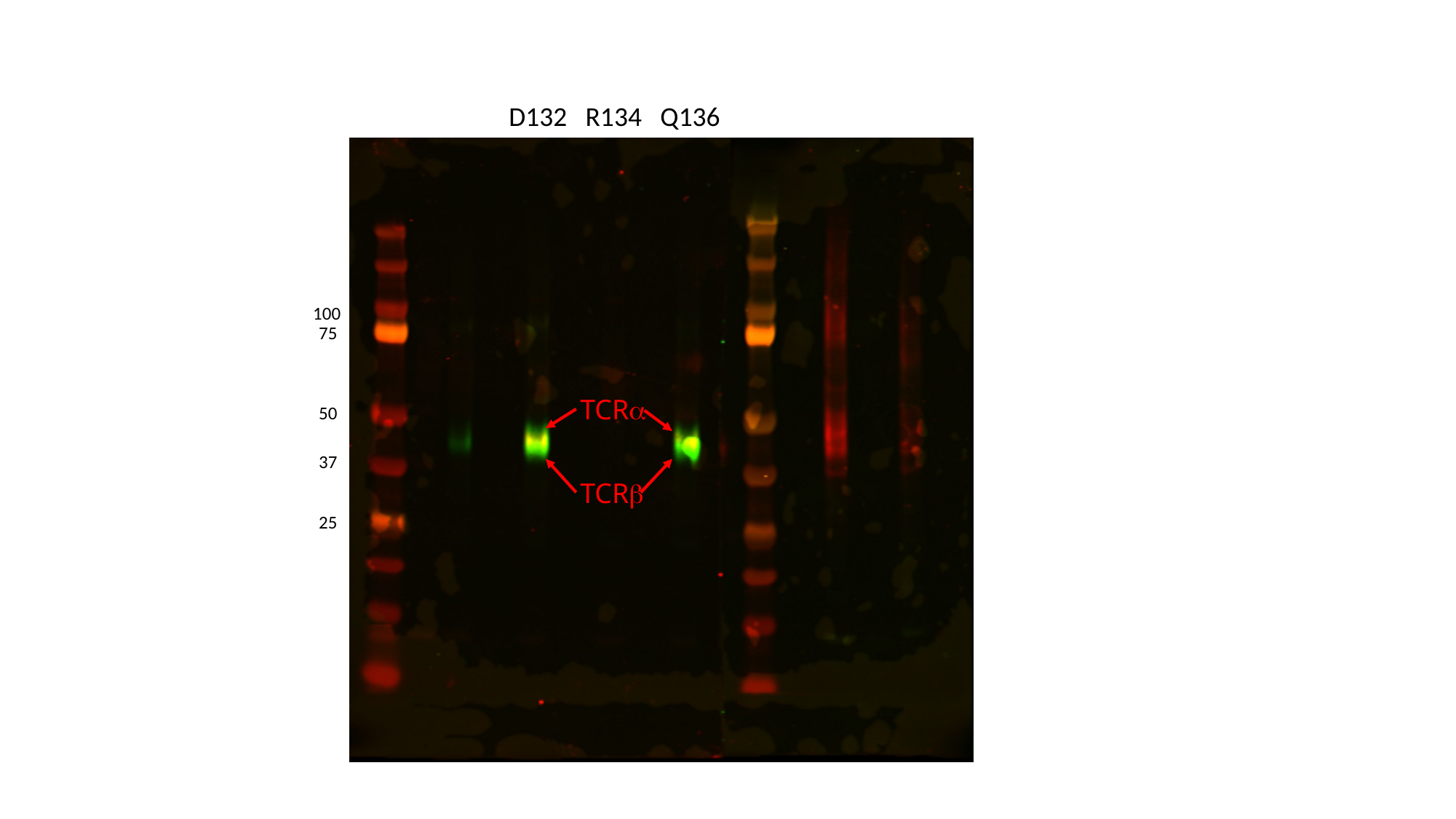

D132 R134 Q136
100
75
TCRa
50
37
TCRb
25

Supplement: Supplementary file 7 — Source data Fig. 2 [file 44319_2024_314_MOESM7_ESM.zip › Fig2_WB/Figure 2D-ABloop/Figure2-figure supplement 3- full.pptx]

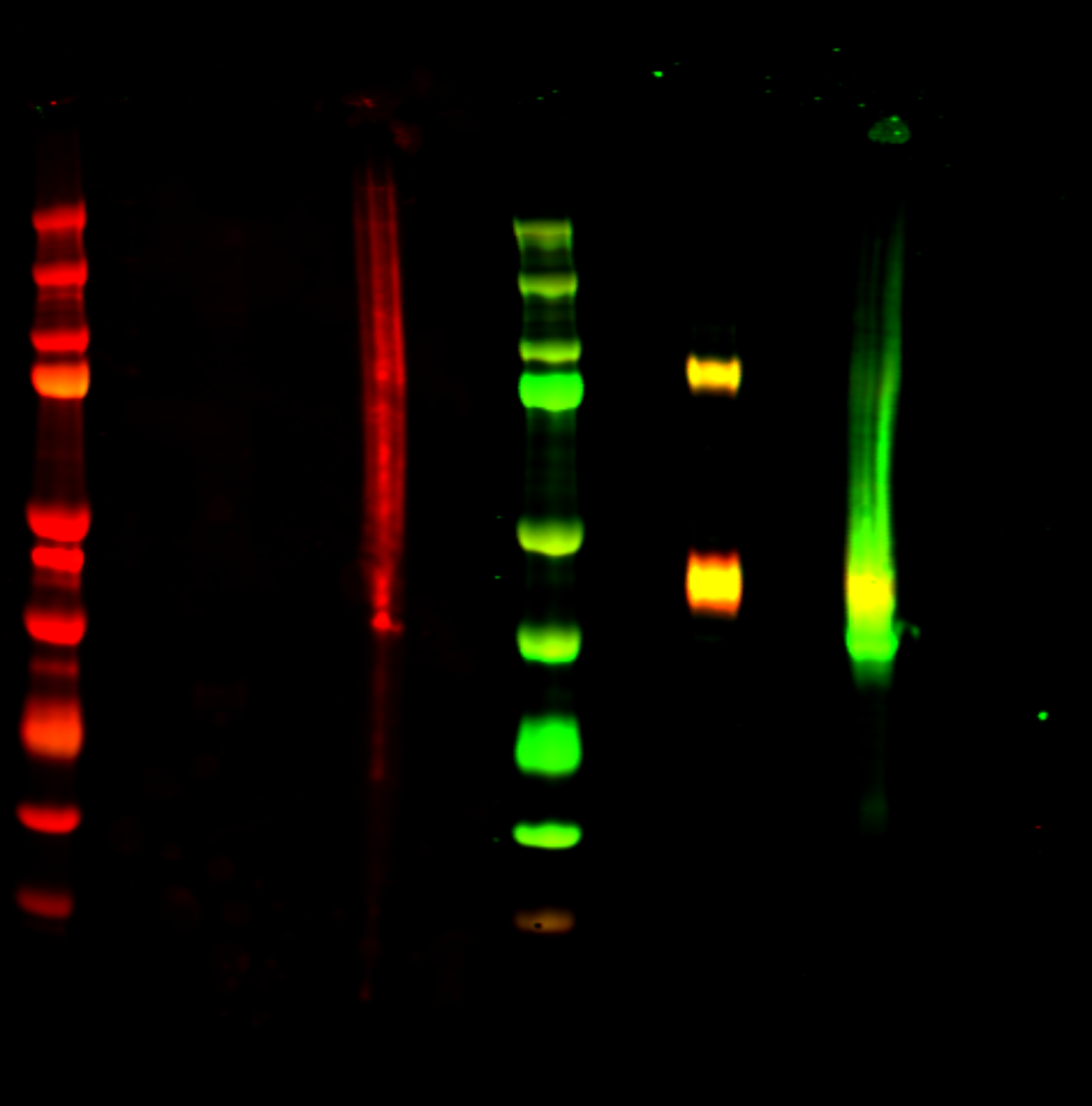

Supplement: Supplementary file 7 — Source data Fig. 2 [file 44319_2024_314_MOESM7_ESM.zip › Fig2_WB/Figure 2D-ABloop/S138-mCmyc-rV5-full.png]

D132 R134 Q136

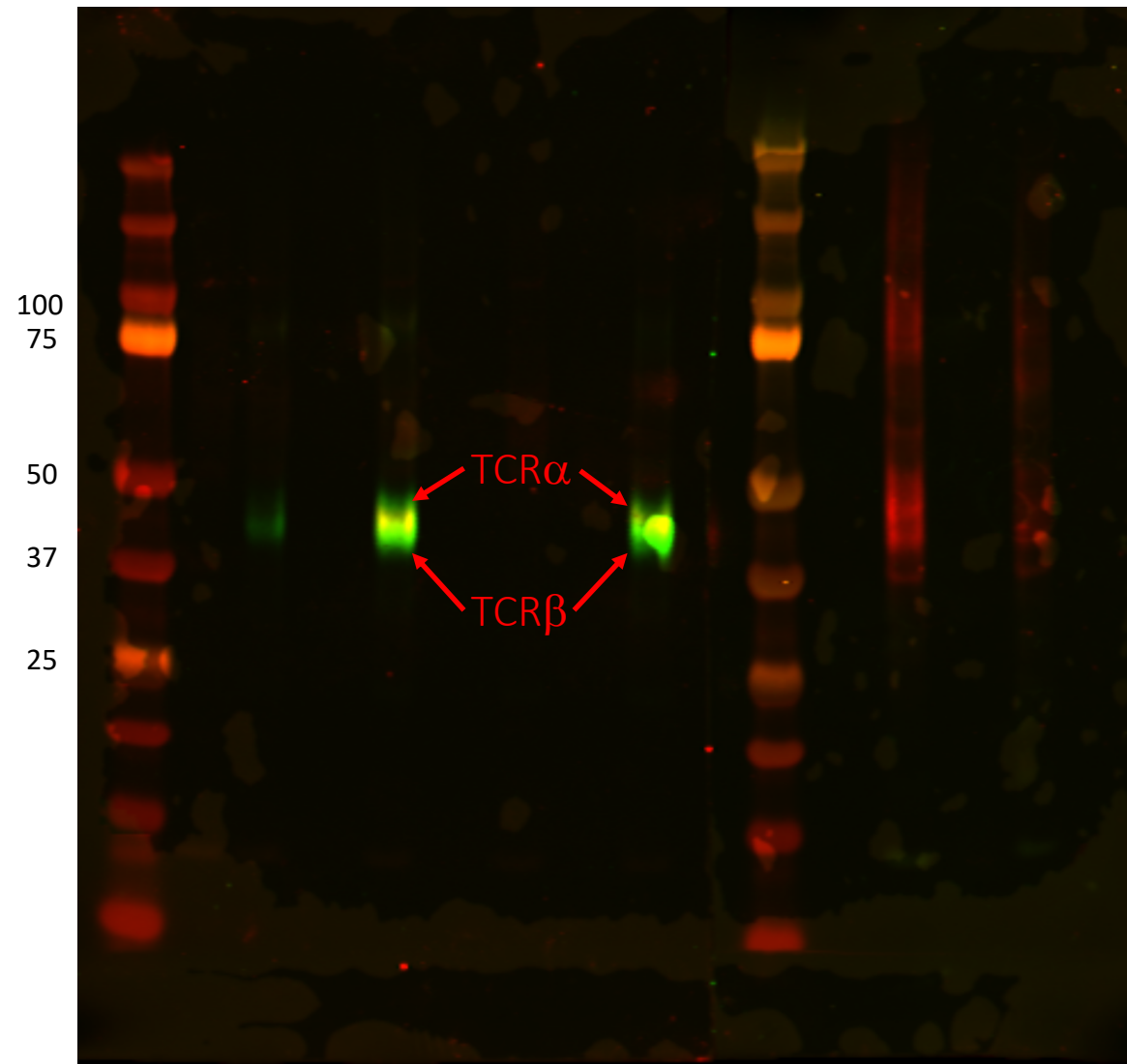

Supplement: Supplementary file 7 — Source data Fig. 2 [file 44319_2024_314_MOESM7_ESM.zip › Fig2_WB/Figure 2D-ABloop/Figure2-figure supplement 3- full-labeled.pdf]

## Slide 1
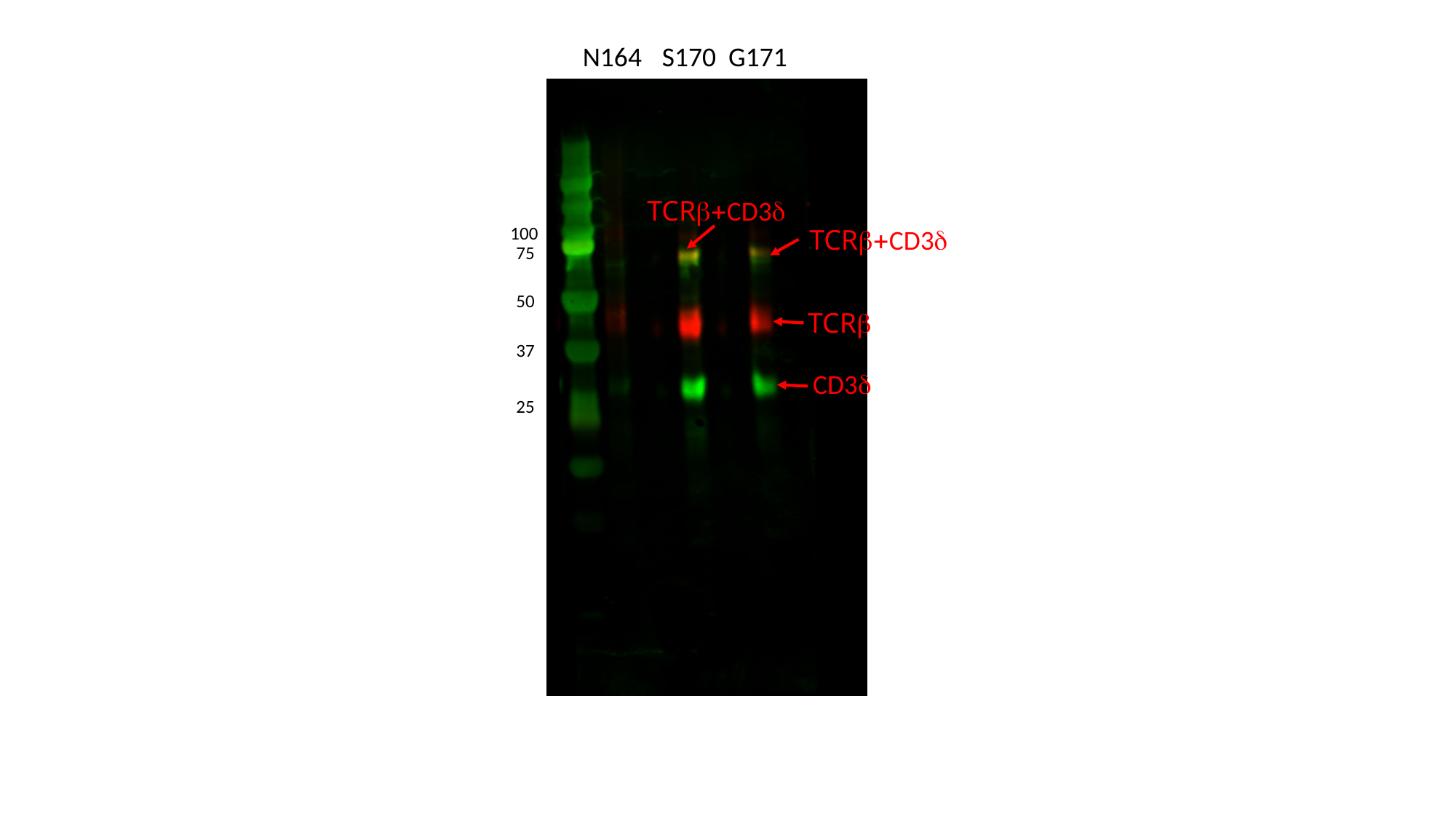

S170 G171
N164
TCRb+CD3d
100
TCRb+CD3d
75
50
TCRb
37
CD3d
25

Supplement: Supplementary file 7 — Source data Fig. 2 [file 44319_2024_314_MOESM7_ESM.zip › Fig2_WB/Figure 2C-CCloop/Figure2C-full-labeled.pptx]

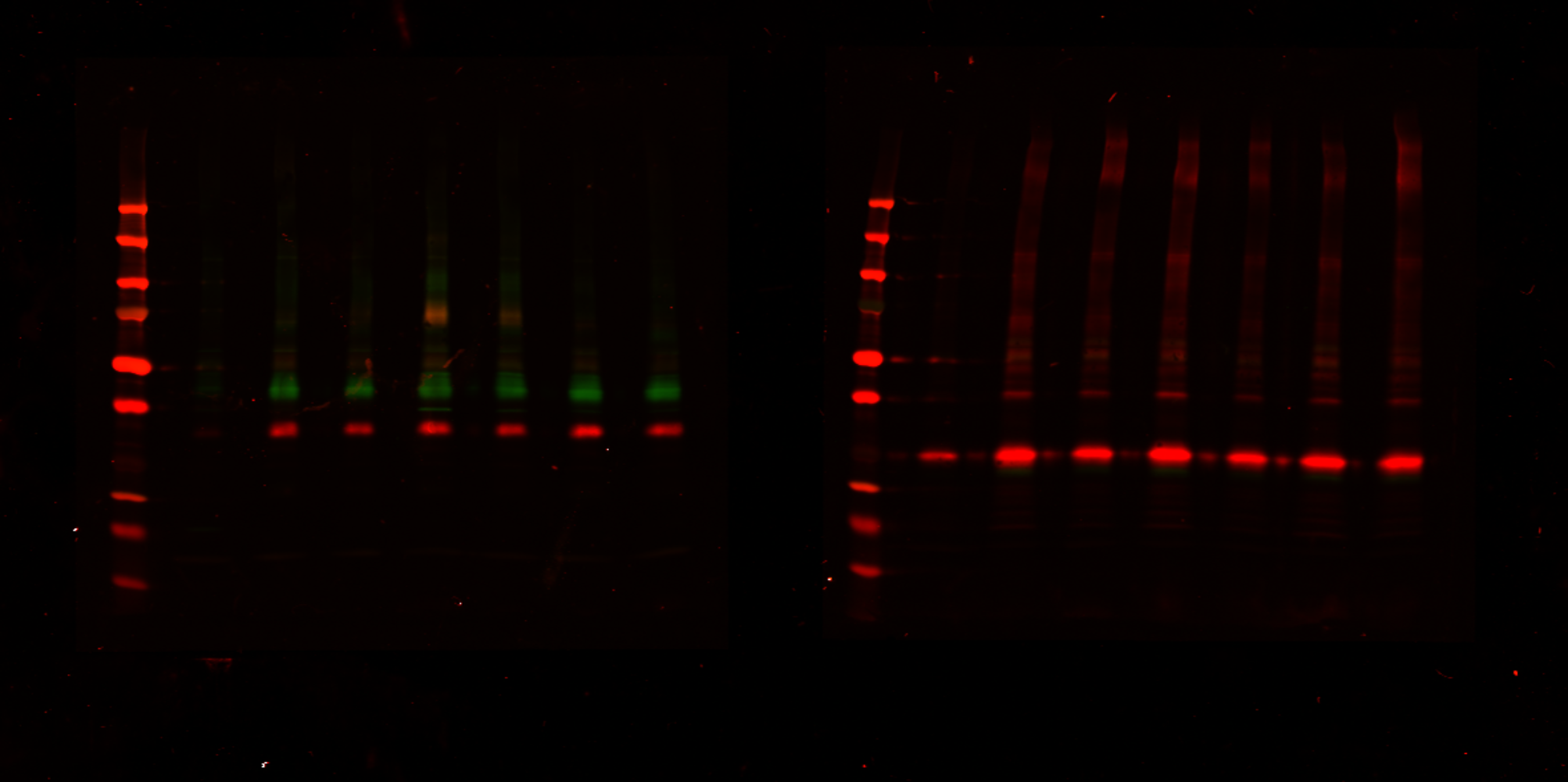

Supplement: Supplementary file 7 — Source data Fig. 2 [file 44319_2024_314_MOESM7_ESM.zip › Fig2_WB/Figure 2C-CCloop/Figure2-figure supplement 2- full.png]

N164 K166 V168 S170 G171

N164 K166 V168 S170 G171

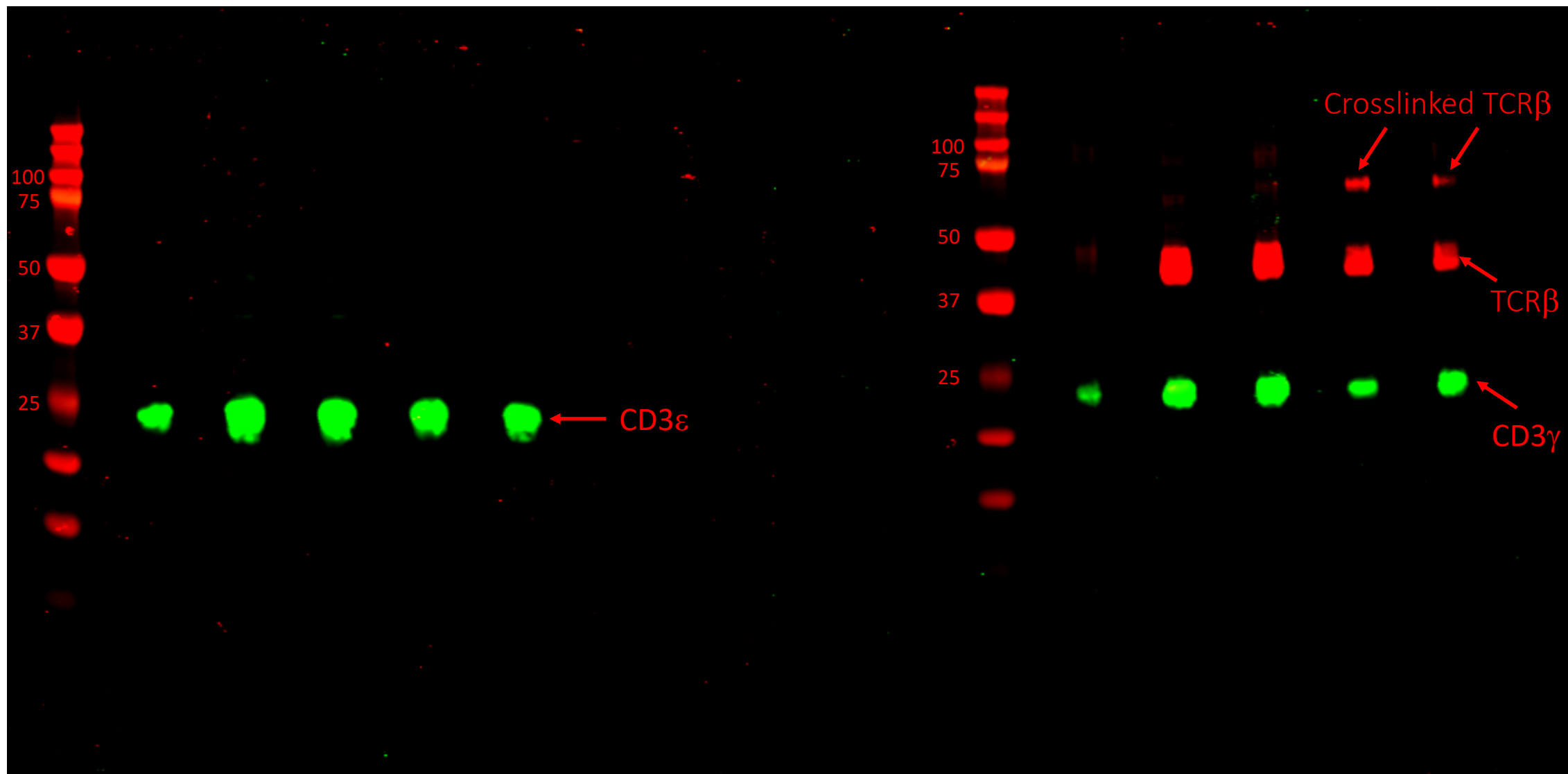

Supplement: Supplementary file 7 — Source data Fig. 2 [file 44319_2024_314_MOESM7_ESM.zip › Fig2_WB/Figure 2C-CCloop/Figure2-figure supplement 2-2-full-labeled.pdf]

## Slide 1
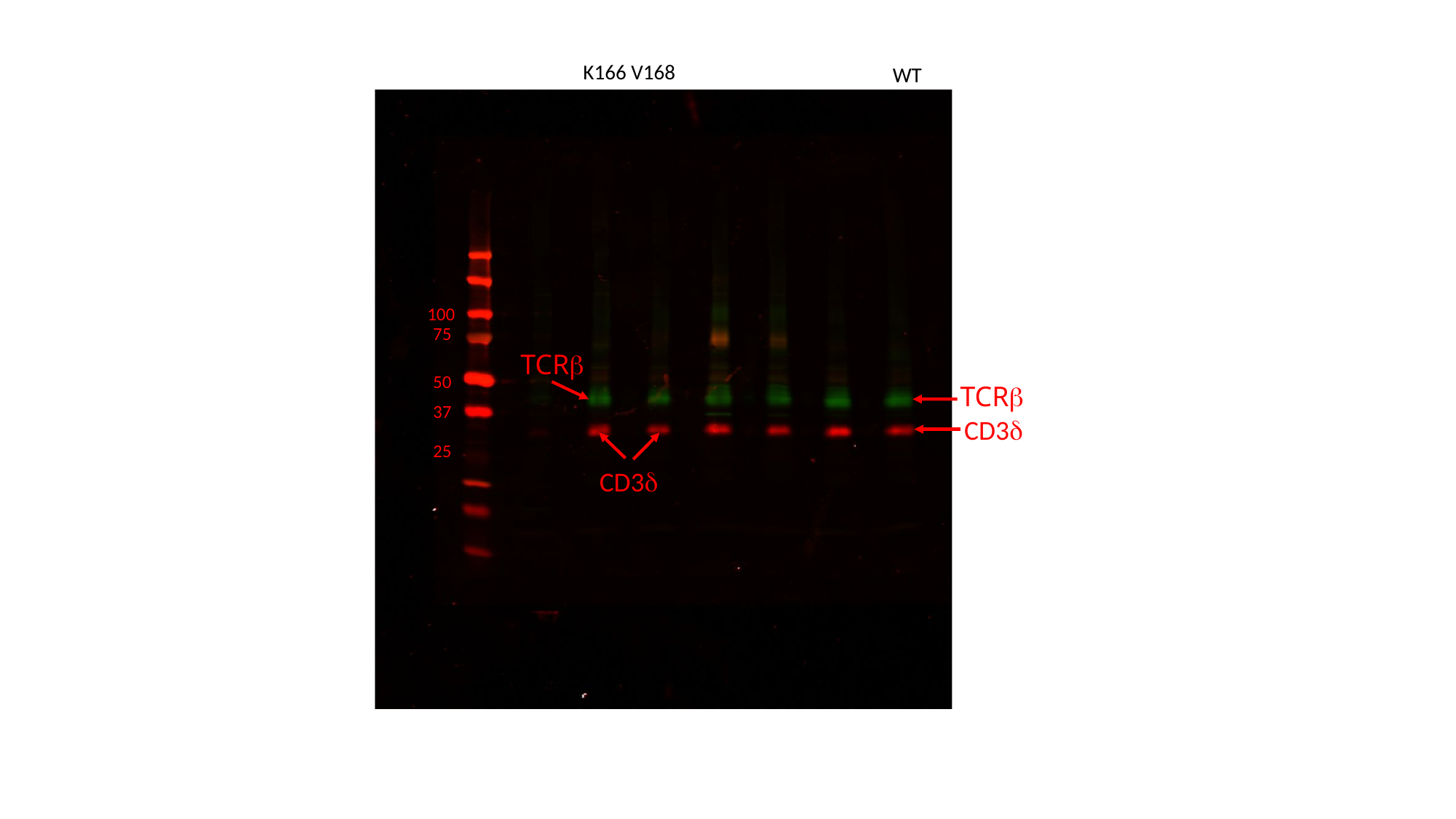

K166 V168
WT
100
75
TCRb
50
TCRb
37
CD3d
25
CD3d

Supplement: Supplementary file 7 — Source data Fig. 2 [file 44319_2024_314_MOESM7_ESM.zip › Fig2_WB/Figure 2C-CCloop/Figure2-figure supplement 2- full.pptx]

N164 S170 G171

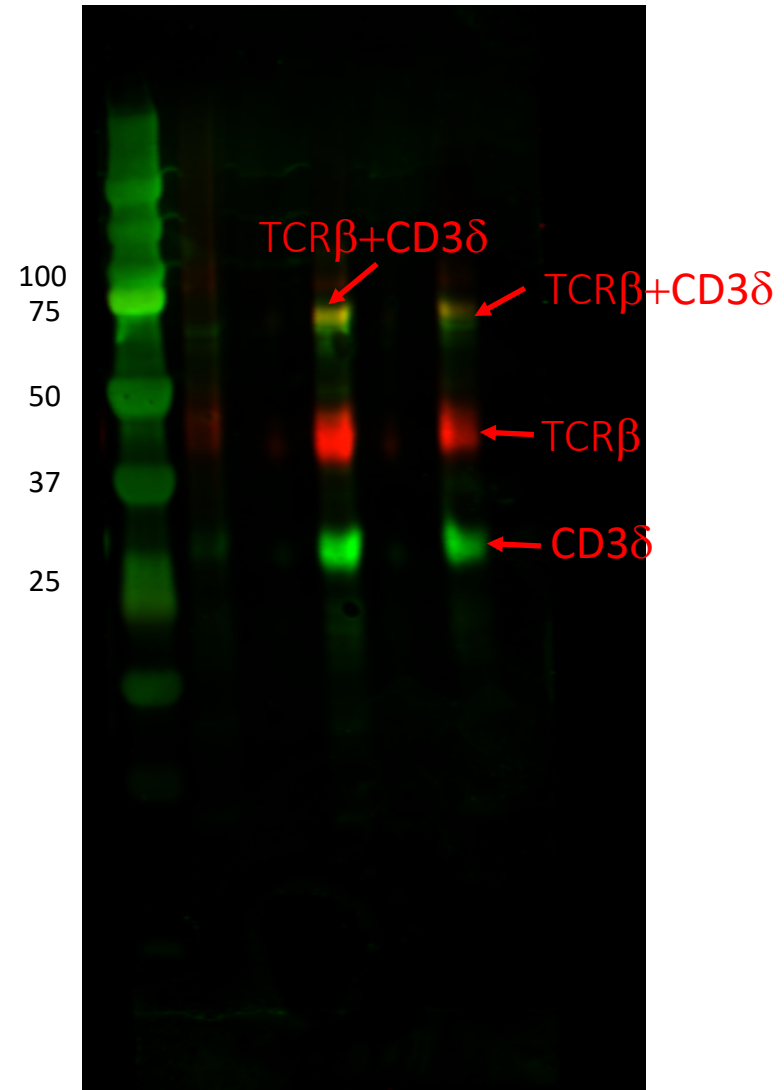

Supplement: Supplementary file 7 — Source data Fig. 2 [file 44319_2024_314_MOESM7_ESM.zip › Fig2_WB/Figure 2C-CCloop/Figure2C-full-labeled.pdf]

## Slide 1
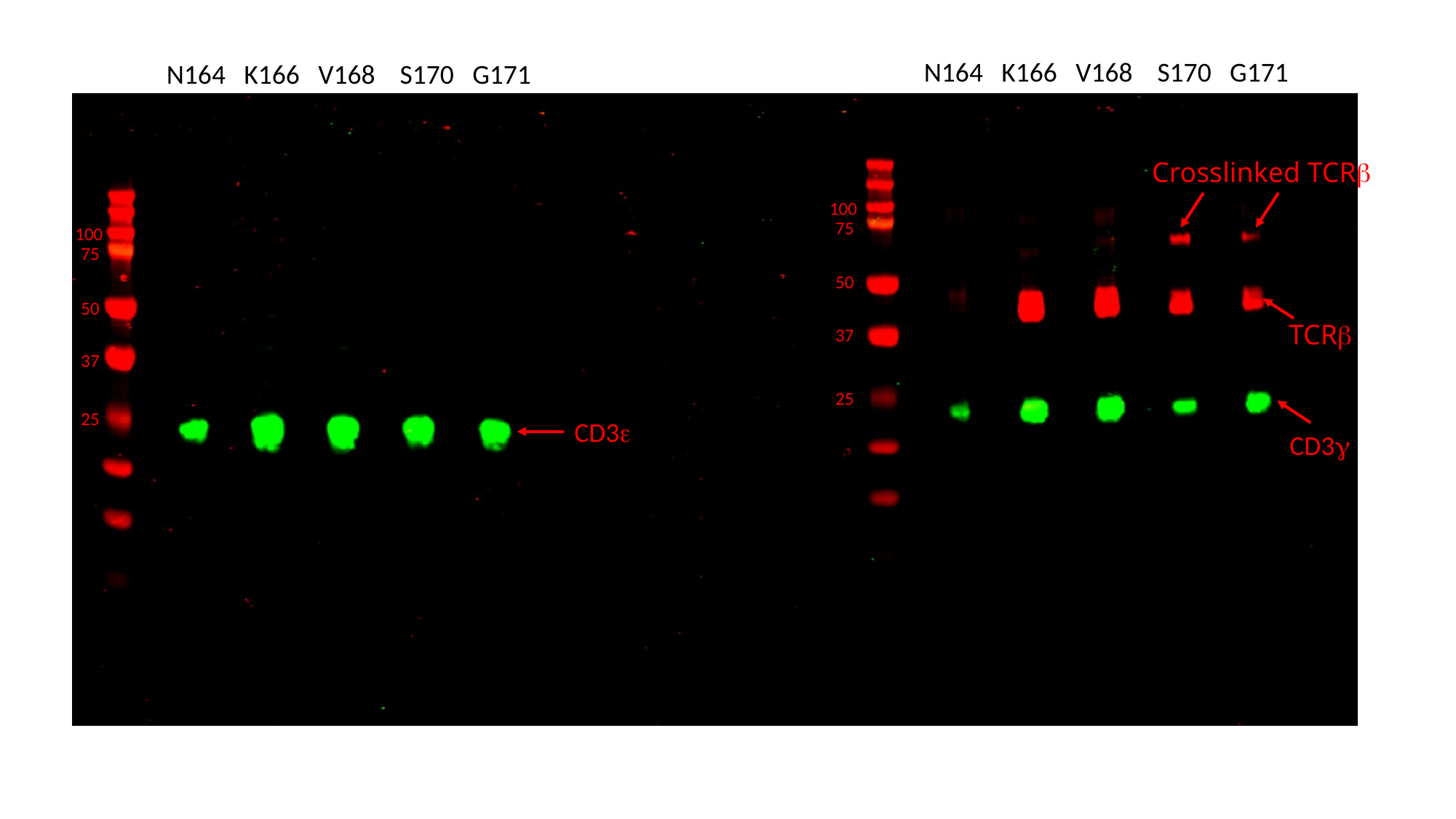

N164 K166 V168 S170 G171
N164 K166 V168 S170 G171
Crosslinked TCRb
100
75
100
75
50
50
TCRb
37
37
25
25
CD3e
CD3g

Supplement: Supplementary file 7 — Source data Fig. 2 [file 44319_2024_314_MOESM7_ESM.zip › Fig2_WB/Figure 2C-CCloop/Figure2-figure supplement 2-2-full.pptx]

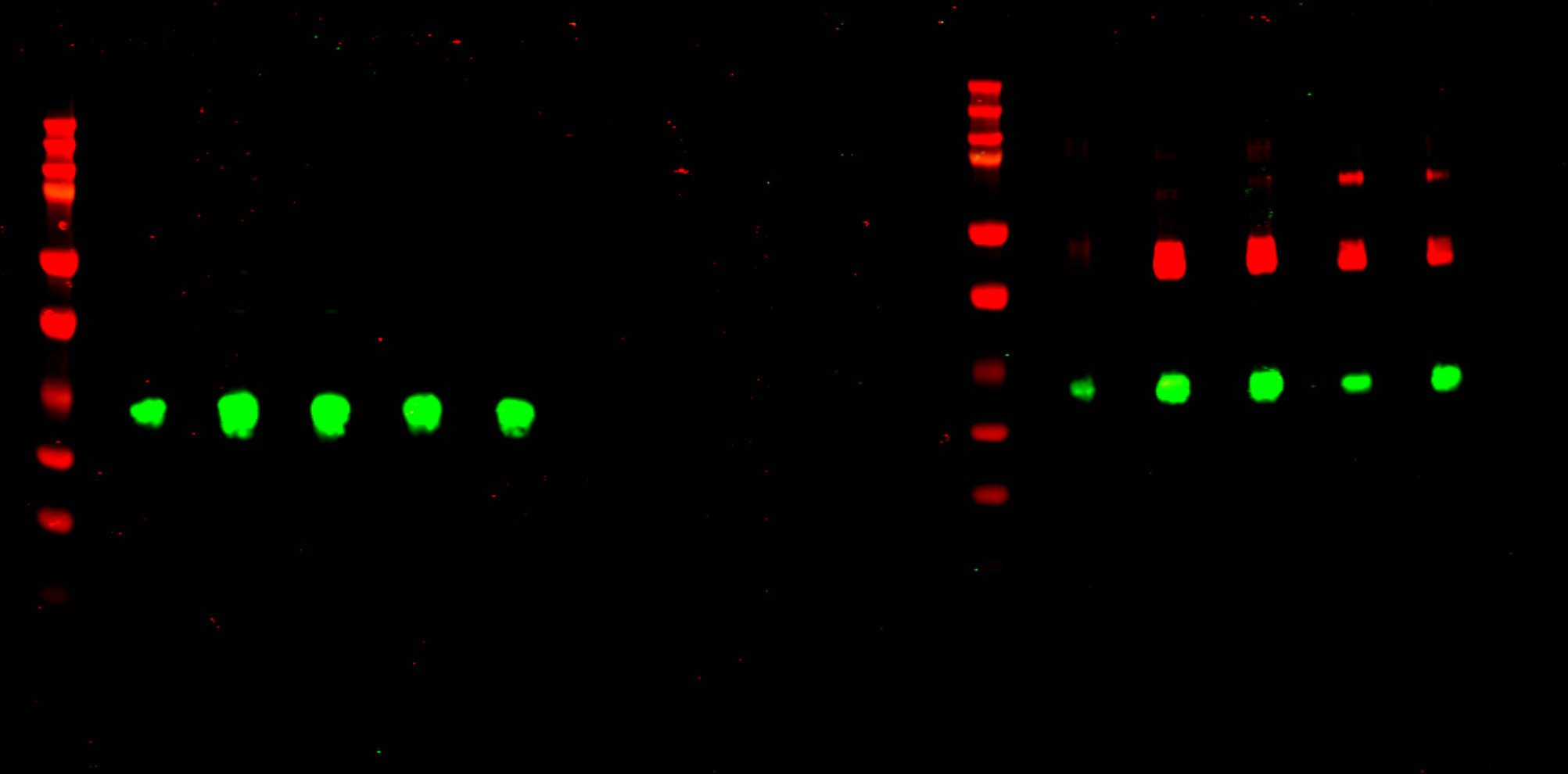

Supplement: Supplementary file 7 — Source data Fig. 2 [file 44319_2024_314_MOESM7_ESM.zip › Fig2_WB/Figure 2C-CCloop/Figure2-figure supplement 2-2-full.png]

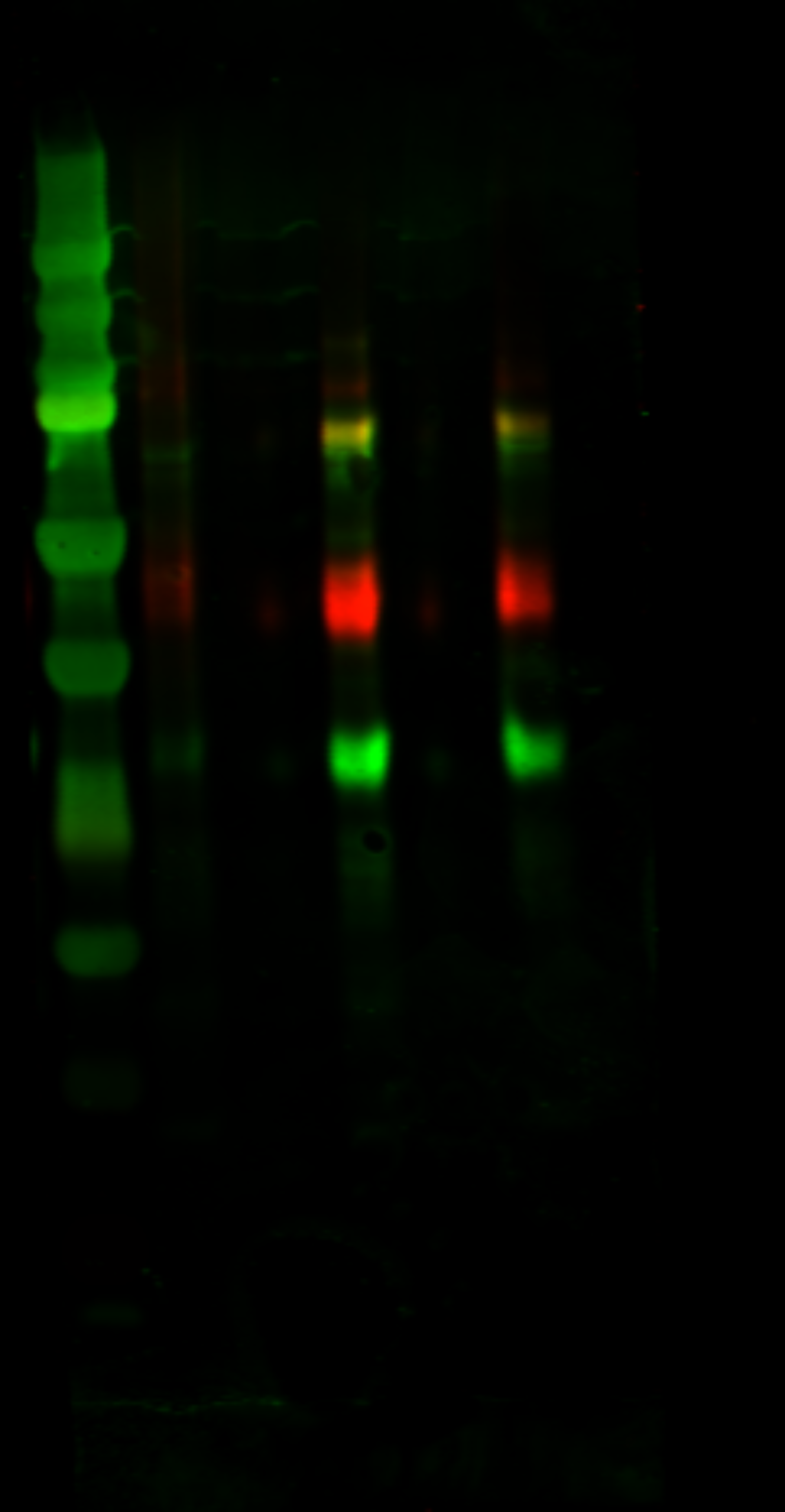

Supplement: Supplementary file 7 — Source data Fig. 2 [file 44319_2024_314_MOESM7_ESM.zip › Fig2_WB/Figure 2C-CCloop/Figure2C-full.png]

K166 V168

WT

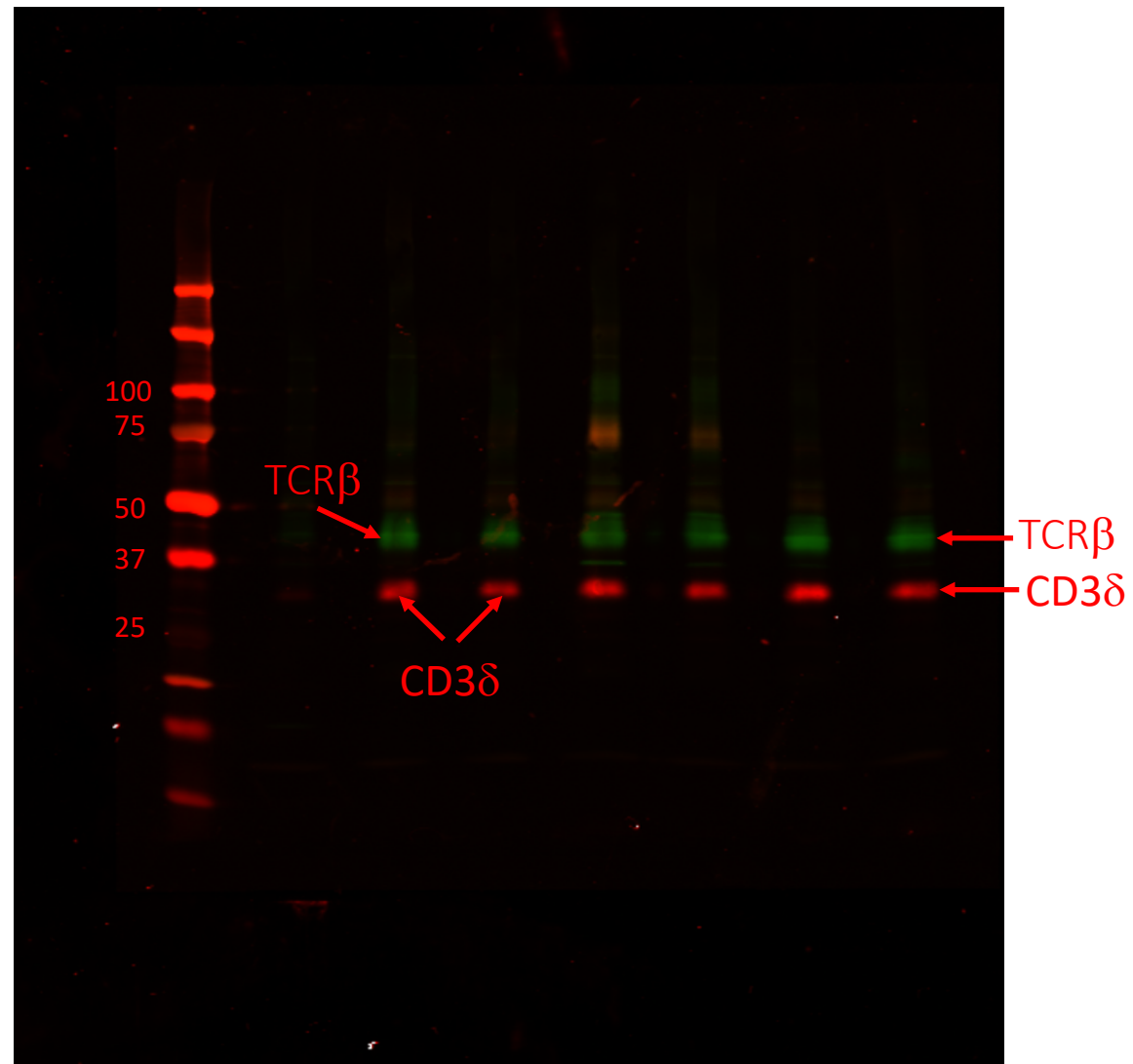

Supplement: Supplementary file 7 — Source data Fig. 2 [file 44319_2024_314_MOESM7_ESM.zip › Fig2_WB/Figure 2C-CCloop/Figure2-figure supplement 2- full-labeled.pdf]

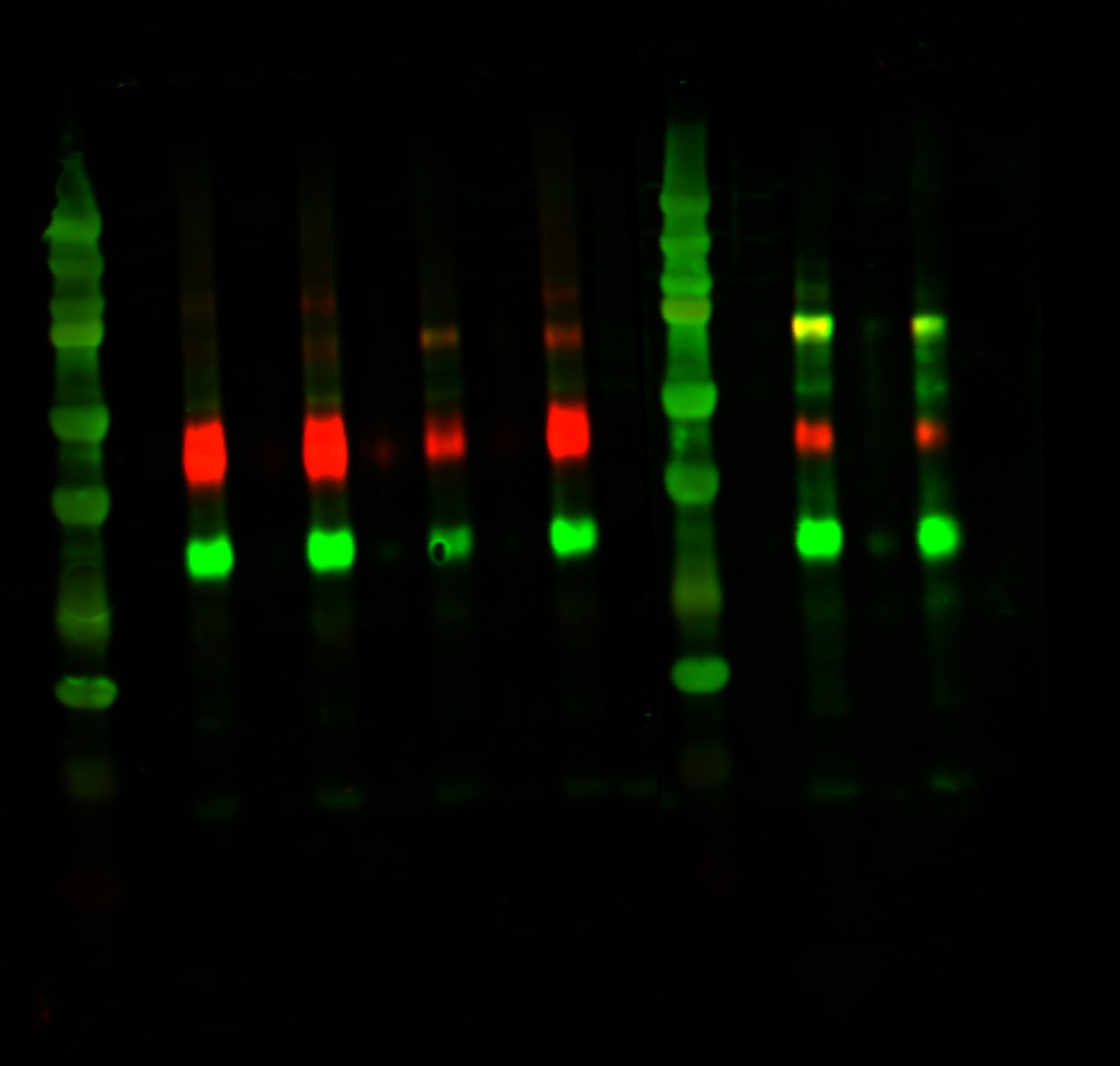

Supplement: Supplementary file 7 — Source data Fig. 2 [file 44319_2024_314_MOESM7_ESM.zip › Fig2_WB/Figure 2G-Helix3/I137Q139-full.png]

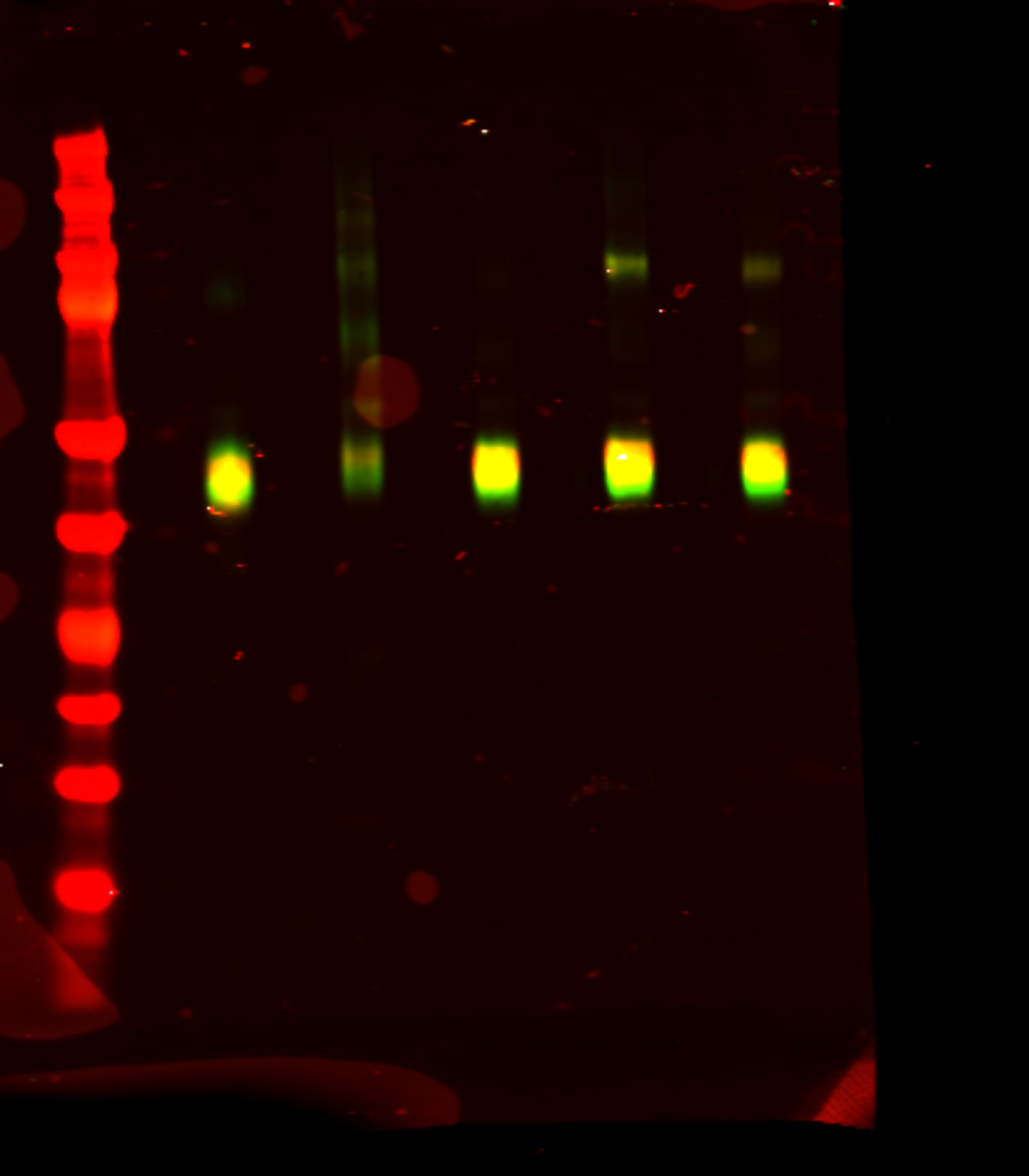

Supplement: Supplementary file 7 — Source data Fig. 2 [file 44319_2024_314_MOESM7_ESM.zip › Fig2_WB/Figure 2G-Helix3/E136A138K140K142-full.png]

## Slide 1
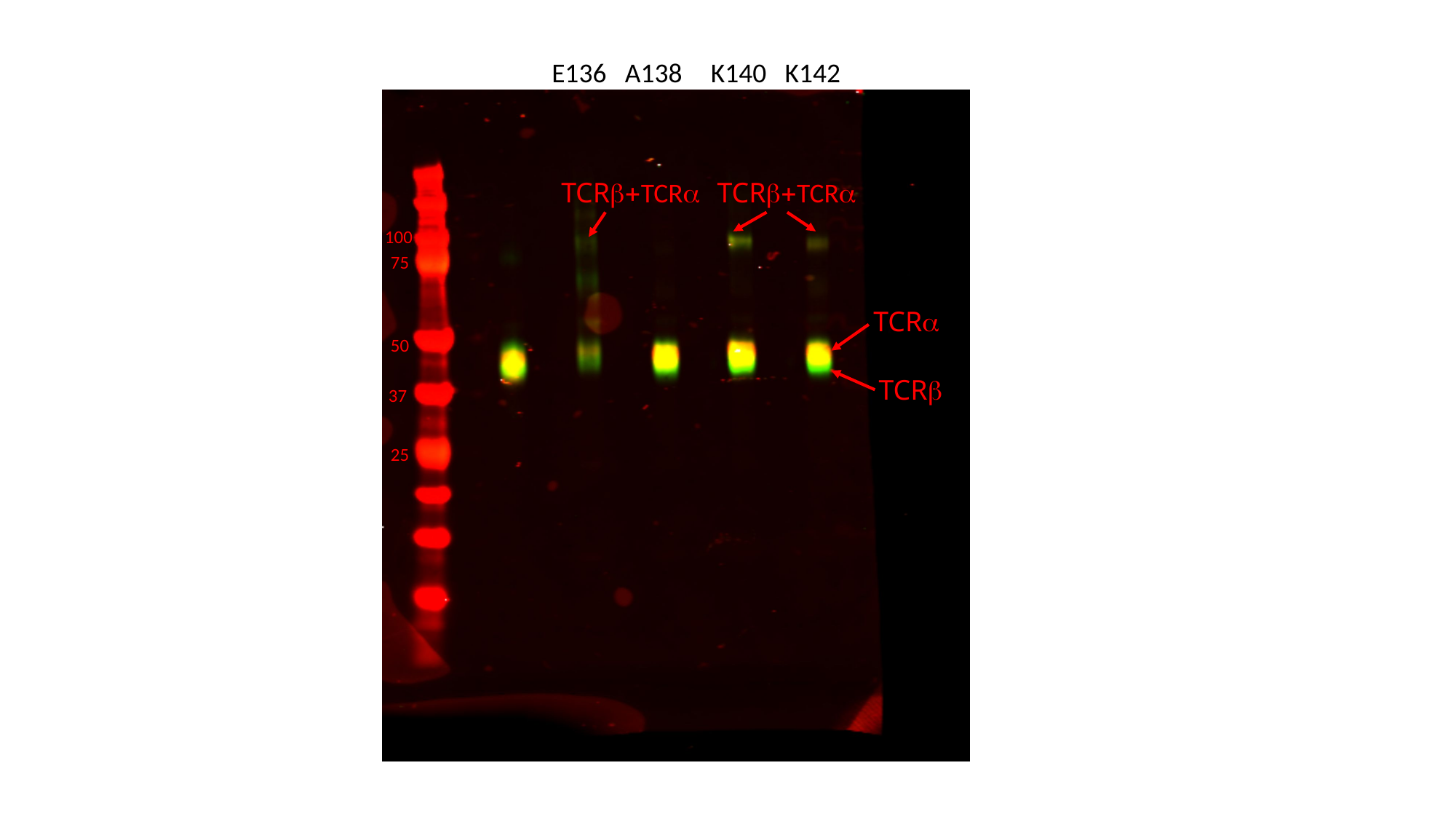

E136 A138
K140 K142
TCRb+TCRa
TCRb+TCRa
100
75
TCRa
50
TCRb
37
25

Supplement: Supplementary file 7 — Source data Fig. 2 [file 44319_2024_314_MOESM7_ESM.zip › Fig2_WB/Figure 2G-Helix3/E136A138K140K142-full.pptx]

## Slide 1
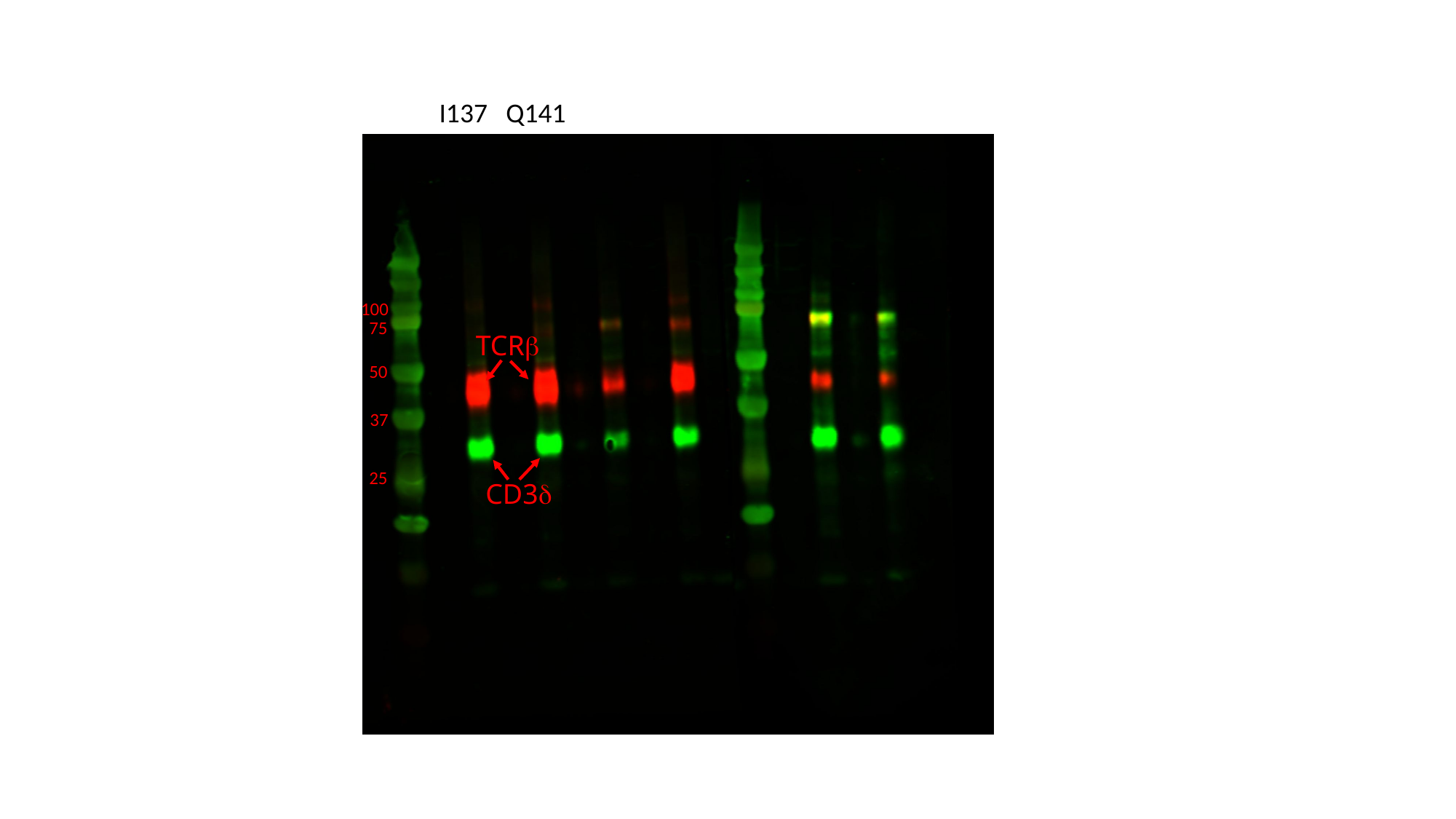

I137 Q141
100
75
TCRb
50
37
25
CD3d

Supplement: Supplementary file 7 — Source data Fig. 2 [file 44319_2024_314_MOESM7_ESM.zip › Fig2_WB/Figure 2G-Helix3/I137Q139-full.pptx]

E136 A138 K140 K142

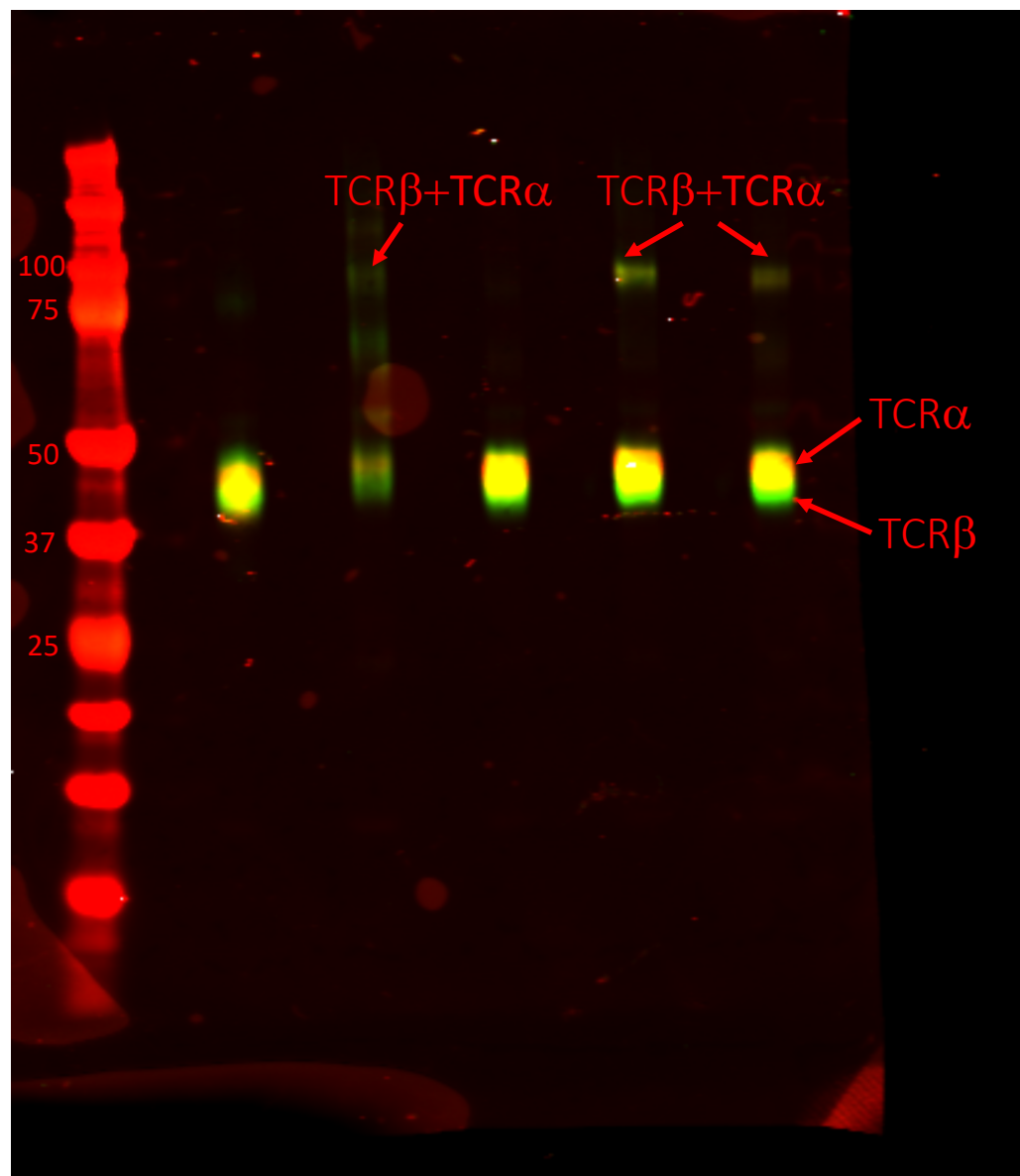

Supplement: Supplementary file 7 — Source data Fig. 2 [file 44319_2024_314_MOESM7_ESM.zip › Fig2_WB/Figure 2G-Helix3/E136A138K140K142-full-labeled.pdf]

I137 Q141

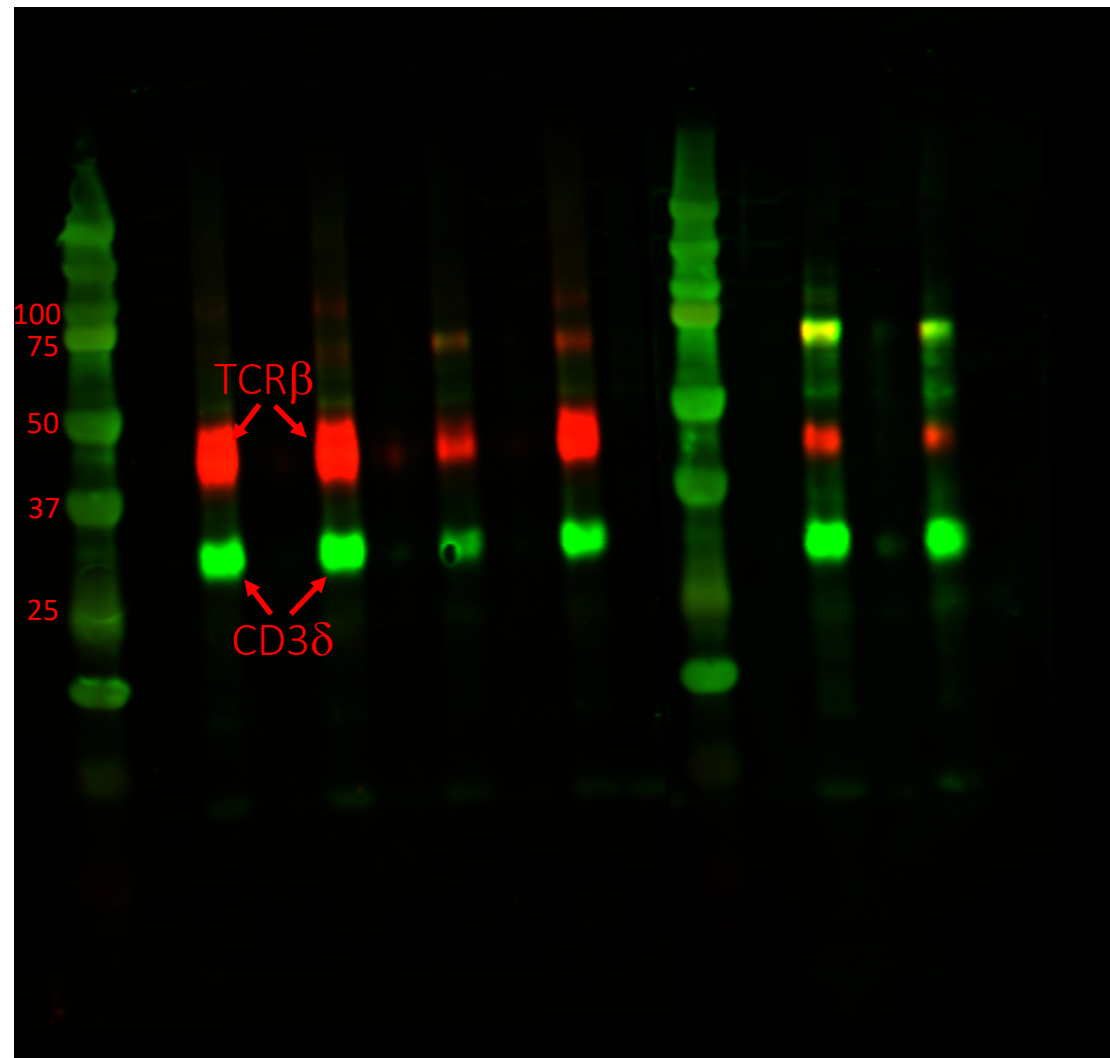

Supplement: Supplementary file 7 — Source data Fig. 2 [file 44319_2024_314_MOESM7_ESM.zip › Fig2_WB/Figure 2G-Helix3/I137Q139-full-labeled.pdf]

## Slide 1
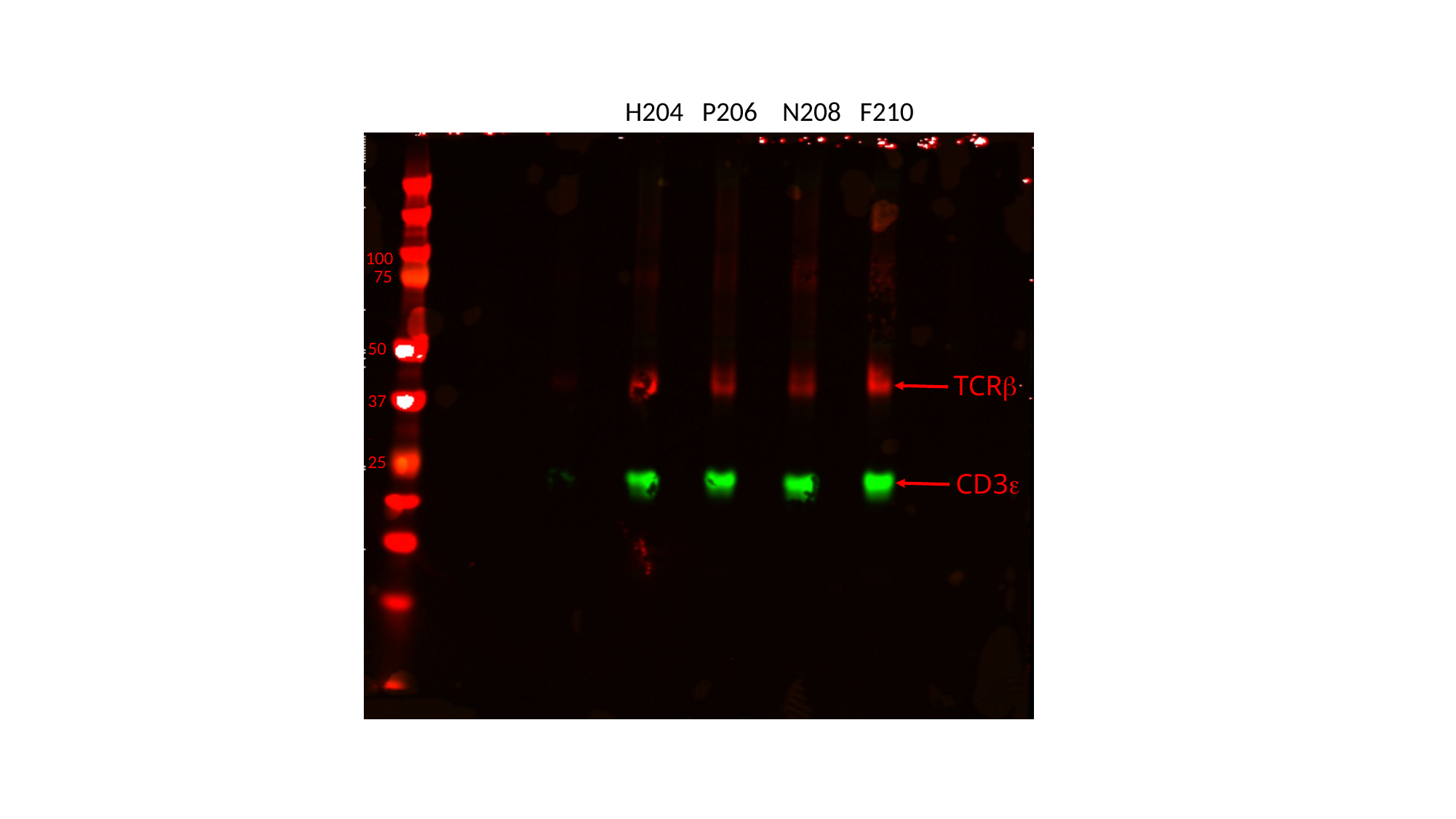

H204 P206 N208 F210
100
75
50
TCRb
37
25
CD3e

Supplement: Supplementary file 7 — Source data Fig. 2 [file 44319_2024_314_MOESM7_ESM.zip › Fig2_WB/Figure 2H-Helix4-Fstrand/H204P206N208F210-full.pptx]

## Slide 1
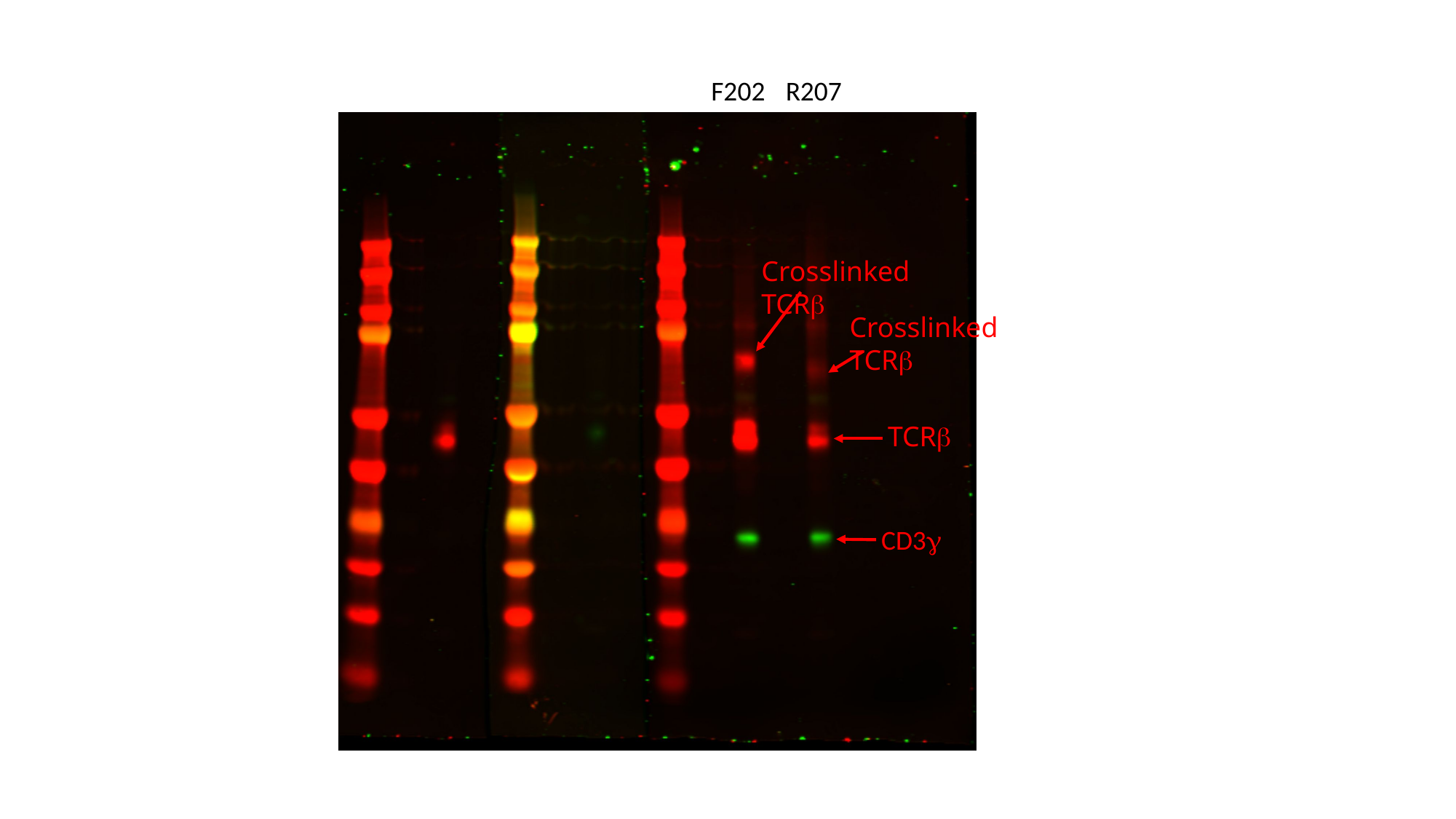

F202
R207
Crosslinked TCRb
Crosslinked TCRb
TCRb
CD3g

Supplement: Supplementary file 7 — Source data Fig. 2 [file 44319_2024_314_MOESM7_ESM.zip › Fig2_WB/Figure 2H-Helix4-Fstrand/F202R207-full-V5VSVG-labeled.pptx]

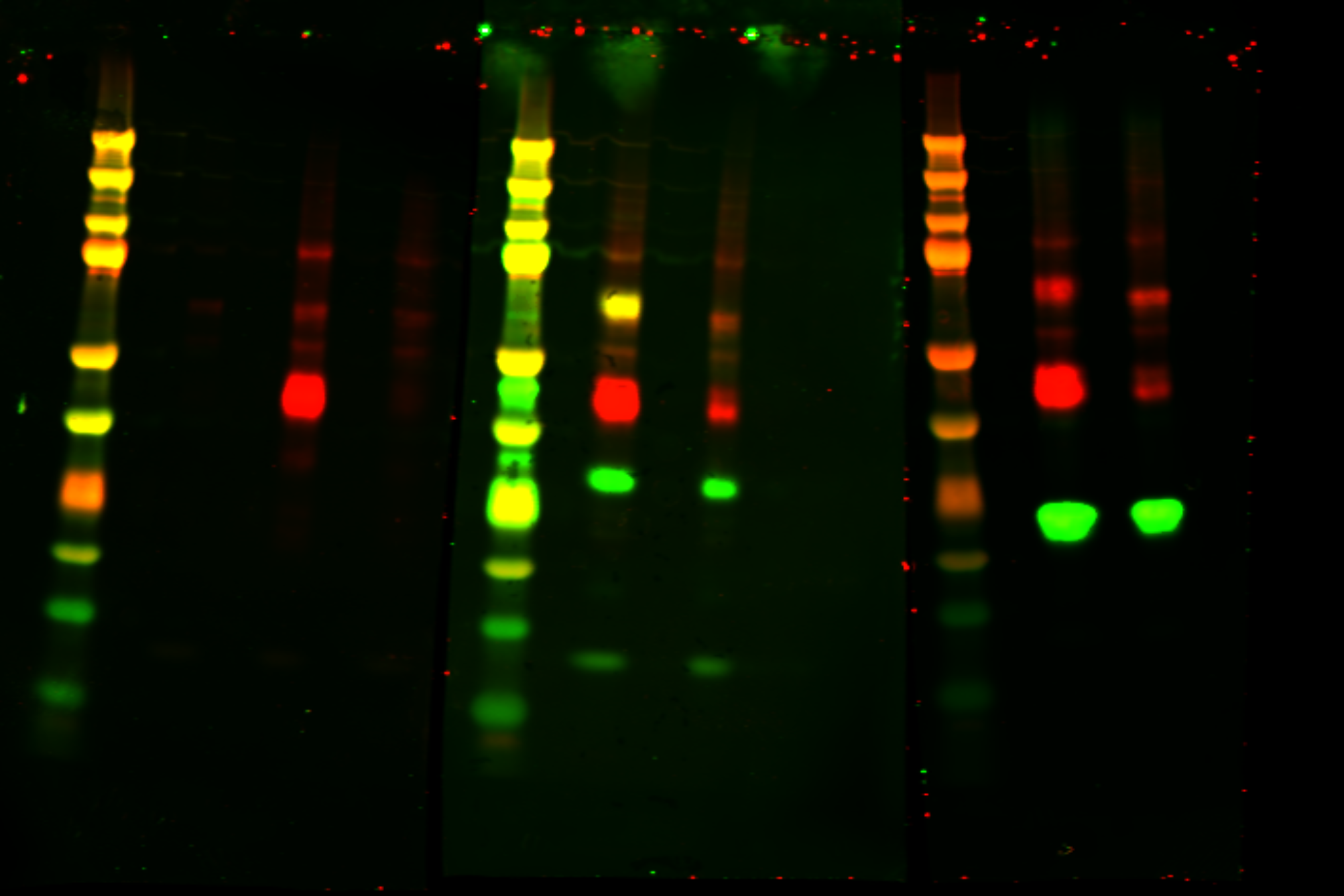

Supplement: Supplementary file 7 — Source data Fig. 2 [file 44319_2024_314_MOESM7_ESM.zip › Fig2_WB/Figure 2H-Helix4-Fstrand/F202R207-full-V5FLAG.png]

## Slide 1
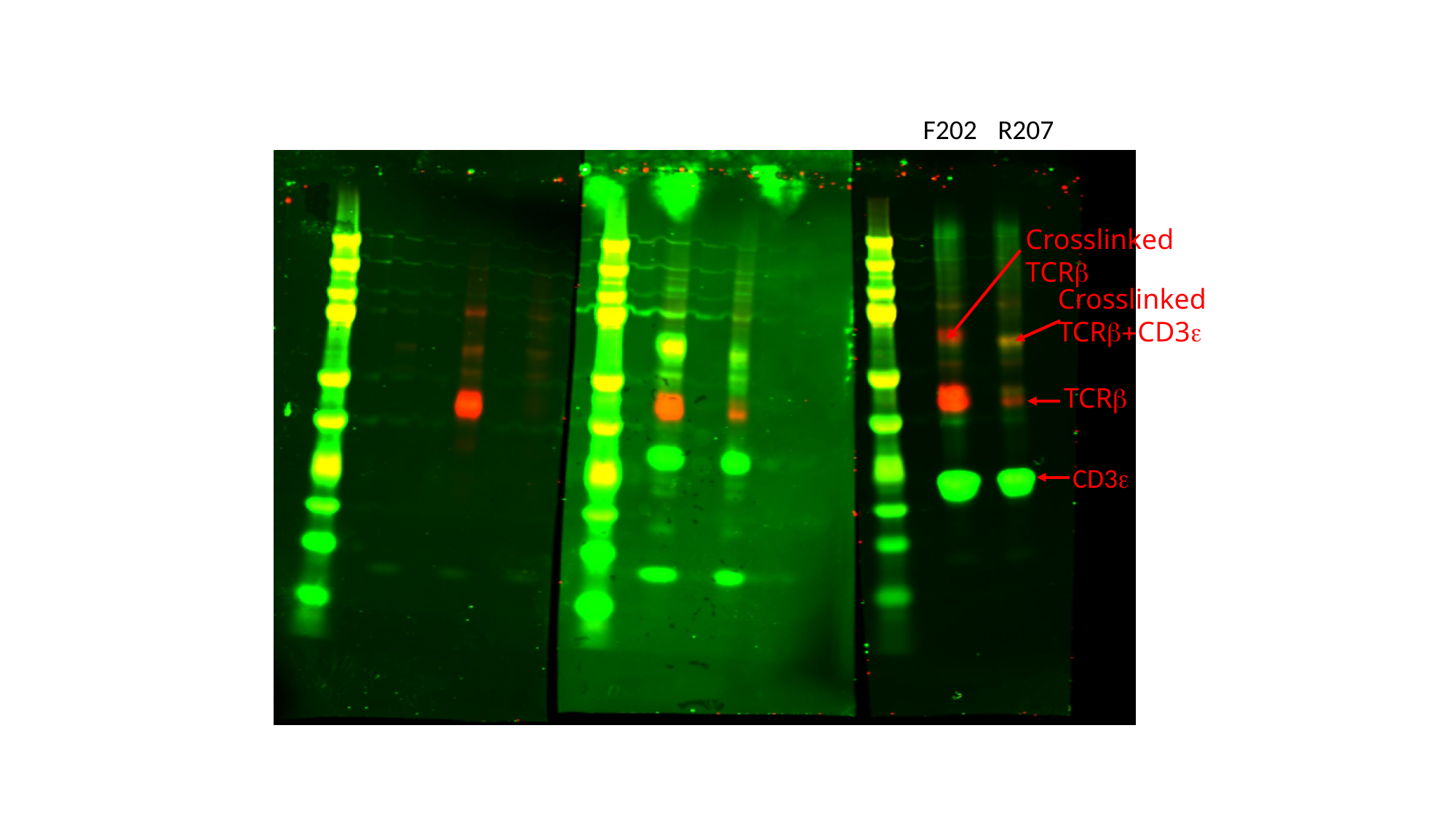

F202
R207
Crosslinked TCRb
Crosslinked TCRb+CD3e
TCRb
CD3e

Supplement: Supplementary file 7 — Source data Fig. 2 [file 44319_2024_314_MOESM7_ESM.zip › Fig2_WB/Figure 2H-Helix4-Fstrand/F202R207-full-V5HA-labeled.pptx]

F202 R207

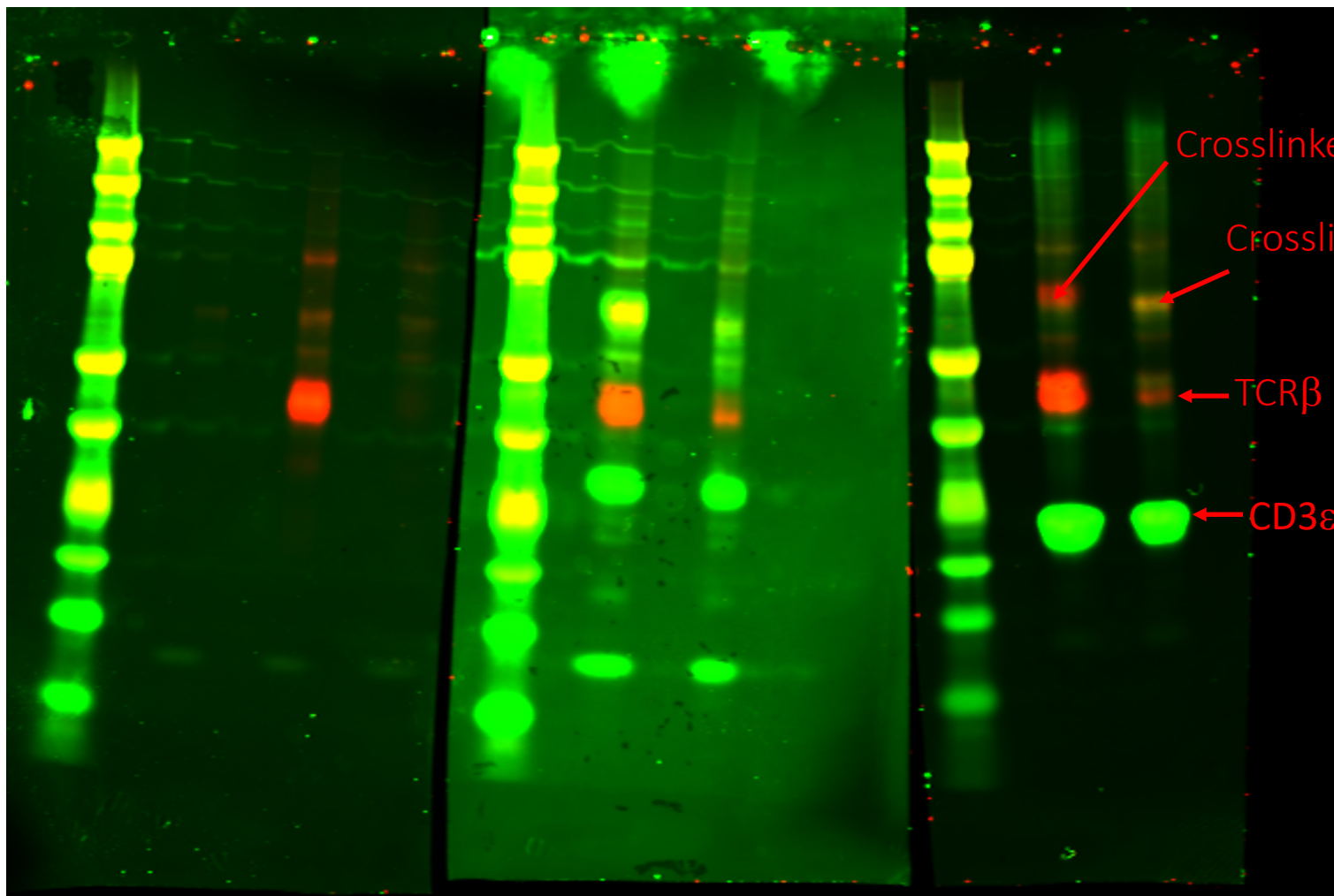

Supplement: Supplementary file 7 — Source data Fig. 2 [file 44319_2024_314_MOESM7_ESM.zip › Fig2_WB/Figure 2H-Helix4-Fstrand/F202R207-full-V5HA-labeled.pdf]

## Slide 1
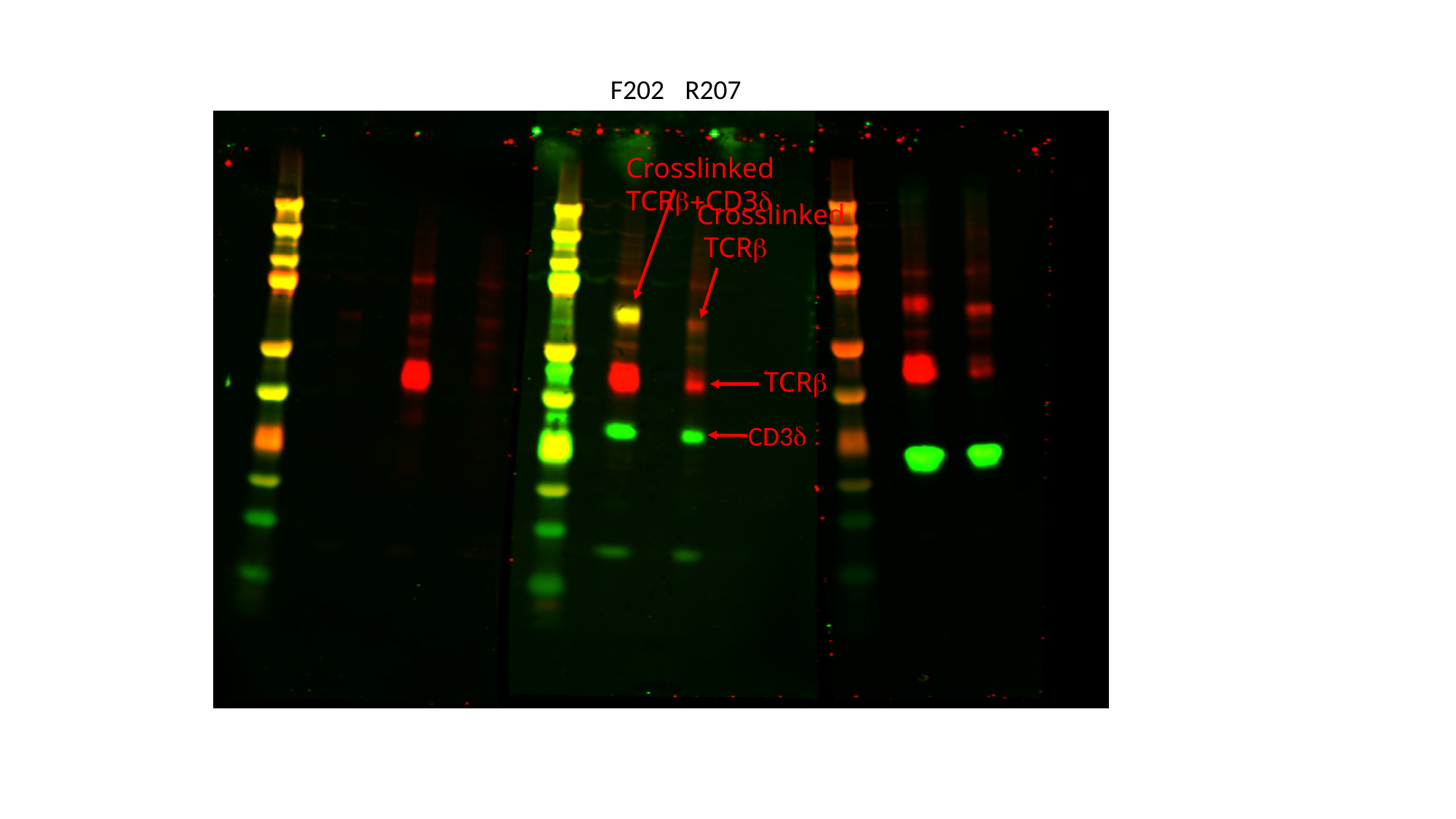

F202
R207
Crosslinked TCRb+CD3d
Crosslinked
 TCRb
TCRb
CD3d

Supplement: Supplementary file 7 — Source data Fig. 2 [file 44319_2024_314_MOESM7_ESM.zip › Fig2_WB/Figure 2H-Helix4-Fstrand/F202R207-full-V5FLAG-labeled.pptx]

F202 R207

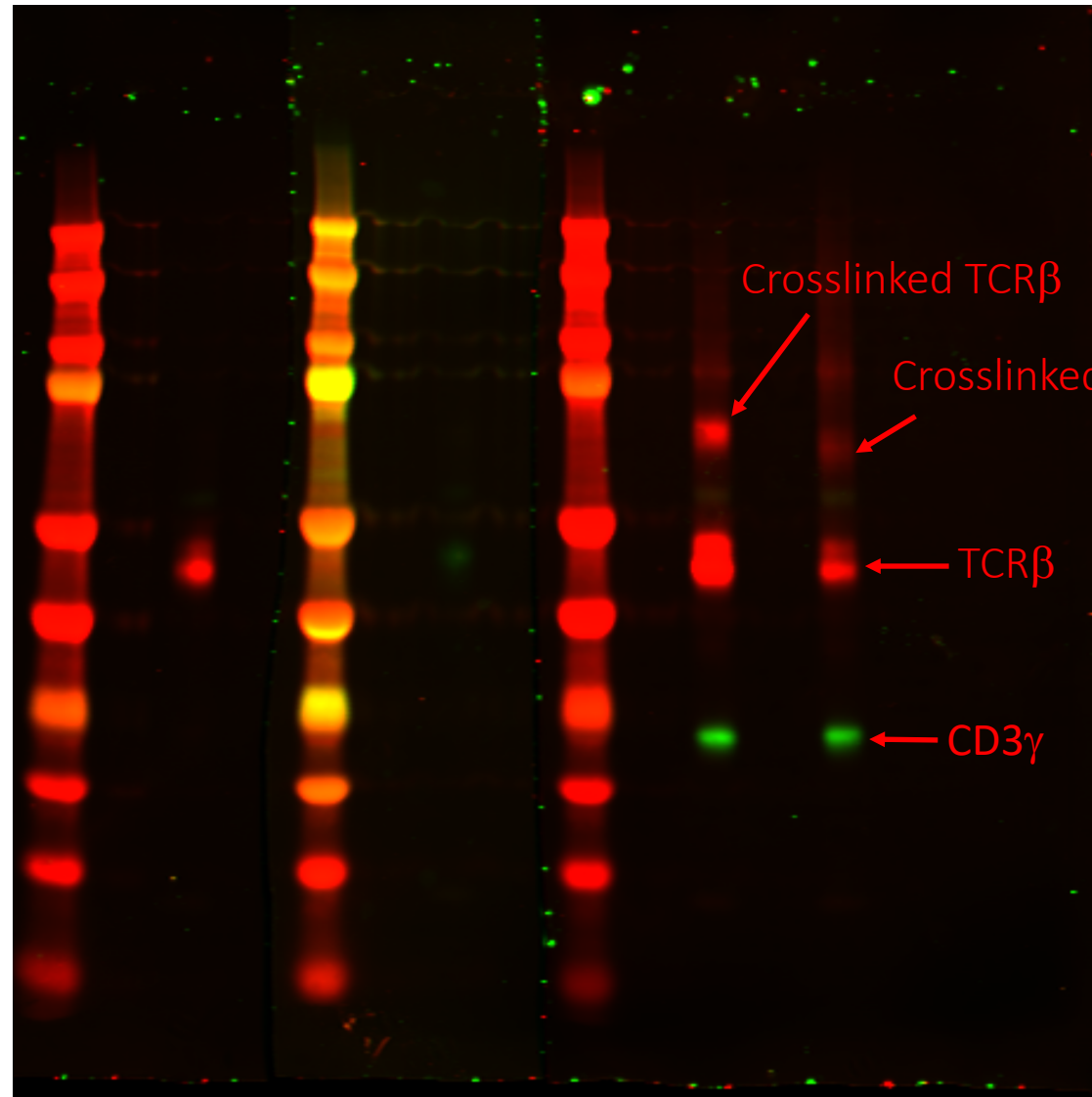

Supplement: Supplementary file 7 — Source data Fig. 2 [file 44319_2024_314_MOESM7_ESM.zip › Fig2_WB/Figure 2H-Helix4-Fstrand/F202R207-full-V5VSVG-labeled.pdf]

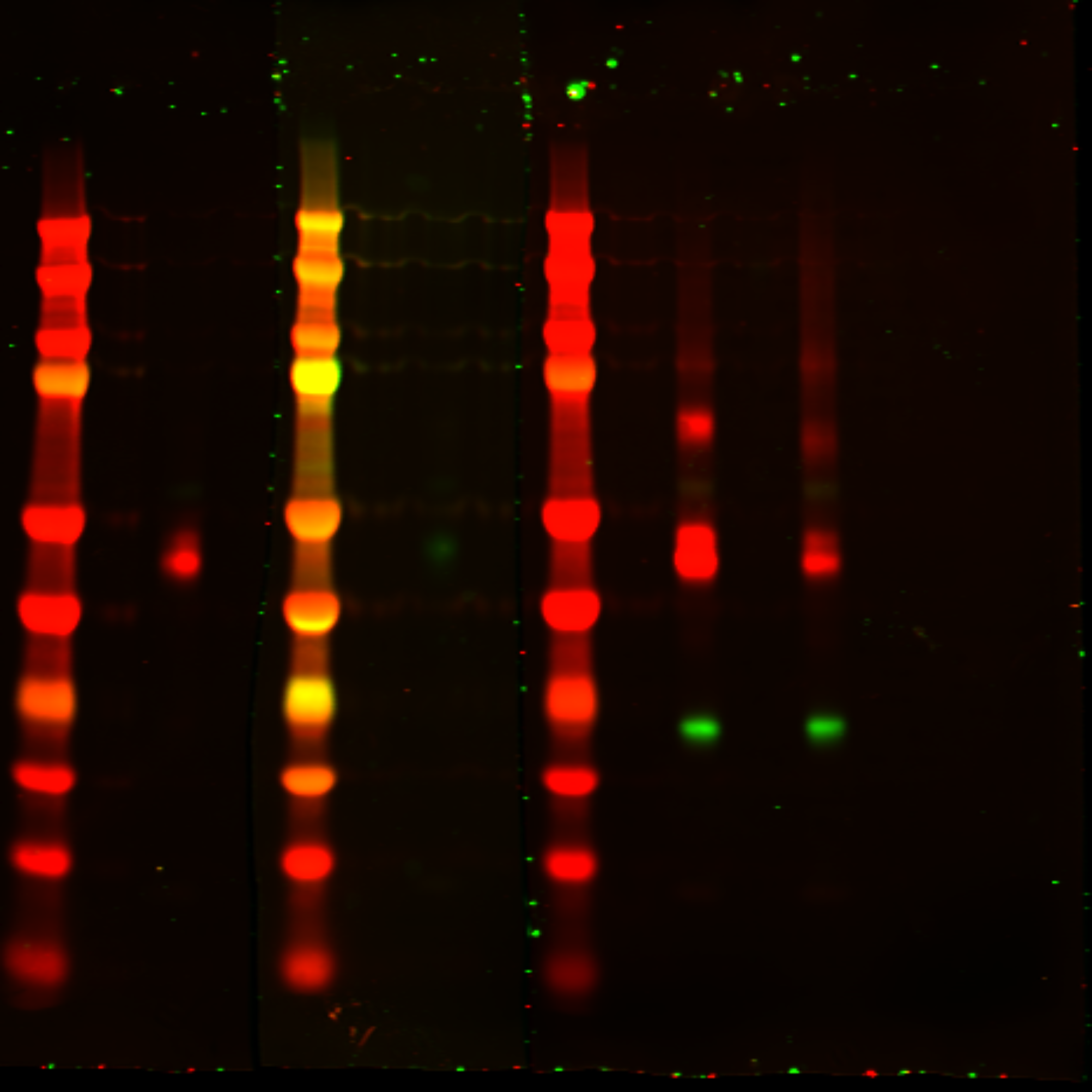

Supplement: Supplementary file 7 — Source data Fig. 2 [file 44319_2024_314_MOESM7_ESM.zip › Fig2_WB/Figure 2H-Helix4-Fstrand/F202R207-full-V5VSVG.png]

H204 P206 N208 F210

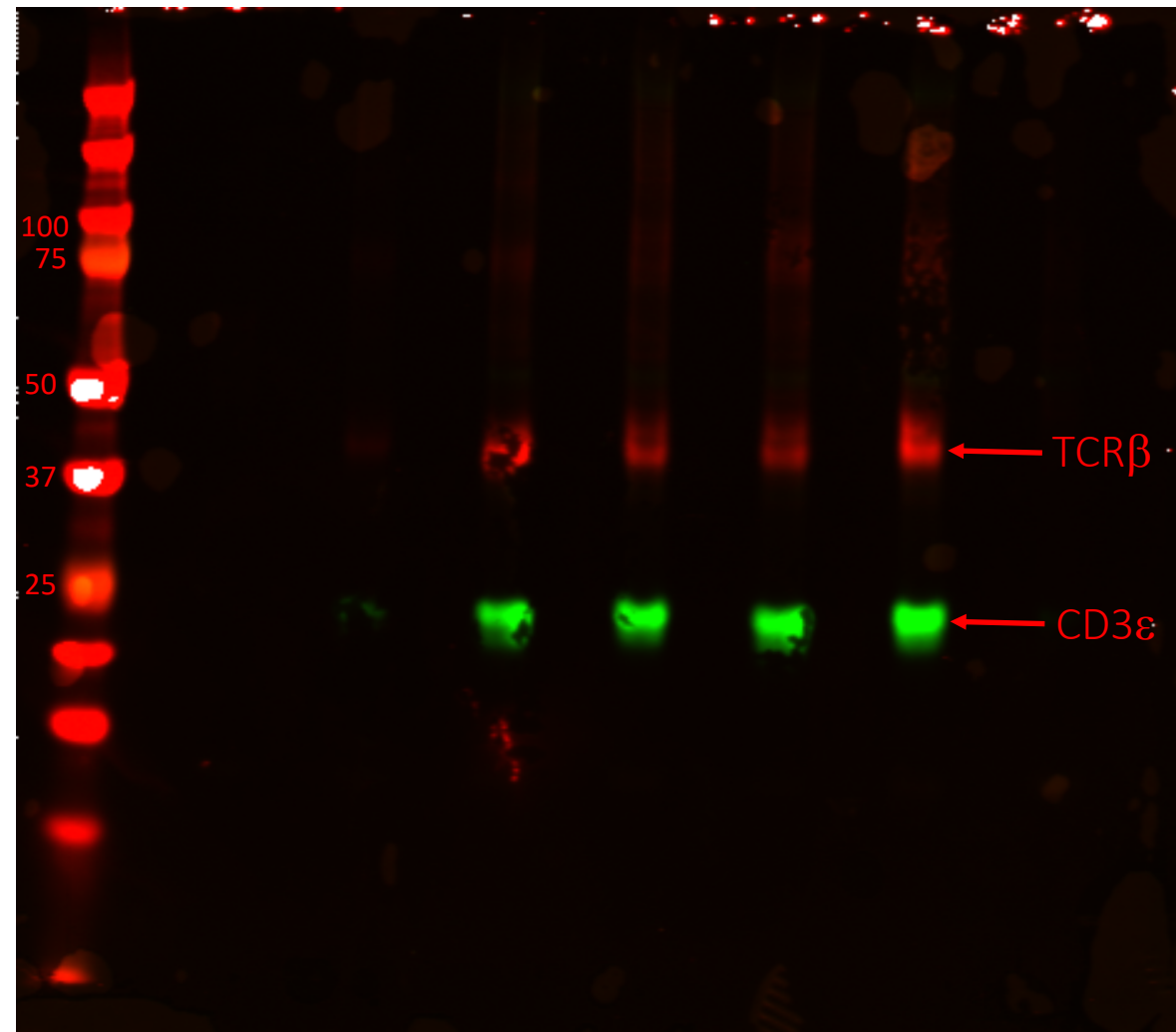

Supplement: Supplementary file 7 — Source data Fig. 2 [file 44319_2024_314_MOESM7_ESM.zip › Fig2_WB/Figure 2H-Helix4-Fstrand/H204P206N208F210-full-labeled.pdf]

F202 R207

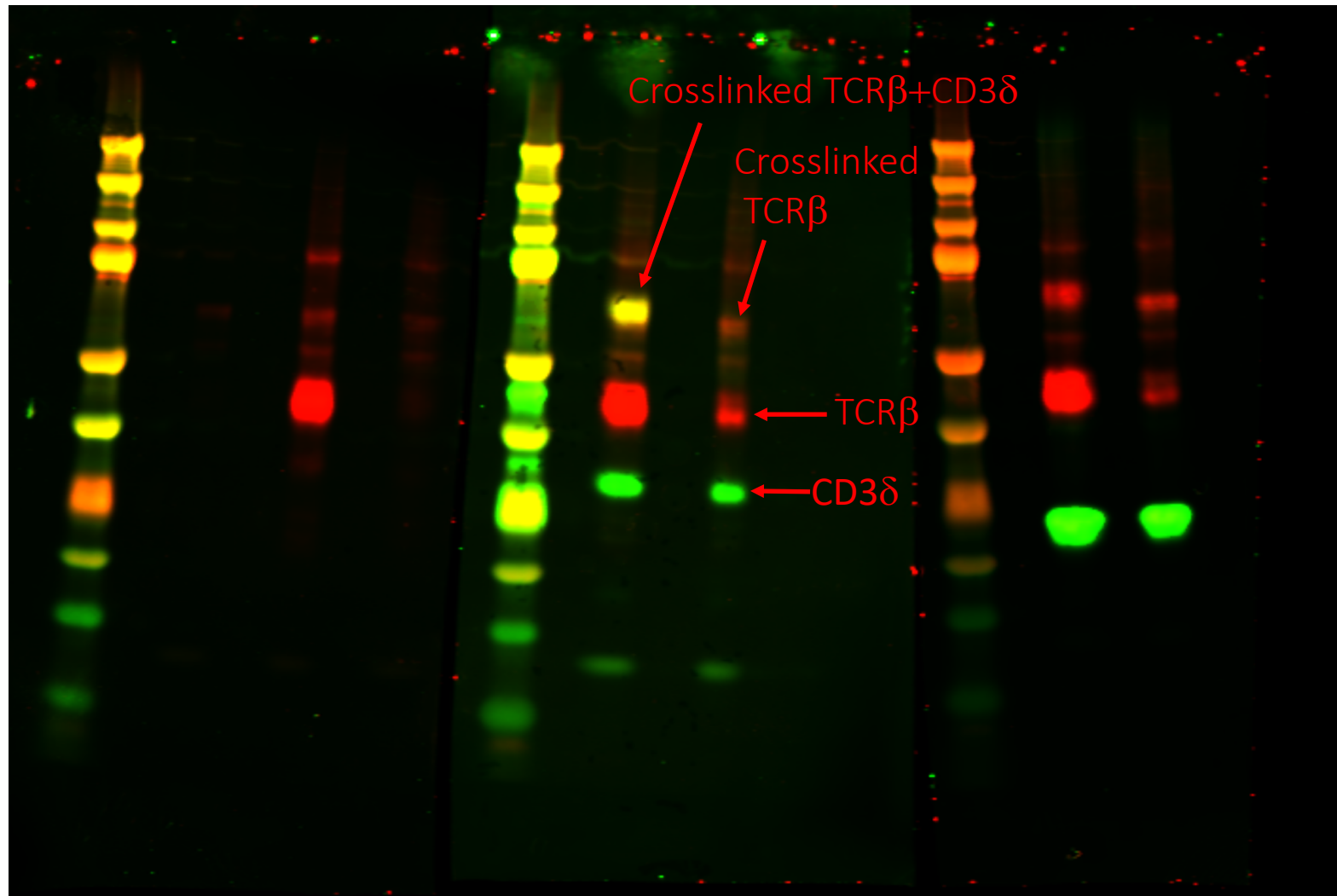

Supplement: Supplementary file 7 — Source data Fig. 2 [file 44319_2024_314_MOESM7_ESM.zip › Fig2_WB/Figure 2H-Helix4-Fstrand/F202R207-full-V5FLAG-labeled.pdf]

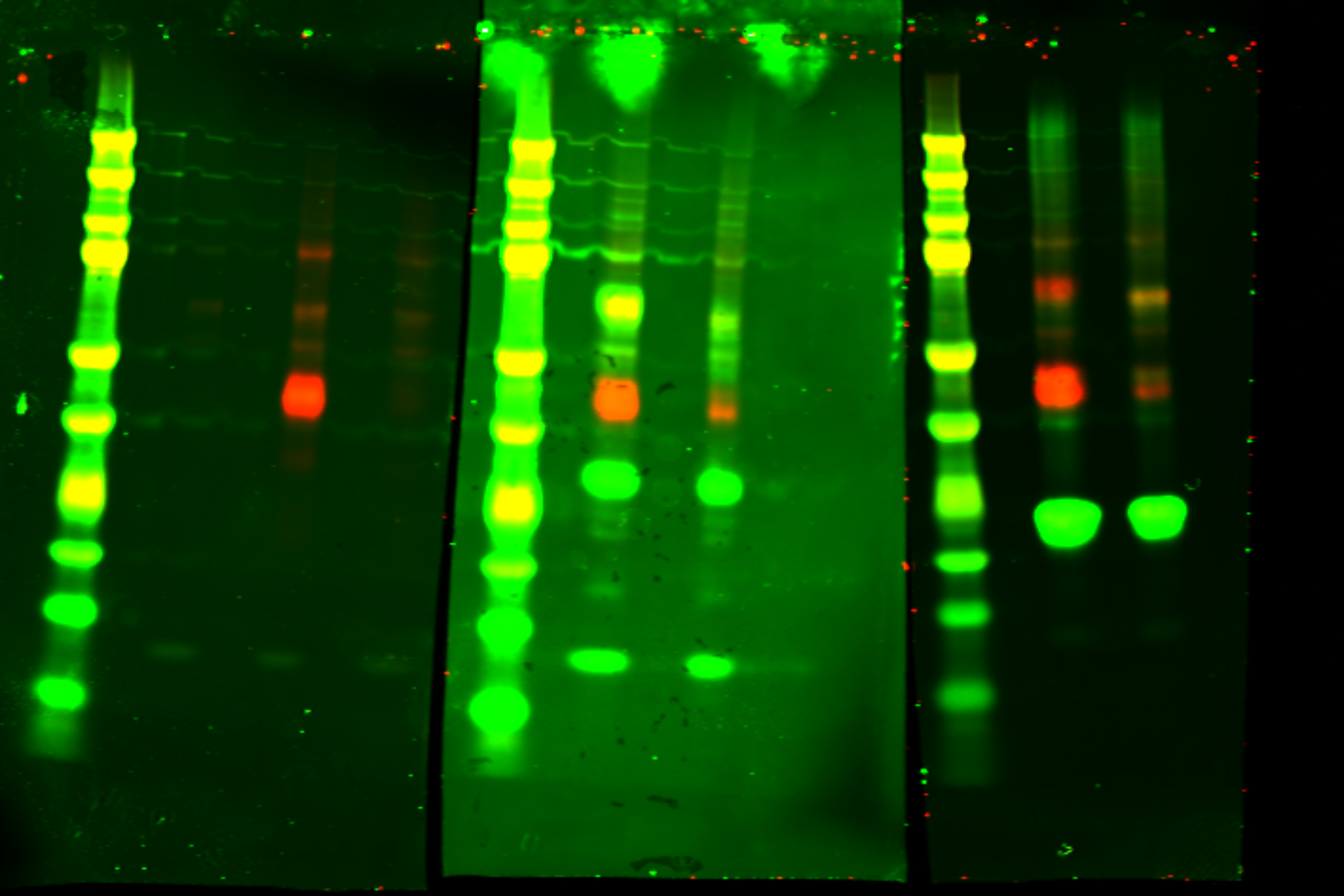

Supplement: Supplementary file 7 — Source data Fig. 2 [file 44319_2024_314_MOESM7_ESM.zip › Fig2_WB/Figure 2H-Helix4-Fstrand/F202R207-full-V5HA.png]

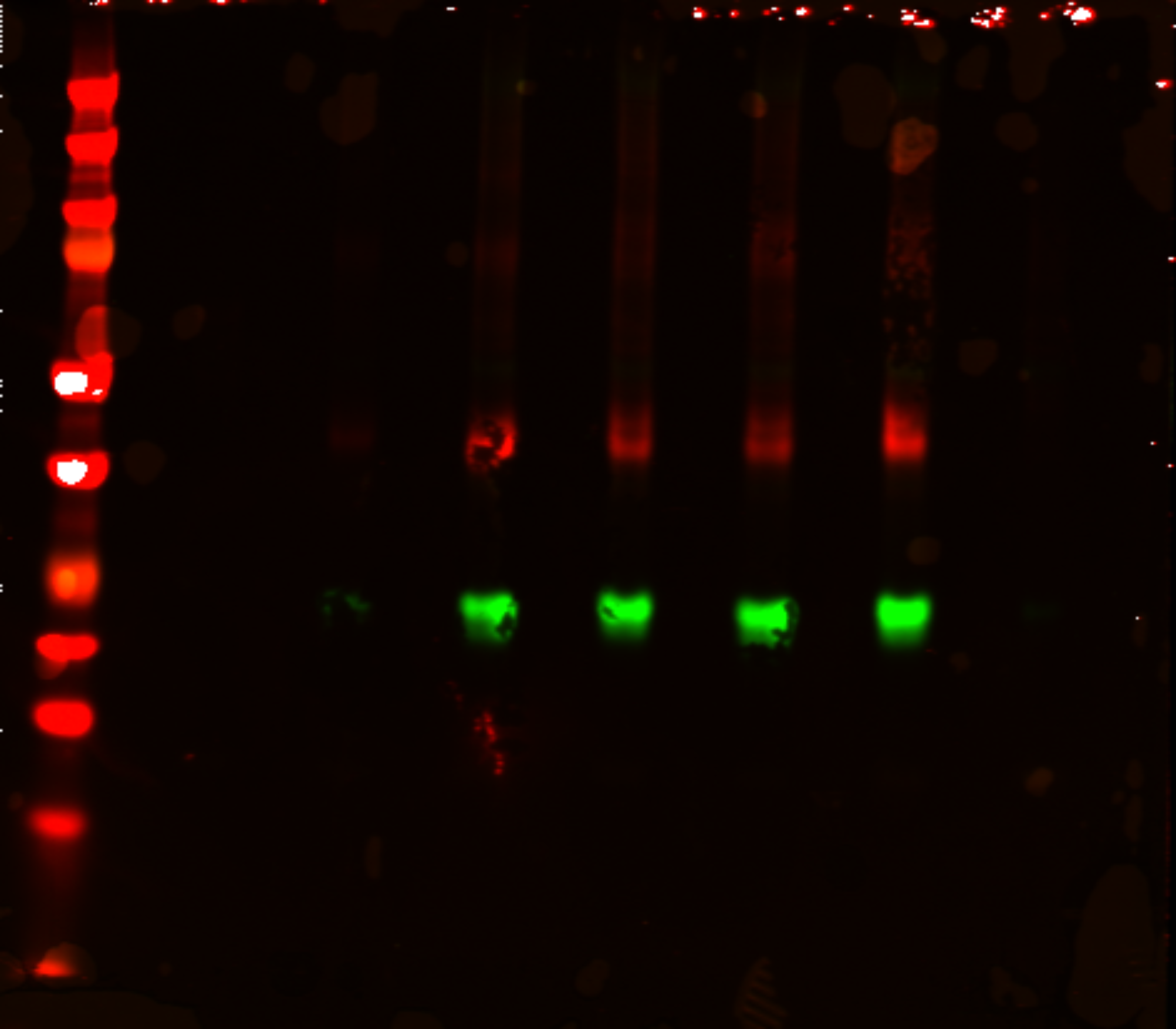

Supplement: Supplementary file 7 — Source data Fig. 2 [file 44319_2024_314_MOESM7_ESM.zip › Fig2_WB/Figure 2H-Helix4-Fstrand/H204P206N208F210-full.png]

S238 W242

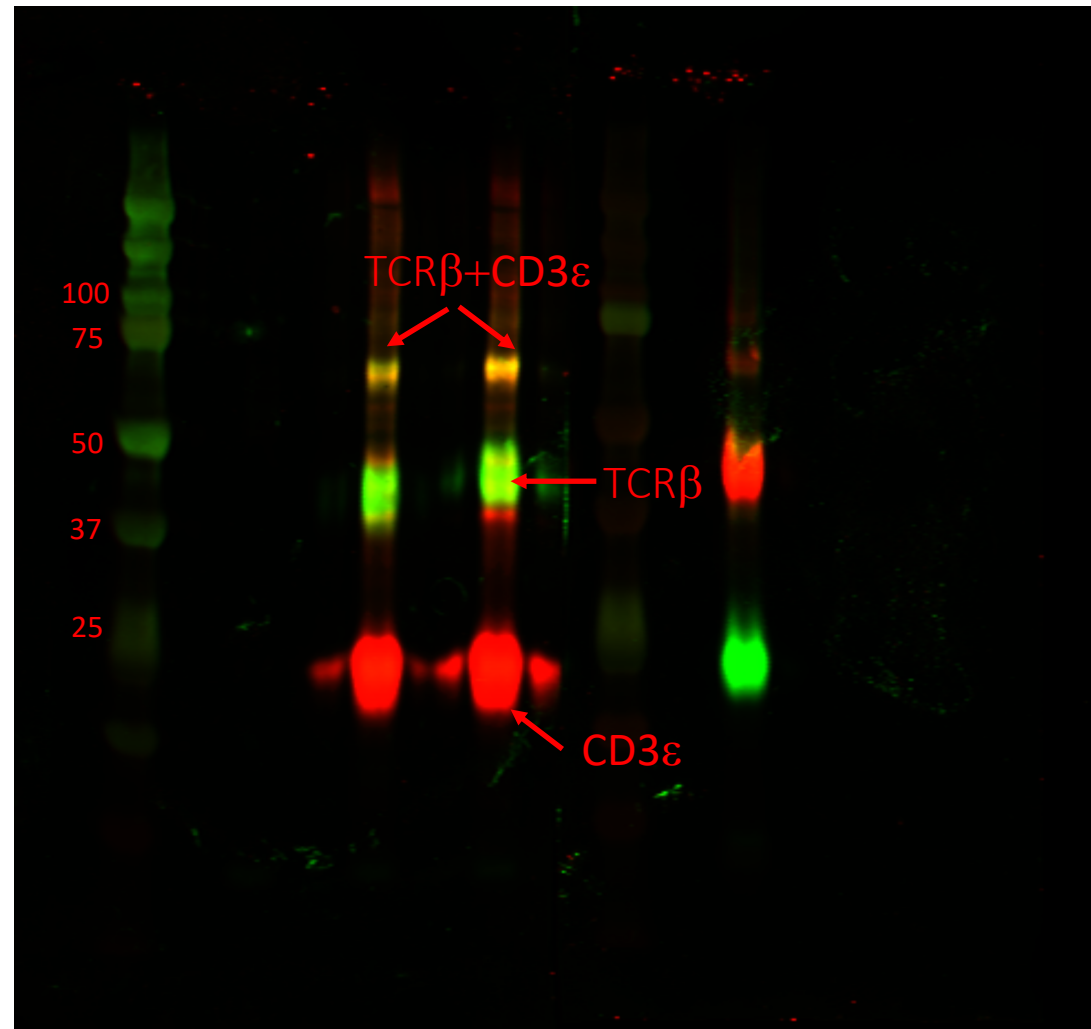

Supplement: Supplementary file 7 — Source data Fig. 2 [file 44319_2024_314_MOESM7_ESM.zip › Fig2_WB/Figure 2F-Gstrand/Gs-S238-W242-merged-labeled.pdf]

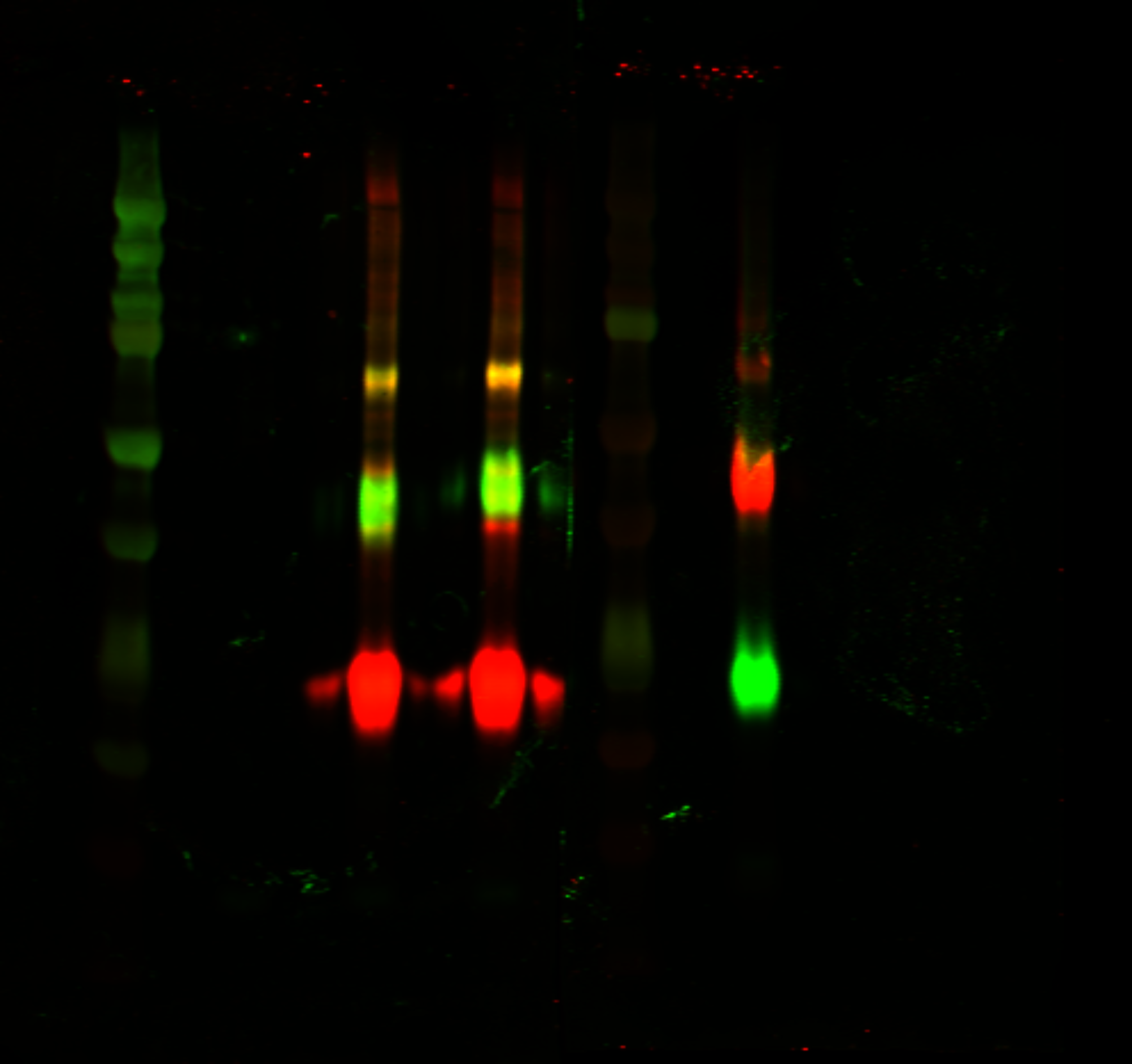

Supplement: Supplementary file 7 — Source data Fig. 2 [file 44319_2024_314_MOESM7_ESM.zip › Fig2_WB/Figure 2F-Gstrand/Gs-S238-W242-merged.png]

N236 S238 E240 W242 R244

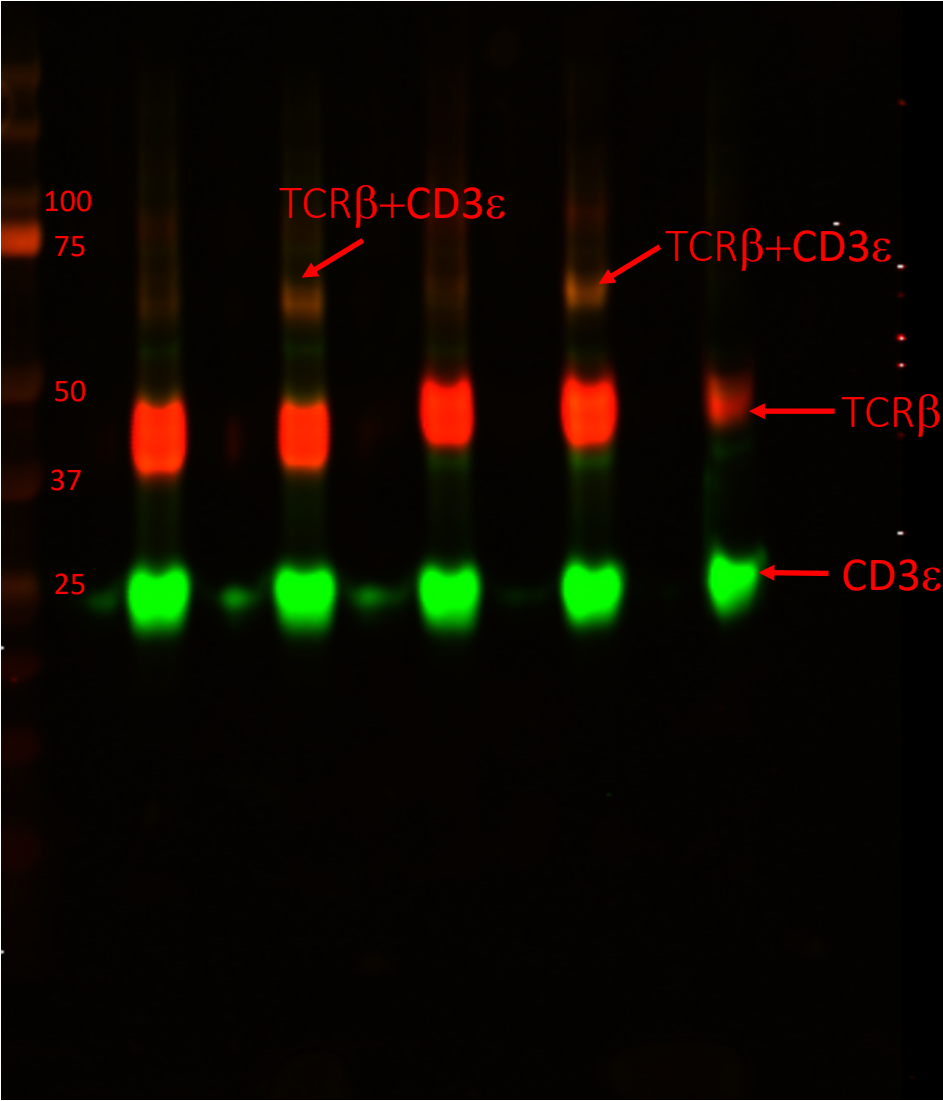

Supplement: Supplementary file 7 — Source data Fig. 2 [file 44319_2024_314_MOESM7_ESM.zip › Fig2_WB/Figure 2F-Gstrand/N236S238E240W242R244-full-labeled.pdf]

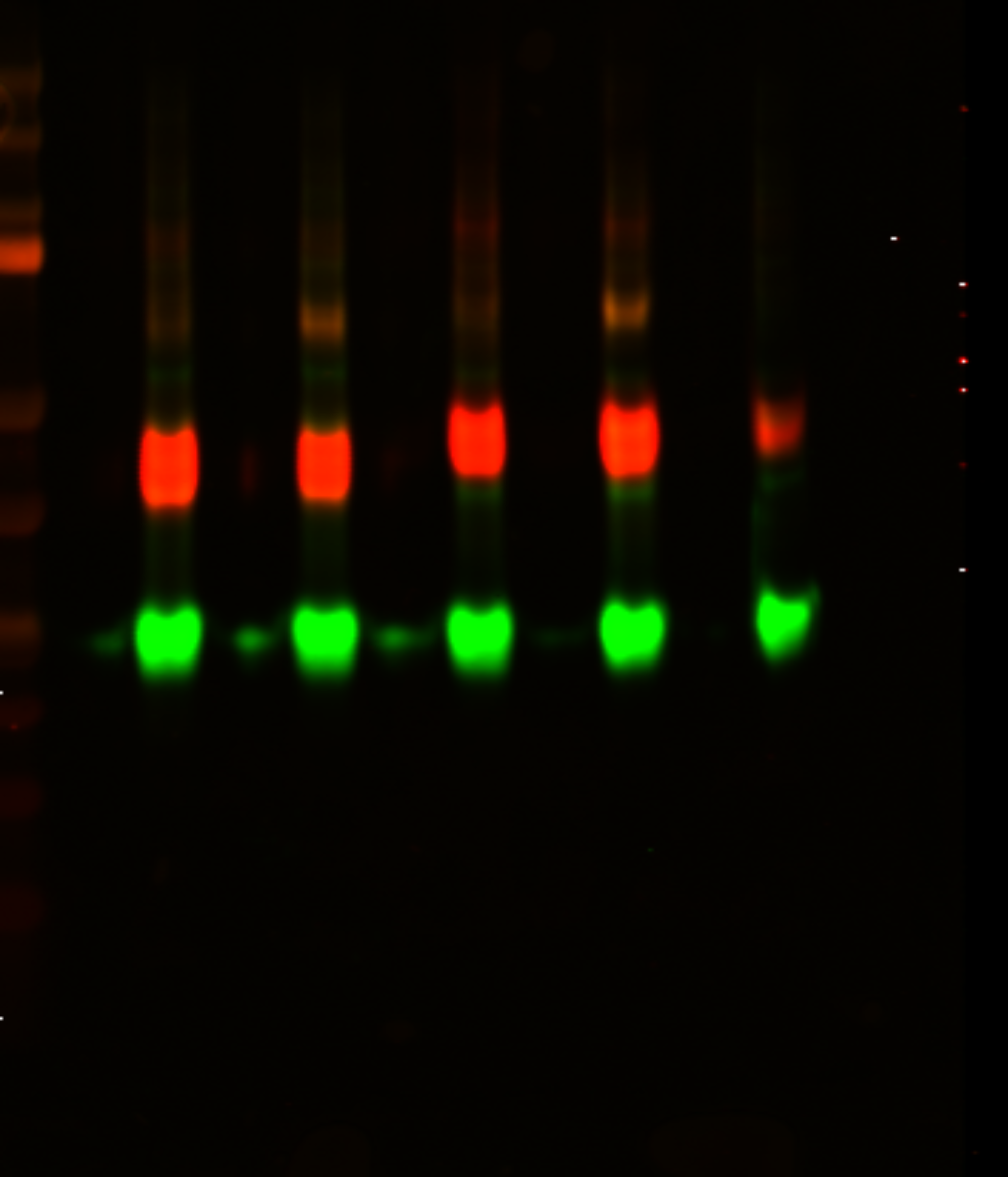

Supplement: Supplementary file 7 — Source data Fig. 2 [file 44319_2024_314_MOESM7_ESM.zip › Fig2_WB/Figure 2F-Gstrand/N236S238E240W242R244-full.png]

## Slide 1
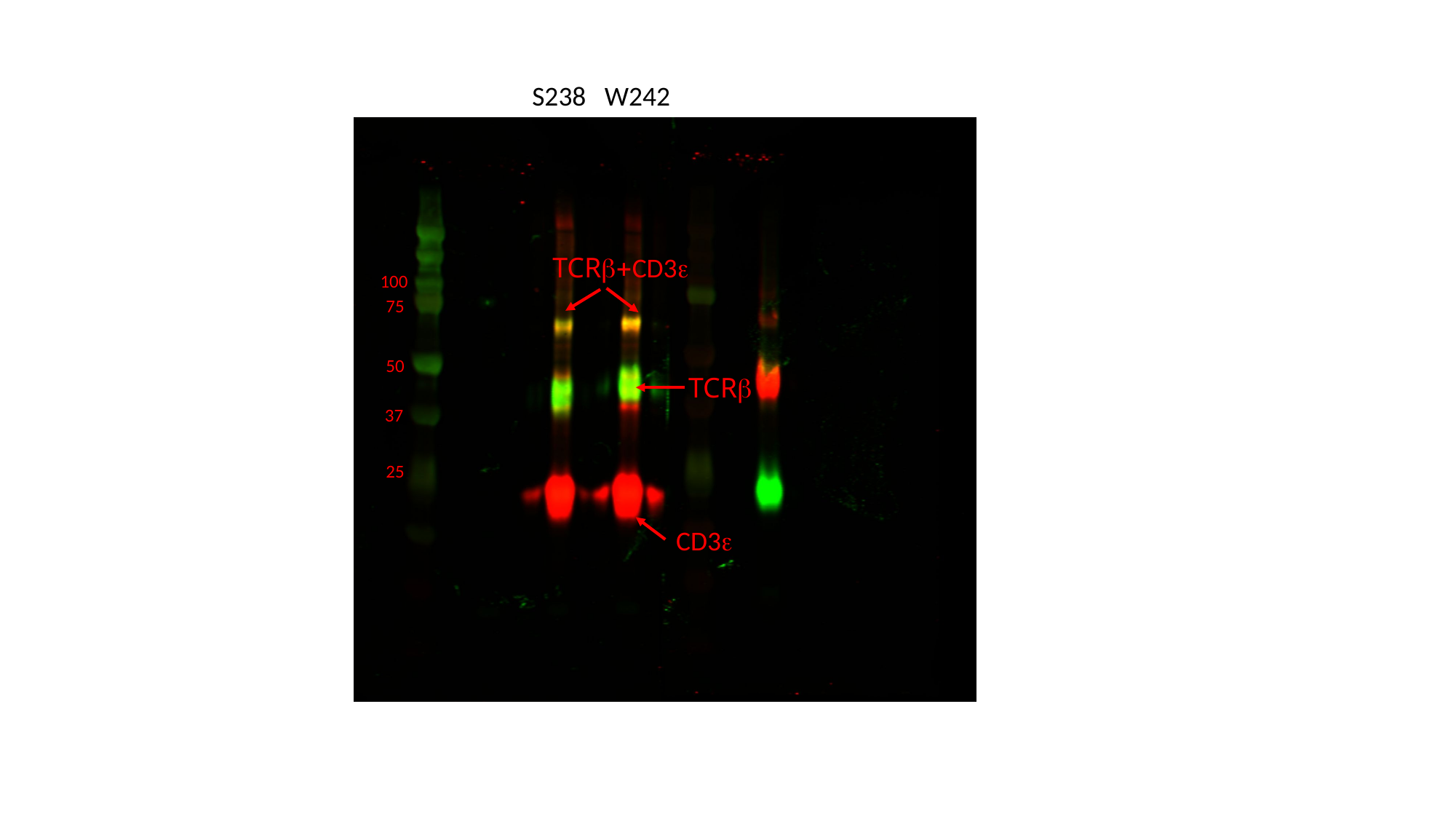

S238 W242
TCRb+CD3e
100
75
50
TCRb
37
25
CD3e

Supplement: Supplementary file 7 — Source data Fig. 2 [file 44319_2024_314_MOESM7_ESM.zip › Fig2_WB/Figure 2F-Gstrand/Gs-S238-W242-merged-labeled.pptx]

S238

W242

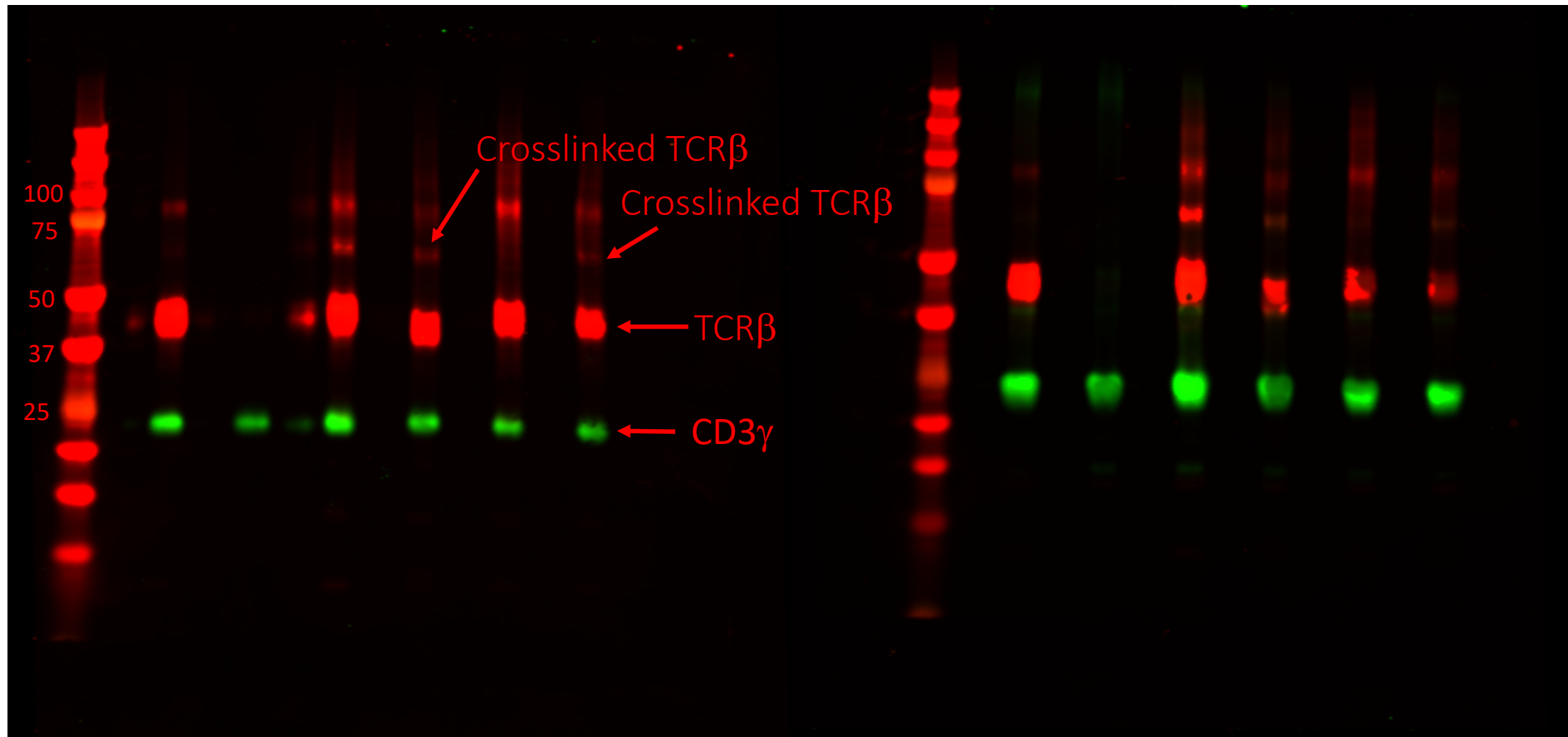

Supplement: Supplementary file 7 — Source data Fig. 2 [file 44319_2024_314_MOESM7_ESM.zip › Fig2_WB/Figure 2F-Gstrand/S238W242-VSVG-full-labeled.pdf]

## Slide 1
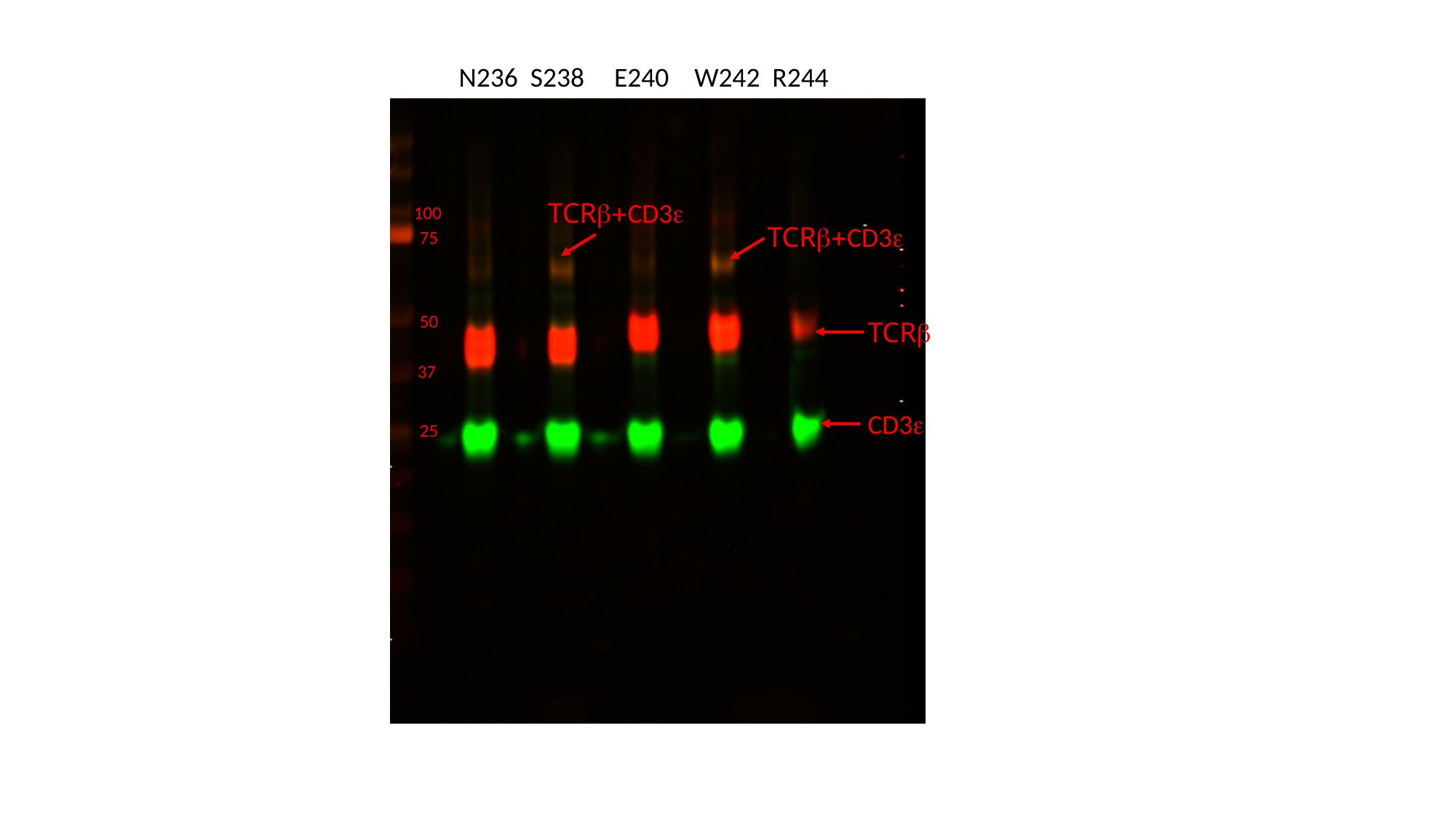

N236 S238
E240
W242 R244
TCRb+CD3e
100
TCRb+CD3e
75
50
TCRb
37
CD3e
25

Supplement: Supplementary file 7 — Source data Fig. 2 [file 44319_2024_314_MOESM7_ESM.zip › Fig2_WB/Figure 2F-Gstrand/N236S238E240W242R244-full.pptx]

## Slide 1
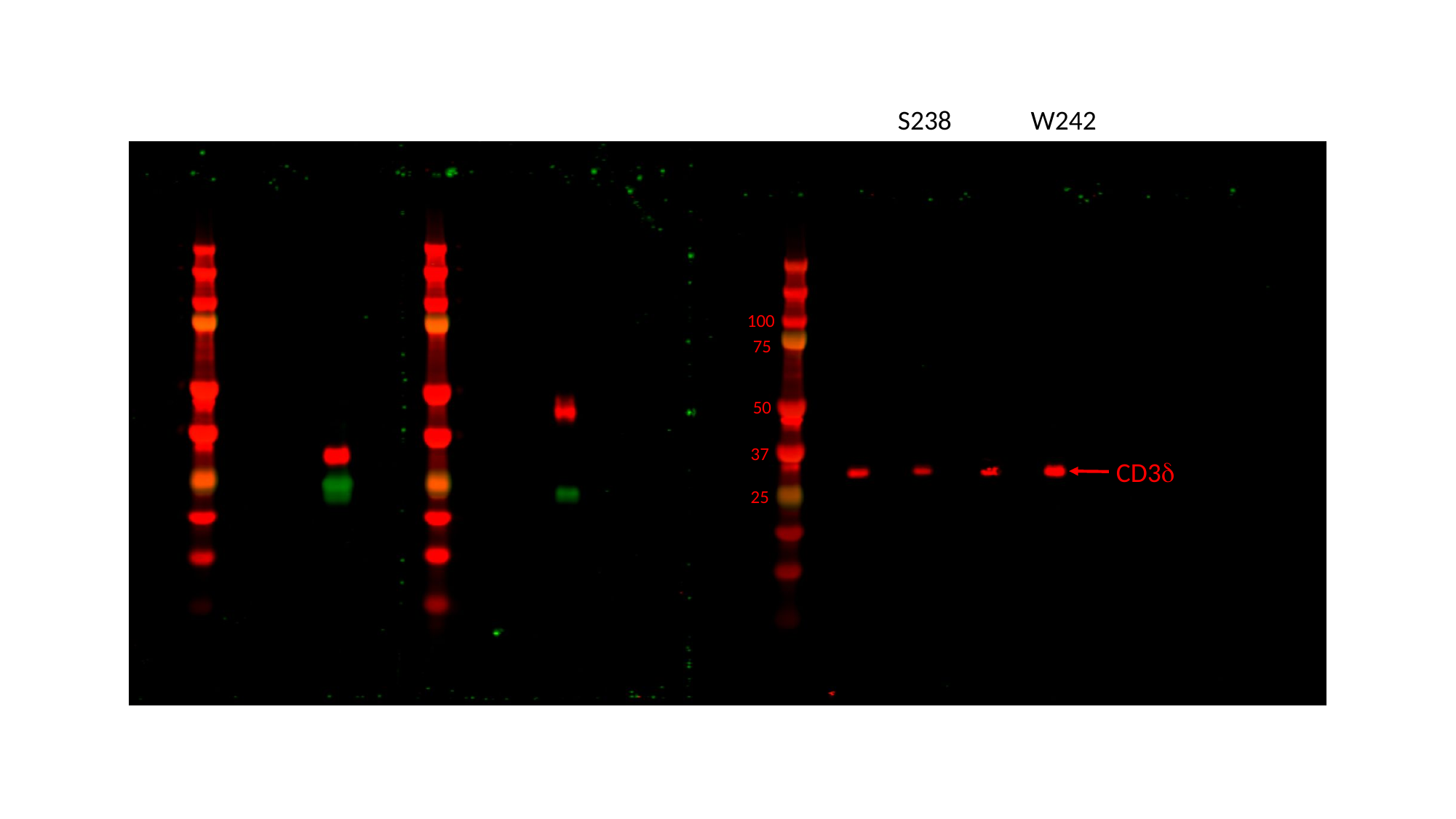

S238
W242
100
75
50
37
CD3d
25

Supplement: Supplementary file 7 — Source data Fig. 2 [file 44319_2024_314_MOESM7_ESM.zip › Fig2_WB/Figure 2F-Gstrand/S238W242-FLAG-full-labeled.pptx]

## Slide 1
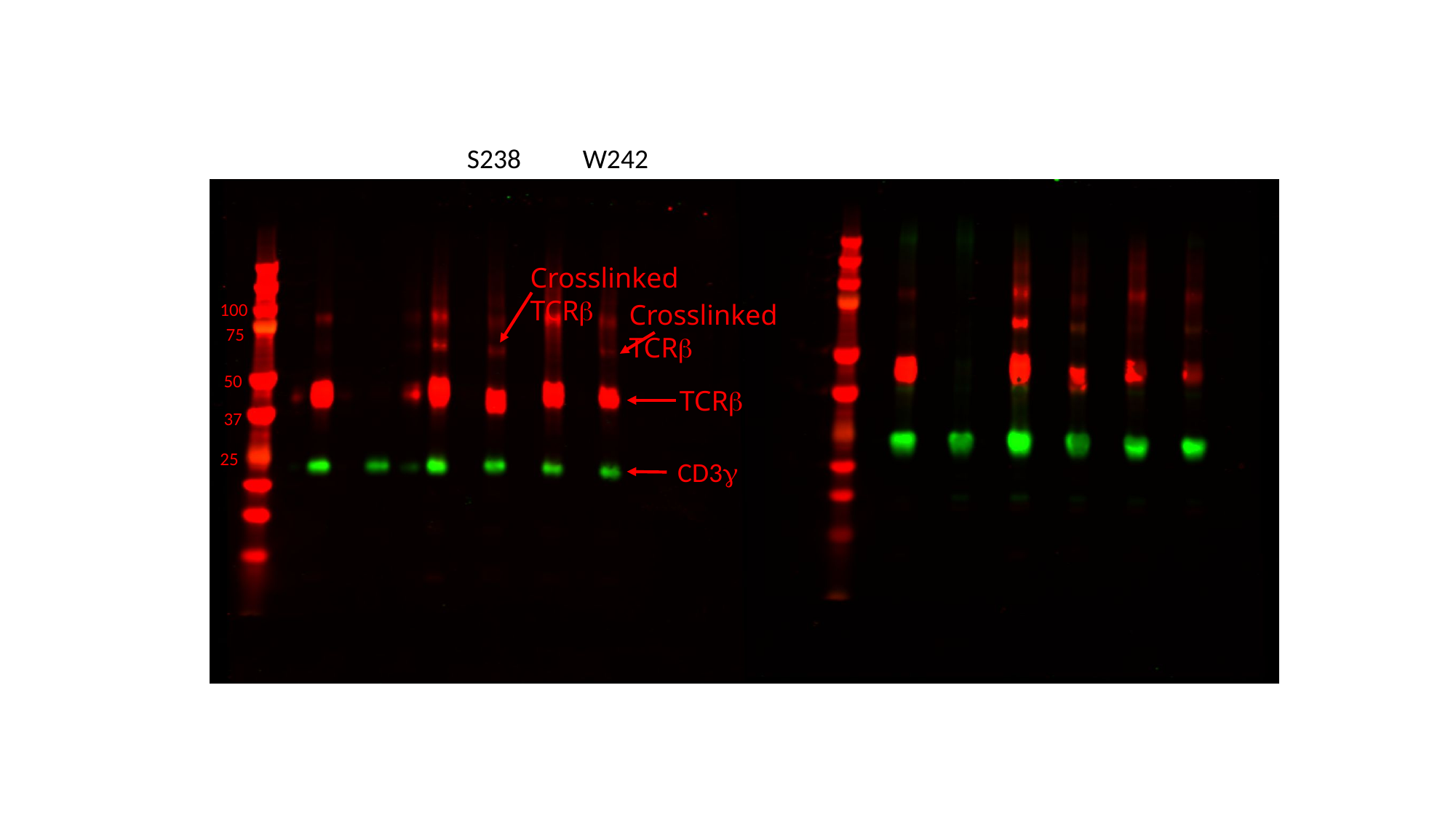

S238
W242
Crosslinked TCRb
100
Crosslinked TCRb
75
50
TCRb
37
25
CD3g

Supplement: Supplementary file 7 — Source data Fig. 2 [file 44319_2024_314_MOESM7_ESM.zip › Fig2_WB/Figure 2F-Gstrand/S238W242-VSVG-full-labeled.pptx]

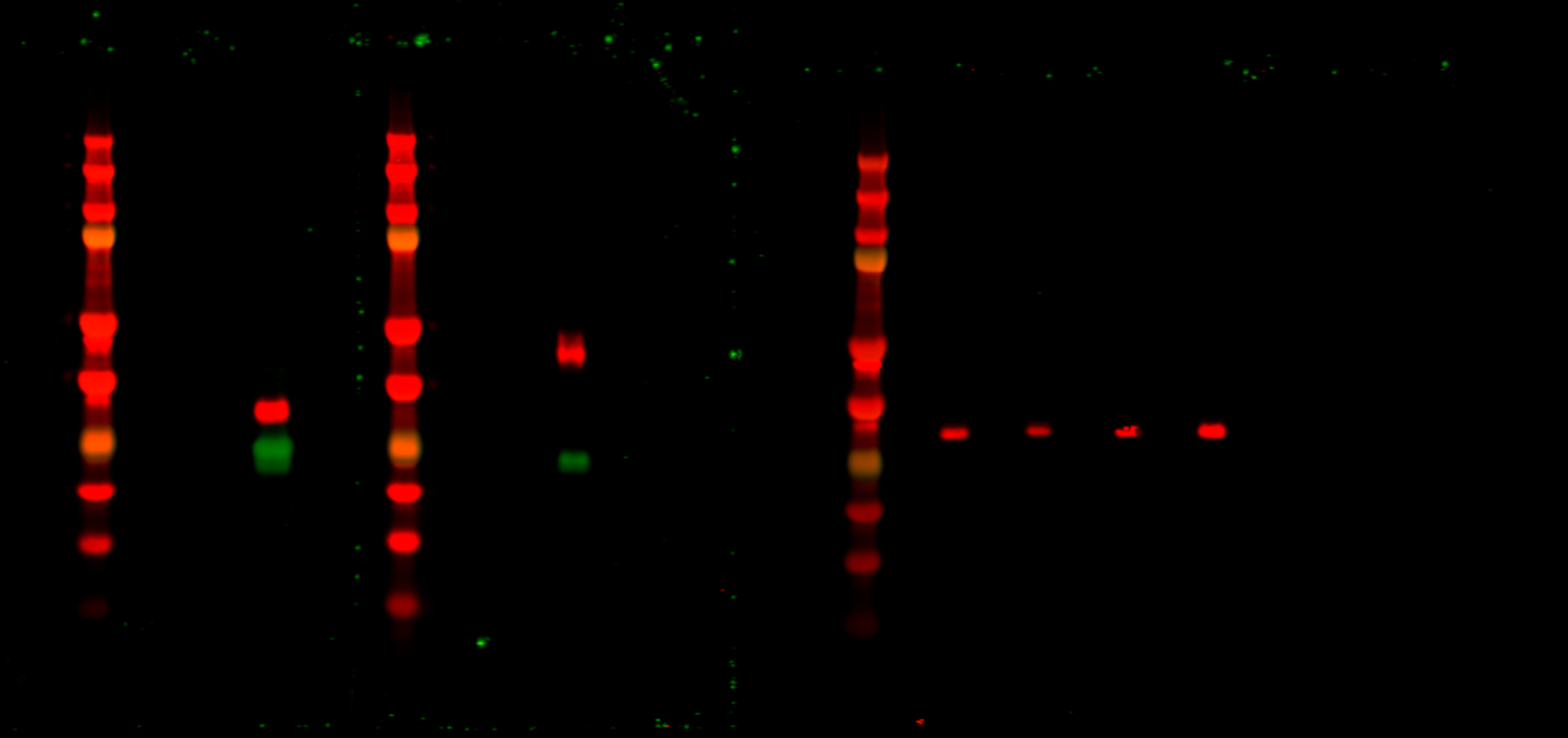

Supplement: Supplementary file 7 — Source data Fig. 2 [file 44319_2024_314_MOESM7_ESM.zip › Fig2_WB/Figure 2F-Gstrand/S238W242-FLAG-full.png]

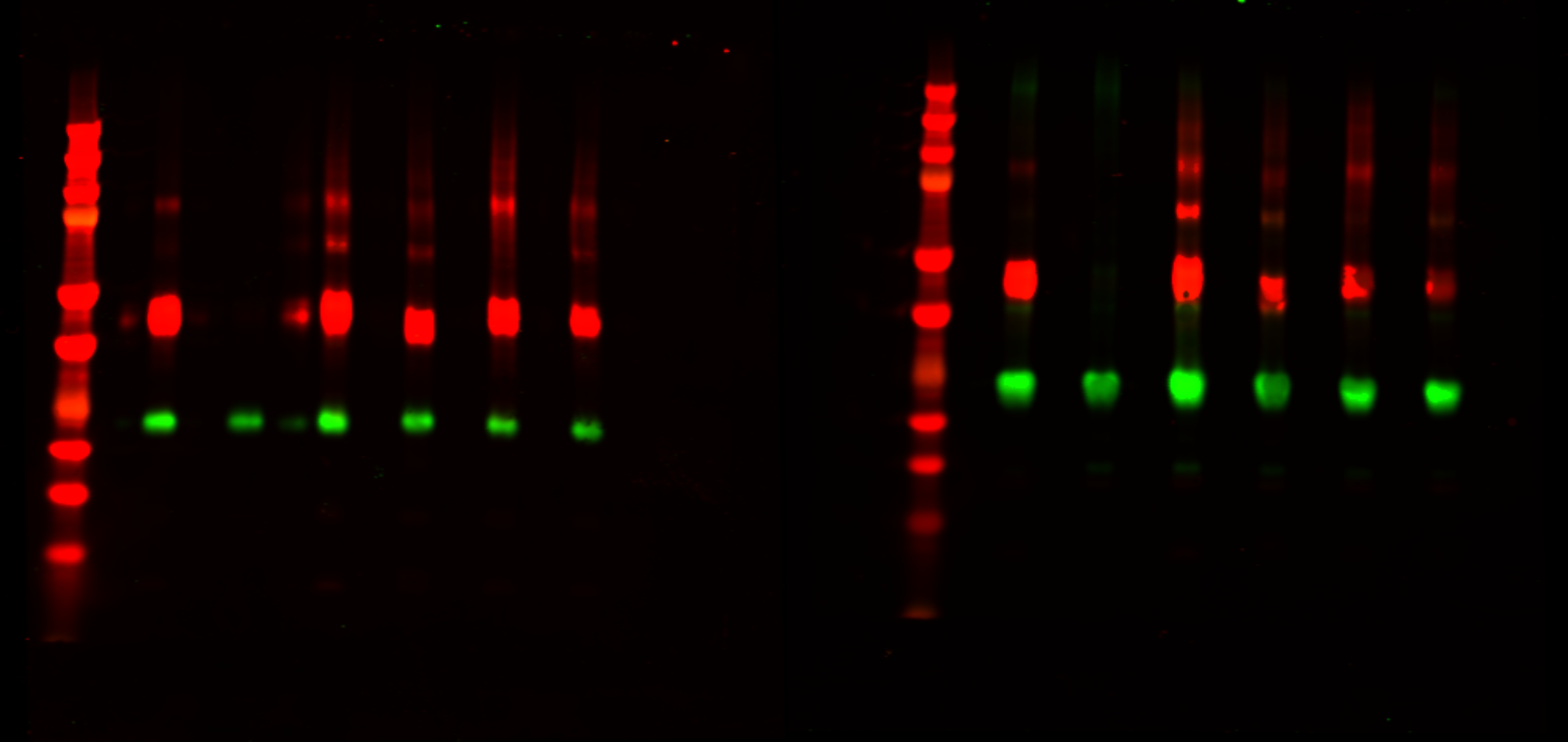

Supplement: Supplementary file 7 — Source data Fig. 2 [file 44319_2024_314_MOESM7_ESM.zip › Fig2_WB/Figure 2F-Gstrand/S238W242-VSVG-full.png]

S238

W242

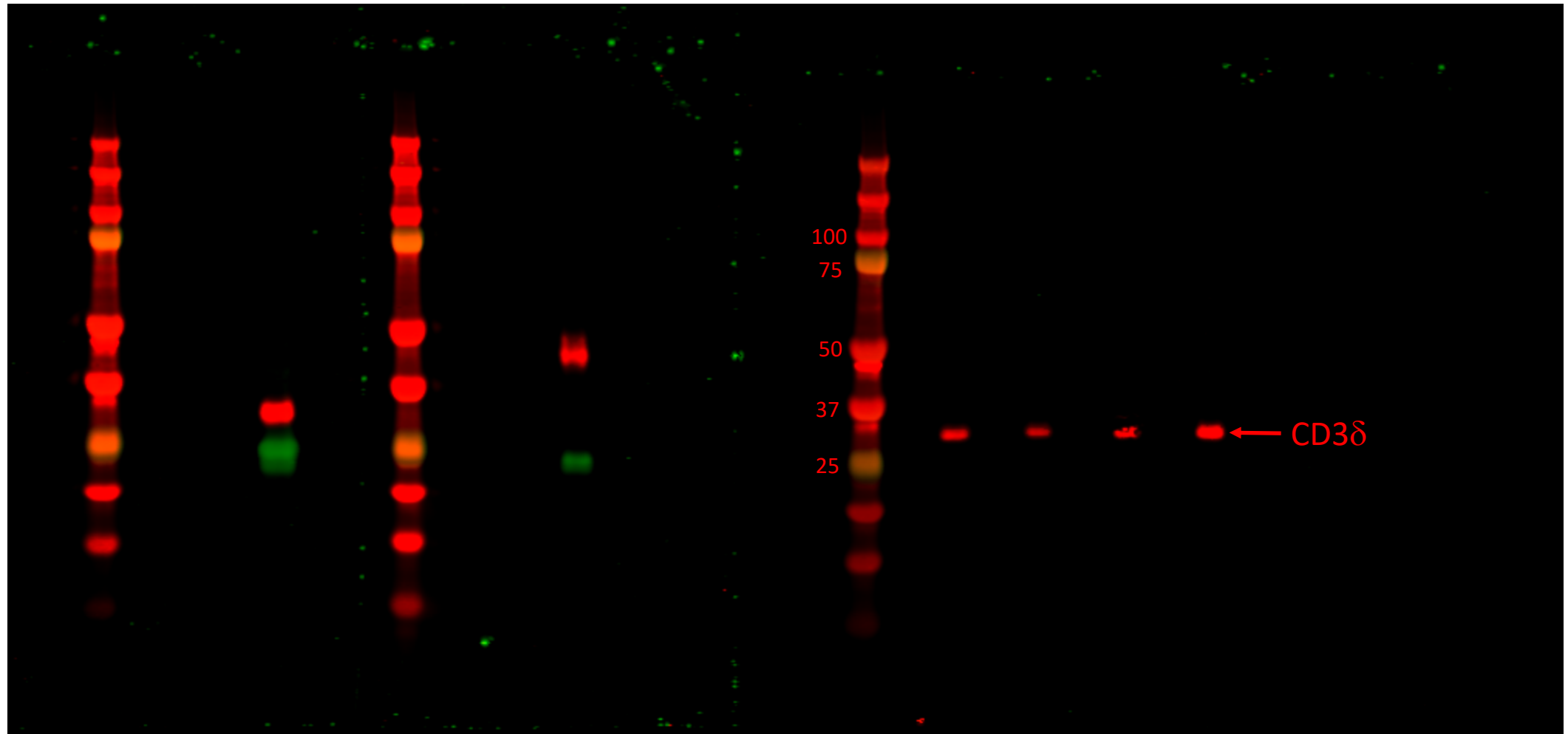

Supplement: Supplementary file 7 — Source data Fig. 2 [file 44319_2024_314_MOESM7_ESM.zip › Fig2_WB/Figure 2F-Gstrand/S238W242-FLAG-full-labeled.pdf]

A172 D174

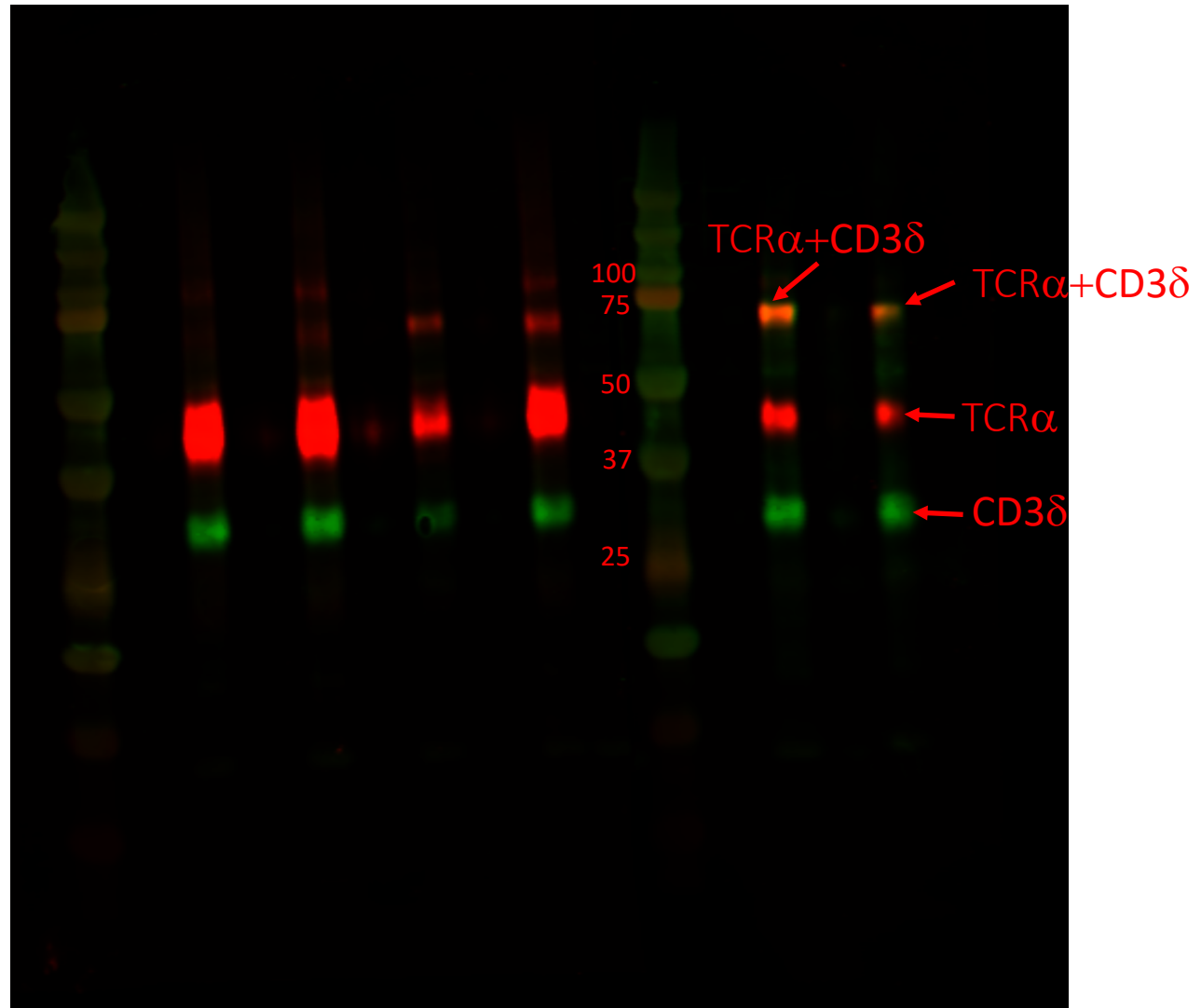

Supplement: Supplementary file 7 — Source data Fig. 2 [file 44319_2024_314_MOESM7_ESM.zip › Fig2_WB/Figure 2B-DEloop/Figure2B-full-labeled.pdf]

A172

D174

A172

D174

A172

D174

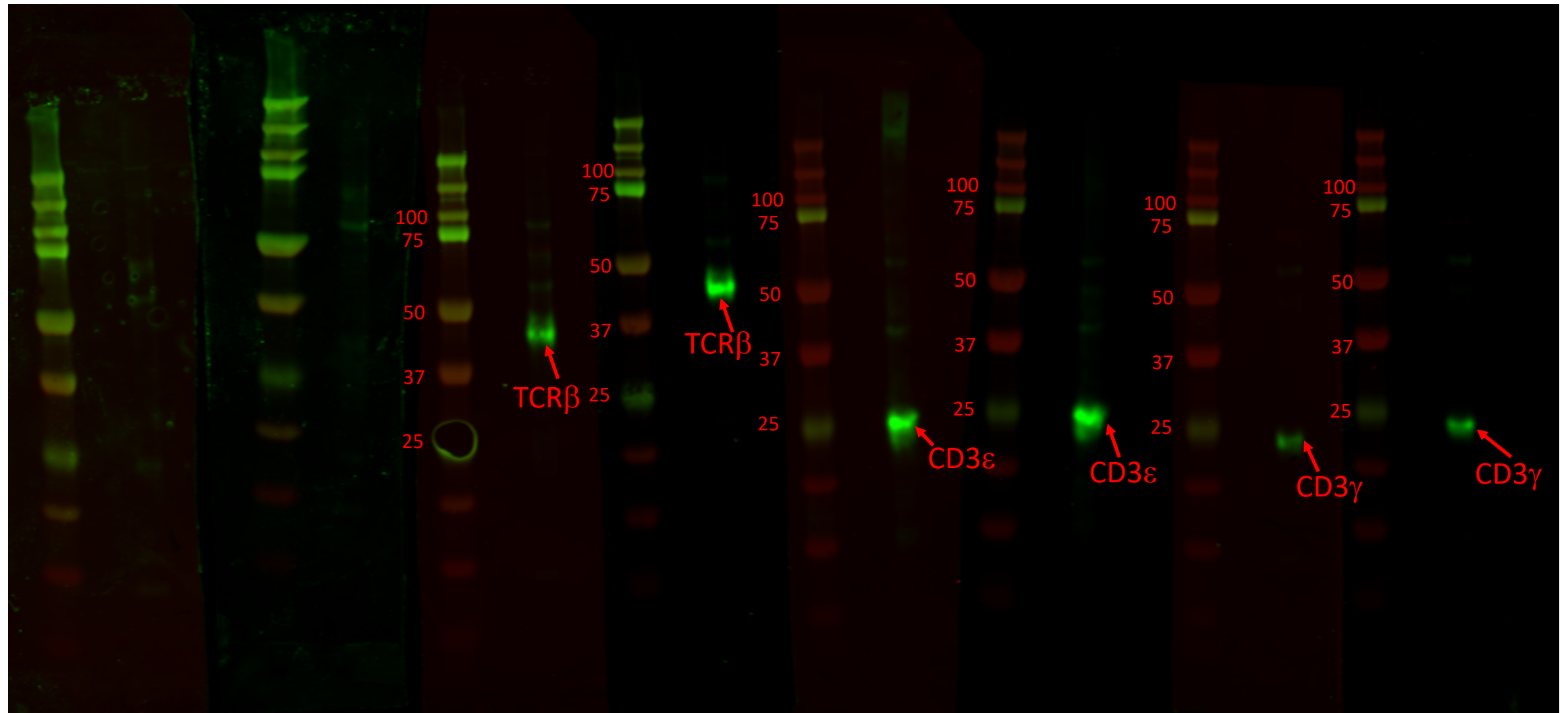

Supplement: Supplementary file 7 — Source data Fig. 2 [file 44319_2024_314_MOESM7_ESM.zip › Fig2_WB/Figure 2B-DEloop/Figure2-figure2B-1-full-labeled.pdf]

## Slide 1
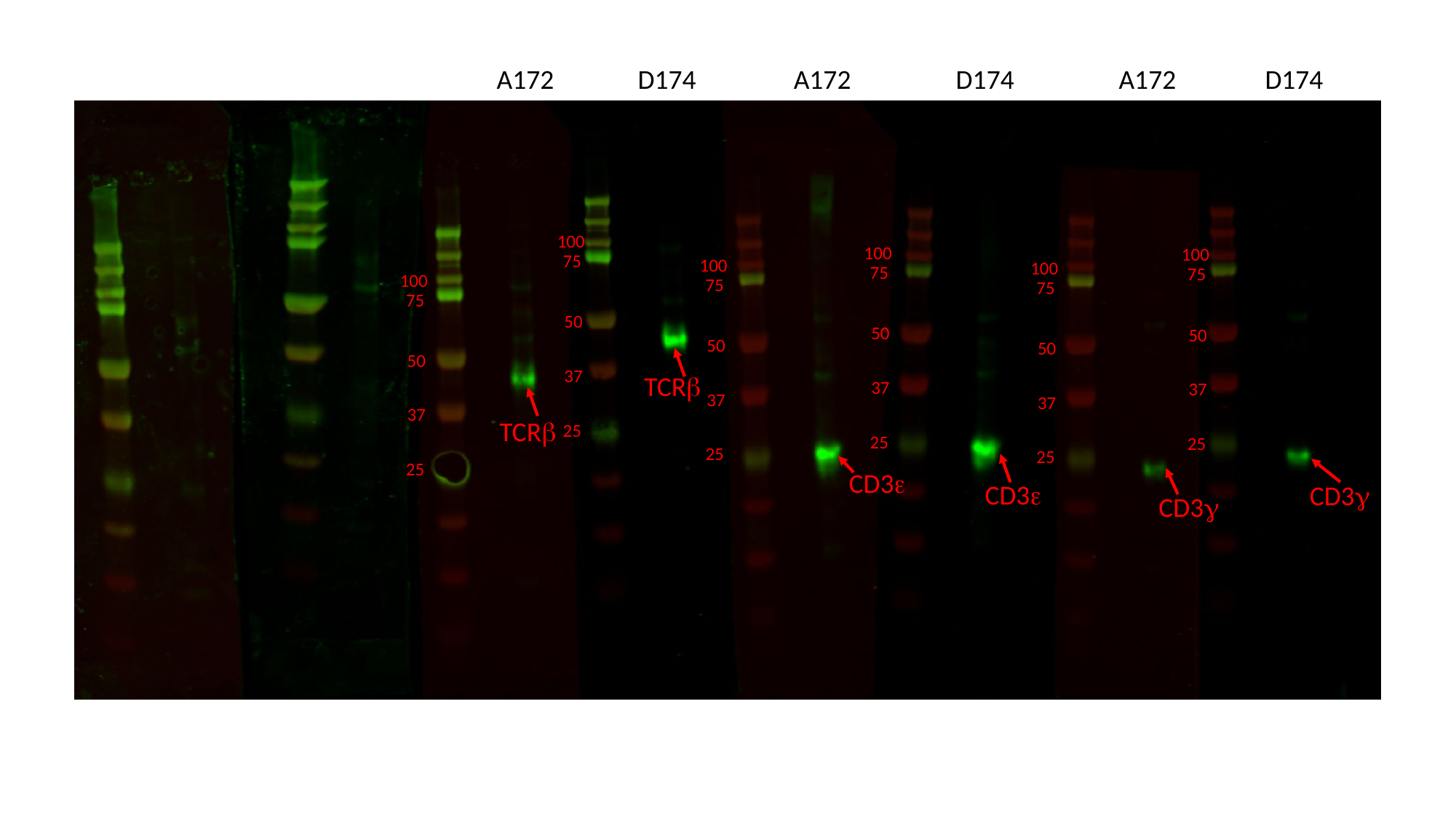

A172
D174
A172
D174
A172
D174
100
100
100
75
100
100
75
75
100
75
75
75
50
50
50
50
50
50
37
TCRb
37
37
37
37
37
TCRb
25
25
25
25
25
25
CD3e
CD3e
CD3g
CD3g

Supplement: Supplementary file 7 — Source data Fig. 2 [file 44319_2024_314_MOESM7_ESM.zip › Fig2_WB/Figure 2B-DEloop/Figure2-figure2B-1-full.pptx]

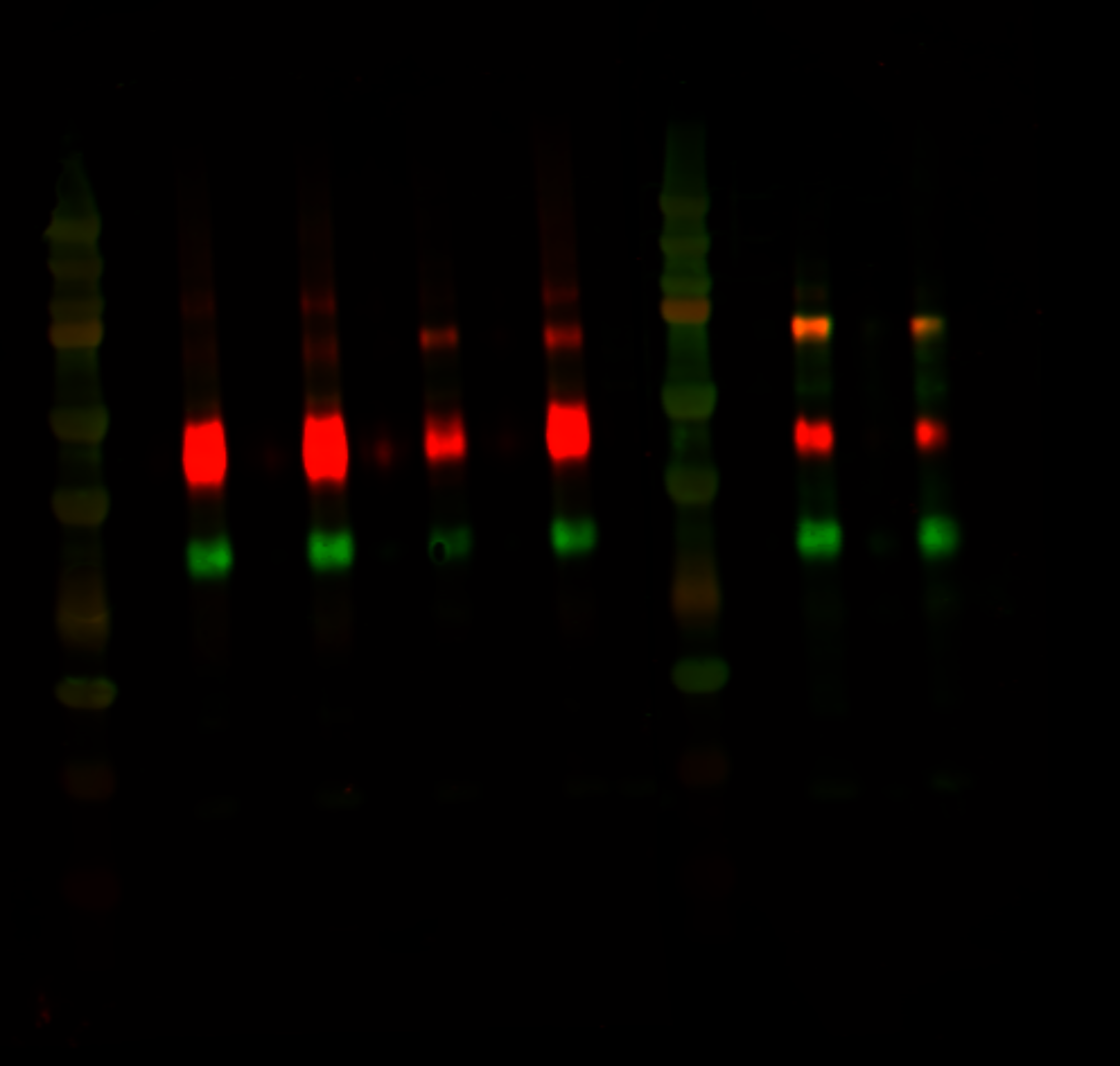

Supplement: Supplementary file 7 — Source data Fig. 2 [file 44319_2024_314_MOESM7_ESM.zip › Fig2_WB/Figure 2B-DEloop/Figure2B-full.png]

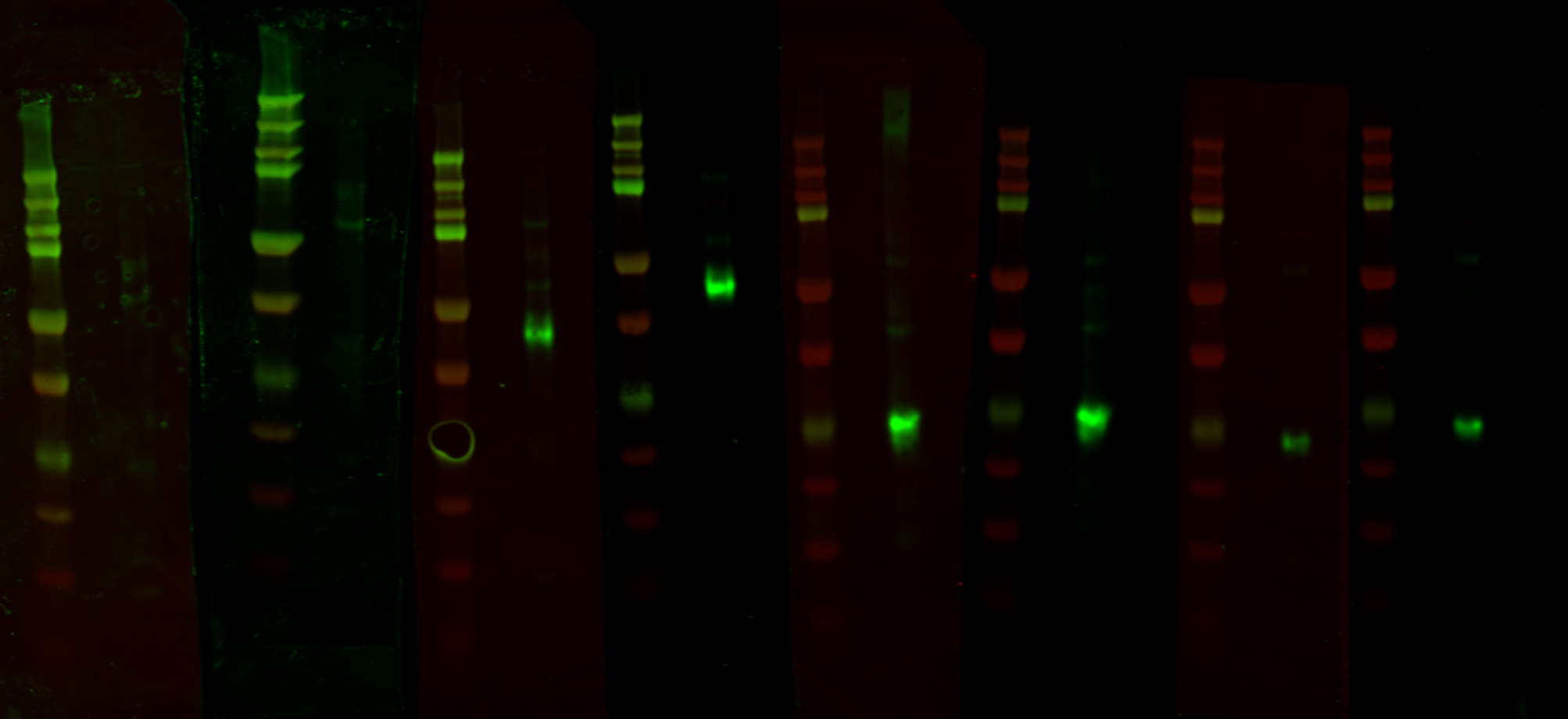

Supplement: Supplementary file 7 — Source data Fig. 2 [file 44319_2024_314_MOESM7_ESM.zip › Fig2_WB/Figure 2B-DEloop/Figure2-figure2B-1-full.png]

## Slide 1
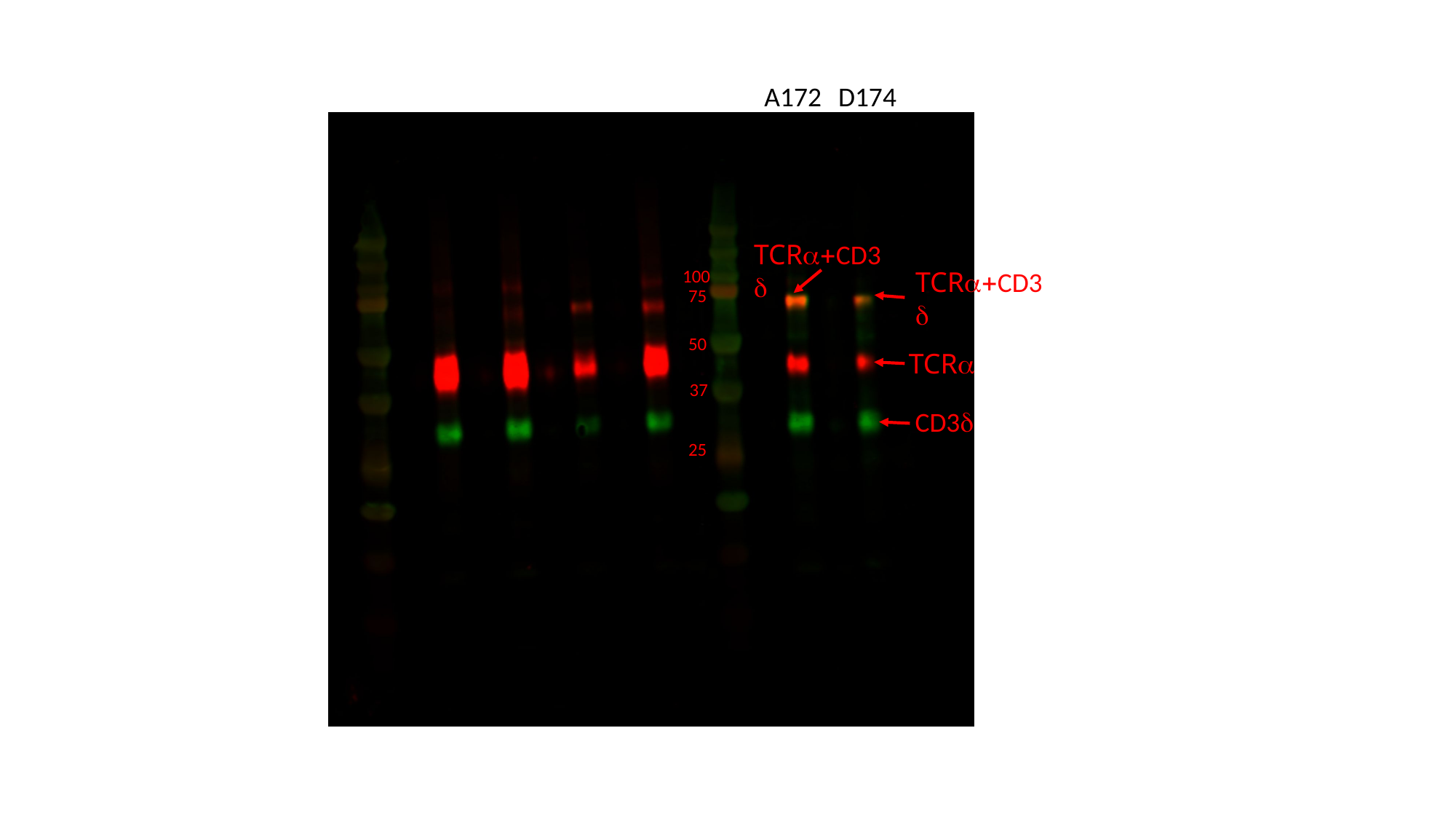

A172
D174
TCRa+CD3d
100
TCRa+CD3d
75
50
TCRa
37
CD3d
25

Supplement: Supplementary file 7 — Source data Fig. 2 [file 44319_2024_314_MOESM7_ESM.zip › Fig2_WB/Figure 2B-DEloop/Figure2B-full.pptx]

## Slide 1
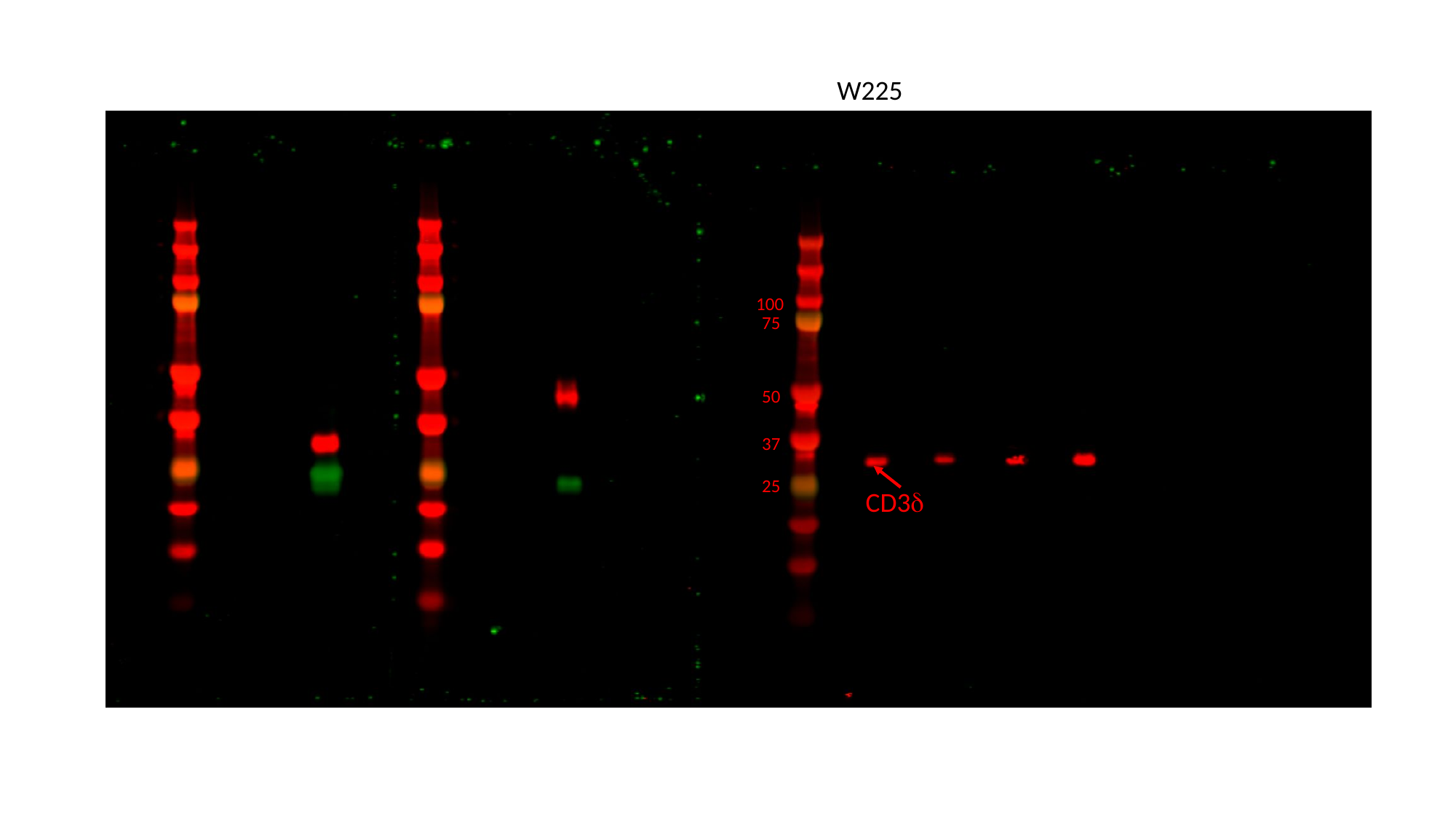

W225
100
75
50
37
25
CD3d

Supplement: Supplementary file 7 — Source data Fig. 2 [file 44319_2024_314_MOESM7_ESM.zip › Fig2_WB/Figure 2E-FGloop/W225-FLAG-full-labeled.pptx]

## Slide 1
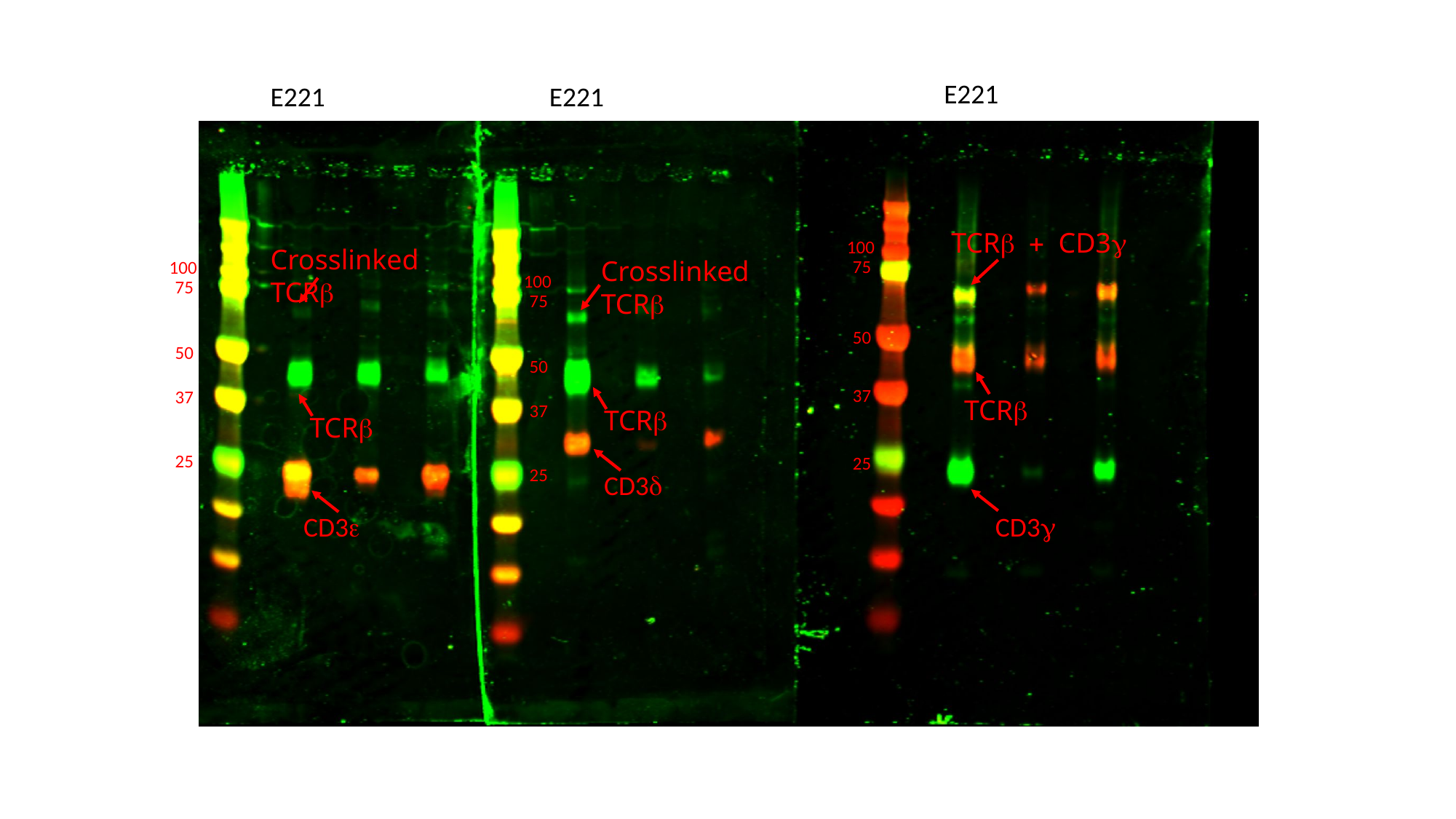

E221
E221
E221
TCRb + CD3g
100
Crosslinked TCRb
Crosslinked TCRb
75
100
100
75
75
50
50
50
37
37
TCRb
37
TCRb
TCRb
25
25
25
CD3d
CD3e
CD3g

Supplement: Supplementary file 7 — Source data Fig. 2 [file 44319_2024_314_MOESM7_ESM.zip › Fig2_WB/Figure 2E-FGloop/E221-full.pptx]

W225

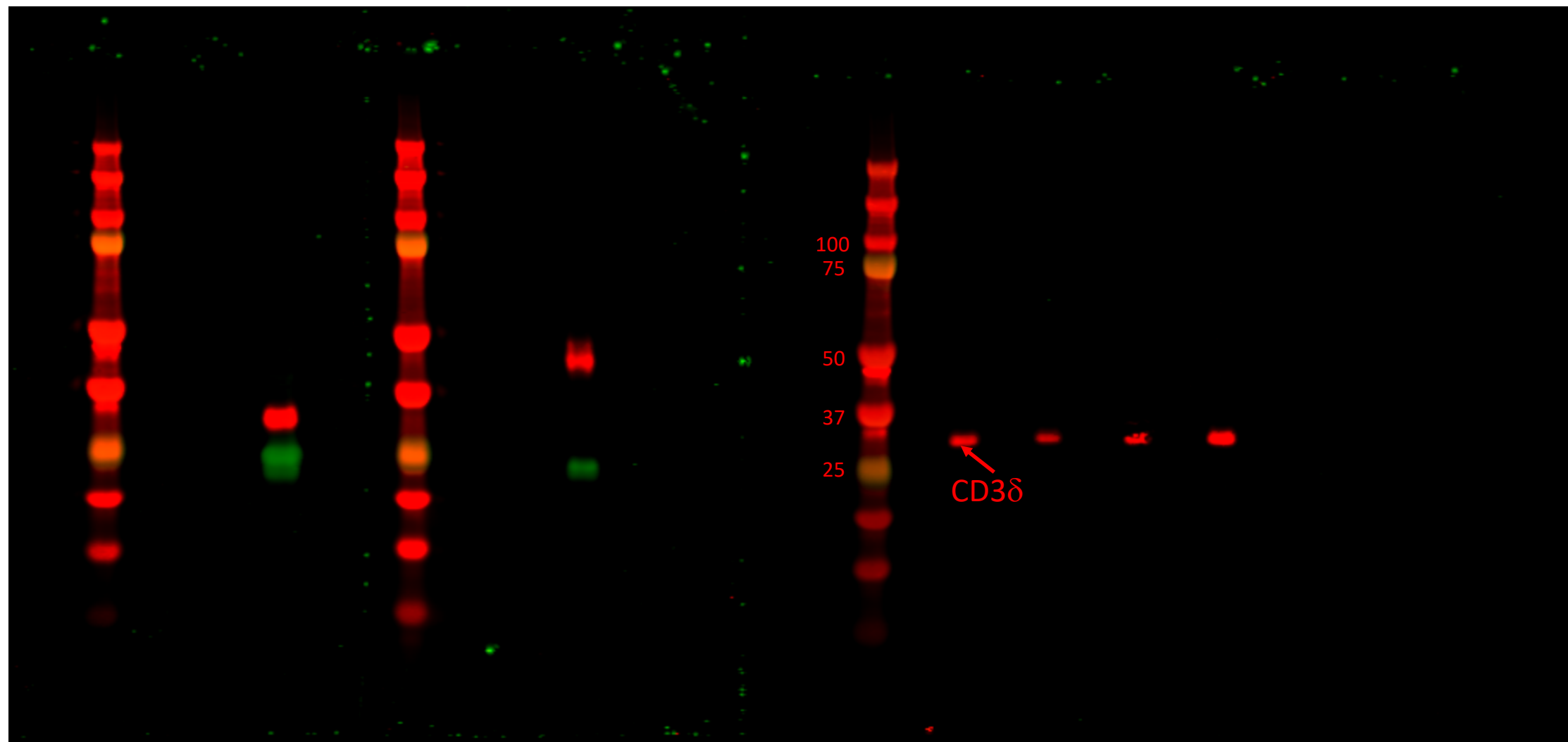

Supplement: Supplementary file 7 — Source data Fig. 2 [file 44319_2024_314_MOESM7_ESM.zip › Fig2_WB/Figure 2E-FGloop/W225-FLAG-full-labeled.pdf]

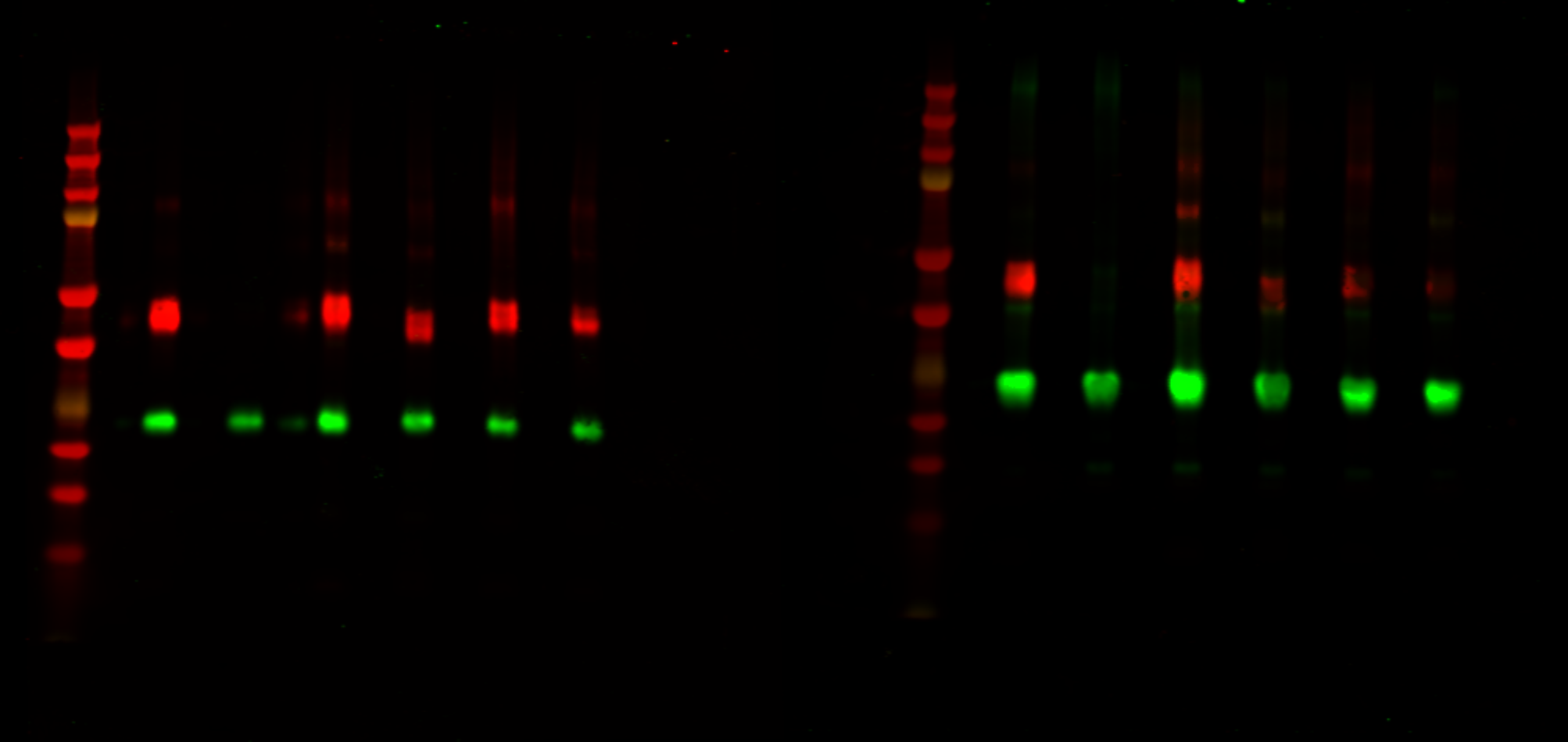

Supplement: Supplementary file 7 — Source data Fig. 2 [file 44319_2024_314_MOESM7_ESM.zip › Fig2_WB/Figure 2E-FGloop/W225-HA-full.png]

## Slide 1
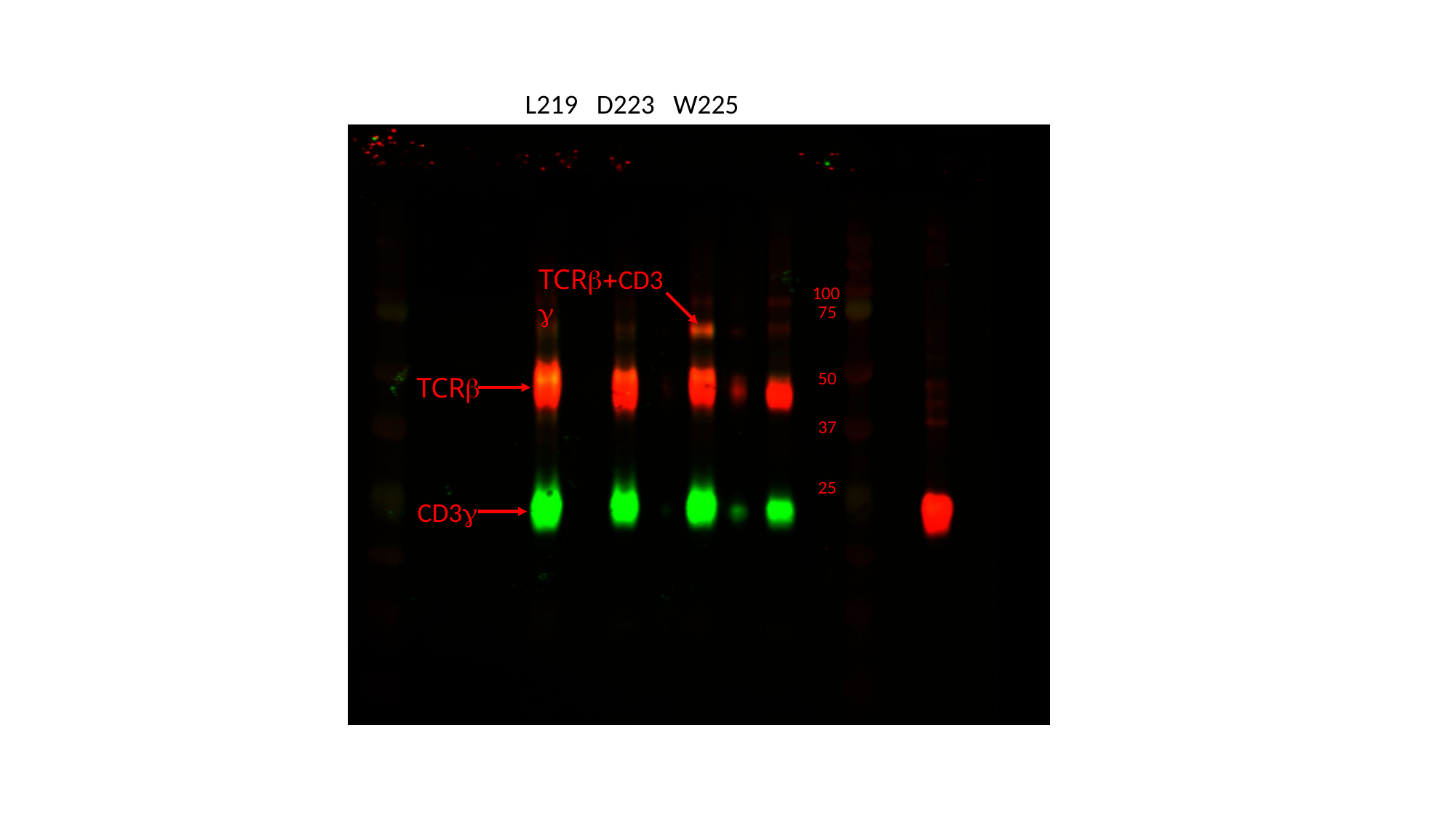

L219 D223 W225
TCRb+CD3g
100
75
50
TCRb
37
25
CD3g

Supplement: Supplementary file 7 — Source data Fig. 2 [file 44319_2024_314_MOESM7_ESM.zip › Fig2_WB/Figure 2E-FGloop/L219D223W225-full.pptx]

E221

E221

E221

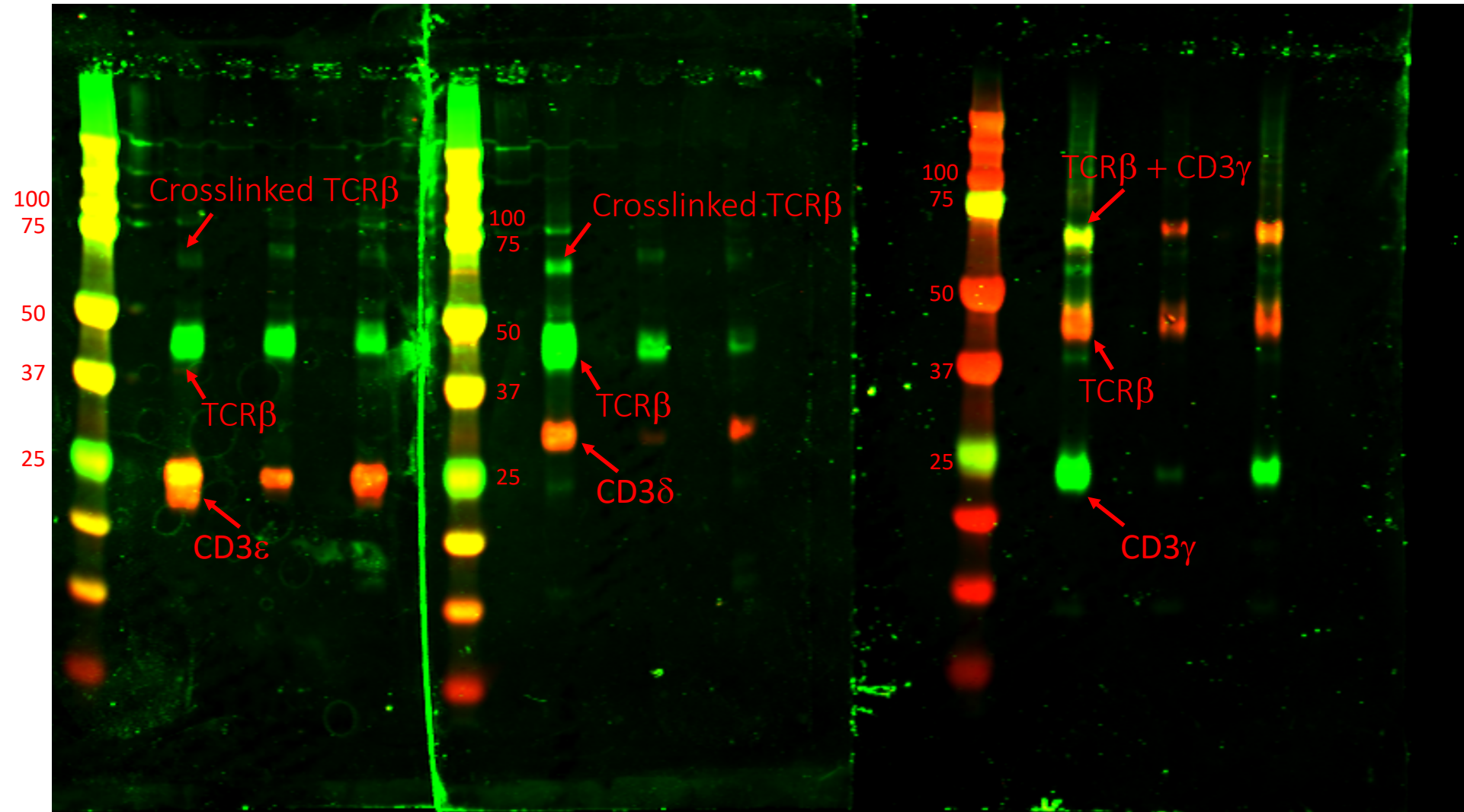

Supplement: Supplementary file 7 — Source data Fig. 2 [file 44319_2024_314_MOESM7_ESM.zip › Fig2_WB/Figure 2E-FGloop/E221-full-labeled.pdf]

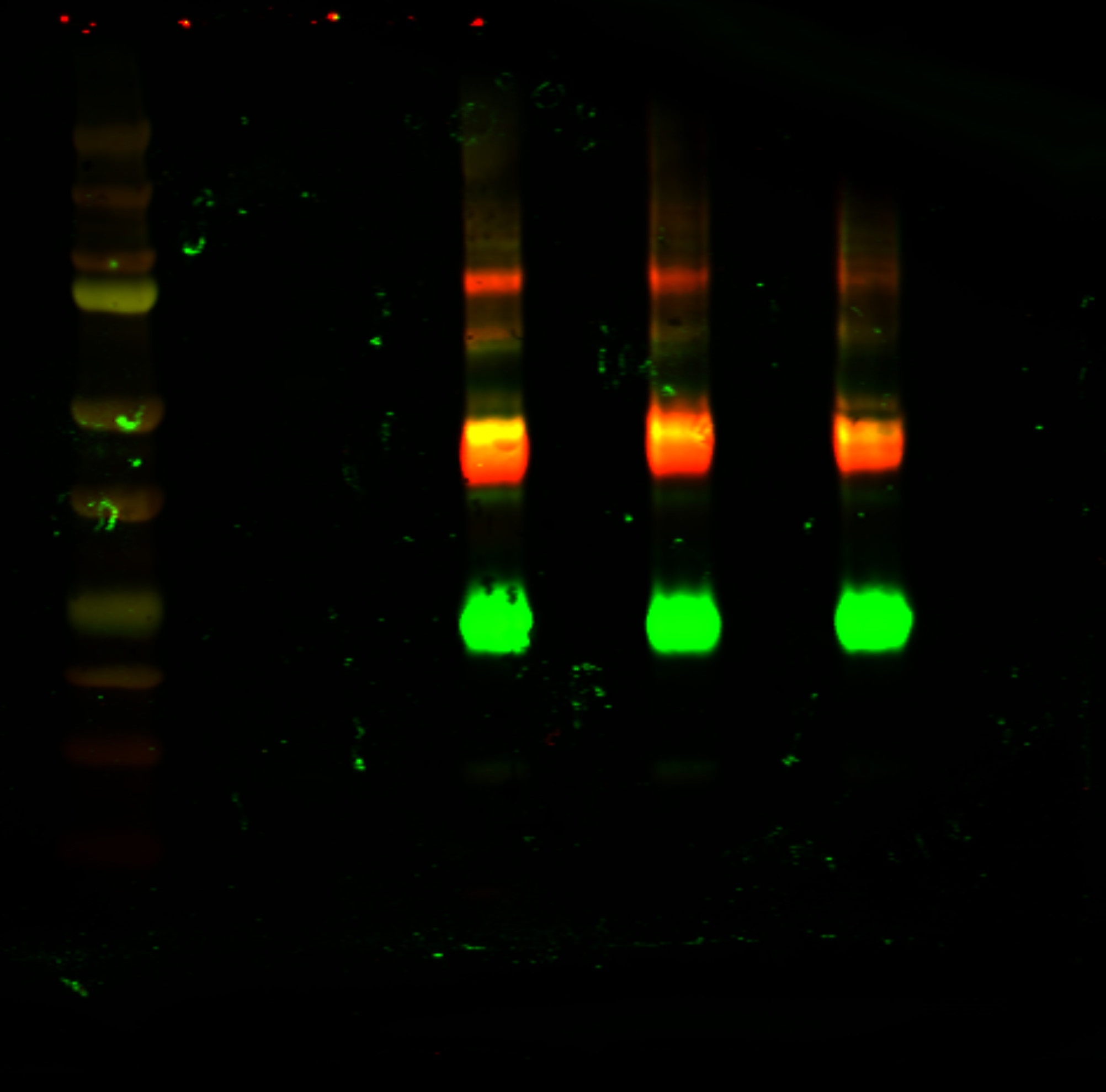

Supplement: Supplementary file 7 — Source data Fig. 2 [file 44319_2024_314_MOESM7_ESM.zip › Fig2_WB/Figure 2E-FGloop/S229K231-full.png]

W225

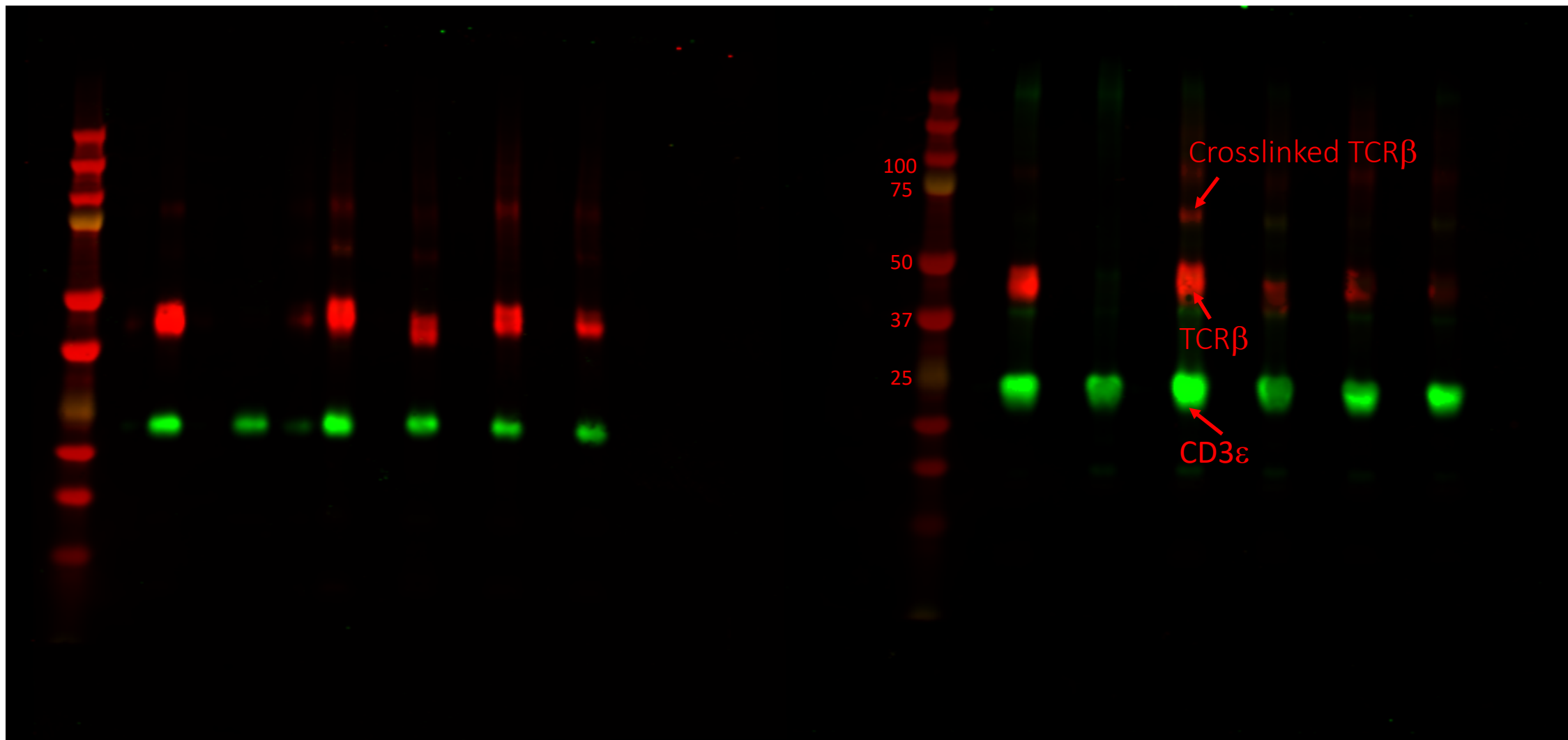

Supplement: Supplementary file 7 — Source data Fig. 2 [file 44319_2024_314_MOESM7_ESM.zip › Fig2_WB/Figure 2E-FGloop/W225-HA-full-labeled.pdf]

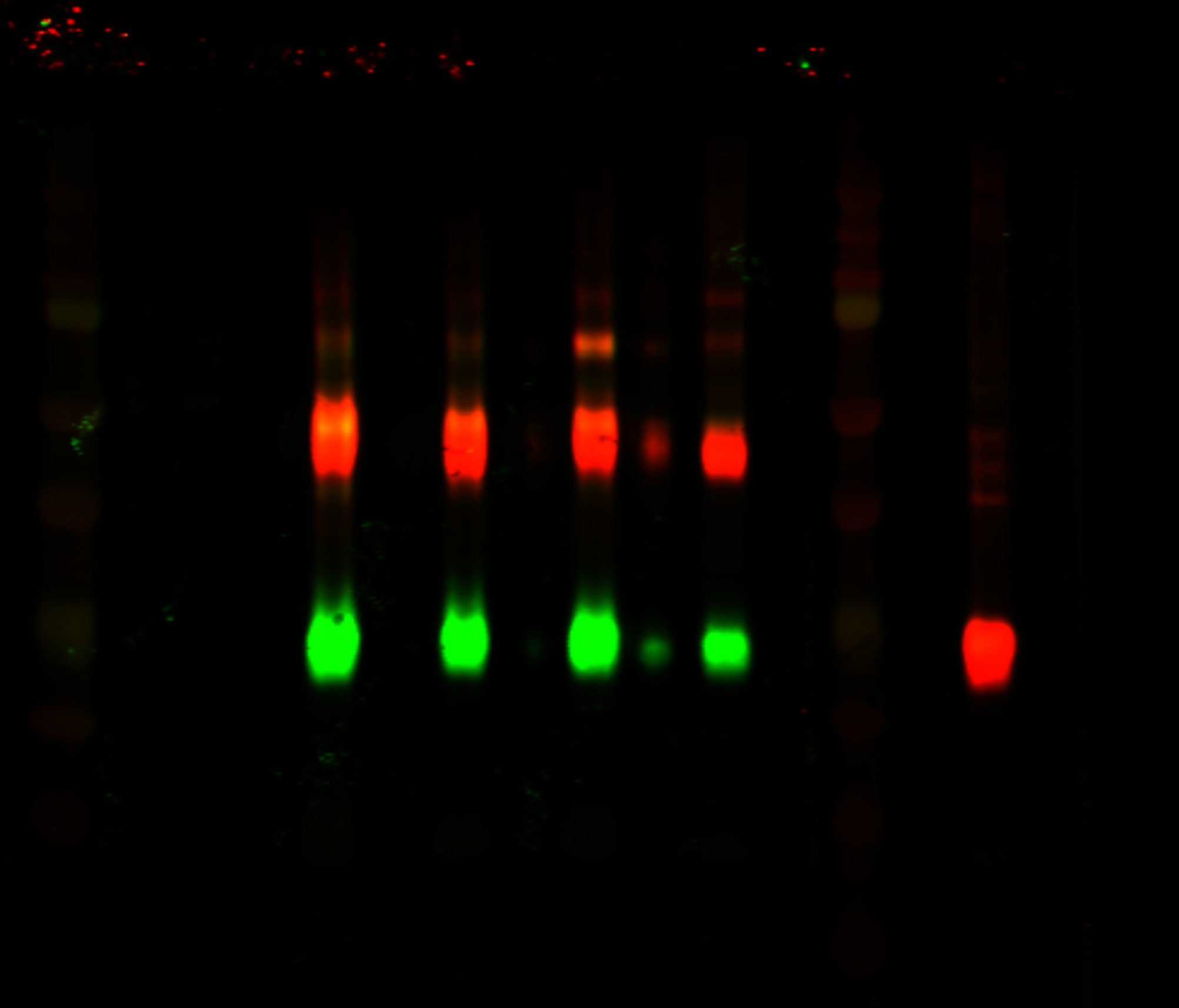

Supplement: Supplementary file 7 — Source data Fig. 2 [file 44319_2024_314_MOESM7_ESM.zip › Fig2_WB/Figure 2E-FGloop/L219D223W225-full.png]

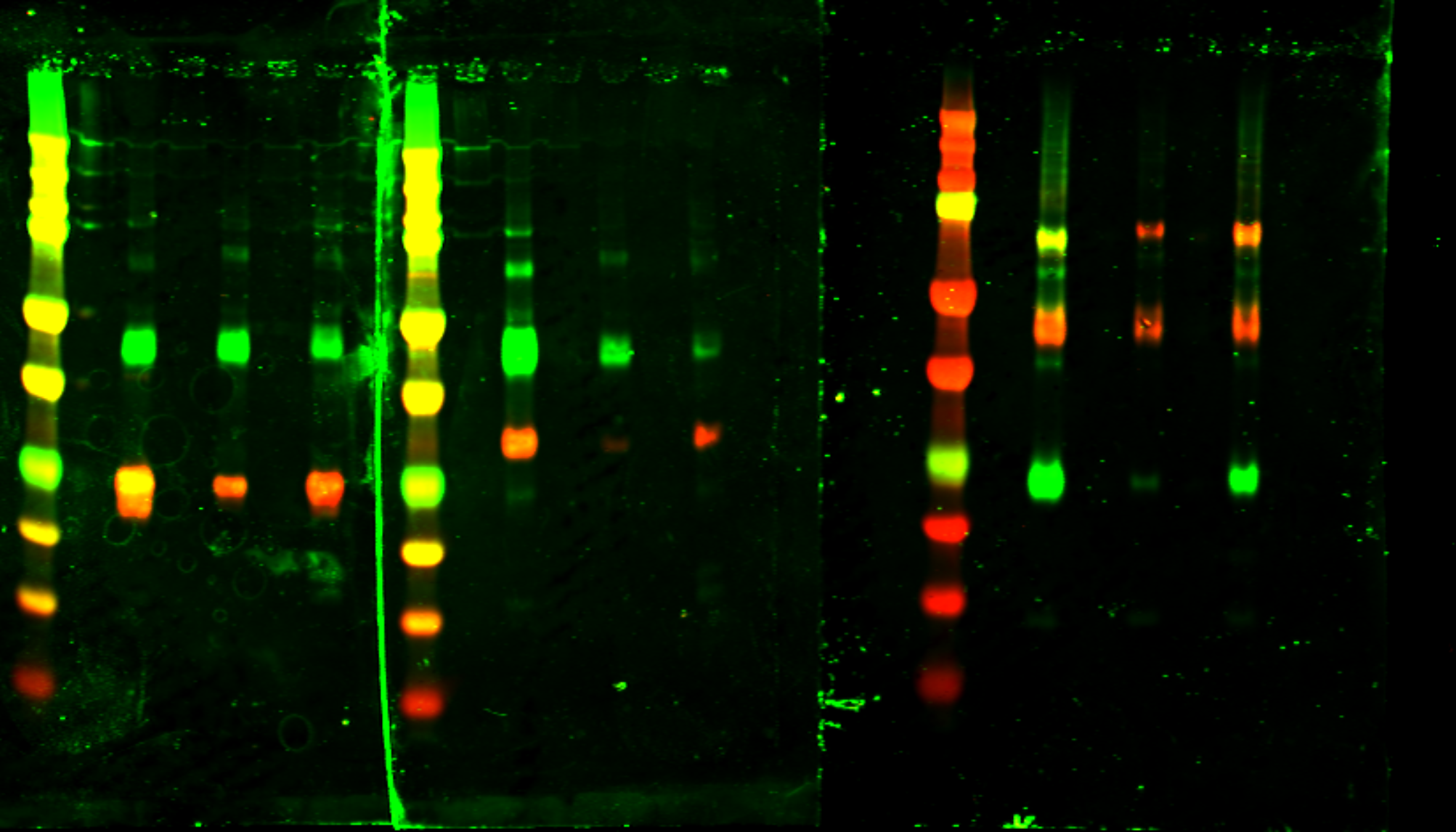

Supplement: Supplementary file 7 — Source data Fig. 2 [file 44319_2024_314_MOESM7_ESM.zip › Fig2_WB/Figure 2E-FGloop/E221-full.png]

## Slide 1
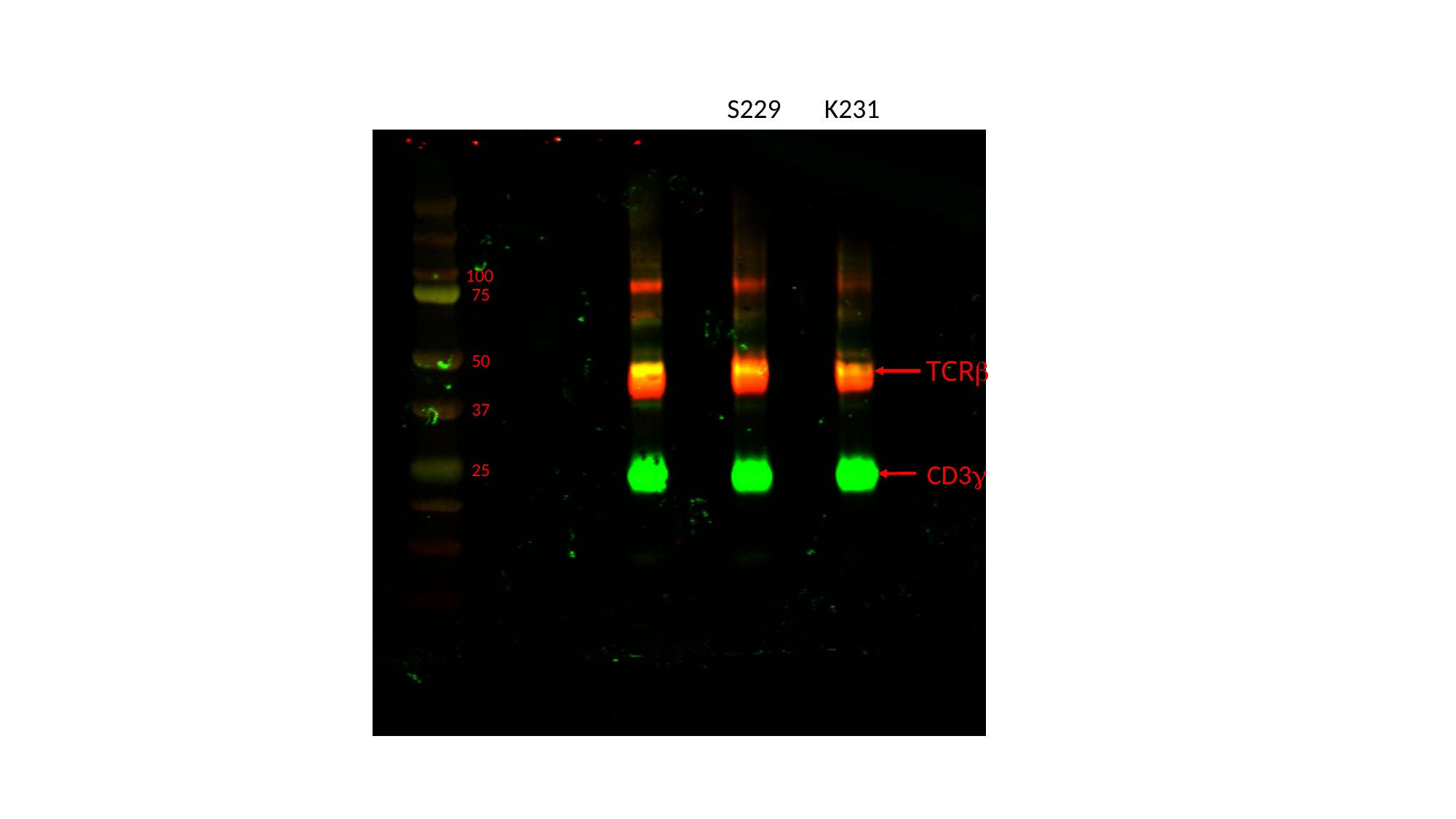

S229 K231
100
75
50
TCRb
37
CD3g
25

Supplement: Supplementary file 7 — Source data Fig. 2 [file 44319_2024_314_MOESM7_ESM.zip › Fig2_WB/Figure 2E-FGloop/S229K231-full.pptx]

S229

K231

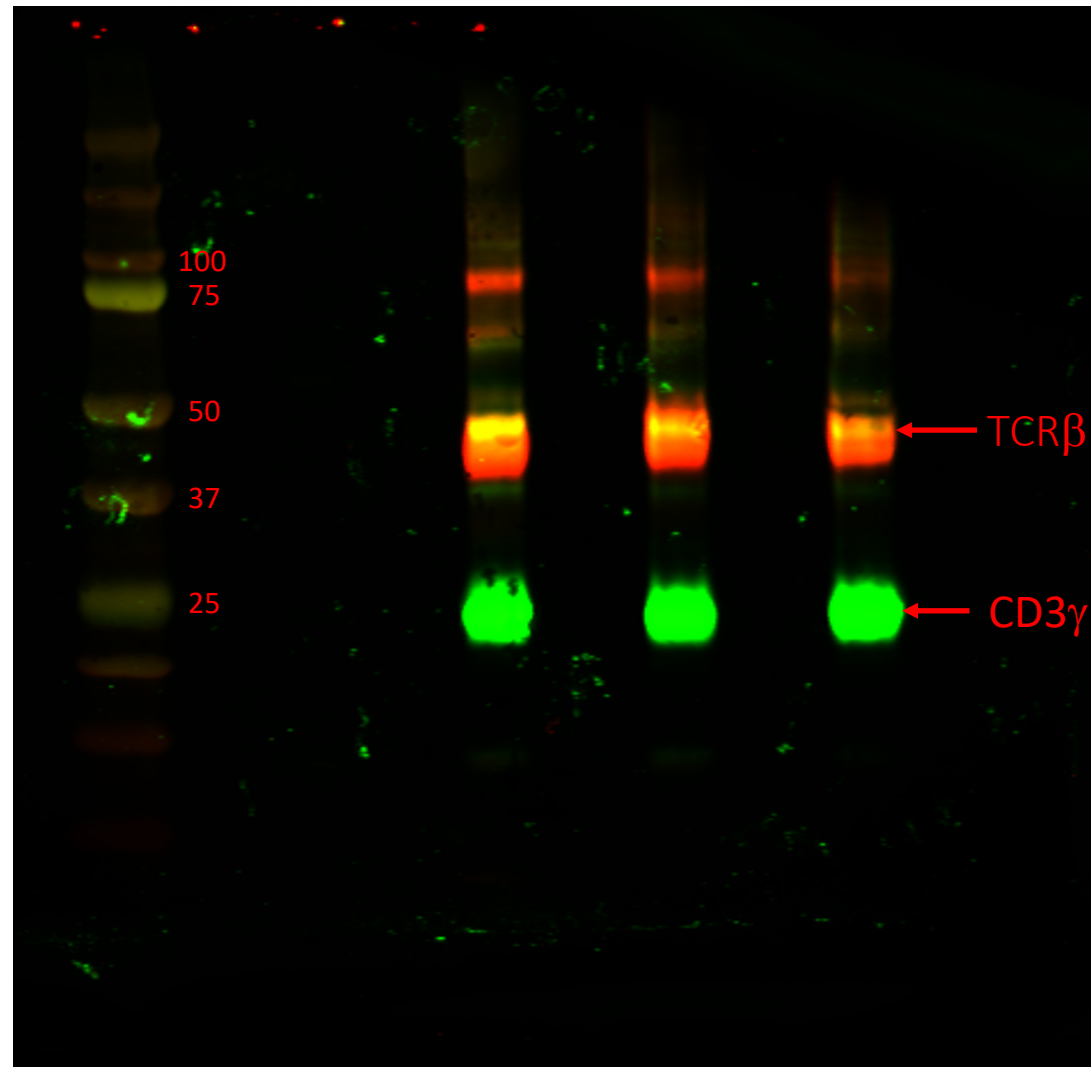

Supplement: Supplementary file 7 — Source data Fig. 2 [file 44319_2024_314_MOESM7_ESM.zip › Fig2_WB/Figure 2E-FGloop/S229K231-full-labeled.pdf]

## Slide 1
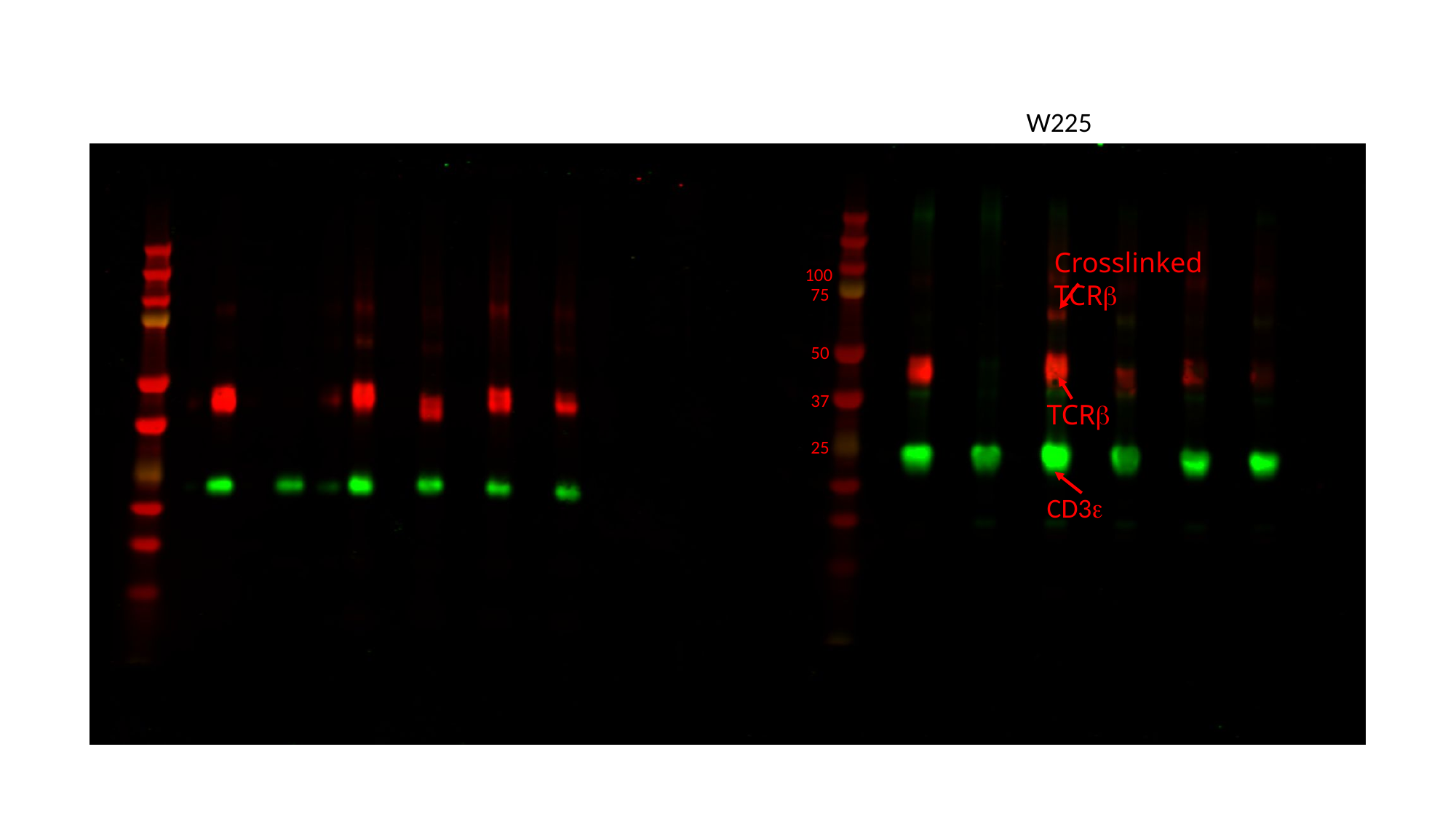

W225
Crosslinked TCRb
100
75
50
37
TCRb
25
CD3e

Supplement: Supplementary file 7 — Source data Fig. 2 [file 44319_2024_314_MOESM7_ESM.zip › Fig2_WB/Figure 2E-FGloop/W225-HA-full-labeled.pptx]

L219 D223 W225

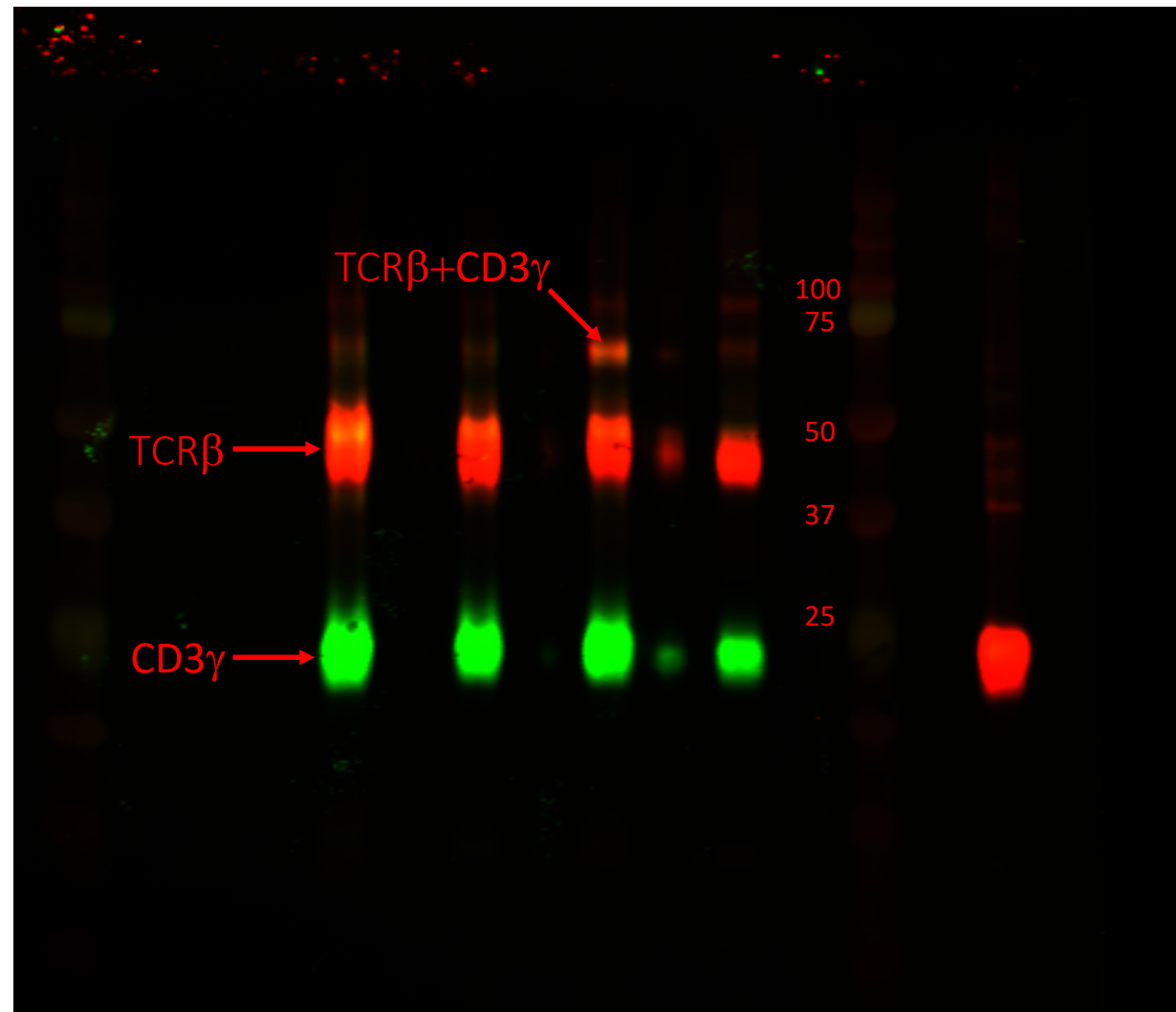

Supplement: Supplementary file 7 — Source data Fig. 2 [file 44319_2024_314_MOESM7_ESM.zip › Fig2_WB/Figure 2E-FGloop/L219D223W225-full-labeled.pdf]

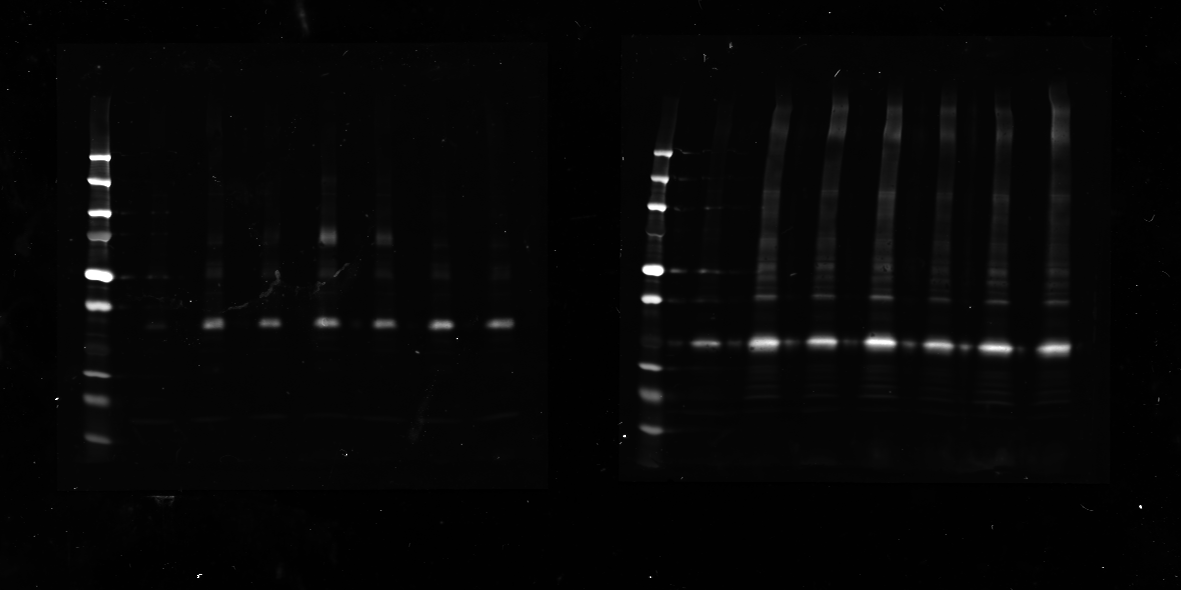

Supplement: Supplementary file 7 — Source data Fig. 2 [file 44319_2024_314_MOESM7_ESM.zip › Fig2_WB/Figure 2C-CCloop/Figure 2 - figure supplement 2 - rawdata/700.TIF]

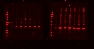

Supplement: Supplementary file 7 — Source data Fig. 2 [file 44319_2024_314_MOESM7_ESM.zip › Fig2_WB/Figure 2C-CCloop/Figure 2 - figure supplement 2 - rawdata/20130128 700In5 800 InL2_20130128 700In5 800 InL2_TH.jpg]

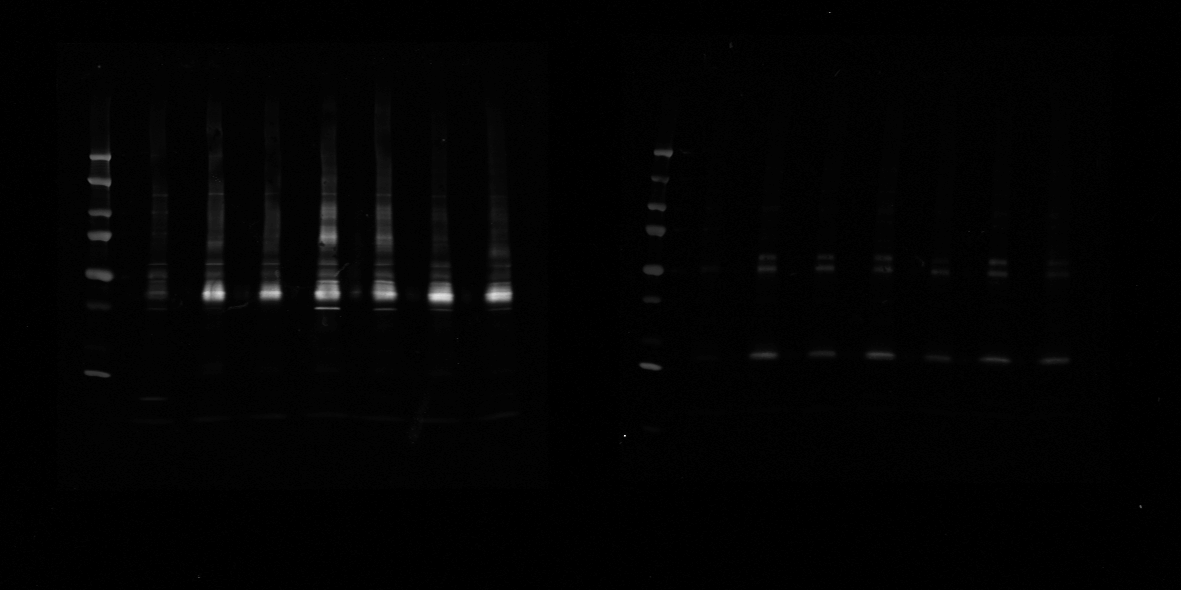

Supplement: Supplementary file 7 — Source data Fig. 2 [file 44319_2024_314_MOESM7_ESM.zip › Fig2_WB/Figure 2C-CCloop/Figure 2 - figure supplement 2 - rawdata/800.TIF]

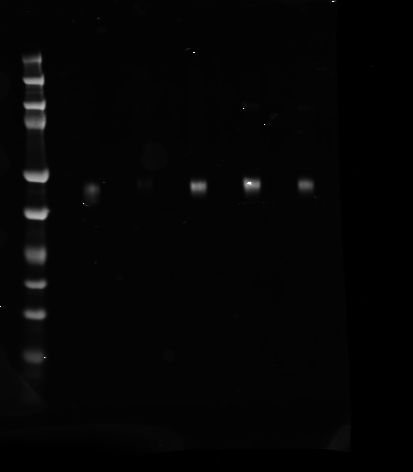

Supplement: Supplementary file 7 — Source data Fig. 2 [file 44319_2024_314_MOESM7_ESM.zip › Fig2_WB/Figure 2G-Helix3/E136A138K140K142-rawdata/700.TIF]

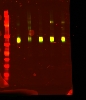

Supplement: Supplementary file 7 — Source data Fig. 2 [file 44319_2024_314_MOESM7_ESM.zip › Fig2_WB/Figure 2G-Helix3/E136A138K140K142-rawdata/2020-01-25-183409_E136A138K140K142-regel-mV5-rCmyc_TH.jpg]

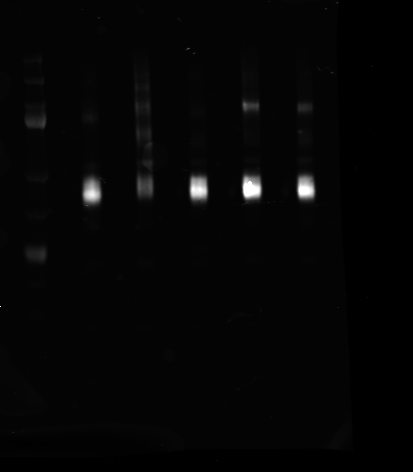

Supplement: Supplementary file 7 — Source data Fig. 2 [file 44319_2024_314_MOESM7_ESM.zip › Fig2_WB/Figure 2G-Helix3/E136A138K140K142-rawdata/800.TIF]

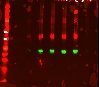

Supplement: Supplementary file 7 — Source data Fig. 2 [file 44319_2024_314_MOESM7_ESM.zip › Fig2_WB/Figure 2H-Helix4-Fstrand/H204P206N208F210-rawdata/2020-02-12-181326_new-H204P206N208F210-mHA-rV5-2_TH.jpg]

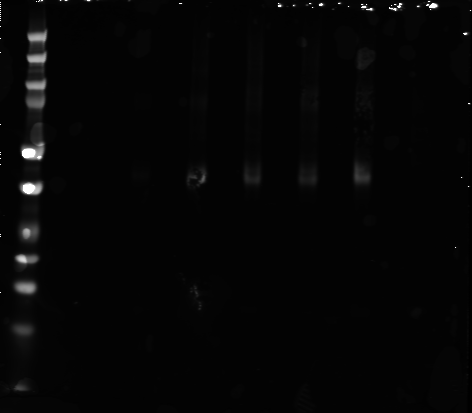

Supplement: Supplementary file 7 — Source data Fig. 2 [file 44319_2024_314_MOESM7_ESM.zip › Fig2_WB/Figure 2H-Helix4-Fstrand/H204P206N208F210-rawdata/700.TIF]

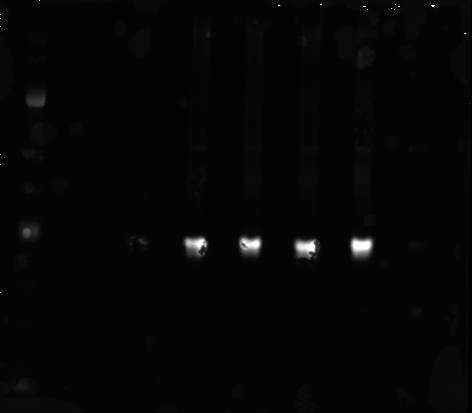

Supplement: Supplementary file 7 — Source data Fig. 2 [file 44319_2024_314_MOESM7_ESM.zip › Fig2_WB/Figure 2H-Helix4-Fstrand/H204P206N208F210-rawdata/800.TIF]

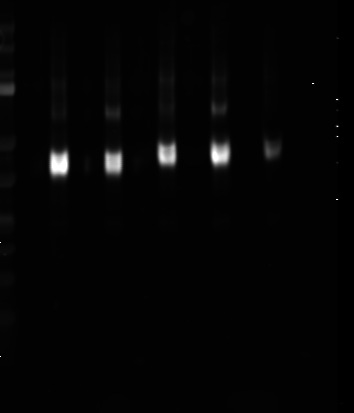

Supplement: Supplementary file 7 — Source data Fig. 2 [file 44319_2024_314_MOESM7_ESM.zip › Fig2_WB/Figure 2F-Gstrand/N236S238E240W242R244-rawdata/700.TIF]

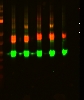

Supplement: Supplementary file 7 — Source data Fig. 2 [file 44319_2024_314_MOESM7_ESM.zip › Fig2_WB/Figure 2F-Gstrand/N236S238E240W242R244-rawdata/2020-03-18-174602_new-N236S238E240W242R244-rV5-mHA_TH.jpg]

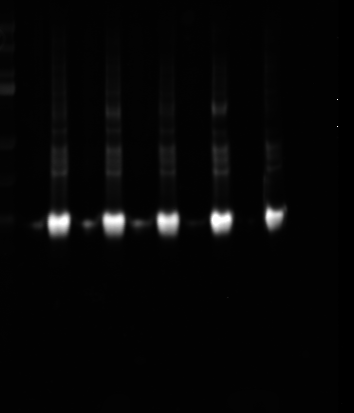

Supplement: Supplementary file 7 — Source data Fig. 2 [file 44319_2024_314_MOESM7_ESM.zip › Fig2_WB/Figure 2F-Gstrand/N236S238E240W242R244-rawdata/800.TIF]

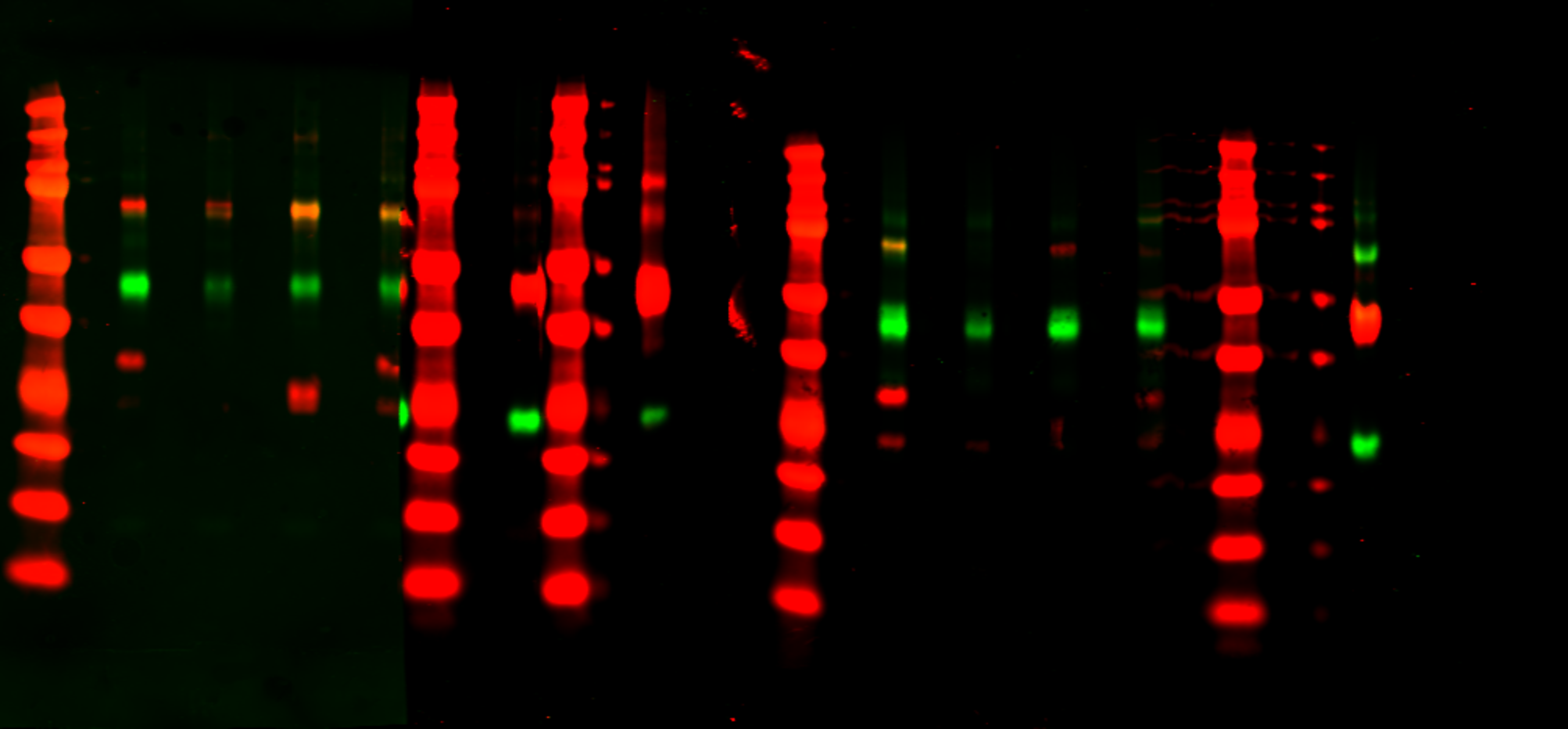

Supplement: Supplementary file 8 — Source data Fig. 3 [file 44319_2024_314_MOESM8_ESM.zip › Fig3_WB /Figure 3B/T5D9T35-full.png]

## Slide 1
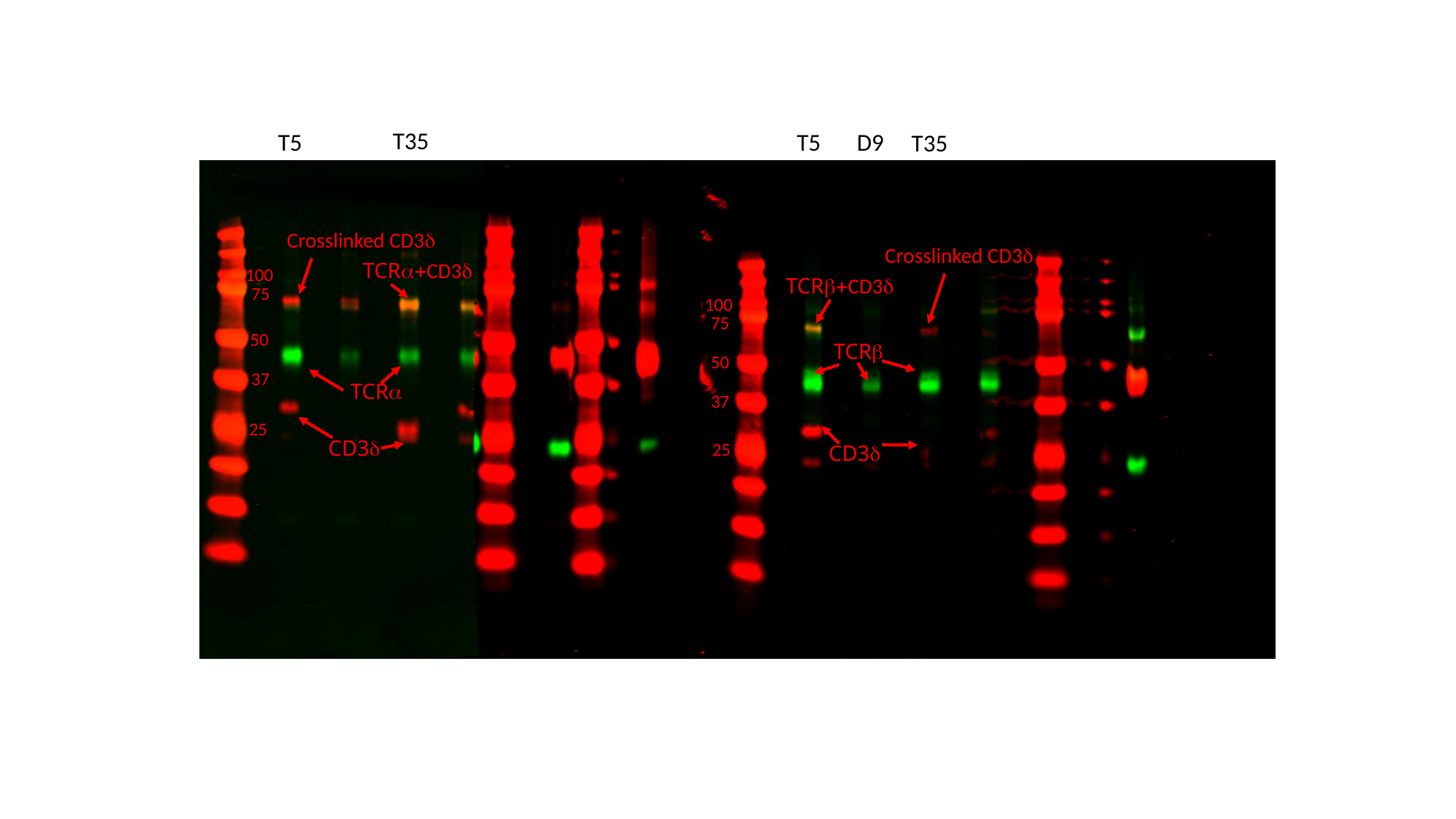

T35
T5
T5
D9
T35
Crosslinked CD3d
Crosslinked CD3d
TCRa+CD3d
100
TCRb+CD3d
75
100
75
50
TCRb
50
37
TCRa
37
25
CD3d
25
CD3d

Supplement: Supplementary file 8 — Source data Fig. 3 [file 44319_2024_314_MOESM8_ESM.zip › Fig3_WB /Figure 3B/T5D9T35-full-labeled.pptx]

## Slide 1
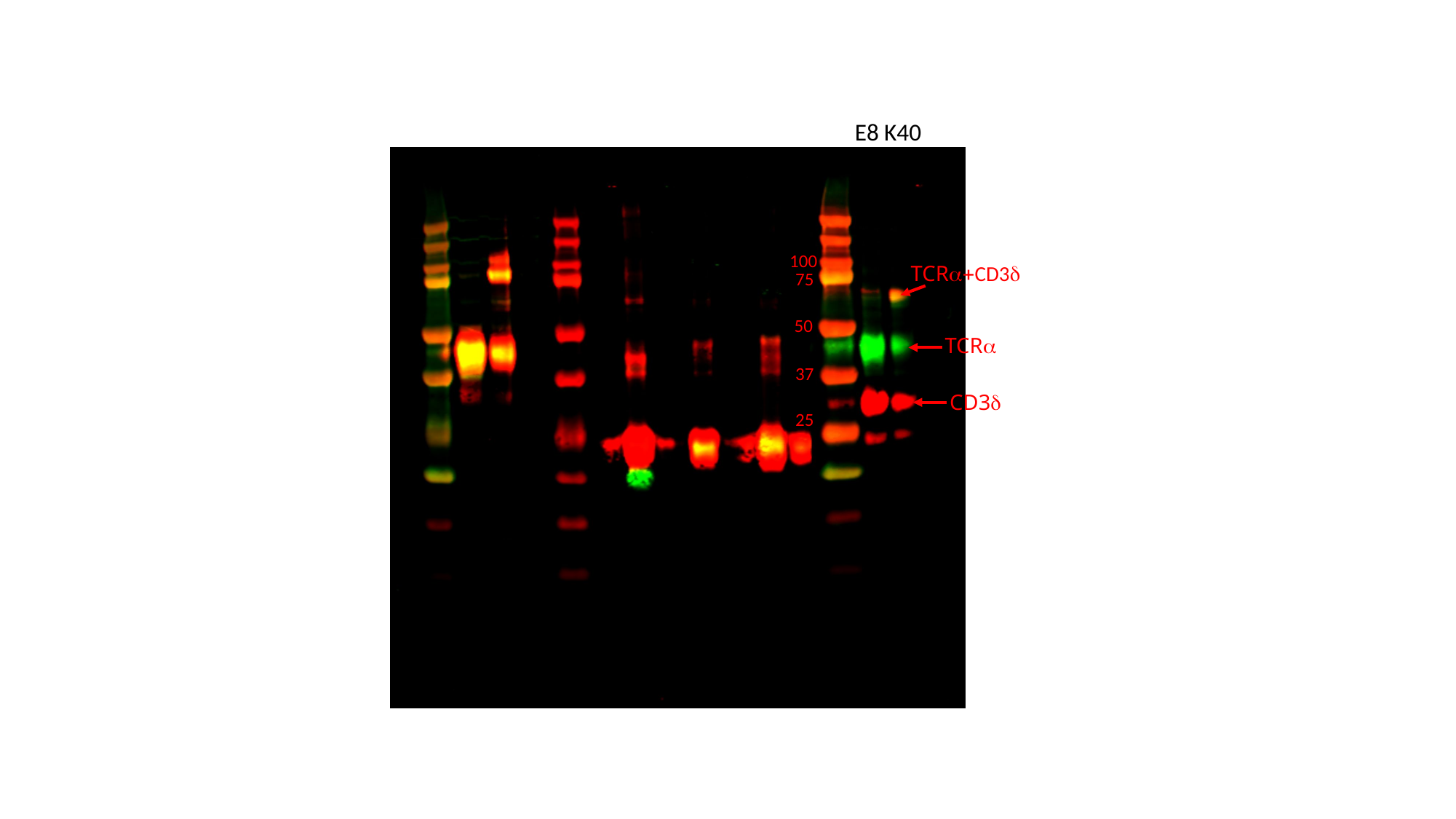

E8 K40
100
TCRa+CD3d
75
50
TCRa
37
CD3d
25

Supplement: Supplementary file 8 — Source data Fig. 3 [file 44319_2024_314_MOESM8_ESM.zip › Fig3_WB /Figure 3B/E8K40-full.pptx]

T17

V26

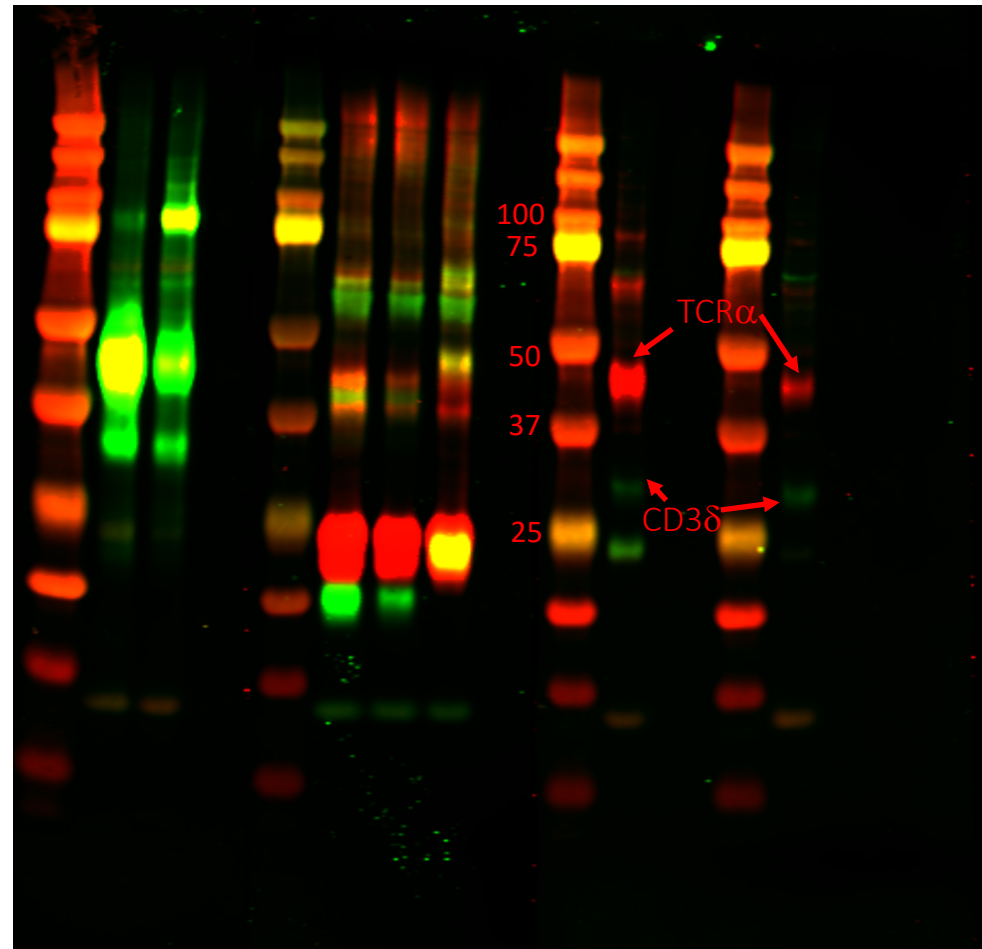

Supplement: Supplementary file 8 — Source data Fig. 3 [file 44319_2024_314_MOESM8_ESM.zip › Fig3_WB /Figure 3B/T17V26-full-labeled.pdf]

E8 K40

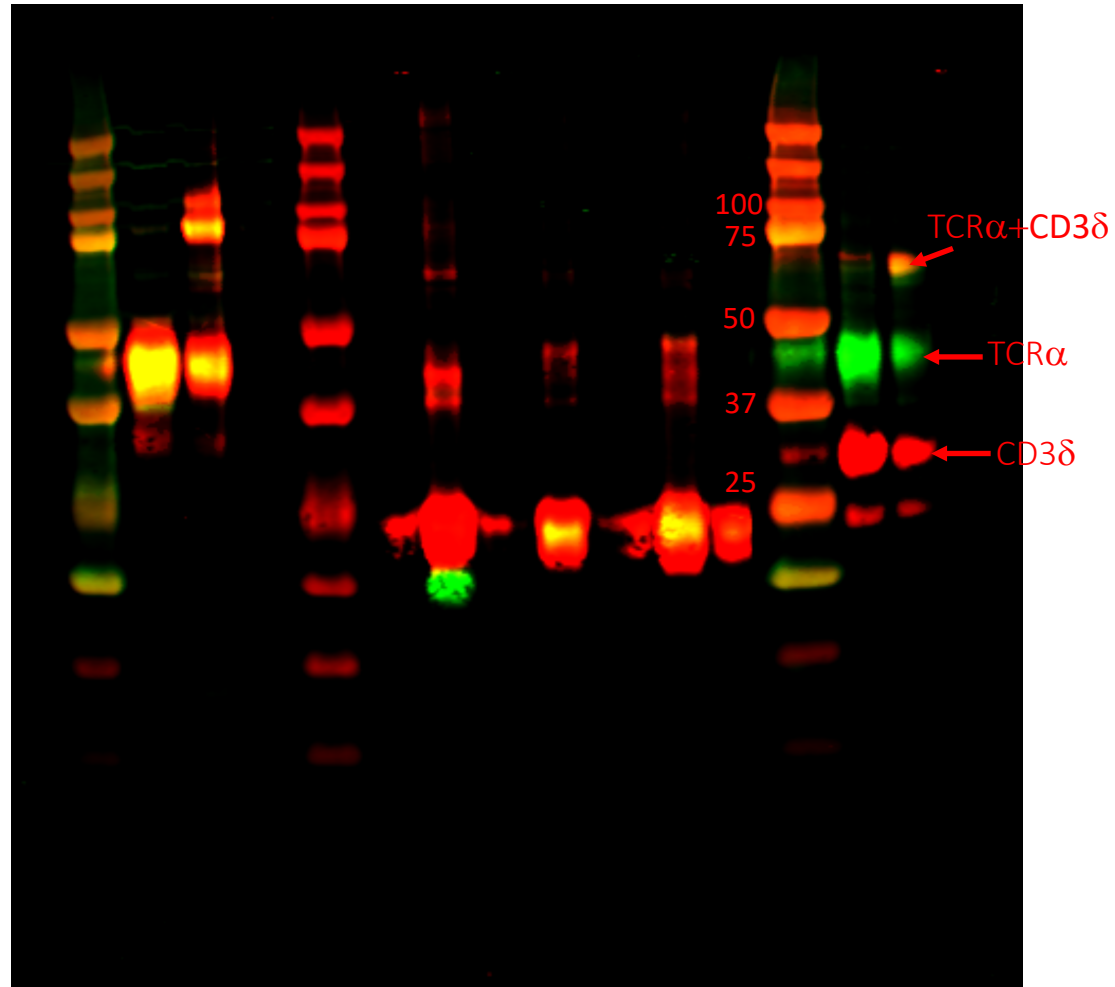

Supplement: Supplementary file 8 — Source data Fig. 3 [file 44319_2024_314_MOESM8_ESM.zip › Fig3_WB /Figure 3B/E8K40-full-labeled.pdf]

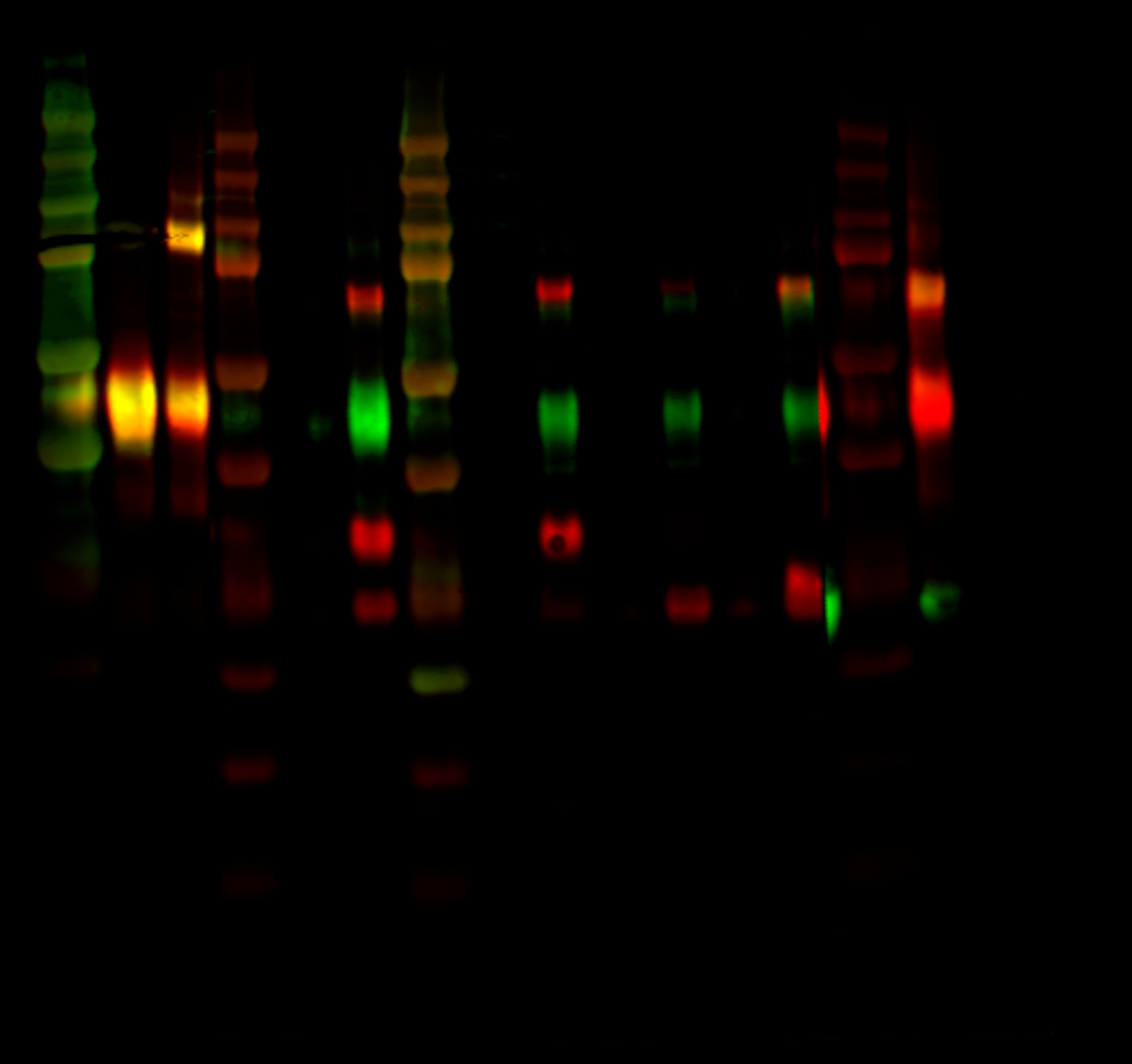

Supplement: Supplementary file 8 — Source data Fig. 3 [file 44319_2024_314_MOESM8_ESM.zip › Fig3_WB /Figure 3B/dK40-V5FLAG-full.png]

K40

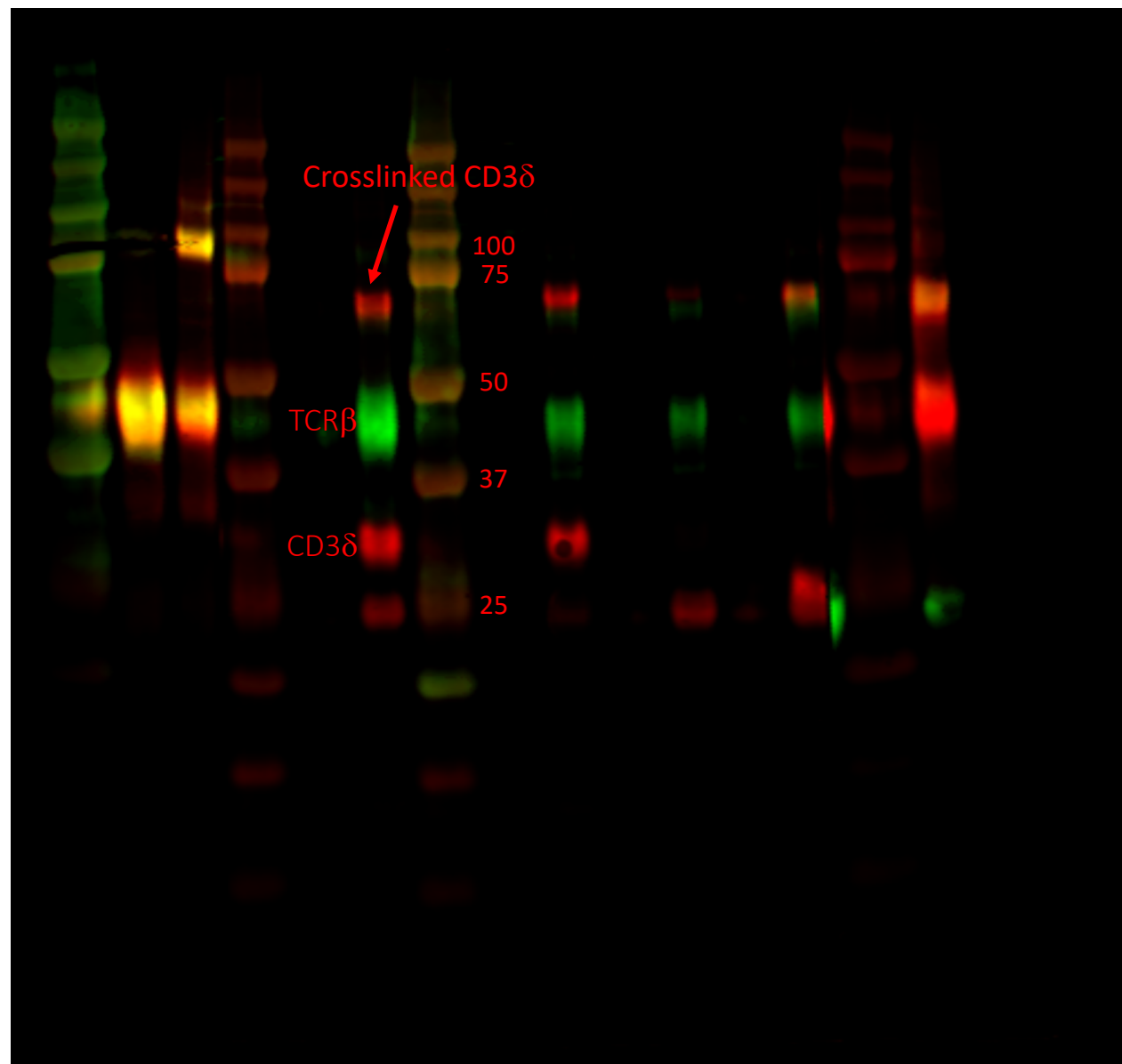

Supplement: Supplementary file 8 — Source data Fig. 3 [file 44319_2024_314_MOESM8_ESM.zip › Fig3_WB /Figure 3B/dK40-V5FLAG-full-labeled.pdf]

## Slide 1
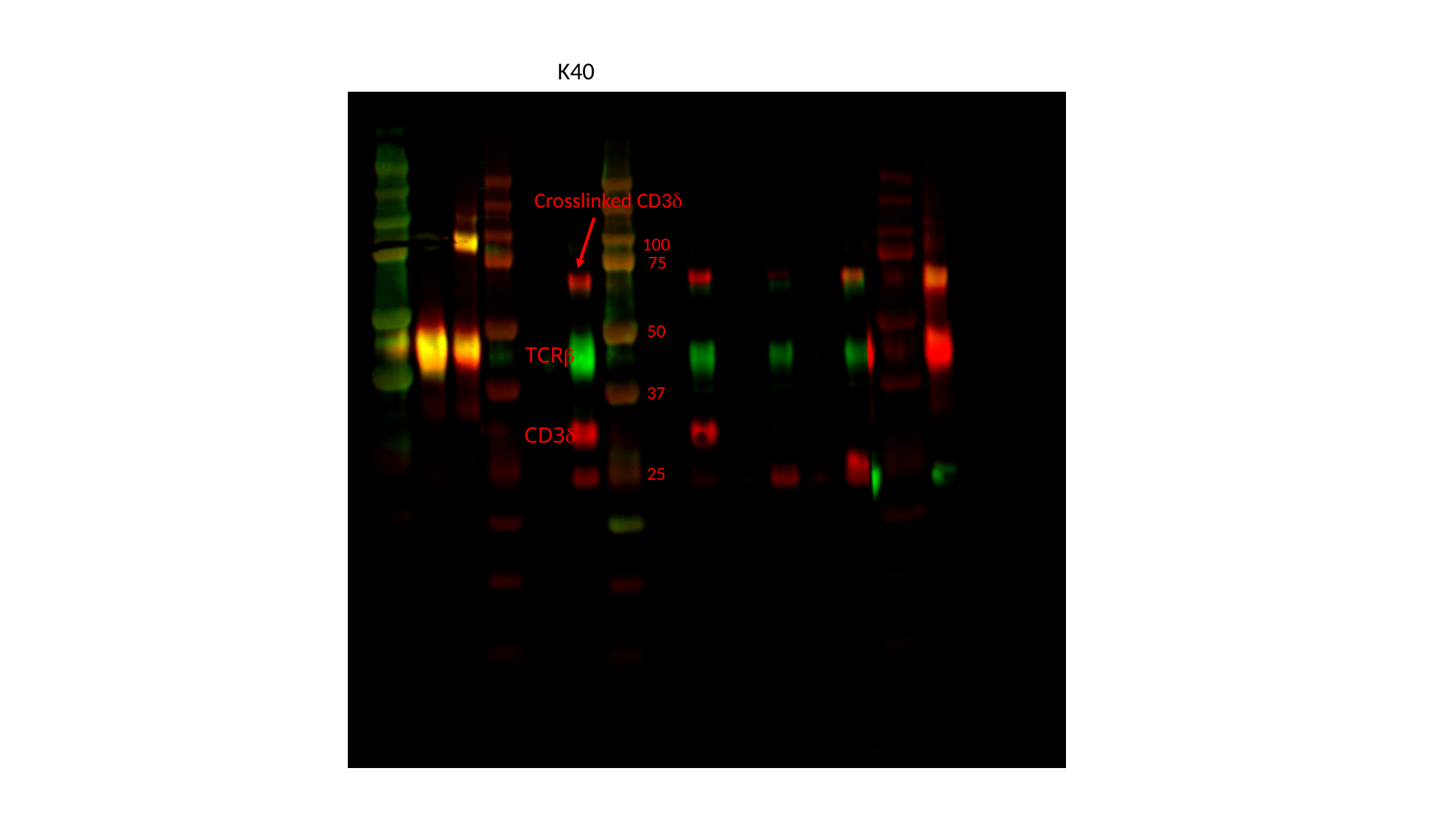

K40
Crosslinked CD3d
100
75
50
TCRb
37
CD3d
25

Supplement: Supplementary file 8 — Source data Fig. 3 [file 44319_2024_314_MOESM8_ESM.zip › Fig3_WB /Figure 3B/dK40-V5FLAG-full-labeled.pptx]

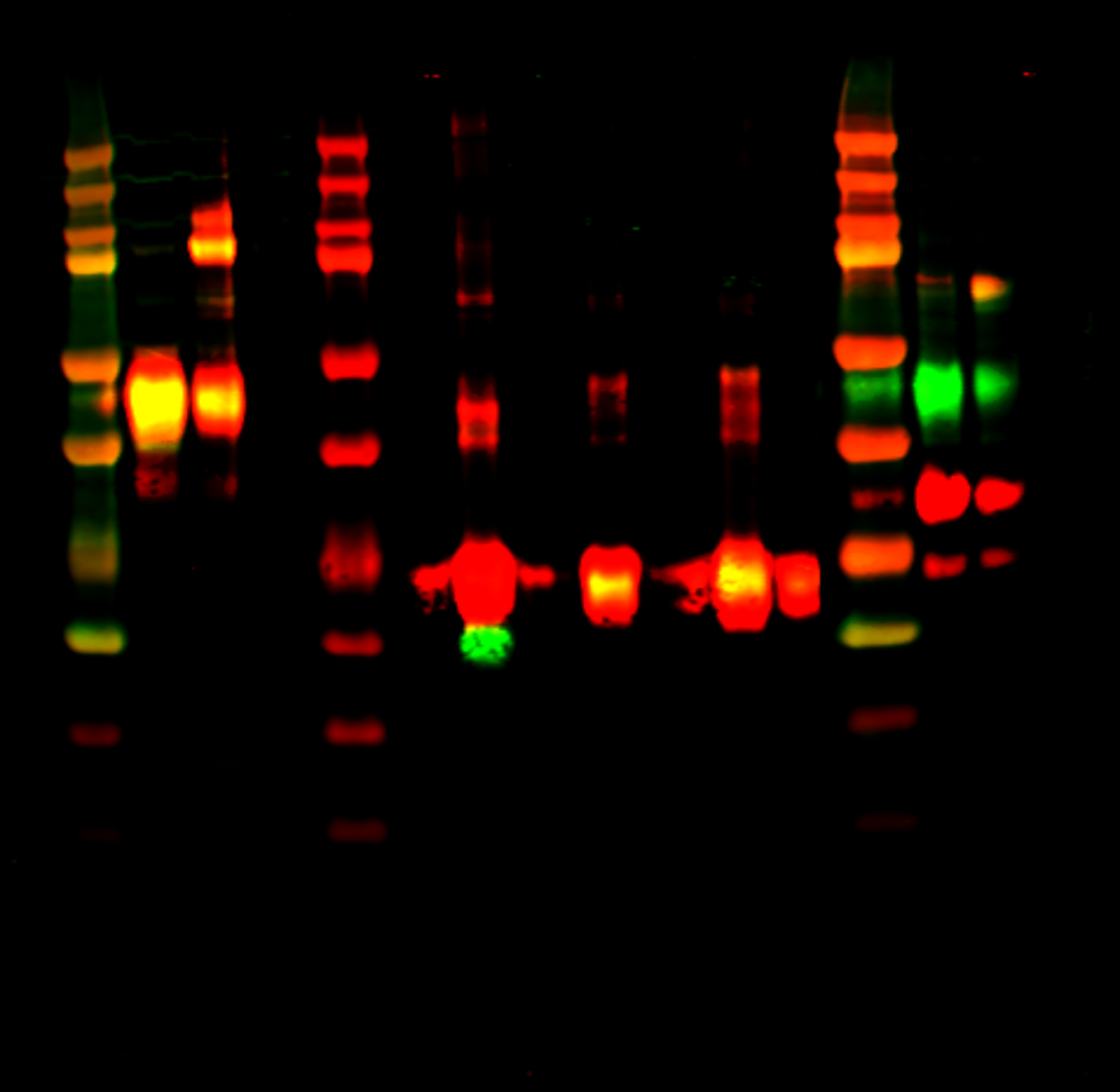

Supplement: Supplementary file 8 — Source data Fig. 3 [file 44319_2024_314_MOESM8_ESM.zip › Fig3_WB /Figure 3B/E8K40-full.png]

## Slide 1
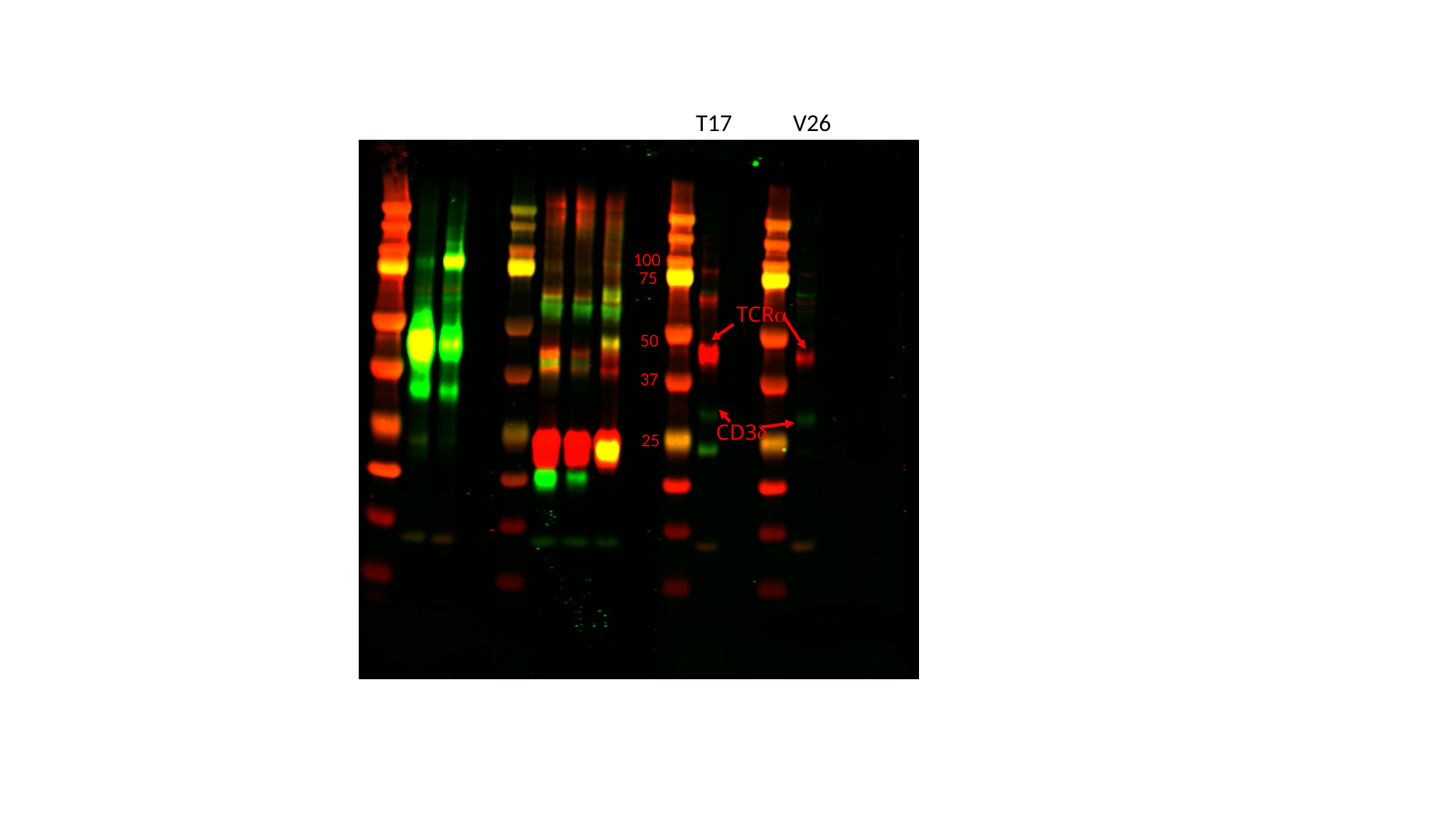

T17
V26
100
75
TCRa
50
37
CD3d
25

Supplement: Supplementary file 8 — Source data Fig. 3 [file 44319_2024_314_MOESM8_ESM.zip › Fig3_WB /Figure 3B/T17V26-full.pptx]

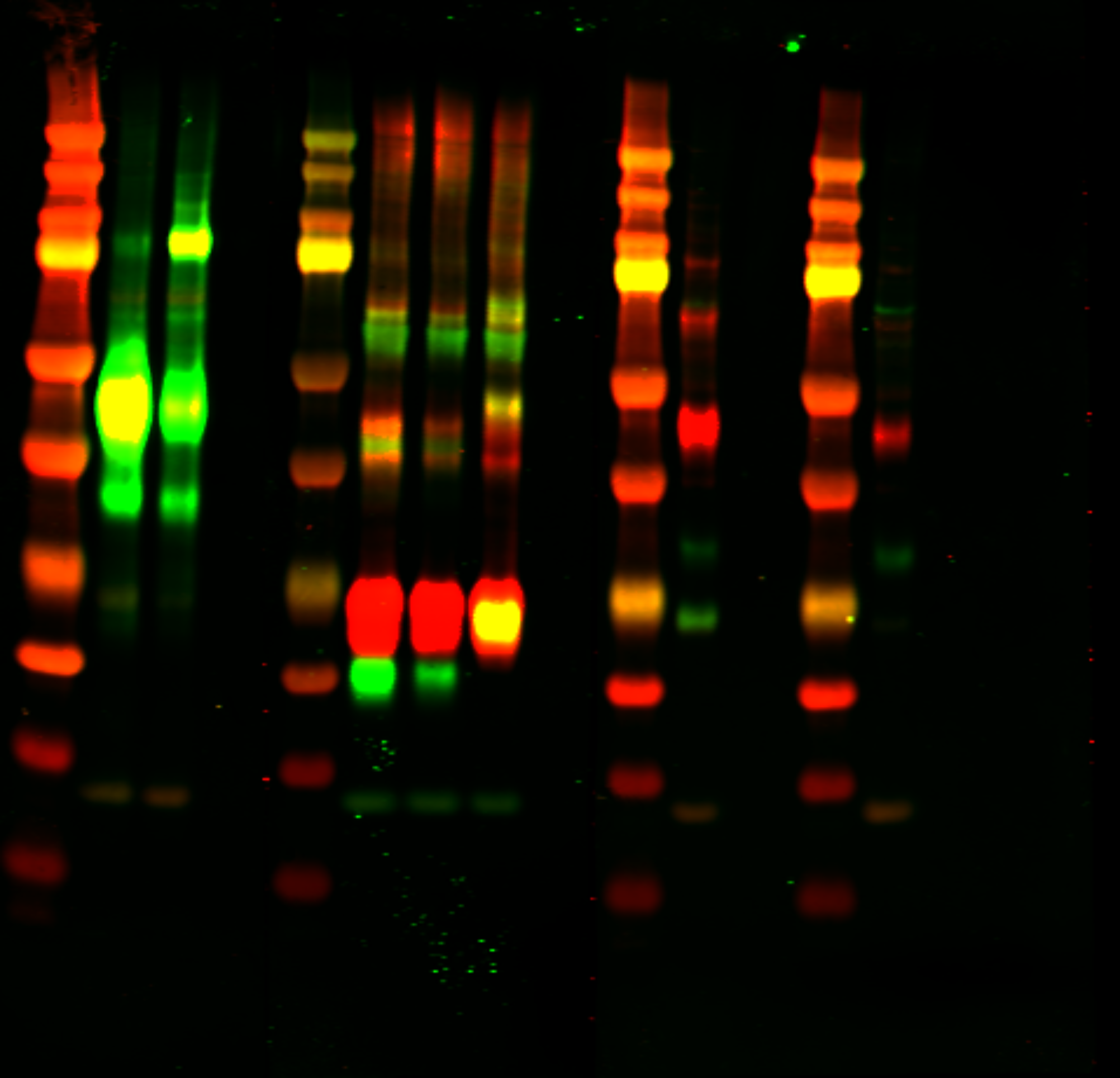

Supplement: Supplementary file 8 — Source data Fig. 3 [file 44319_2024_314_MOESM8_ESM.zip › Fig3_WB /Figure 3B/T17V26-full.png]

T5

T35

T5

D9

T35

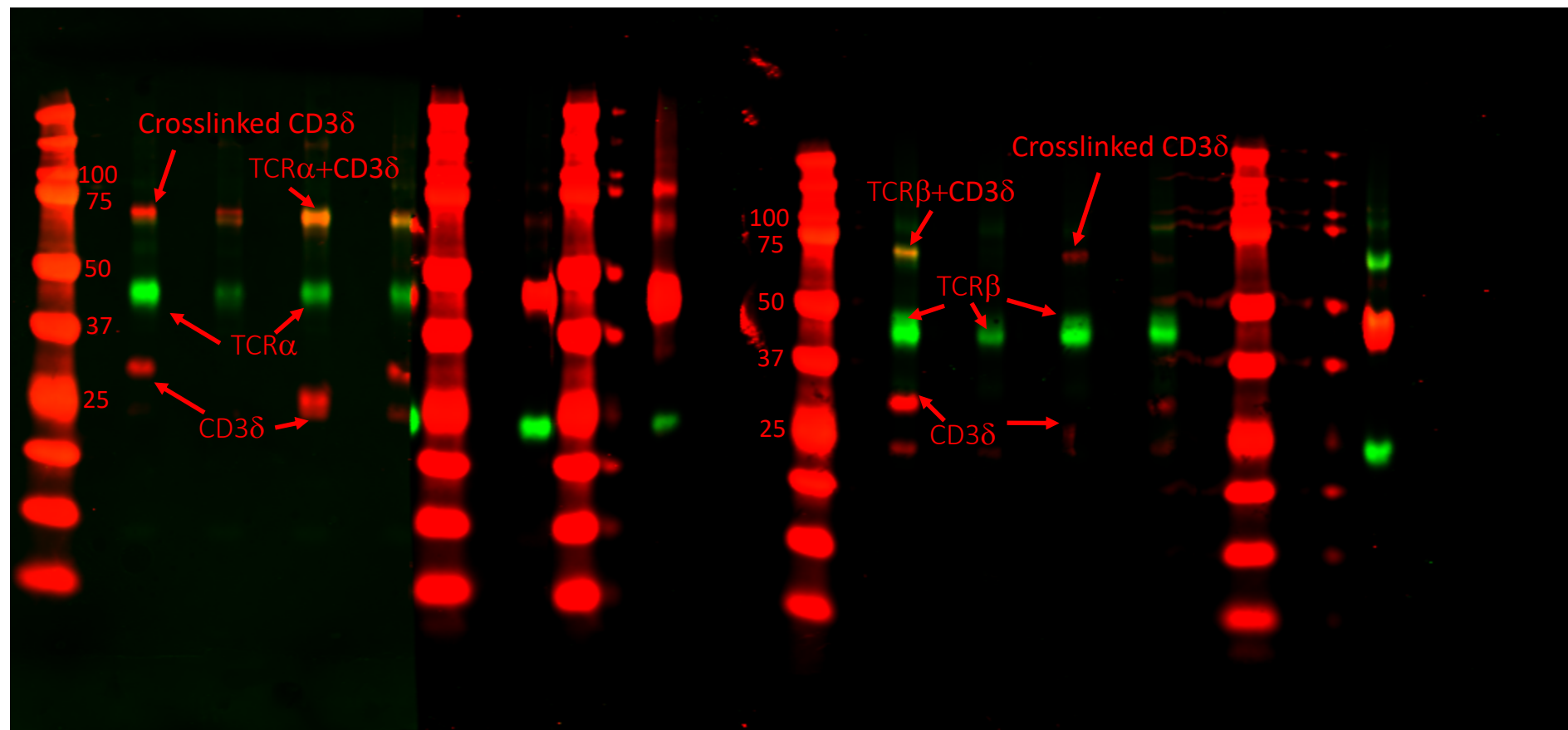

Supplement: Supplementary file 8 — Source data Fig. 3 [file 44319_2024_314_MOESM8_ESM.zip › Fig3_WB /Figure 3B/T5D9T35-full-labeled.pdf]

## Slide 1
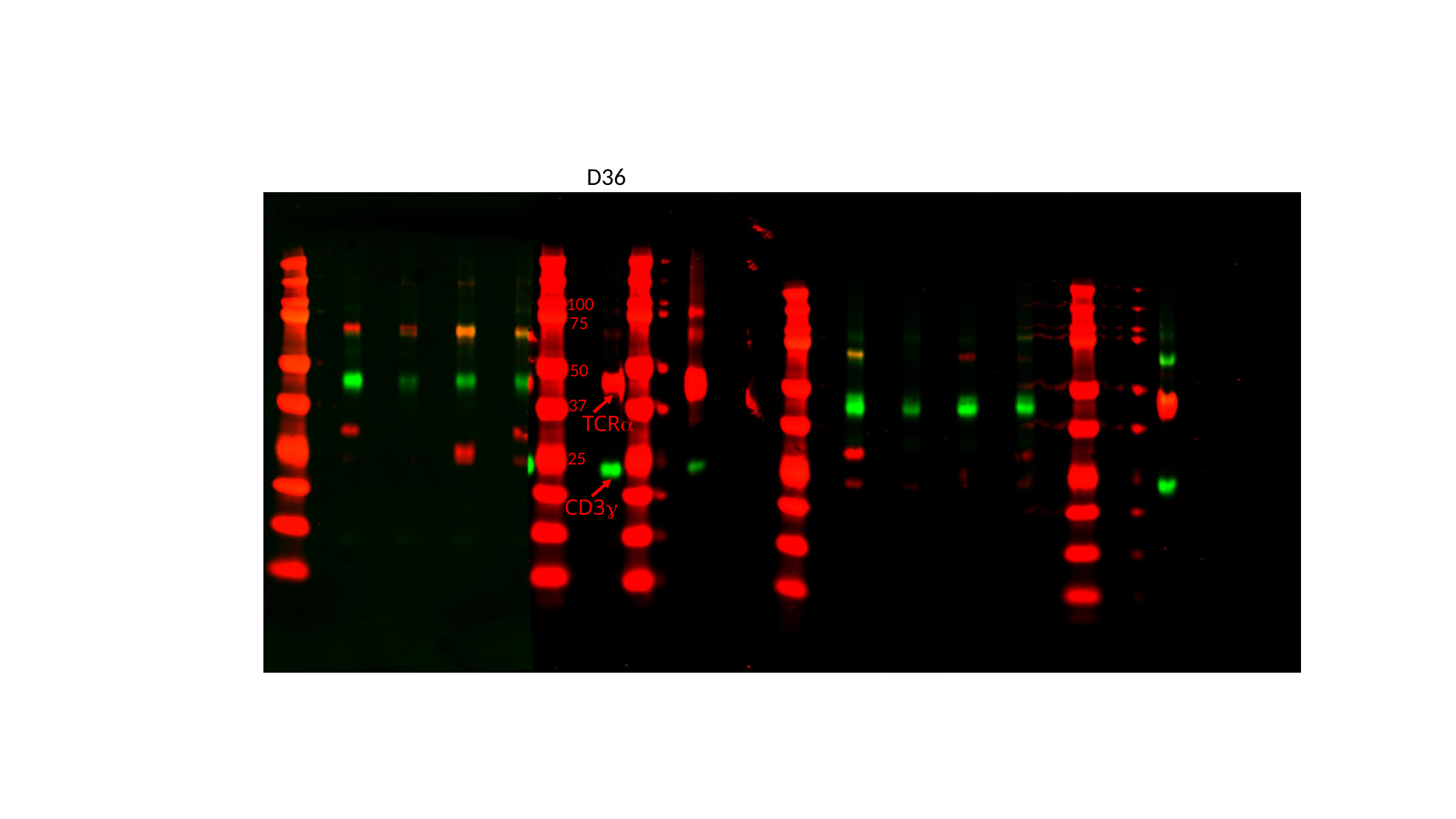

D36
100
75
50
37
TCRa
25
CD3g

Supplement: Supplementary file 8 — Source data Fig. 3 [file 44319_2024_314_MOESM8_ESM.zip › Fig3_WB /Figure 3C/D36-full-labeled.pptx]

## Slide 1
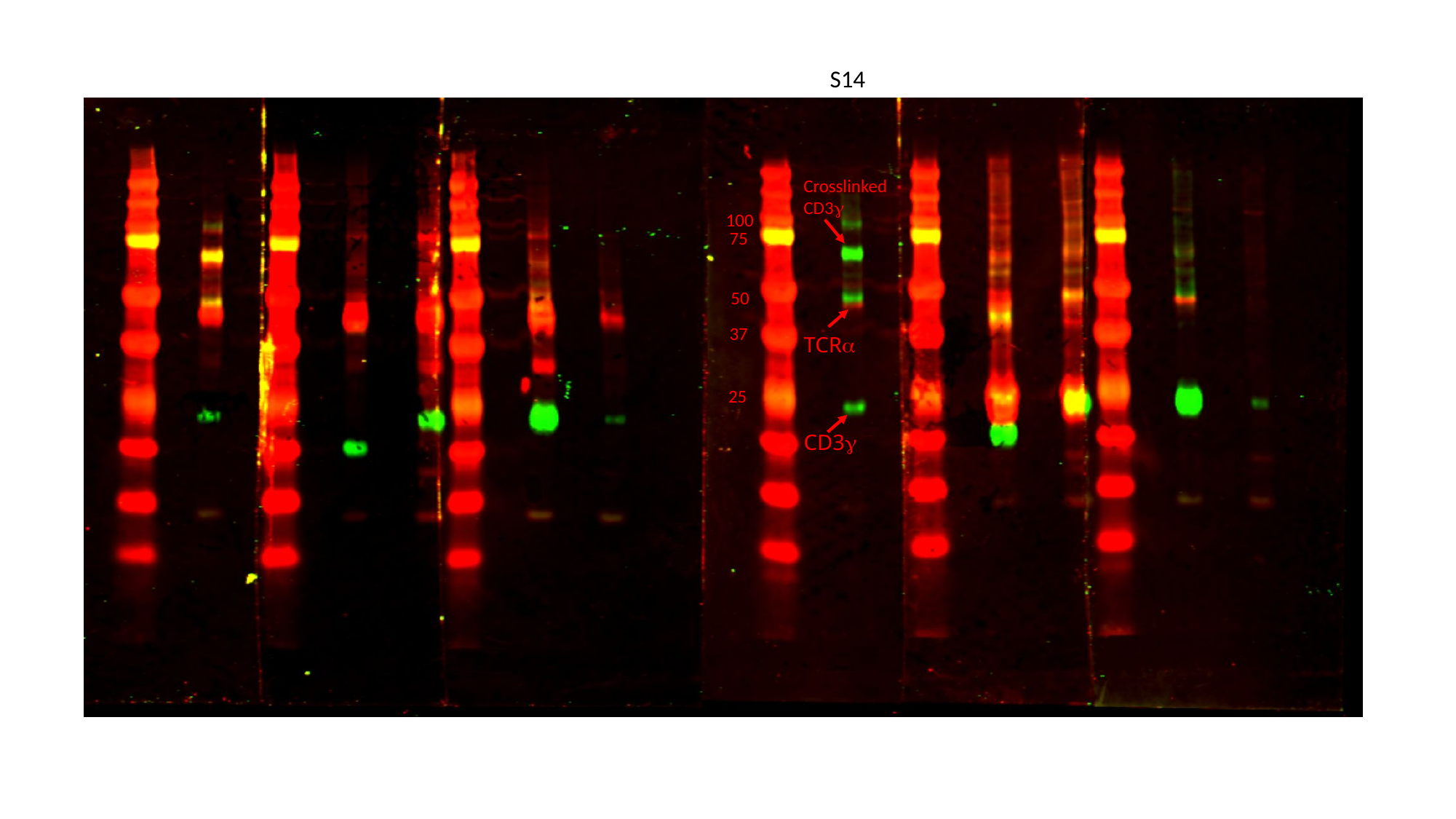

S14
Crosslinked
CD3g
100
75
50
37
TCRa
25
CD3g

Supplement: Supplementary file 8 — Source data Fig. 3 [file 44319_2024_314_MOESM8_ESM.zip › Fig3_WB /Figure 3C/gS14-full-cMycVSVG-labeled.pptx]
