# Supplementary figures and images for: In situ cell-surface conformation of the TCR-CD3 signaling complex (part 2 of 2)
Source: EMBO Rep. 2024 Nov 7;25(12):26. doi: 10.1038/s44319-024-00314-3 (PMC11624261; doi:10.1038/s44319-024-00314-3)

## Slide 1
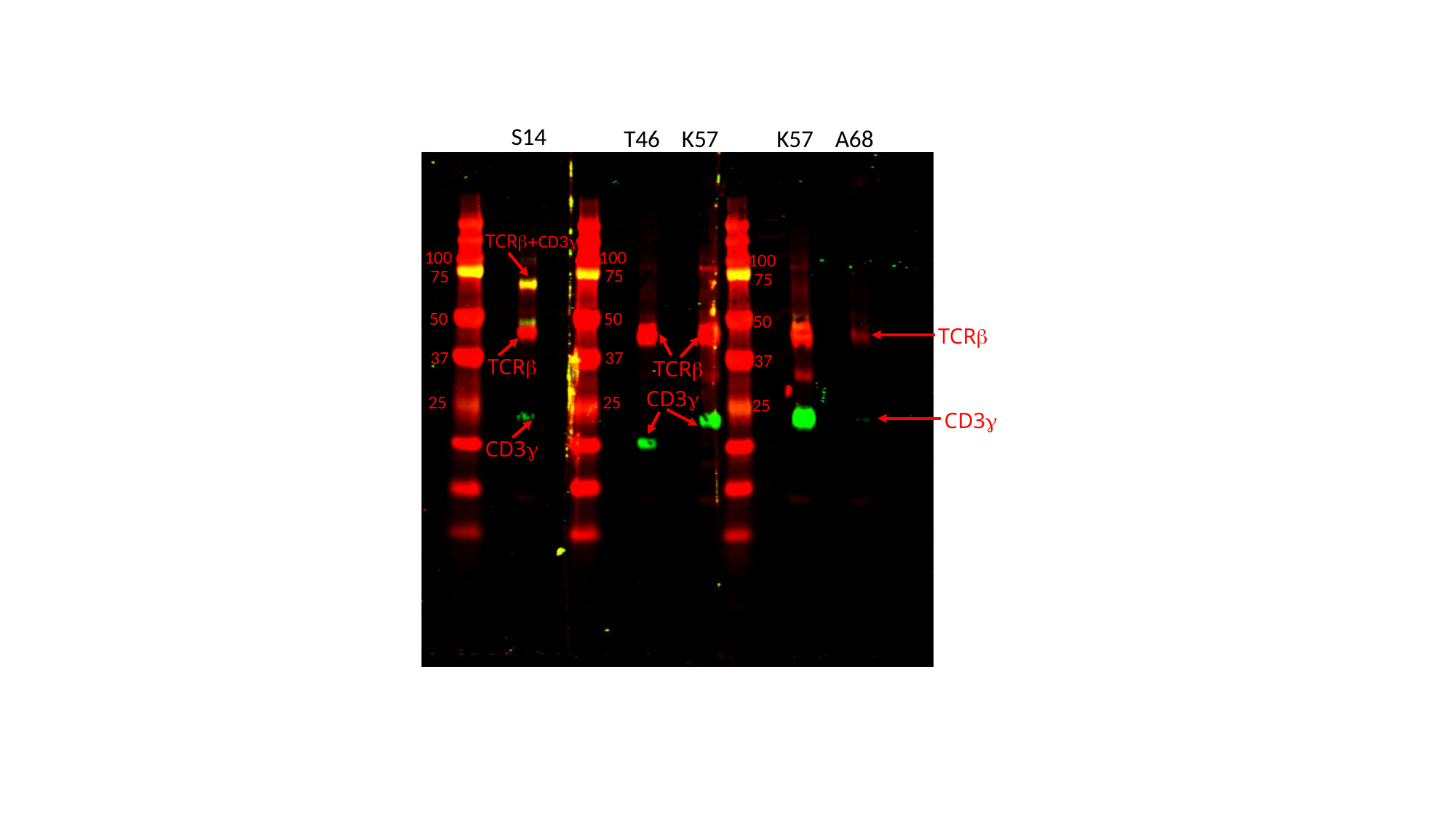

S14
T46 K57
K57 A68
TCRb+CD3g
100
100
100
75
75
75
50
50
50
TCRb
37
37
37
TCRb
TCRb
CD3g
25
25
25
CD3g
CD3g

Supplement: Supplementary file 8 — Source data Fig. 3 [file 44319_2024_314_MOESM8_ESM.zip › Fig3_WB /Figure 3C/S14T46K47K57A68-full.pptx]

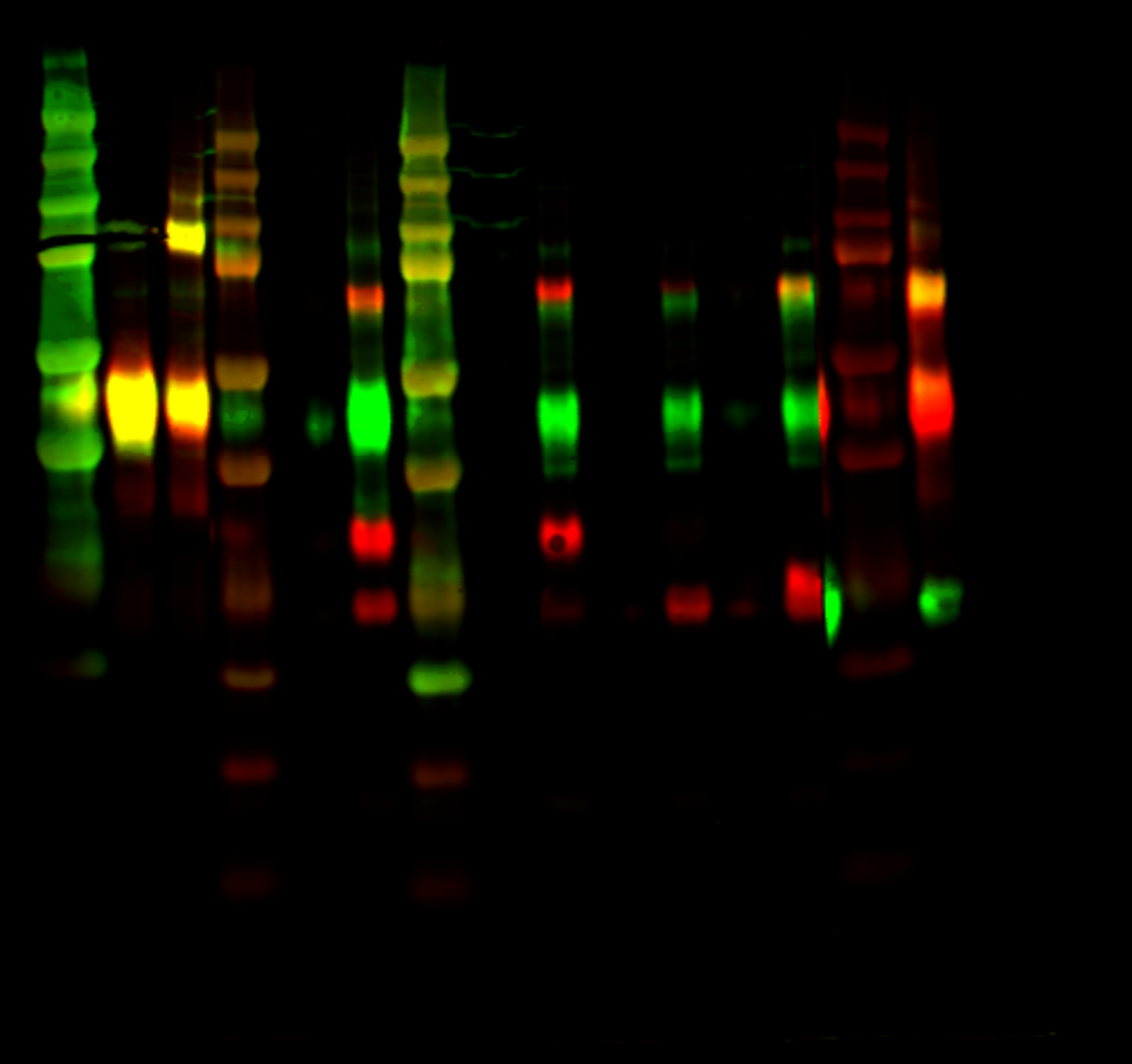

Supplement: Supplementary file 8 — Source data Fig. 3 [file 44319_2024_314_MOESM8_ESM.zip › Fig3_WB /Figure 3C/R15-full.png]

R15

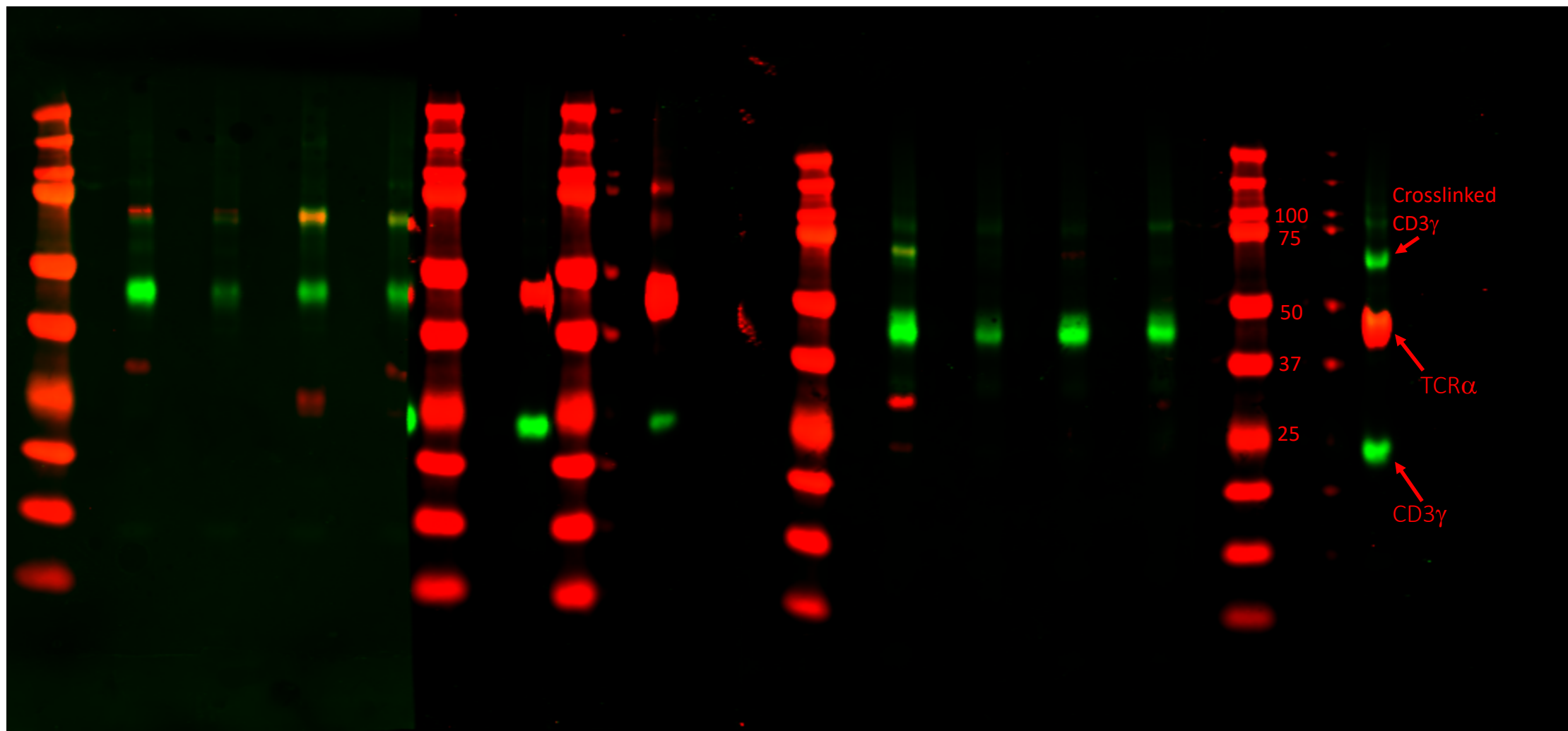

Supplement: Supplementary file 8 — Source data Fig. 3 [file 44319_2024_314_MOESM8_ESM.zip › Fig3_WB /Figure 3C/gR15-full-cMycVSVG-labeled.pdf]

## Slide 1
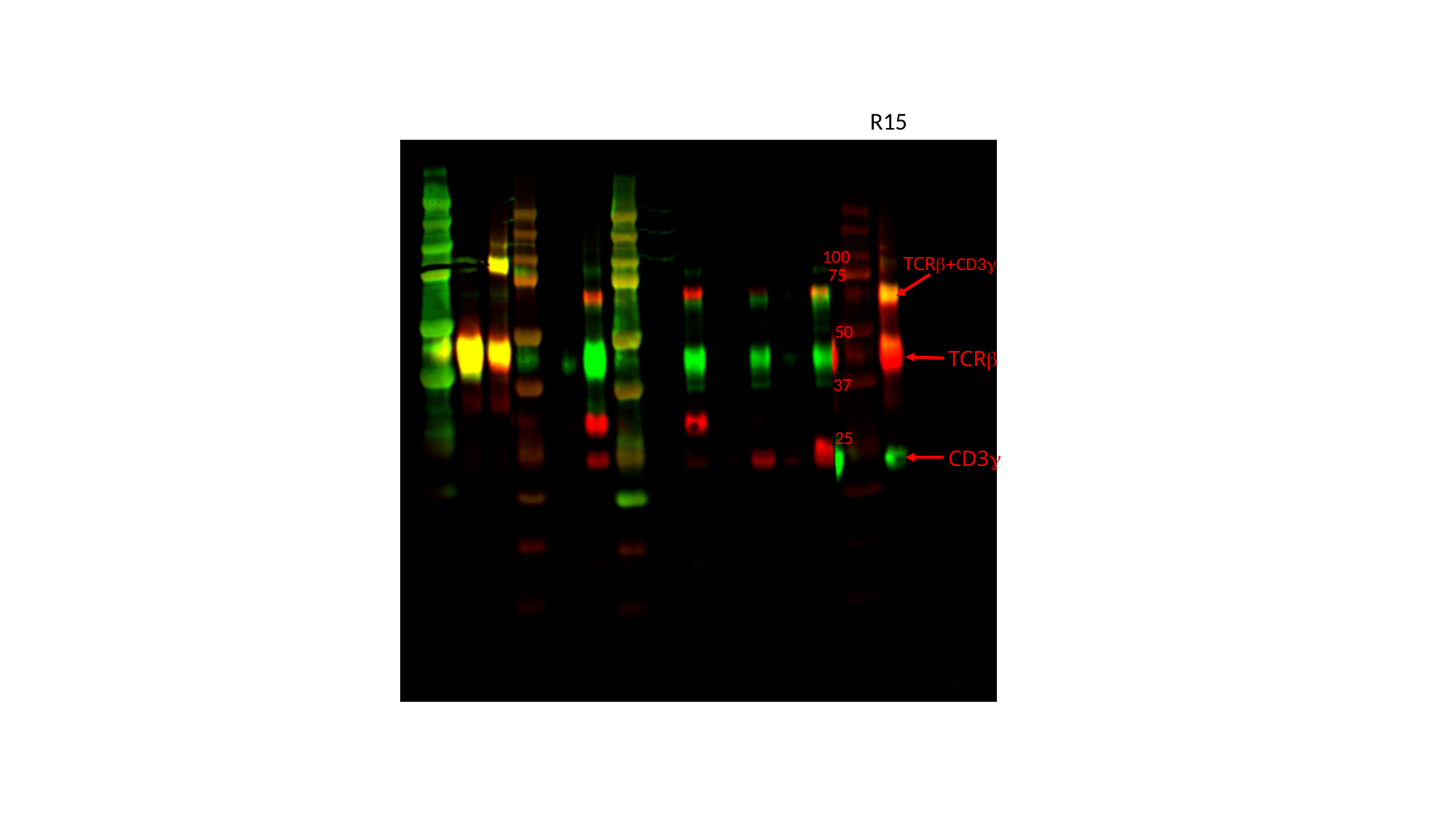

R15
100
TCRb+CD3g
75
50
TCRb
37
25
CD3g

Supplement: Supplementary file 8 — Source data Fig. 3 [file 44319_2024_314_MOESM8_ESM.zip › Fig3_WB /Figure 3C/R15-full.pptx]

## Slide 1
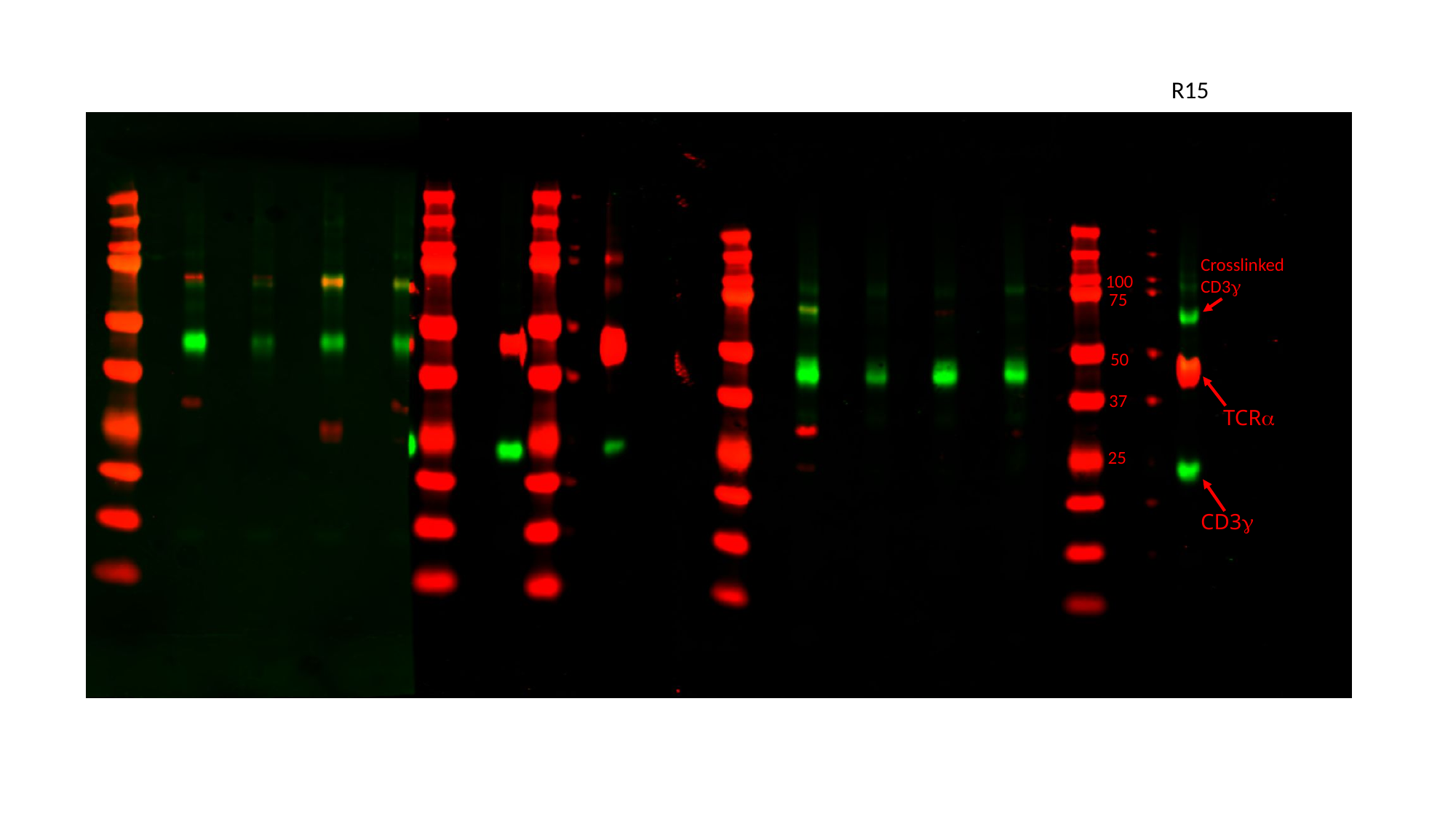

R15
Crosslinked
CD3g
100
75
50
37
TCRa
25
CD3g

Supplement: Supplementary file 8 — Source data Fig. 3 [file 44319_2024_314_MOESM8_ESM.zip › Fig3_WB /Figure 3C/gR15-full-cMycVSVG-labeled.pptx]

D36

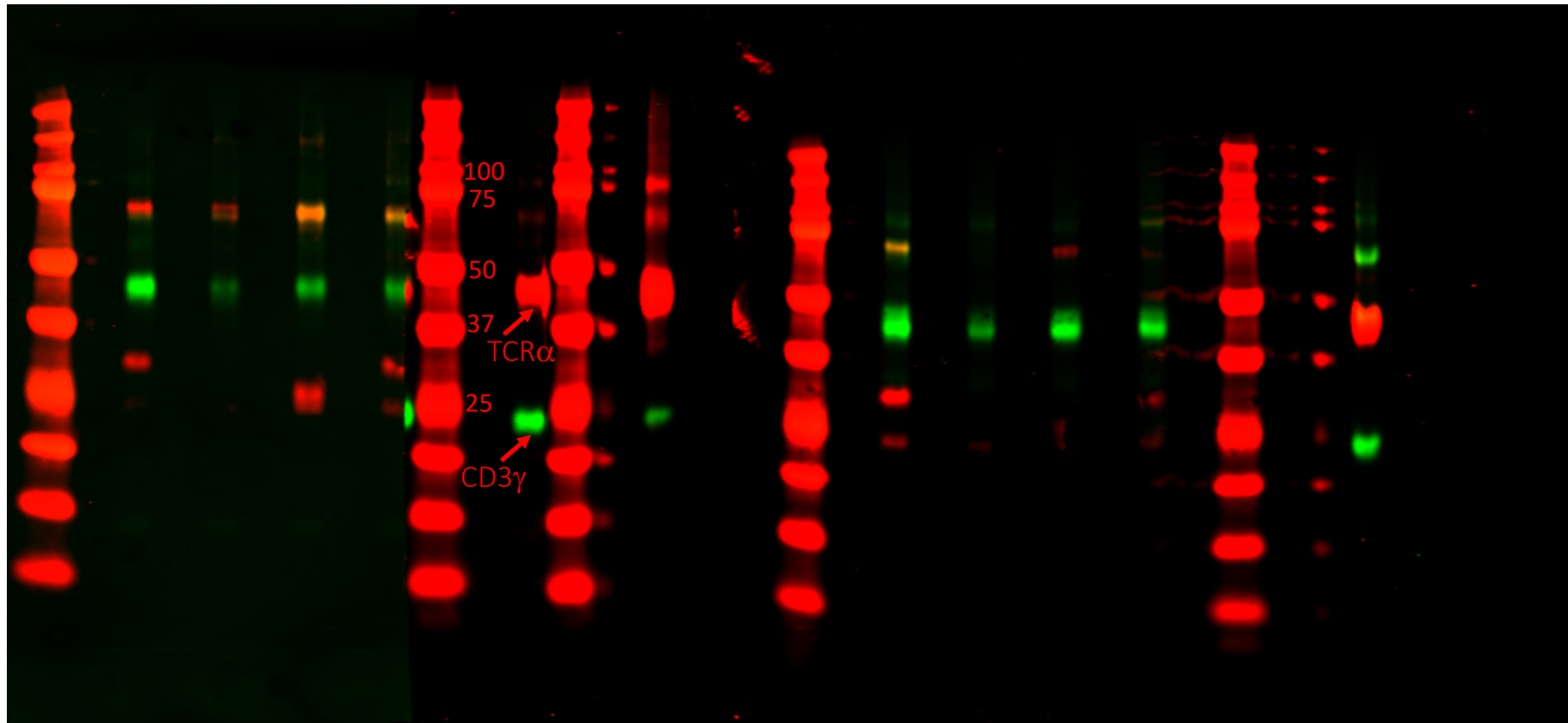

Supplement: Supplementary file 8 — Source data Fig. 3 [file 44319_2024_314_MOESM8_ESM.zip › Fig3_WB /Figure 3C/D36-full-labeled.pdf]

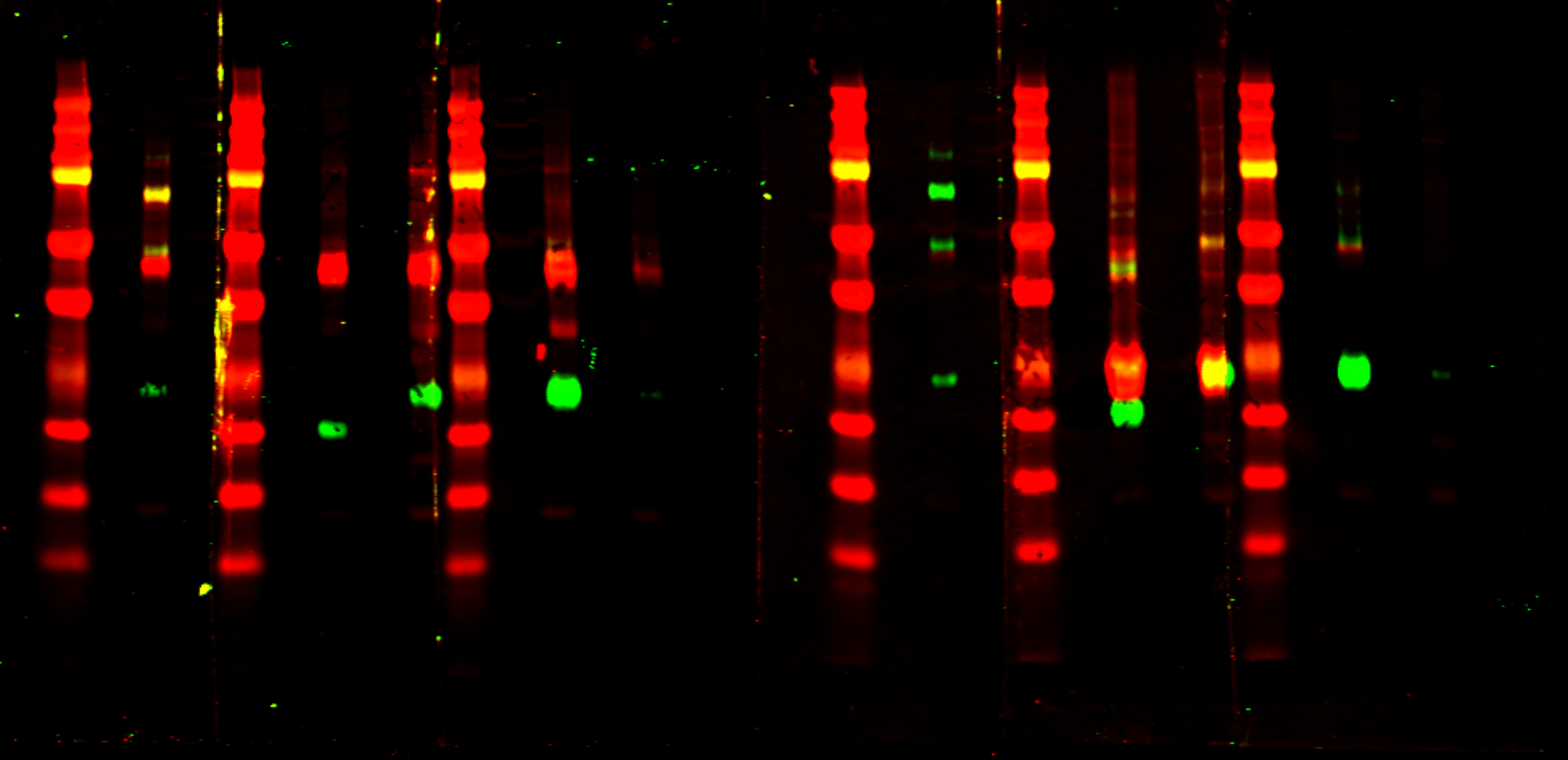

Supplement: Supplementary file 8 — Source data Fig. 3 [file 44319_2024_314_MOESM8_ESM.zip › Fig3_WB /Figure 3C/S14T46K47K57A68-full.png]

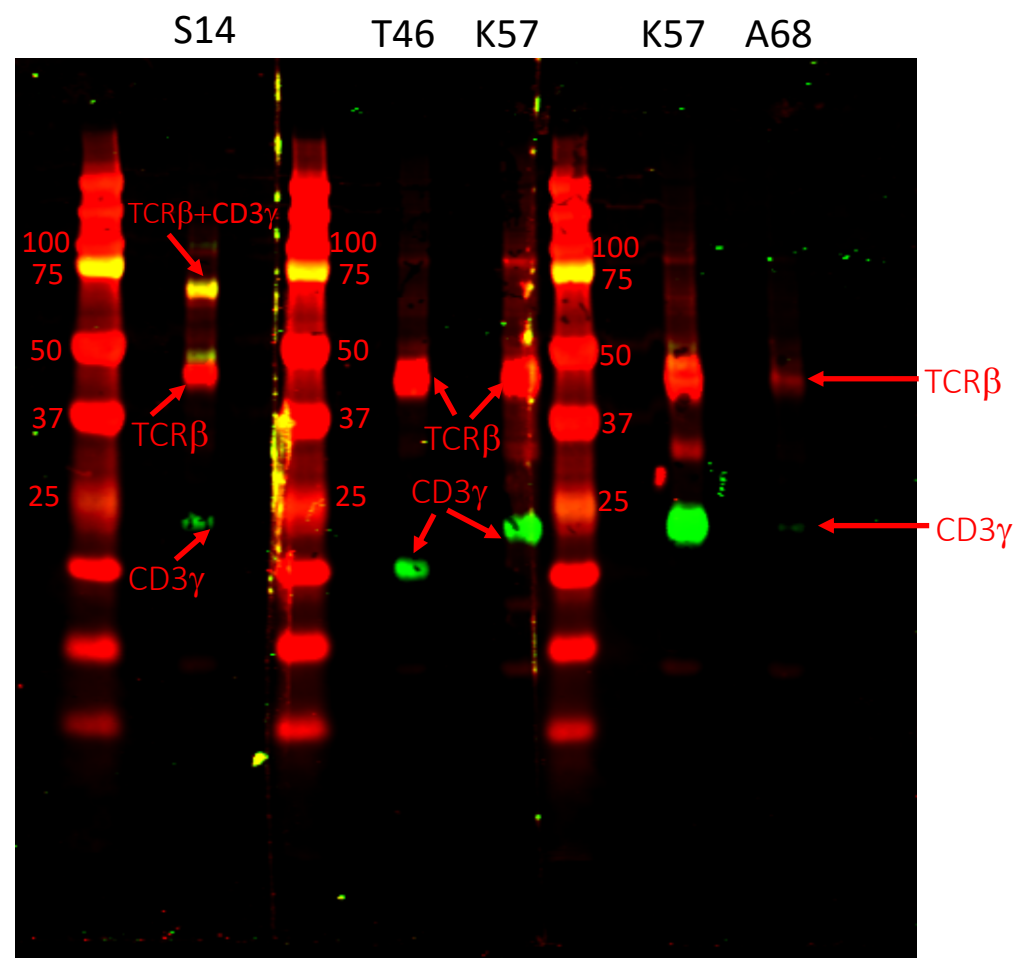

Supplement: Supplementary file 8 — Source data Fig. 3 [file 44319_2024_314_MOESM8_ESM.zip › Fig3_WB /Figure 3C/S14T46K47K57A68-full.pdf]

S14

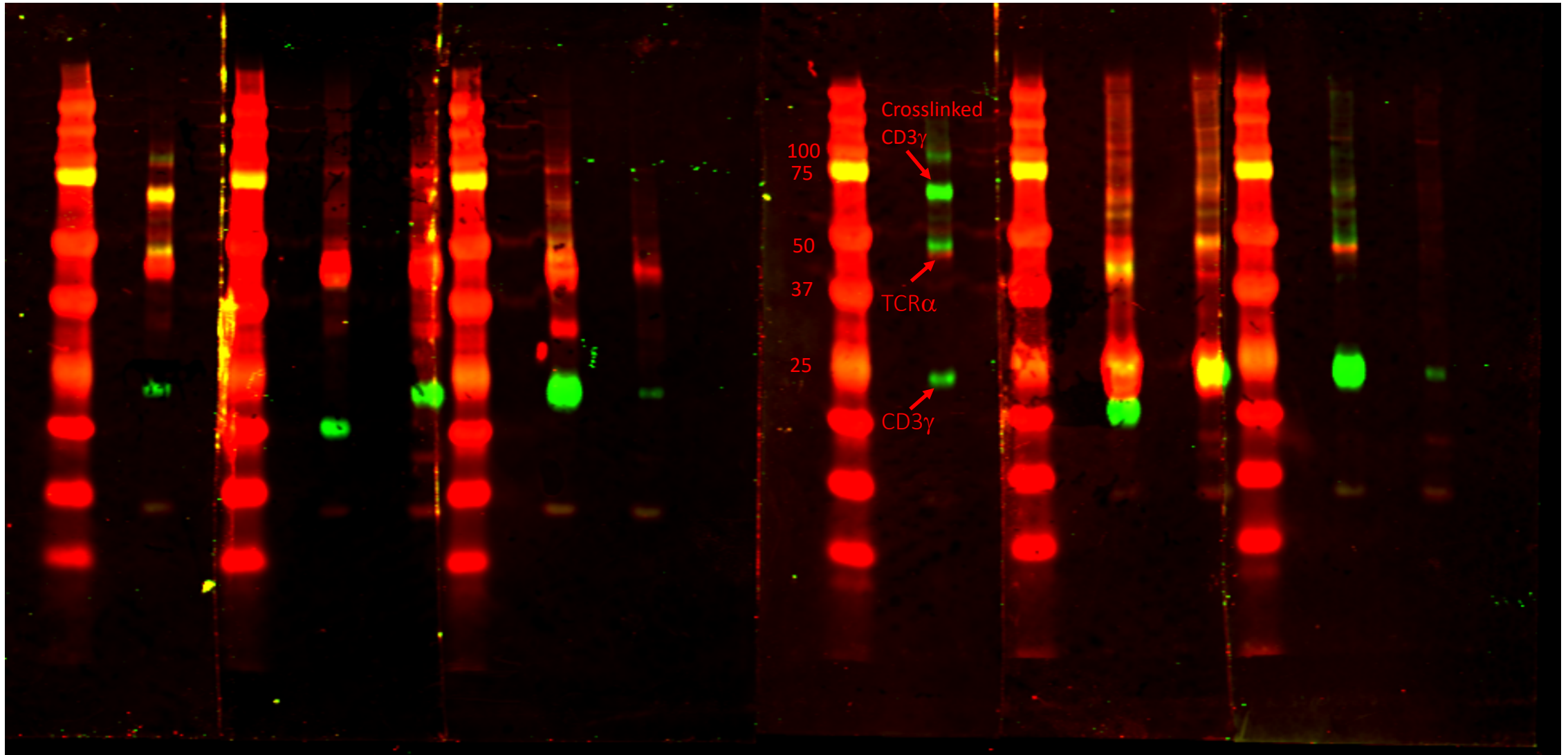

Supplement: Supplementary file 8 — Source data Fig. 3 [file 44319_2024_314_MOESM8_ESM.zip › Fig3_WB /Figure 3C/gS14-full-cMycVSVG-labeled.pdf]

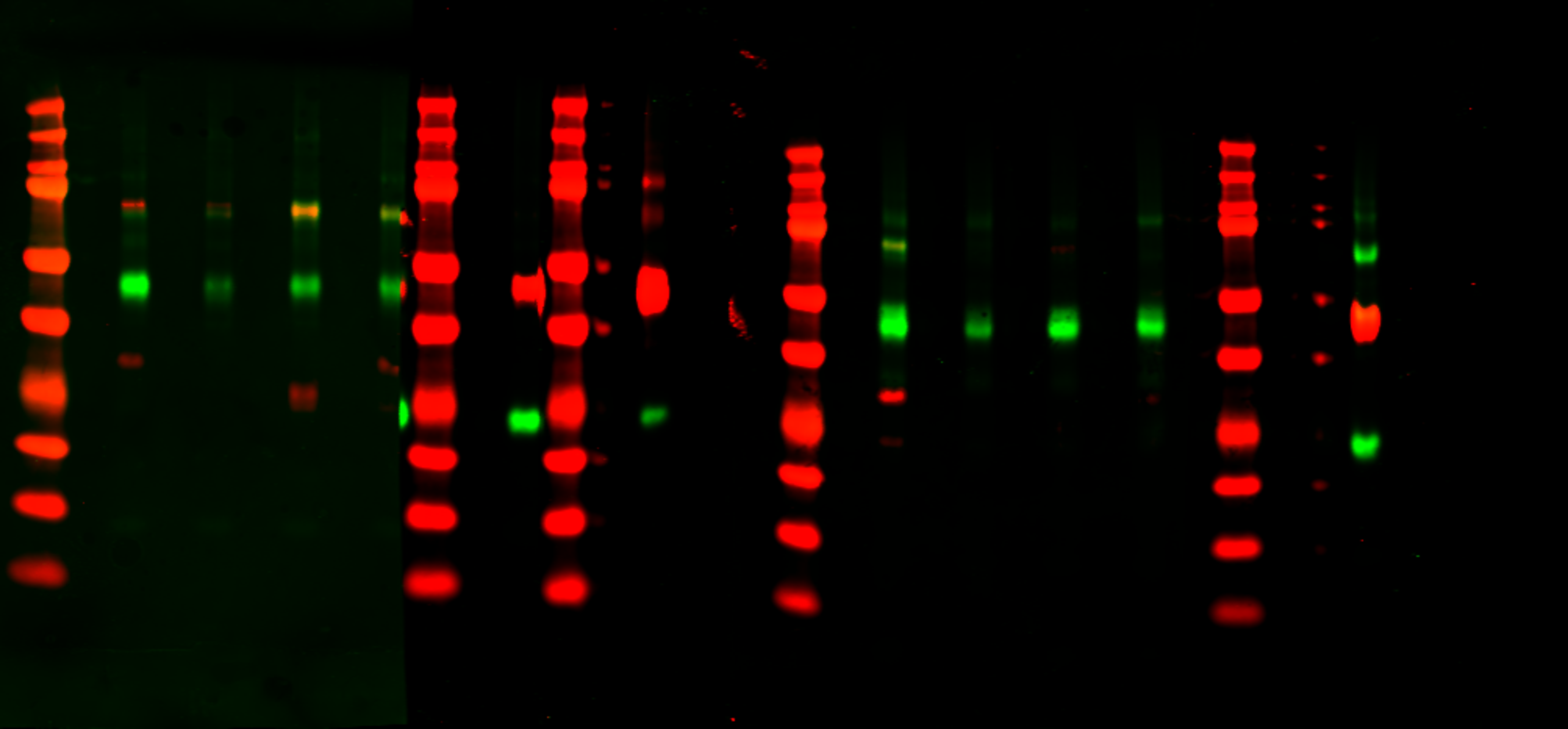

Supplement: Supplementary file 8 — Source data Fig. 3 [file 44319_2024_314_MOESM8_ESM.zip › Fig3_WB /Figure 3C/gR15-full-cMycVSVG.png]

R15

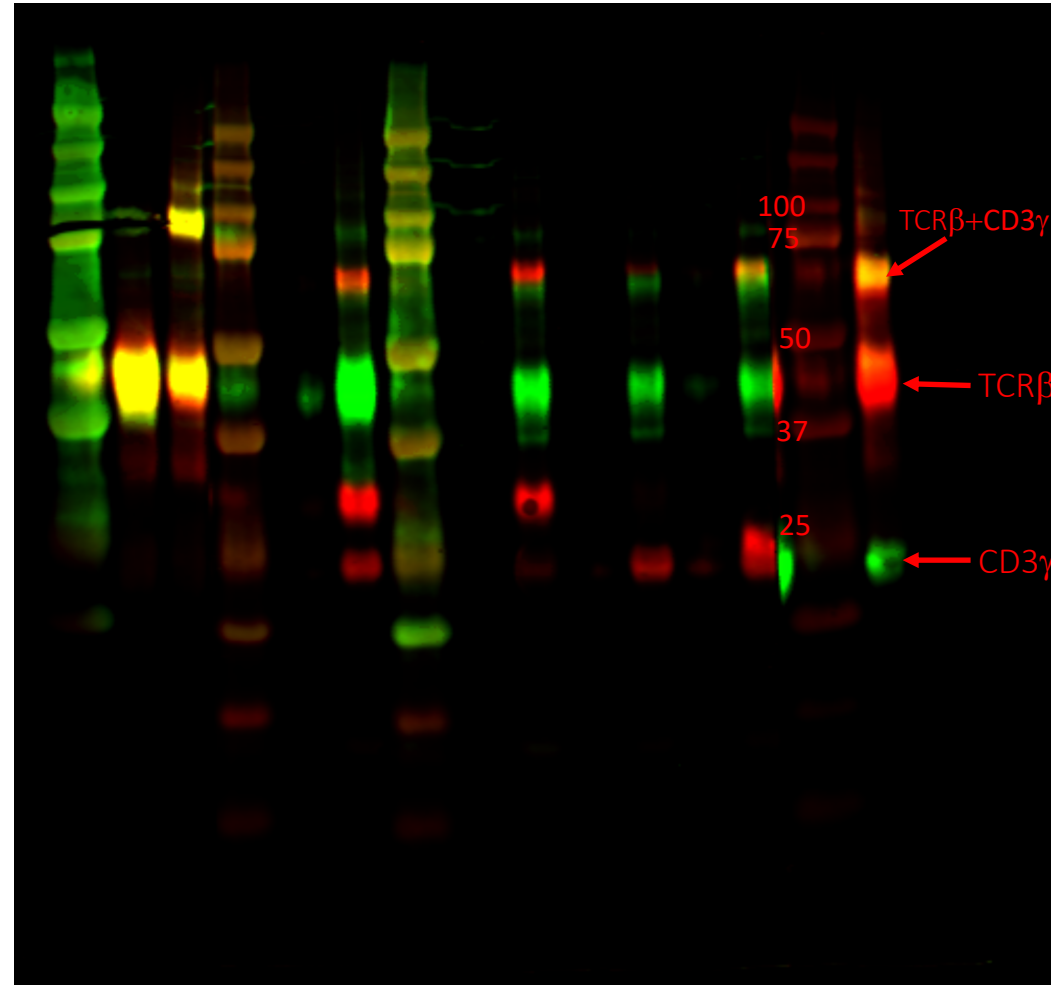

Supplement: Supplementary file 8 — Source data Fig. 3 [file 44319_2024_314_MOESM8_ESM.zip › Fig3_WB /Figure 3C/R15-full-labeled.pdf]

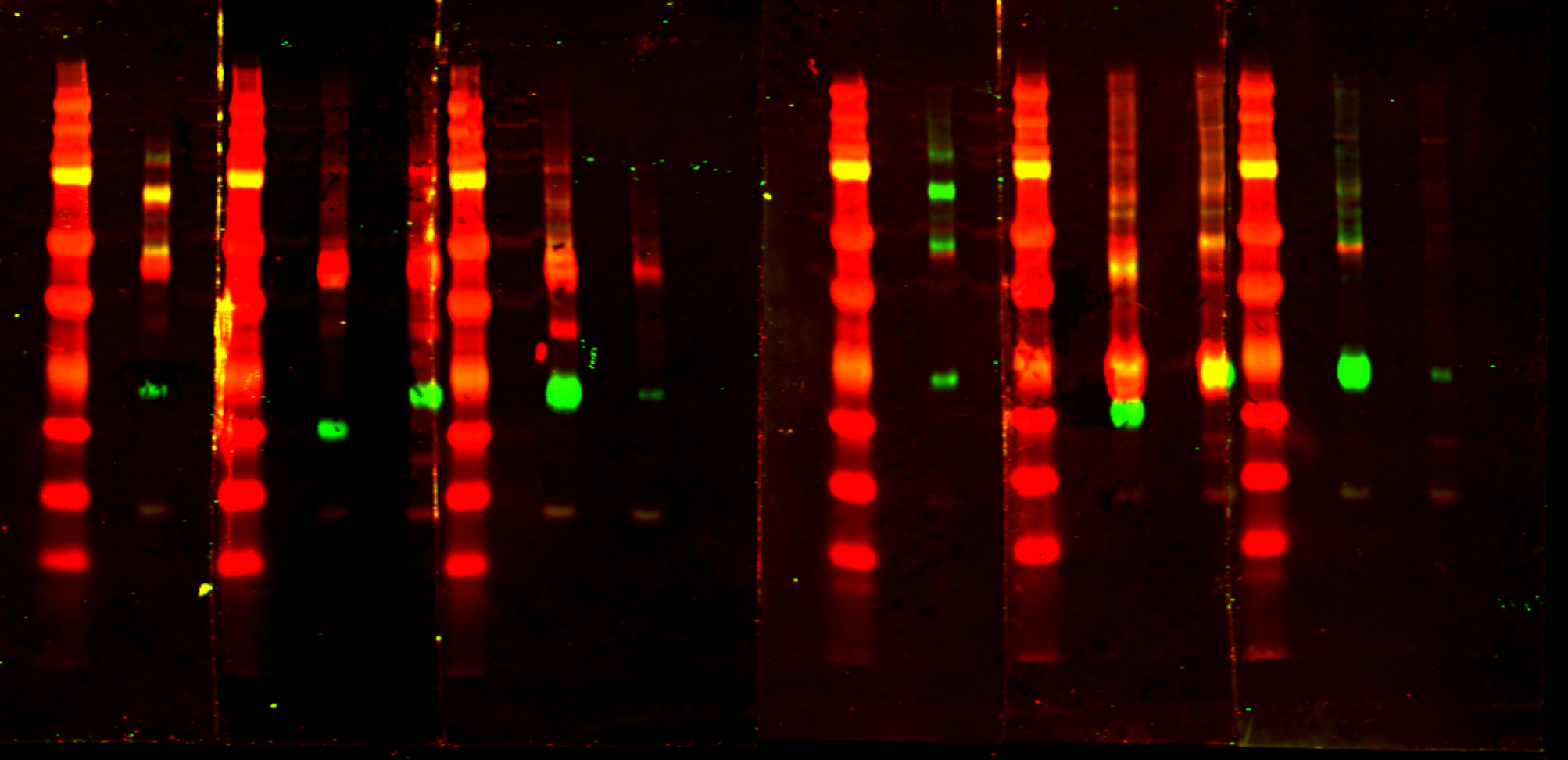

Supplement: Supplementary file 8 — Source data Fig. 3 [file 44319_2024_314_MOESM8_ESM.zip › Fig3_WB /Figure 3C/gS14-full-cMycVSVG.png]

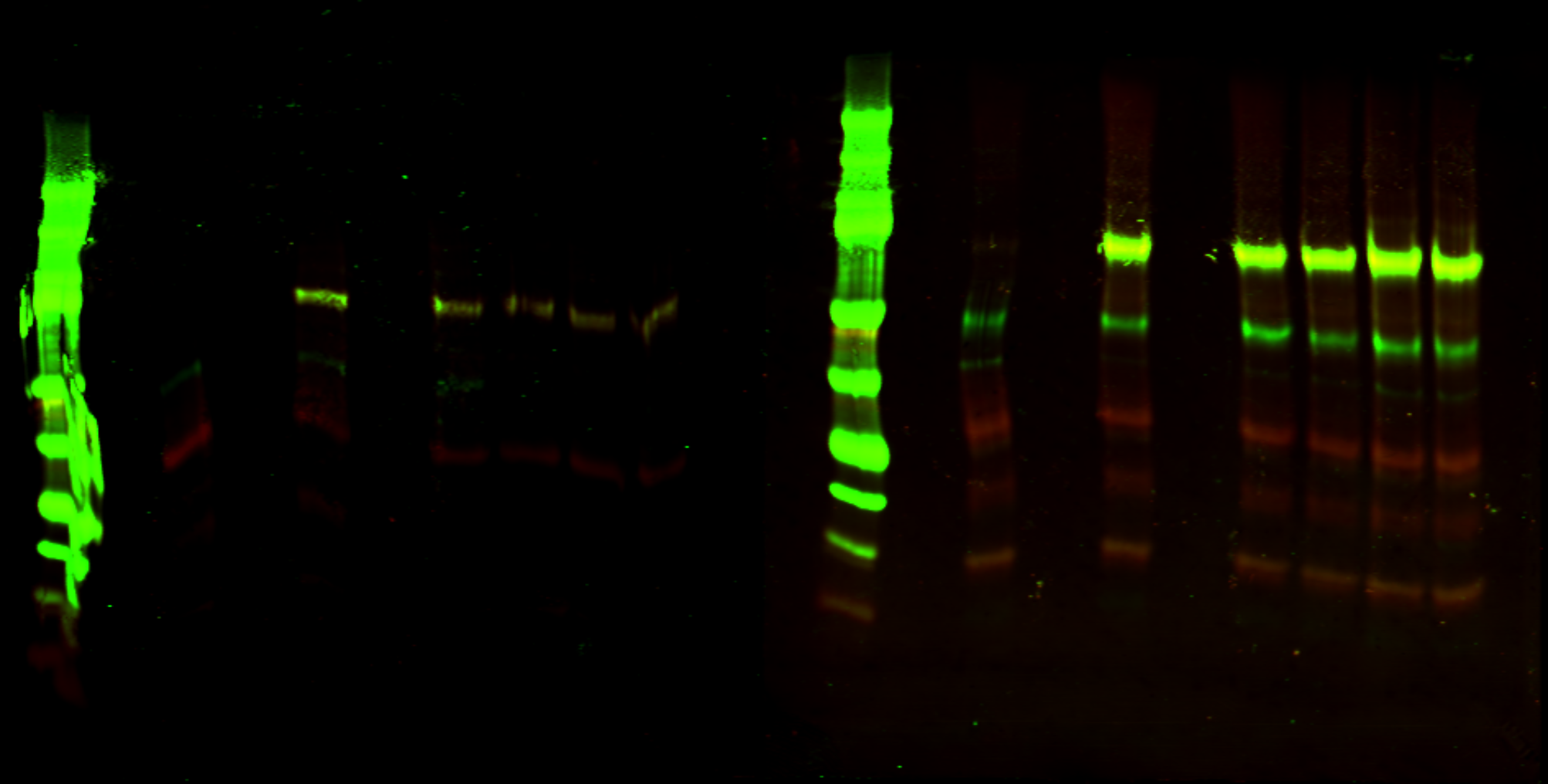

Supplement: Supplementary file 10 — Source data Fig. 6 [file 44319_2024_314_MOESM10_ESM.zip › Fig6_WB/Fig6C/aD174-tetramers-full.png]

## Slide 1
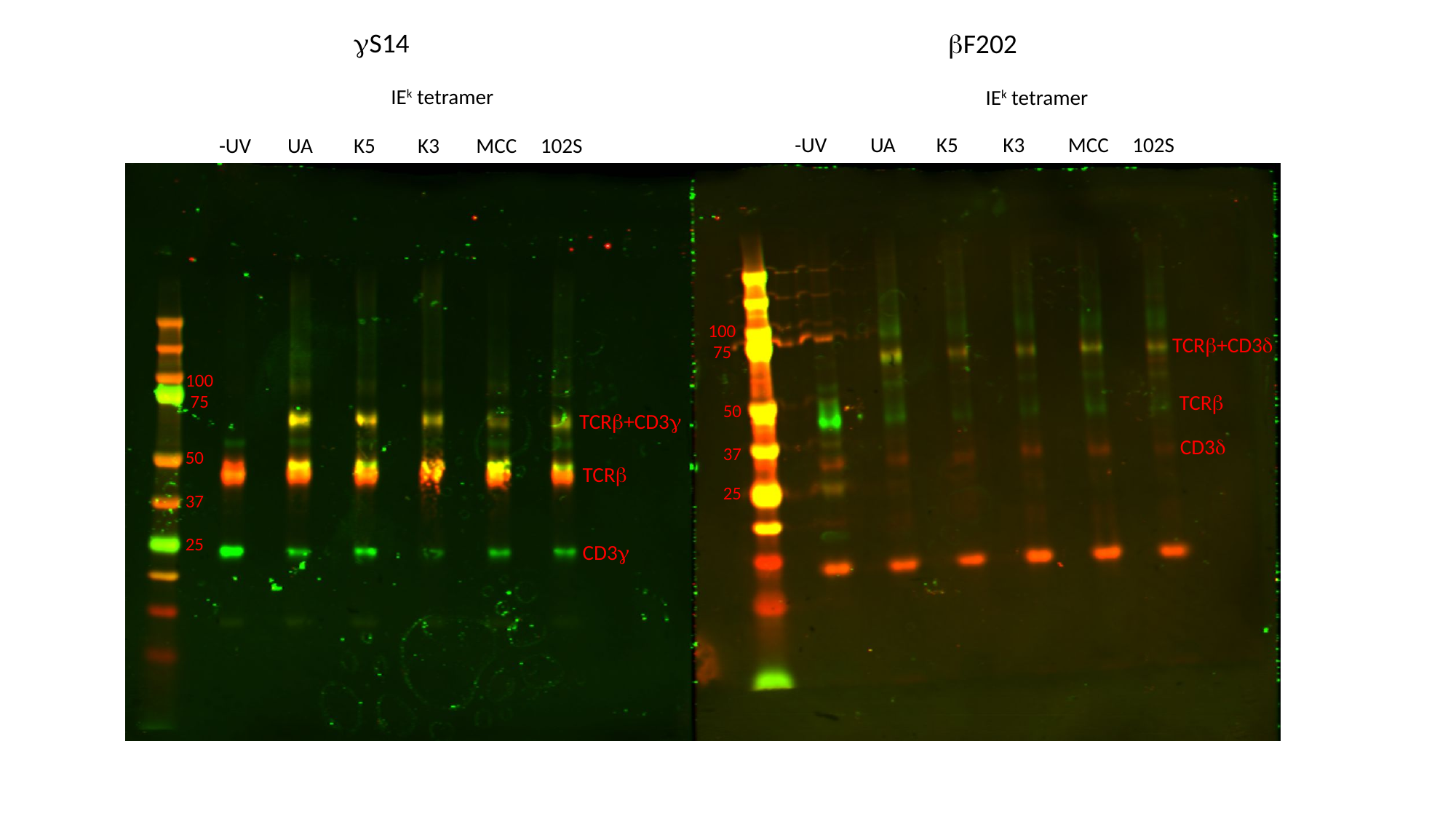

gS14
bF202
IEk tetramer
IEk tetramer
102S
K5
K3
MCC
-UV
UA
102S
K5
K3
MCC
-UV
UA
100
TCRb+CD3d
75
100
TCRb
75
50
TCRb+CD3g
CD3d
37
50
TCRb
25
37
25
CD3g

Supplement: Supplementary file 10 — Source data Fig. 6 [file 44319_2024_314_MOESM10_ESM.zip › Fig6_WB/Fig6C/gS14bF202-tetramers-full-labeled.pptx]

$\alpha$ D174

IE<sup>k</sup> tetramer

-UV

UA

K5

K3

MCC 102S

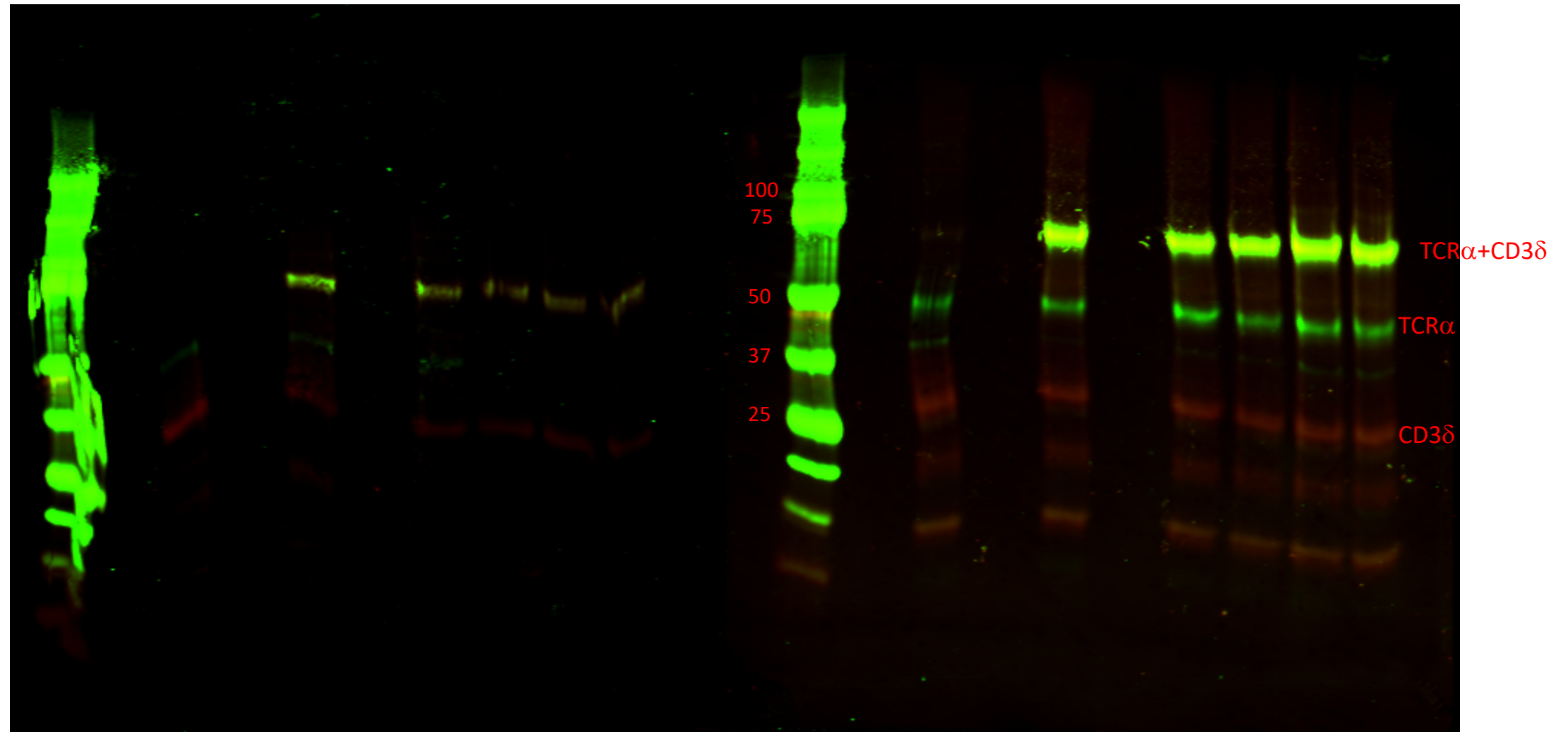

Supplement: Supplementary file 10 — Source data Fig. 6 [file 44319_2024_314_MOESM10_ESM.zip › Fig6_WB/Fig6C/aD174-tetramers-full-labeled.pdf]

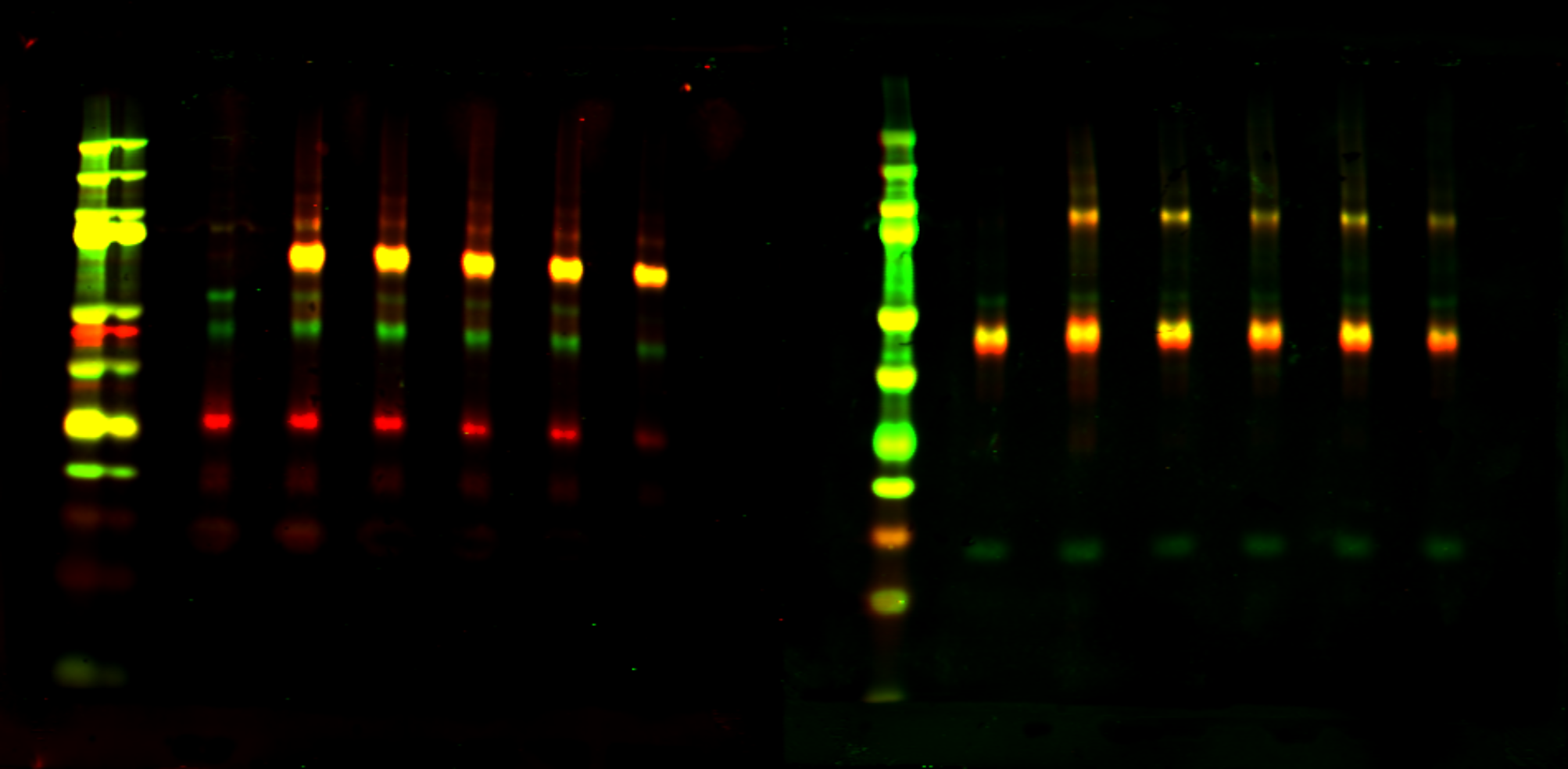

Supplement: Supplementary file 10 — Source data Fig. 6 [file 44319_2024_314_MOESM10_ESM.zip › Fig6_WB/Fig6C/dT35aS138-tetramers-full.png]

$\delta$ T35

$\alpha$ S138

IE<sup>k</sup> tetramer

IE<sup>k</sup> tetramer

-UV    UA    K5    K3    MCC    102S

-UV    UA    K5    K3    MCC    102S

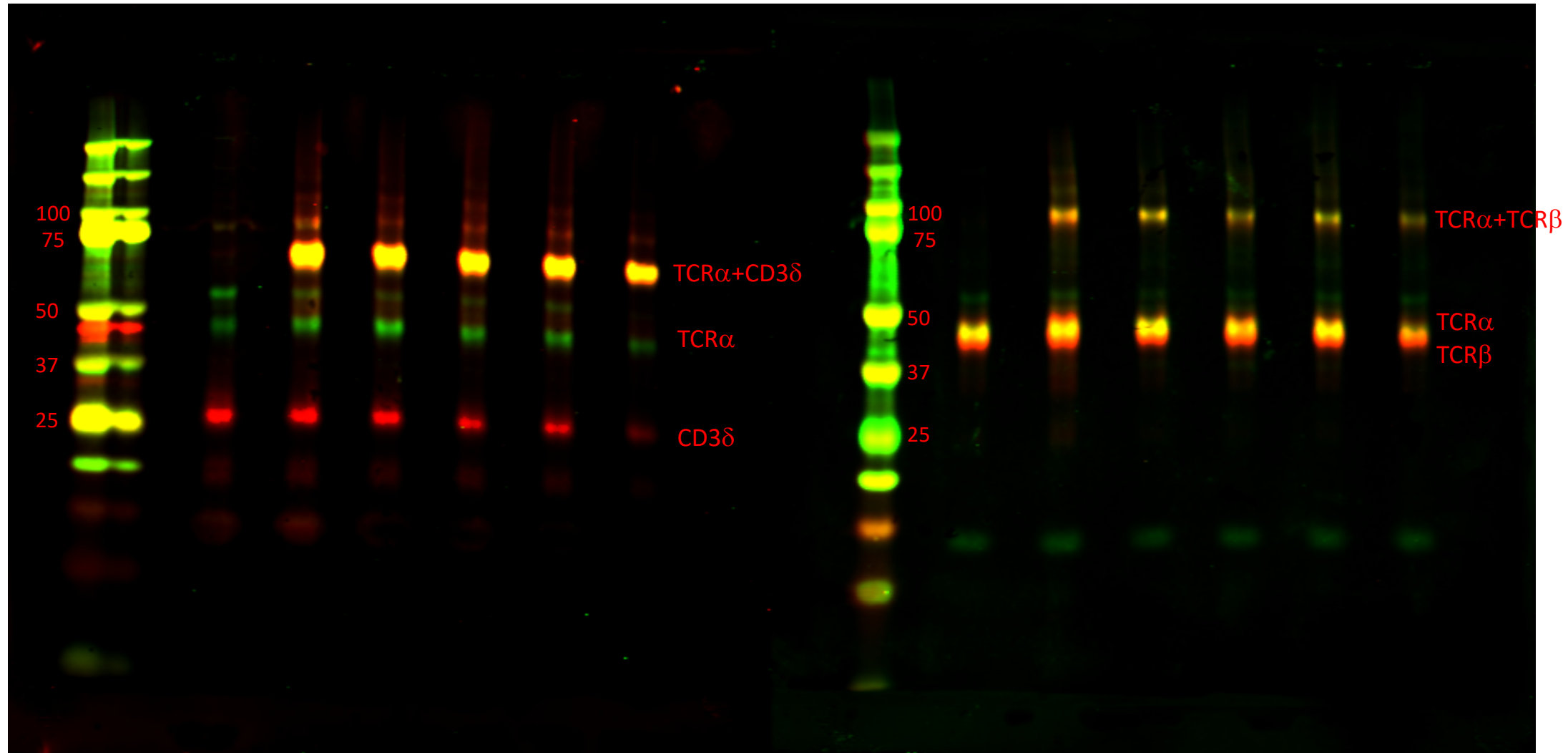

Supplement: Supplementary file 10 — Source data Fig. 6 [file 44319_2024_314_MOESM10_ESM.zip › Fig6_WB/Fig6C/dT35aS138-tetramers-full-labeled.pdf]

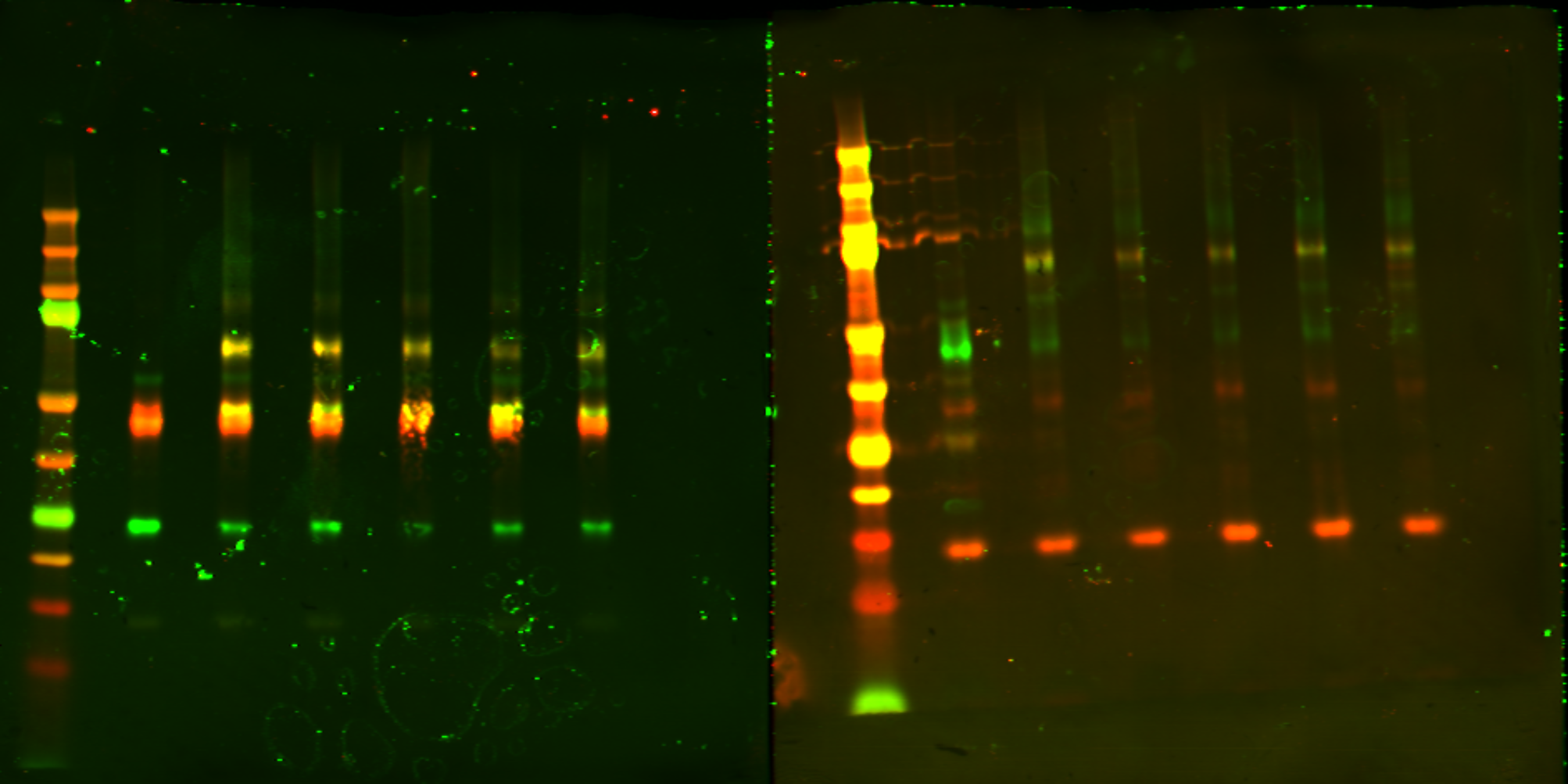

Supplement: Supplementary file 10 — Source data Fig. 6 [file 44319_2024_314_MOESM10_ESM.zip › Fig6_WB/Fig6C/gS14bF202-tetramers-full.png]

## Slide 1
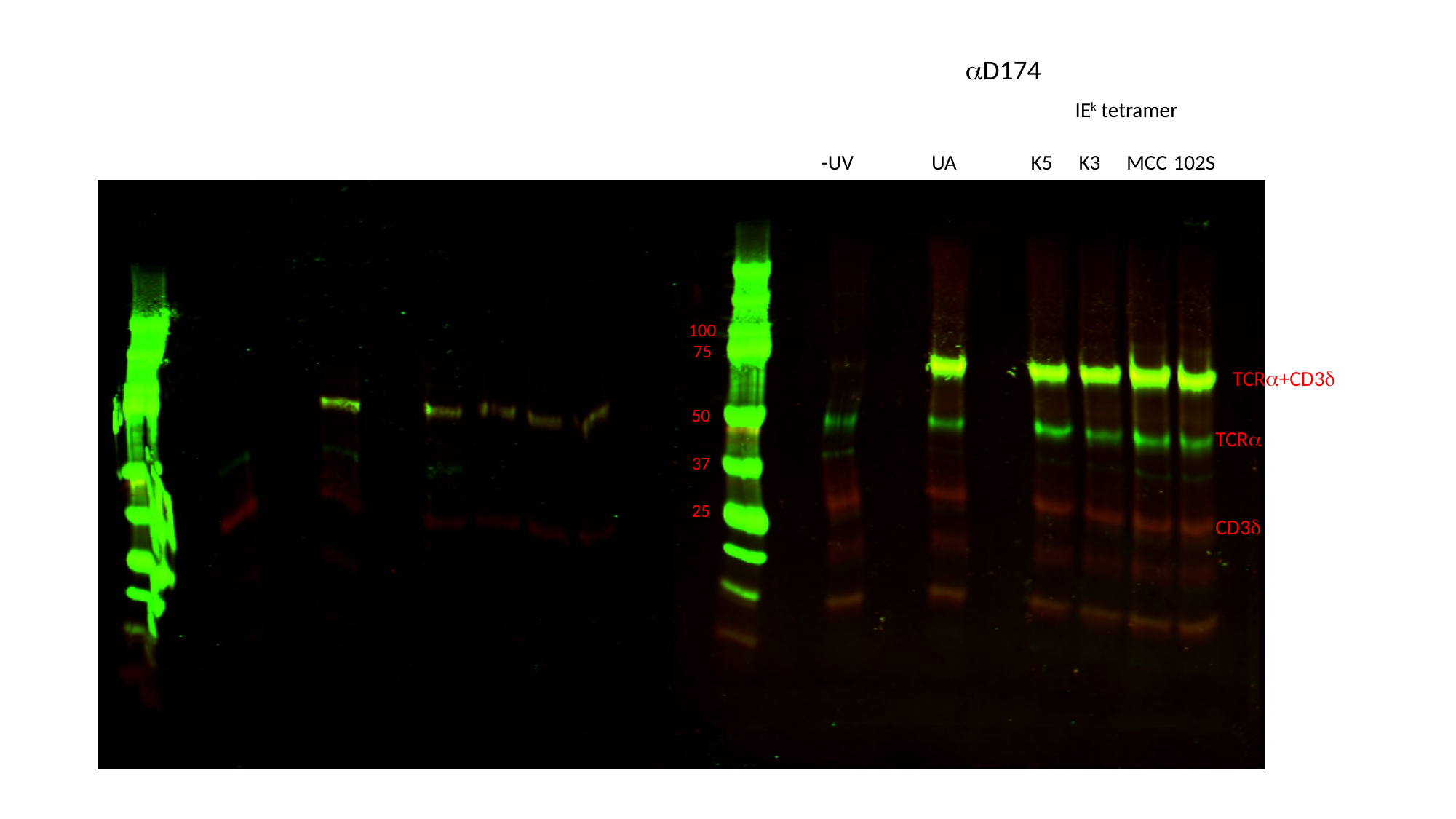

aD174
IEk tetramer
-UV
UA
102S
K5
K3
MCC
100
75
TCRa+CD3d
50
TCRa
37
25
CD3d

Supplement: Supplementary file 10 — Source data Fig. 6 [file 44319_2024_314_MOESM10_ESM.zip › Fig6_WB/Fig6C/aD174-tetramers-full-labeled.pptx]

$\gamma$ S14

$\beta$ F202

IE<sup>k</sup> tetramer

IE<sup>k</sup> tetramer

-UV    UA    K5    K3    MCC    102S

-UV    UA    K5    K3    MCC    102S

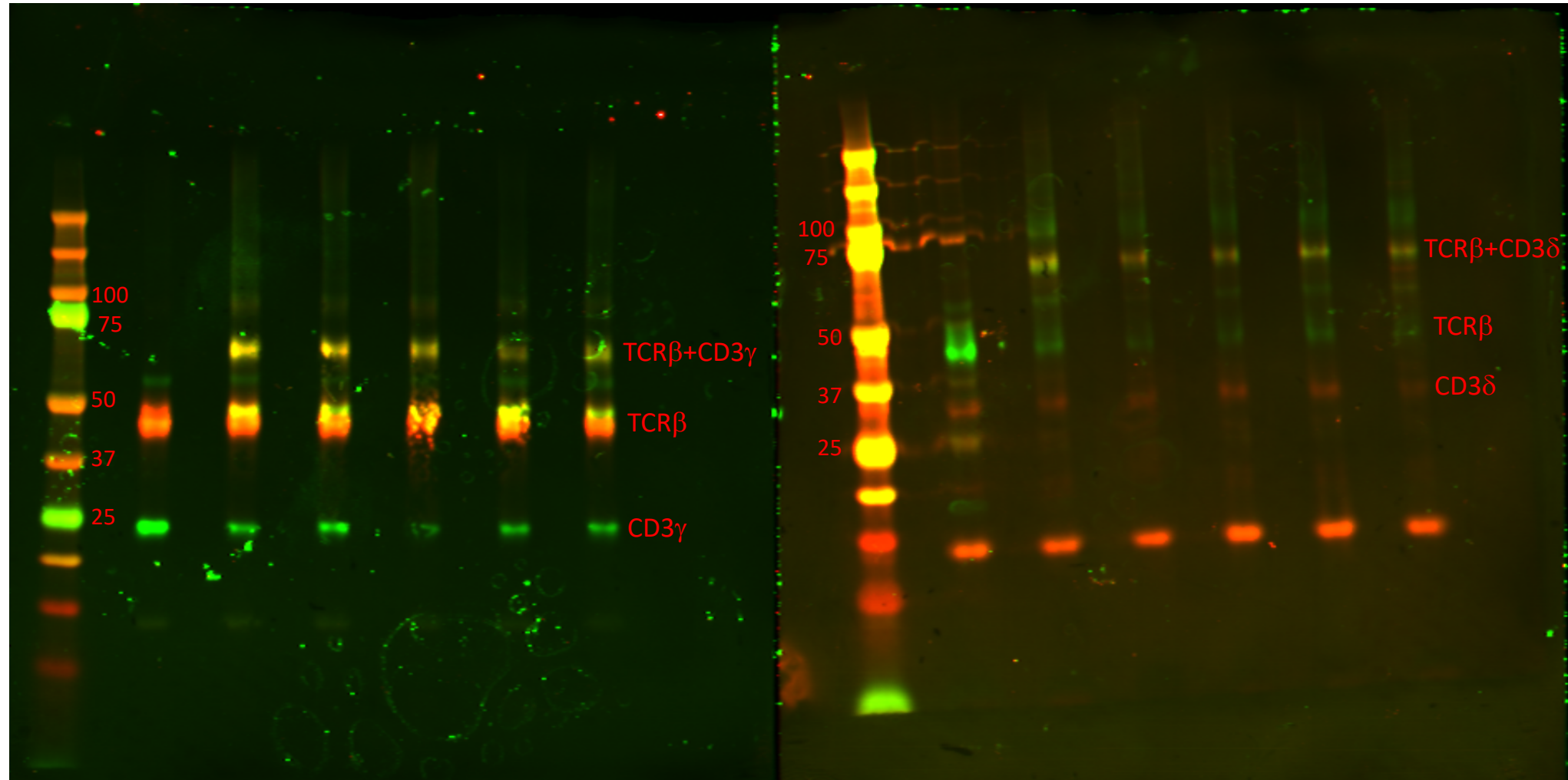

Supplement: Supplementary file 10 — Source data Fig. 6 [file 44319_2024_314_MOESM10_ESM.zip › Fig6_WB/Fig6C/gS14bF202-tetramers-full-labeled.pdf]

## Slide 1
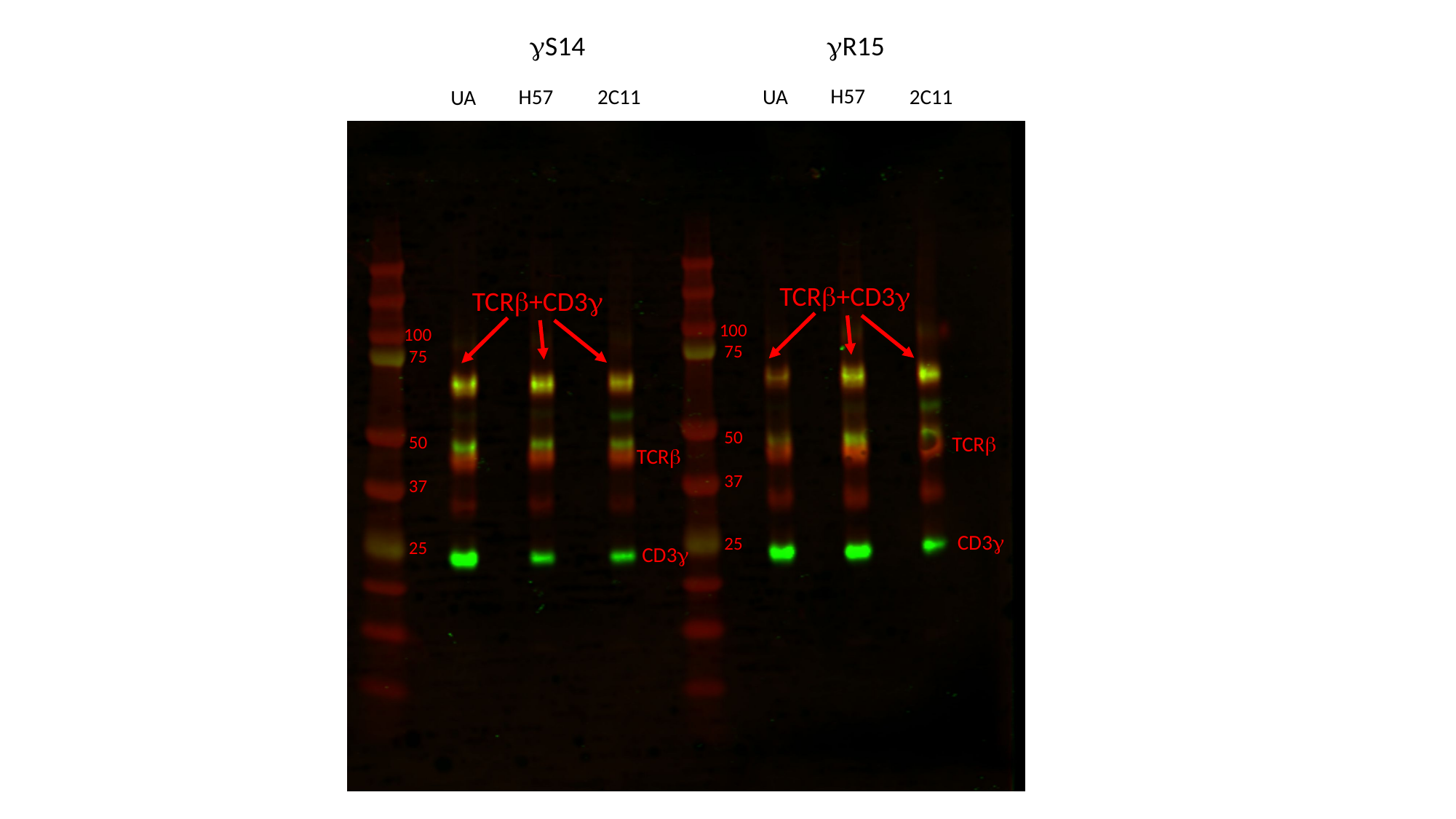

gS14
gR15
H57
UA
H57
2C11
2C11
UA
TCRb+CD3g
TCRb+CD3g
100
100
75
75
50
50
TCRb
TCRb
37
37
CD3g
25
25
CD3g

Supplement: Supplementary file 10 — Source data Fig. 6 [file 44319_2024_314_MOESM10_ESM.zip › Fig6_WB/Fig6B/gS14R15-H57-2C11-full-labeled.pptx]

$\gamma$ S14

$\gamma$ R15

UA

H57

2C11

UA

H57

2C11

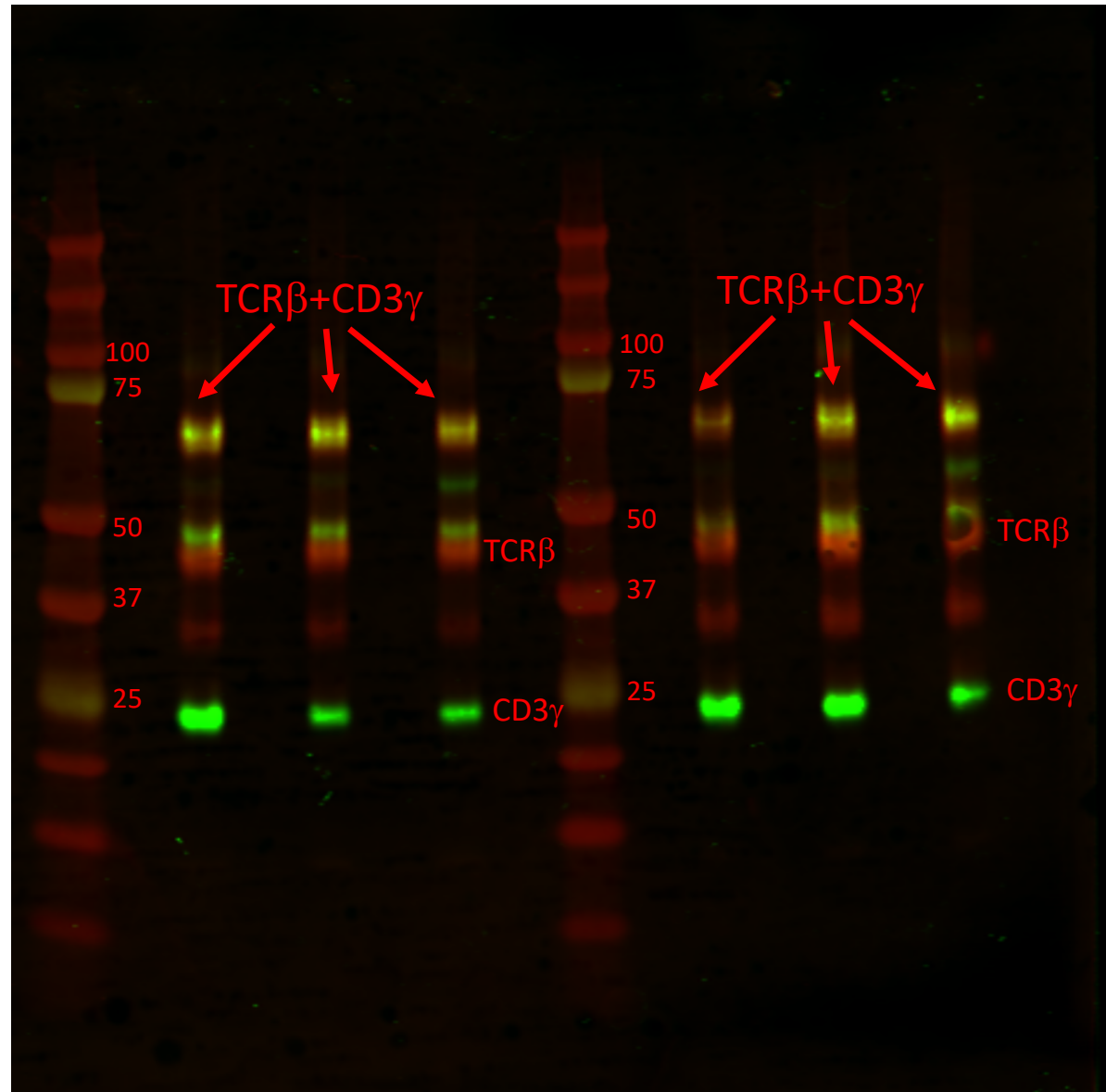

Supplement: Supplementary file 10 — Source data Fig. 6 [file 44319_2024_314_MOESM10_ESM.zip › Fig6_WB/Fig6B/gS14R15-H57-2C11-full-labeled.pdf]

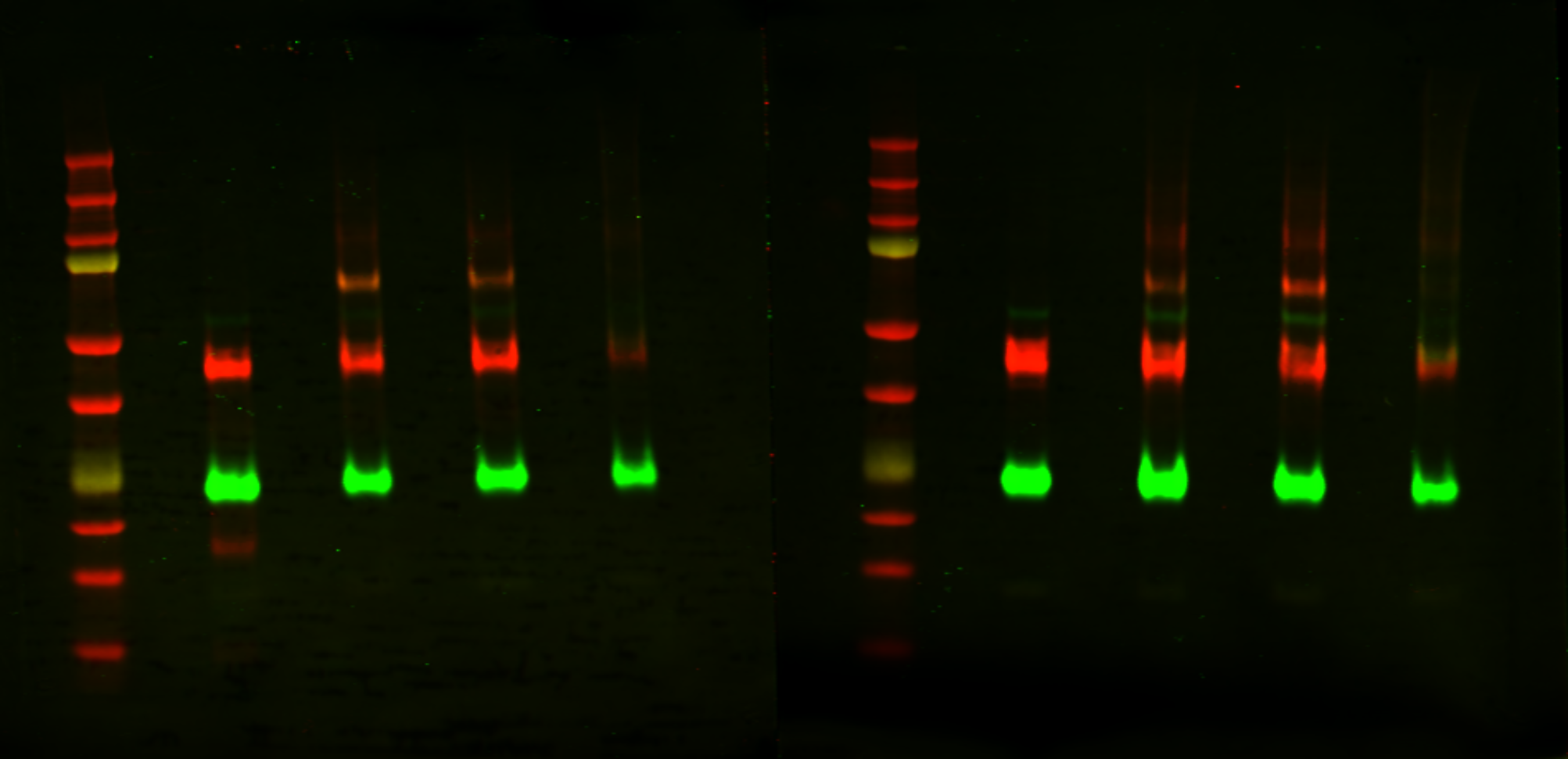

Supplement: Supplementary file 10 — Source data Fig. 6 [file 44319_2024_314_MOESM10_ESM.zip › Fig6_WB/Fig6B/bE221W225-H57-full.png]

$\beta$ E221

$\beta$ W225

-UV

UA

H57

-UV

UA

H57

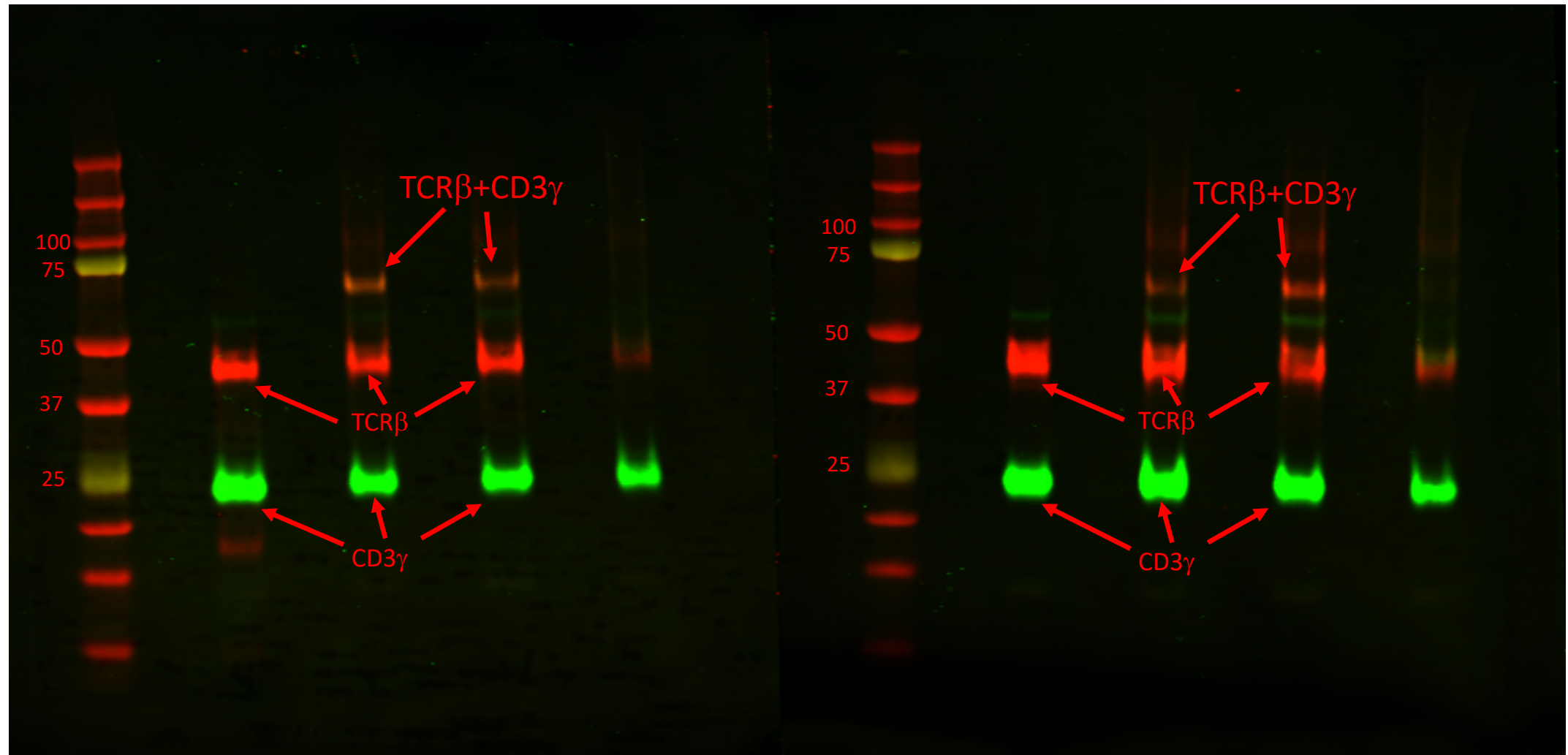

Supplement: Supplementary file 10 — Source data Fig. 6 [file 44319_2024_314_MOESM10_ESM.zip › Fig6_WB/Fig6B/bE221W225-H57-full-labeled.pdf]

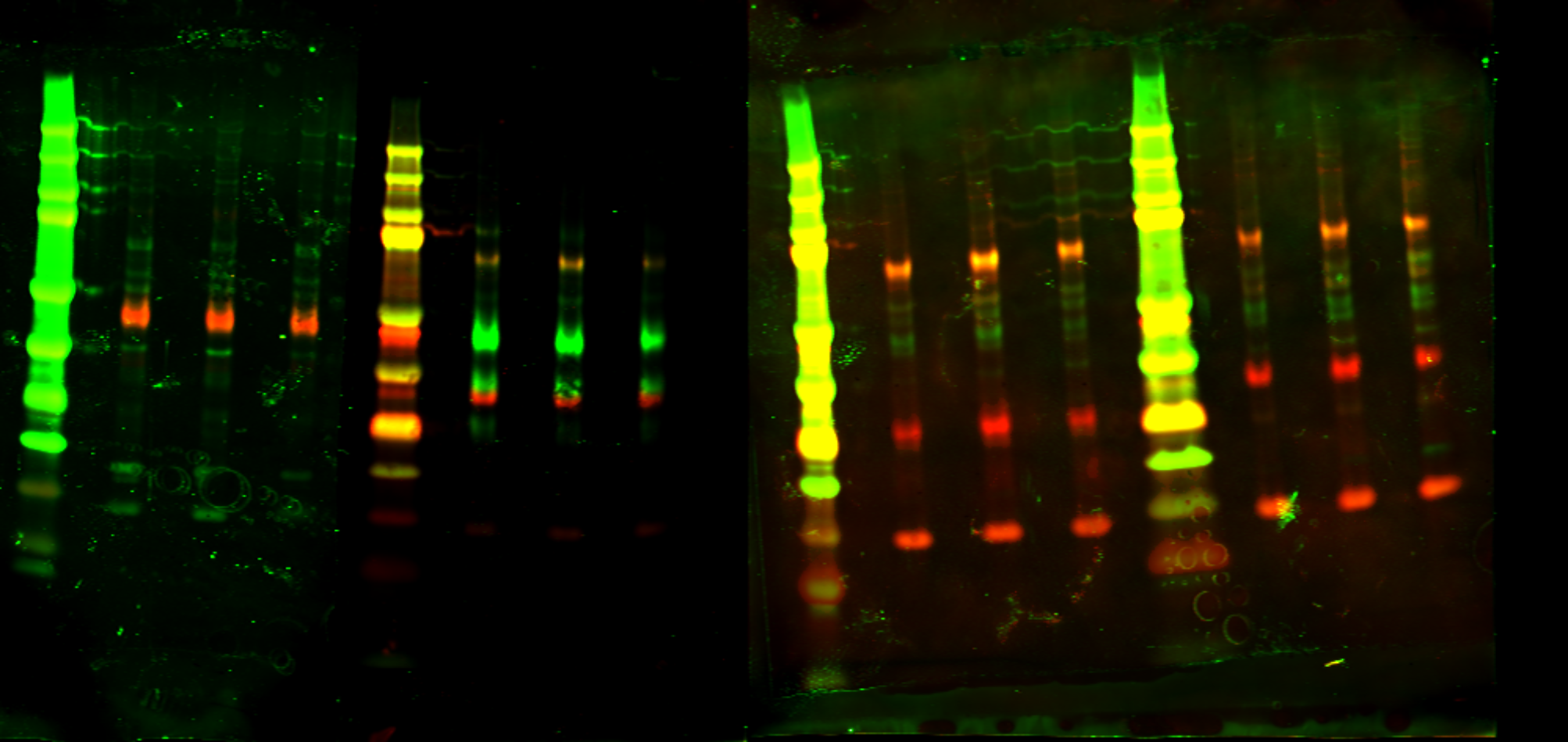

Supplement: Supplementary file 10 — Source data Fig. 6 [file 44319_2024_314_MOESM10_ESM.zip › Fig6_WB/Fig6B/dT5dT35K40-H57-2C11-full.png]

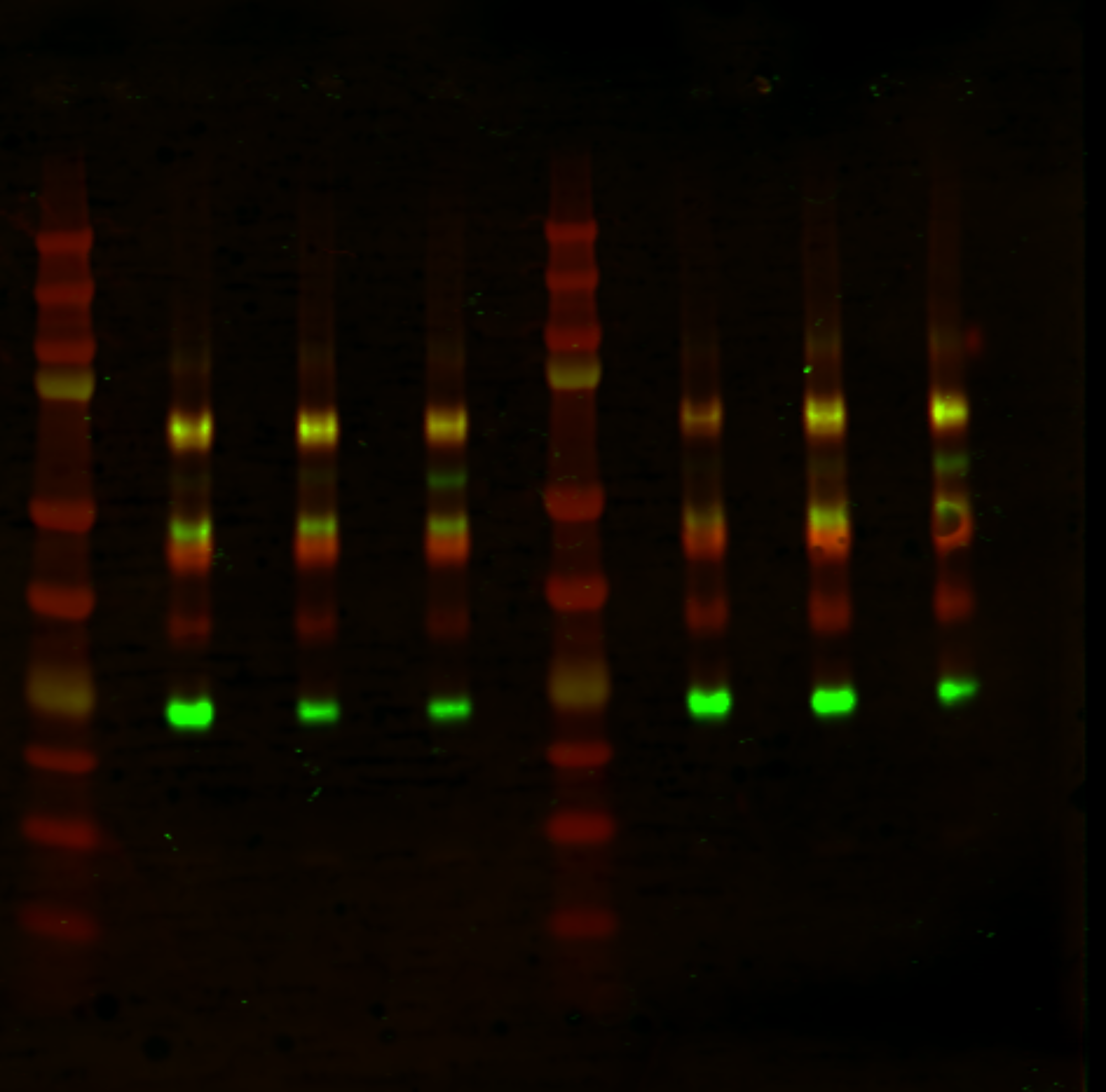

Supplement: Supplementary file 10 — Source data Fig. 6 [file 44319_2024_314_MOESM10_ESM.zip › Fig6_WB/Fig6B/gS14R15-H57-2C11-full.png]

## Slide 1
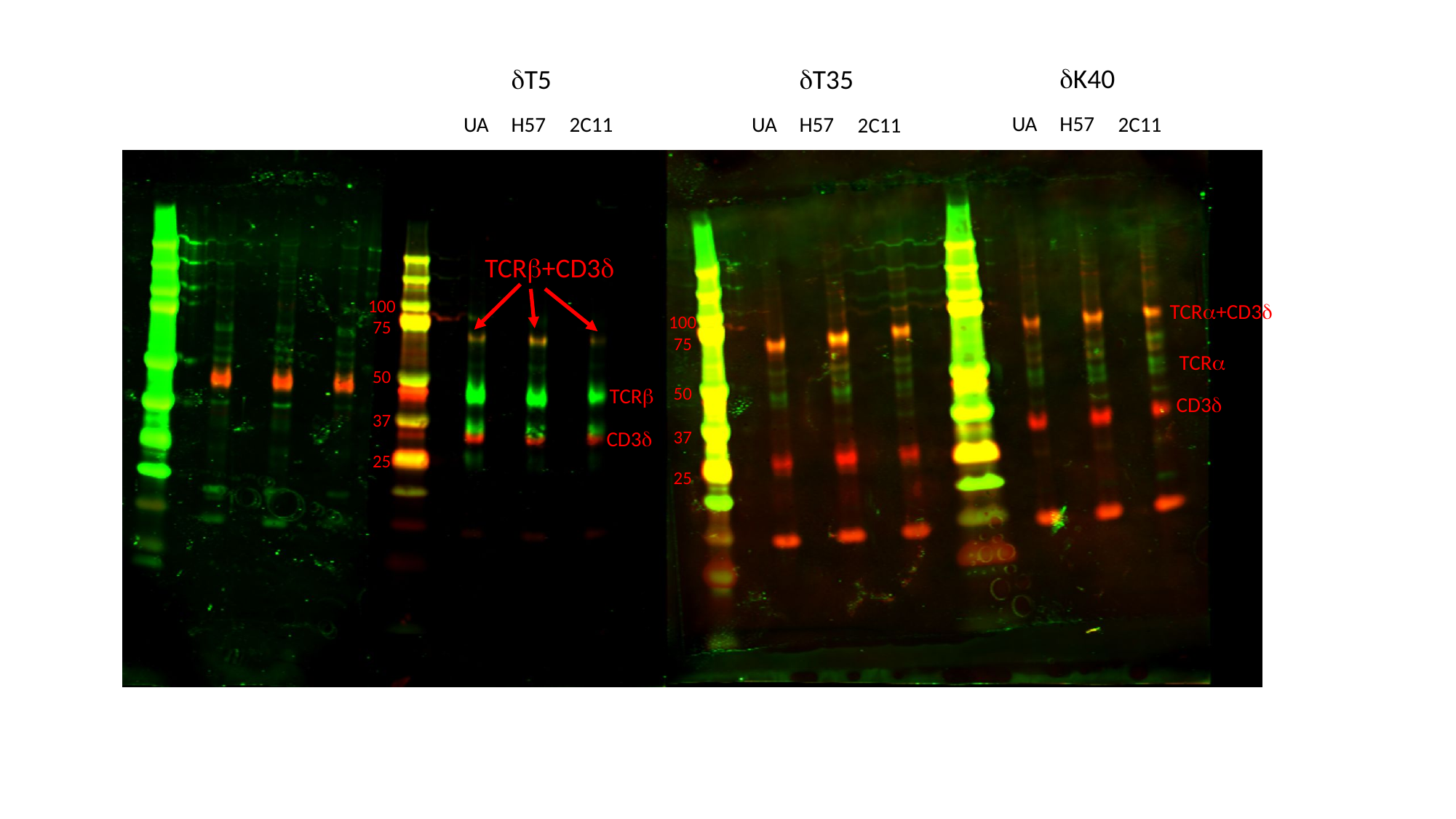

dK40
dT5
dT35
UA
H57
UA
H57
2C11
UA
H57
2C11
2C11
TCRb+CD3d
100
TCRa+CD3d
100
75
75
TCRa
50
50
TCRb
CD3d
37
37
CD3d
25
25

Supplement: Supplementary file 10 — Source data Fig. 6 [file 44319_2024_314_MOESM10_ESM.zip › Fig6_WB/Fig6B/dT5dT35K40-H57-2C11-full-labeled.pptx]

$\delta$ T5

UA H57 2C11

$\delta$ T35

UA H57 2C11

$\delta$ K40

UA H57 2C11

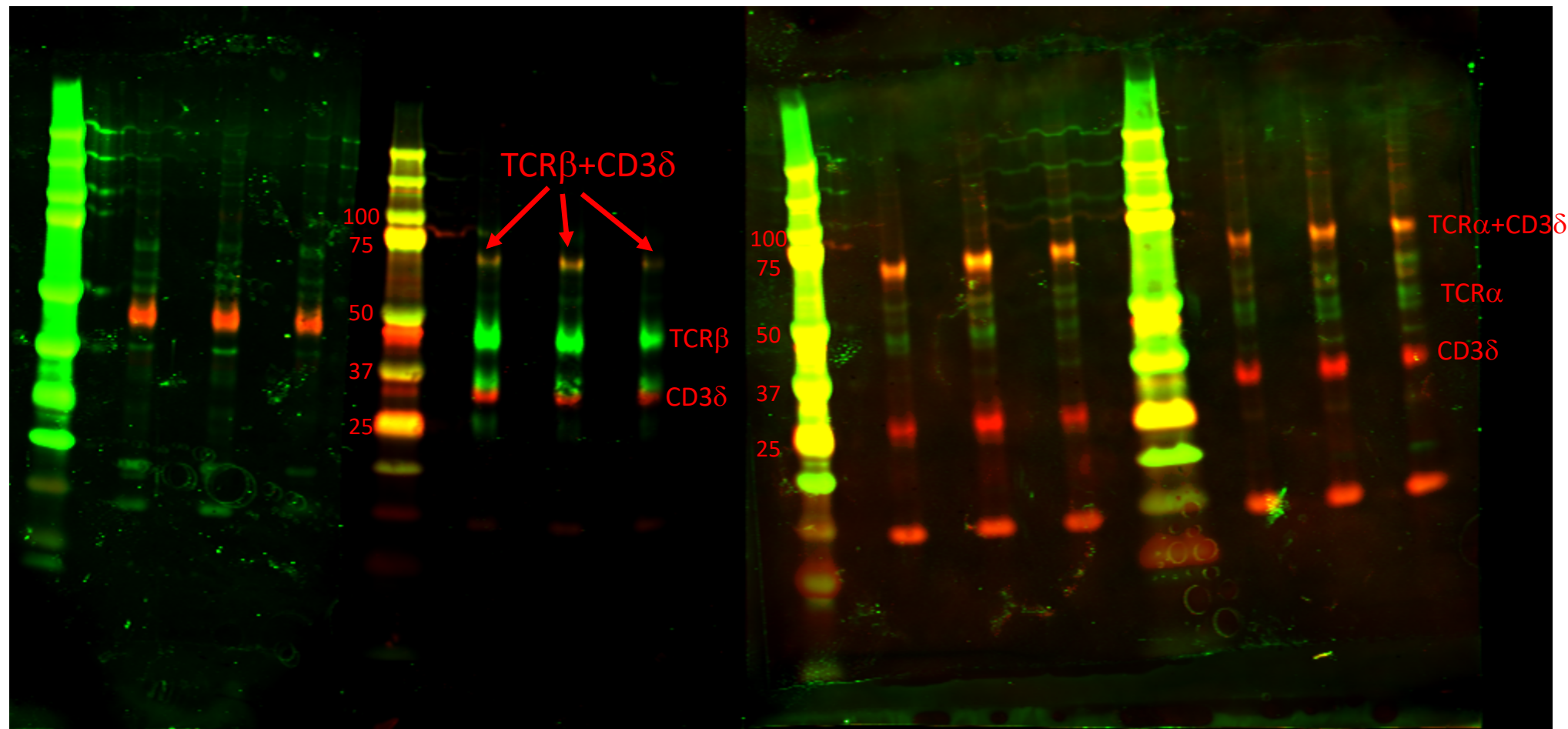

Supplement: Supplementary file 10 — Source data Fig. 6 [file 44319_2024_314_MOESM10_ESM.zip › Fig6_WB/Fig6B/dT5dT35K40-H57-2C11-full-labeled.pdf]

## Slide 1
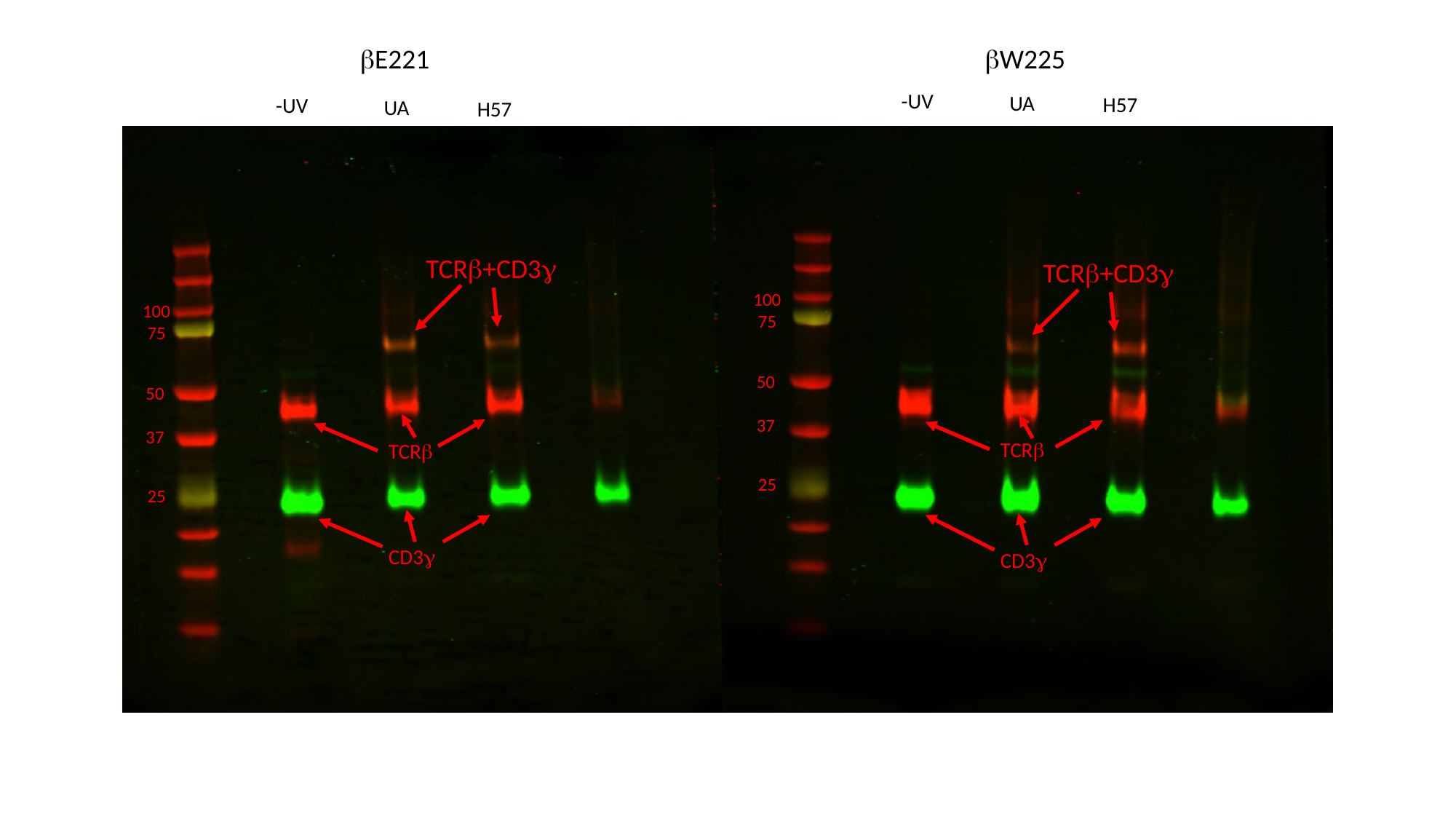

bE221
bW225
-UV
UA
H57
-UV
UA
H57
TCRb+CD3g
TCRb+CD3g
100
100
75
75
50
50
37
37
TCRb
TCRb
25
25
CD3g
CD3g

Supplement: Supplementary file 10 — Source data Fig. 6 [file 44319_2024_314_MOESM10_ESM.zip › Fig6_WB/Fig6B/bE221W225-H57-full-labeled.pptx]
