# Supplementary material for: In situ cell-surface conformation of the TCR-CD3 signaling complex
Source: EMBO Rep. 2024 Nov 7;25(12):26. doi: 10.1038/s44319-024-00314-3 (PMC11624261; doi:10.1038/s44319-024-00314-3)
Supplement: Supplementary file 10 — Source data Fig. 6 [file 44319_2024_314_MOESM10_ESM.zip › Fig6_WB/Fig6C/dT35aS138-tetramers-full-labeled.pptx]

## Slide 1
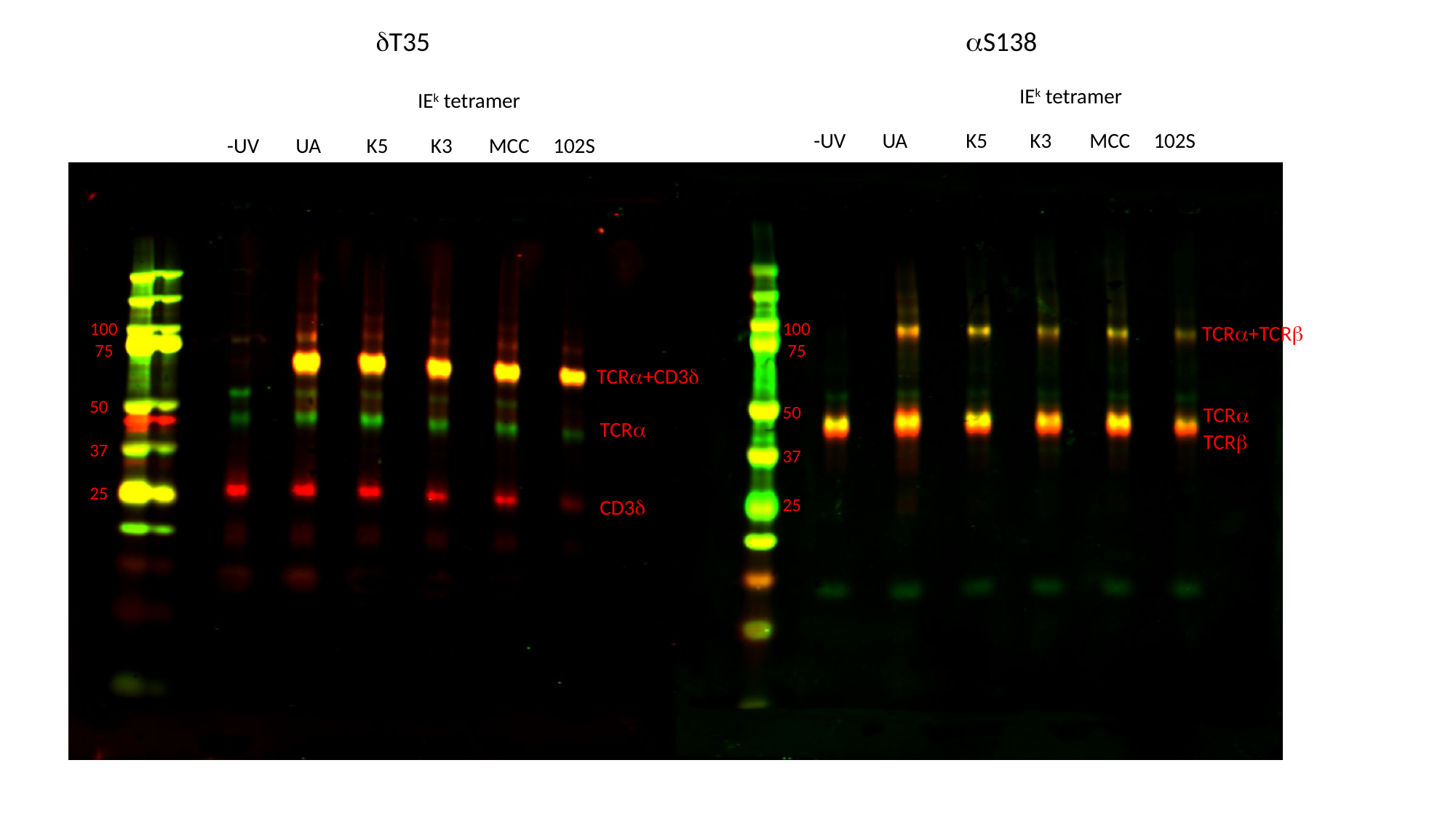

dT35
aS138
IEk tetramer
IEk tetramer
102S
K5
K3
MCC
-UV
UA
102S
K5
K3
MCC
-UV
UA
100
100
TCRa+TCRb
75
75
TCRa+CD3d
50
50
TCRa
TCRa
TCRb
37
37
25
25
CD3d
