# Supplementary material for: In situ cell-surface conformation of the TCR-CD3 signaling complex
Source: EMBO Rep. 2024 Nov 7;25(12):26. doi: 10.1038/s44319-024-00314-3 (PMC11624261; doi:10.1038/s44319-024-00314-3)
Supplement: Supplementary file 12 — Expanded View Figures [file 44319_2024_314_MOESM12_ESM.pdf]

Expanded View Figures

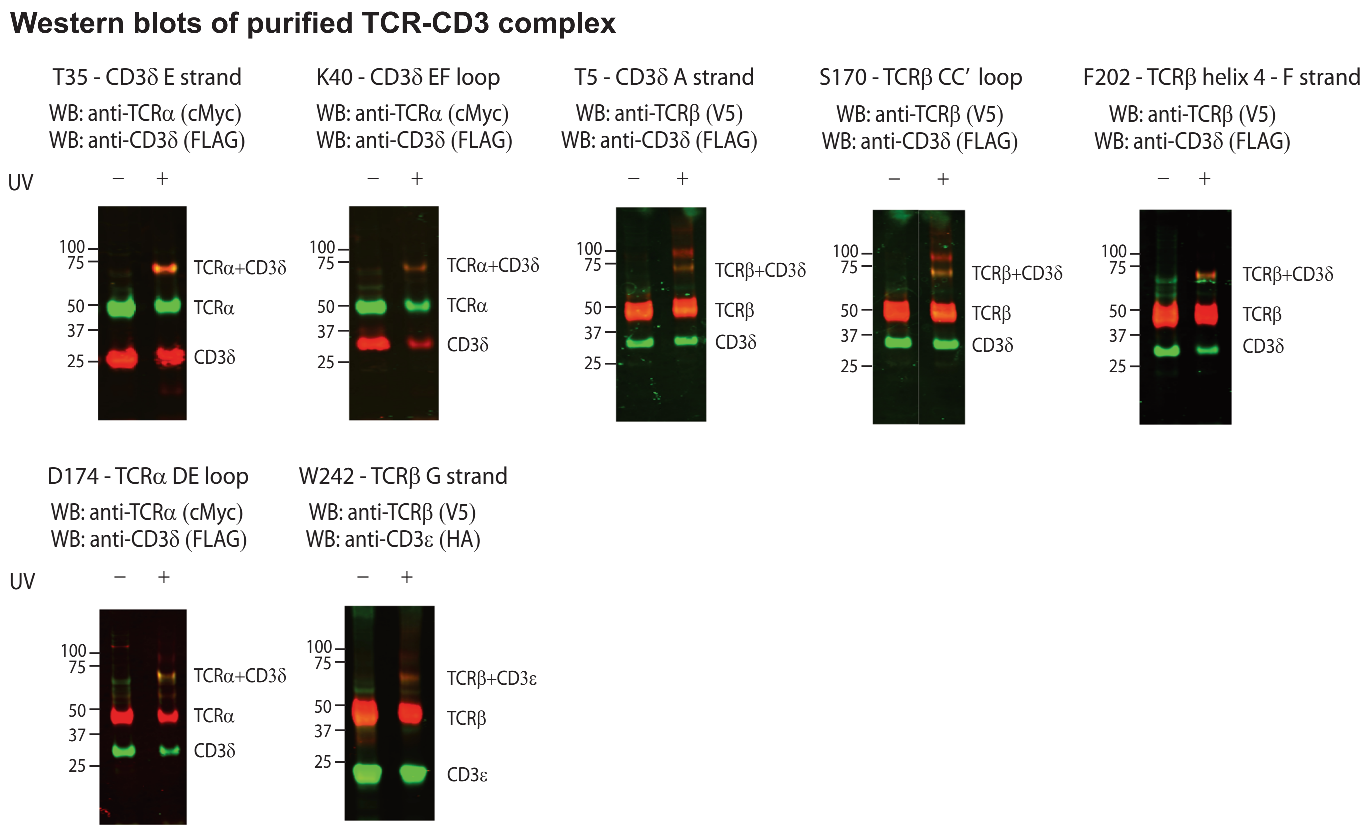

Figure EV1. Crosslinking in purified TCR-CD3 complex.

Western blots of purified mutant TCR-CD3 complexes—δT35, δK40, δT5, βS170, βF202, αD174, and βW242. TCRα + CD3δ crosslinked bands for δT35, δK40 and αD174 are present below 75 kDa. These blots were stained with anti-TCRα (cMyc) antibody and anti-CD3δ (FLAG). TCRβ + CD3δ crosslinked bands for δT5, βS170 and βF202 are present below 75 kDa. These blots were stained with anti-TCRβ (V5) antibody and anti-CD3δ (FLAG). TCRβ + CD3ε crosslinked band for βW242 is present below 75 kDa. These blots were stained with anti-TCRβ (V5) antibody and anti-CD3ε (HA). Anti-rabbit IRDye 680LT- and anti-mouse IRDye 800CW were used as secondary antibodies for detection for all blots.

© The Author(s)

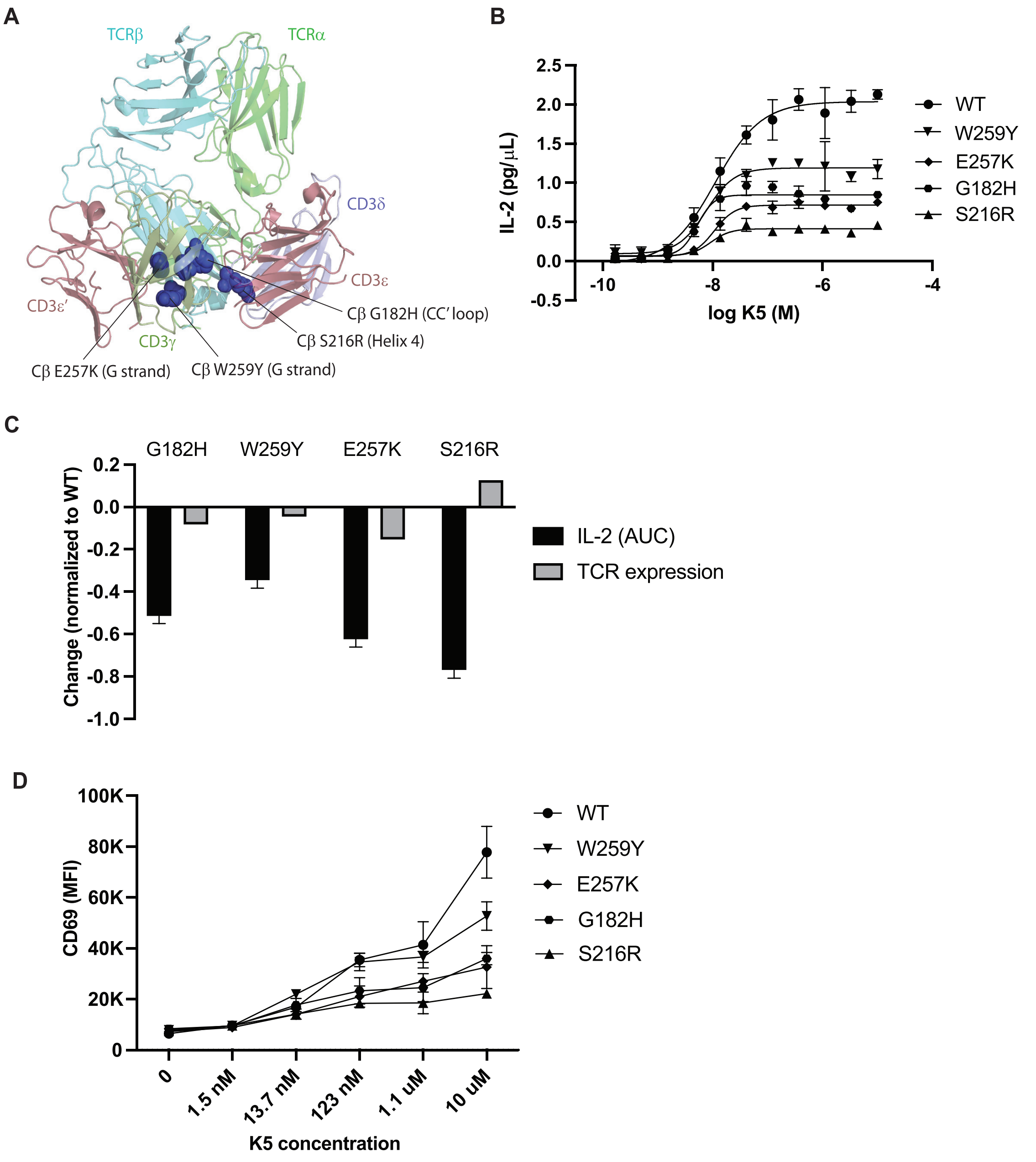

Figure EV2. Stabilizing TCR-CD3 extracellular interactions reduces TCR signaling.

(**A**) Locations of the mutated residues indicated on the human TCR-CD3 cryo-EM structure (PDB: 6JXR). $G_{182}H$ is located in the Cβ CC′ loop, $E_{257}K$ and $W_{259}Y$ are located in the Cβ G strand and $S_{216}R$ is located in Cβ Helix 4. (**B**) ELISA assays (plot of IL-2 produced vs concentration of activating peptide) for mutant 2B4 T cell hybridoma clones activated with CHO/I-E^k/K5 in biological replicates ($n = 3$ for each peptide concentration). (**C**) Change in the area under the curve for IL-2 production (black) between the indicated mutant T cell hybridoma and wild type 2B4 hybridoma when activated with CHO cells expressing the cognate pMHC IE^k/K5 calculated based on (**B**). Change in TCR expression when compared to the wild type 2B4 TCR expression (MFI) is plotted in gray. (**D**) Plots of CD69 expression (MFI) on Jurkats expressing 2B4 mutant and wild type TCR when activated with indicated concentrations of K5 peptide (CHO/I-E^k/K5) in biological replicates ($n = 3$ for each peptide concentration) for 16 h. Data information: In (**B**, **D**), data are presented as mean ± SD. In (**C**), area under the curves is represented as mean ± SEM. For (**D**), Appendix Table S7 shows non-parametric unpaired t-test values for wild type-mutant comparisons.

 © The Author(s)
